# Supplementary material for: Visible-light promoted late-stage chlorination and bromination of quinones and (hetero)arenes utilizing aqueous HCl or HBr as halogen donors
Source: Commun Chem. 2025 Dec 11;9:23. doi: 10.1038/s42004-025-01831-5 (PMC12804898; doi:10.1038/s42004-025-01831-5)
Supplement: Supplementary file 2 — Supplementary Information [file 42004_2025_1831_MOESM2_ESM.pdf]

Supporting Information

Visible-light promoted late-stage chlorination and bromination of quinones and (hetero)arenes utilizing aqueous HCl or HBr as halogen donors

Yangyang Zhang, Jinglian Nong, and Yaxin Wang*

College of Pharmacy, Nanjing University of Chinese Medicine, Nanjing 210023, China.

Content

1. **Reagents**......S2
2. **Instruments**......S2
3. **Substrates of the study**......S2
4. **Reaction optimization for C-H chlorination and bromination of naproxen**......S6
5. **General procedures and substrate scope for C(sp^2)-H chlorination of quinones and (hetero)arenes**......S8
6. **General procedures and substrate scope for C(sp^2)-H bromination of quinones and (hetero)arenes**......S20
7. **Visible-light-promoted C(sp^2)-H chlorination and bromination of electron-poor arenes and benzene**......S33
8. **Synthetic applications**......S34
9. **Mechanistic studies**......S36
10. **References**......S45

1. Reagents

All commercial materials were used as received unless otherwise noted. Solvents and deuterated solvents were purchased from *J&K* Chemical. Starting materials were synthesized according to reported procedures. Aqueous HCl (37% in water, GENERAL-REAGENT Titan), aqueous HBr (40% in water, GENERAL-REAGENT Titan), and $NaNO_2$ (99.99%, Adamas-beta) were used in the chlorination and bromination of quinones and (hetero)arenes. TLC were performed on silica gel Huanghai HSGF254 plates and visualization of the developed chromatogram was performed by fluorescence quenching (λ_{max} = 254 nm). Flash chromatography was performed using silica gel (200-300 mesh) purchased from Qingdao Haiyang Chemical Co., China.

2. Instruments

NMR spectra were recorded on Bruker AVANCE AV 500 instruments and all NMR experiments were reported in units, parts per million (ppm), using residual solvent peaks as internal reference. Multiplicities are recorded as: s = singlet, d = doublet, t = triplet, dd = doublet of doublets, td = triplet of doublets, br = broad singlet, m = multiplet. High resolution ESI mass experiments were operated on a Waters LCT Premier instrument. All reactions were carried out in a 4 mL glass vial (Thermo SCIENTIFIC National B7999-2, made from superior quality 33 expansion borosilicate clear glass), sealed with a PTEF cap on bench top if necessay.

Lights: PHILIPS TORNADO 24W CFL, Cnlight 220V/24W UV (254nm), Cnlight 220V/24W UV (365nm), Cnlight LED lights 24 W (red LED, yellow LED, green LED, blue LED, purple LED, white LED) were used in the screening conditions of chlorination and bromination of quinones and (hetero)arenes. Cnlight blue LED 24 W was used in the chlorination and bromination of quinones and (hetero)arenes.

3. Substrates for the study

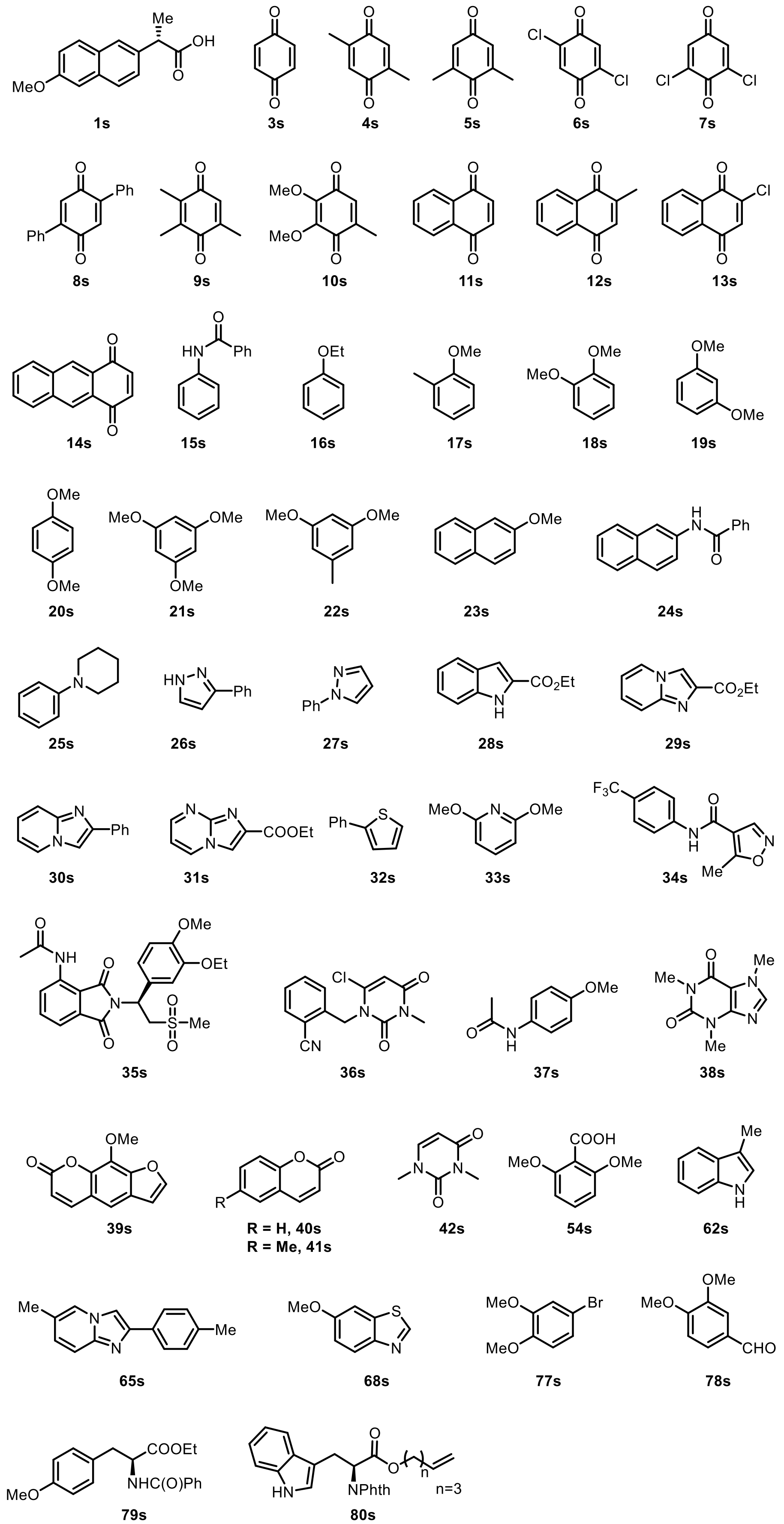

Scheme S1. Substrates for this study

Compounds **15s**, **24s**, **37s, 78s and 79s** were known compounds and synthesized following the reported procedures. Other compounds for this study were commercial available and used as received.

3.1 Synthesis of compounds 15s and 24s

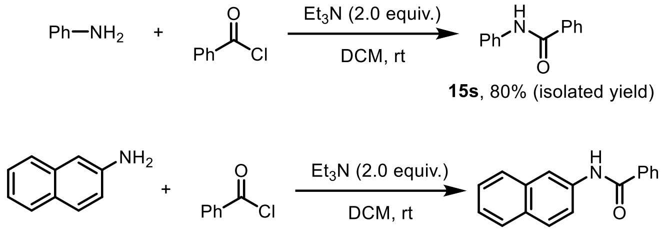

Scheme S2. Synthesis of compound **15s** and **24s**

A solution of primary amine (10.0 mmol, 1.0 equiv) and triethylamine (Et_3N, 20.0 mmol, 2.0 equiv) in dichloromethane (DCM, 50.0 mL) was cooled to 0 °C, and benzoyl chloride (10.0 mmol, 1.0 equiv) was subsequently added dropwise. The reaction mixture was stirred at 0 °C for 1 hour and then allowed to warm to room temperature and stirred for an additional 5 hours. The reaction was quenched by pouring the mixture into water (75.0 mL) and the resulting mixture was extracted with dichloromethane (CH_2Cl_2, 3 × 50 mL). The combined organic layers were dried over anhydrous sodium sulfate (Na_2SO_4), filtered, and concentrated in vacuo. The crude product was purified by silica gel column chromatography, eluting with a mixture of petroleum ether and ethyl acetate (v/v 1:1), to afford the desired compounds **15s** and **24s**. Compounds **5s** and **24s** are known compounds and their spectra data are consistent with those reported in the literature.[1]

3.2 Synthesis of compound 37s and 78s

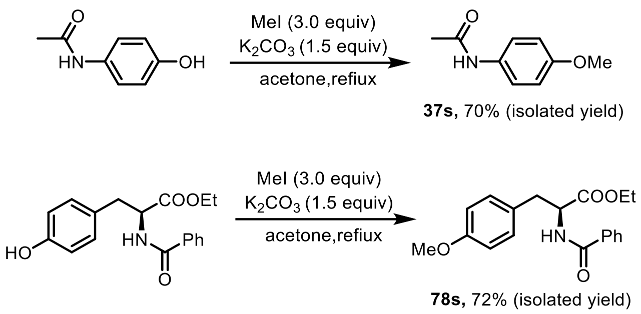

Scheme S3. Synthesis of compound **37s** and **78s**

Phenol derivative (10 mmol, 1.0 equiv), potassium carbonate (K_2CO_3, 15.0 mmol, 1.5 equiv), and anhydrous acetone (40 mL) were sequentially added in a 100 mL round-bottom flask. Subsequently, a solution of methyl iodide (MeI, 30.0 mmol, 3.0 equiv) in anhydrous acetone (40.0 mL) was added dropwise to the reaction mixture at room temperature. After stirring at room temperature for 30 minutes, the reaction mixture was refluxed for 16 hours. The reaction mixture was then cooled to room temperature and filtered to remove the inorganic salts. The filtrate was concentrated under reduced pressure to obtain the crude product. The crude product was purified by silica gel column chromatography, eluting with a mixture of petroleum ether and ethyl acetate (v/v 2:1), to afford the desired compounds **37s** and **78s**. Compounds **37s** and **78s** are known compounds and their spectra data are consistent with those reported in the literature.[2]

3.3 Synthesis of compound 79s

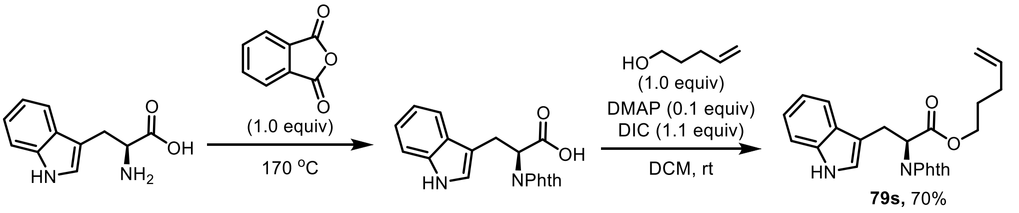

Scheme S4. Synthesis of compound **79s**

L-tryptophan (27.0 mmol, 1.0 equiv) and phthalic anhydride (27.0 mmol, 1.0 equiv) were added in a 100 mL round-bottom flask. The mixture was then heated to 170 °C

and stirred for 2 hours. After cooling to room temperature, the reaction mixture was poured into H_2O (50.0 mL) and extracted with CH_2Cl_2 (3 × 50 mL). The combined organic layers were dried over anhydrous Na_2SO_4, filtered, and concentrated under reduced pressure to afford the corresponding acid, which was used directly in the next step without further purification.

A solution of the *N*-phthaloyl L-tryptophan (10.0 mmol, 1.0 equiv) in DCM (50.0 mL) was treated with *N*, *N'*-diisopropylcarbodiimide (DIC) (11.0 mmol, 1.1 equiv), 4-dimethylaminopyridine (DMAP) (1.0 mmol, 0.1 equiv), and 4-penten-1-ol (10.0 mmol, 1.0 equiv). The reaction mixture was stirred for 12 hours. Subsequently, the mixture was poured into H_2O (100.0 mL) and extracted with CH_2Cl_2 (3 × 50 mL). The combined organic layers were washed with an aqueous KOH solution (0.2 M, 100.0 mL) and water, dried over anhydrous Na_2SO_4, filtered, and concentrated under reduced pressure. Finally, the crude product was purified by silica gel column chromatography, eluting with a mixture of petroleum ether and ethyl acetate (v/v 4:1), to afford the desired compound **79s**. Compound **79s** is known compounds and its spectra data are consistent with those reported in the literature.[3,4]

4. Reaction optimization for C-H chlorination and bromination of naproxen

All screening reactions were carried out at a 0.1 mmol scale in a 4 mL glass vial (Thermo Scientific, National B7999-2) sealed with PTEF cap and stirred on bench top. A 24 W LED light was positioned 5 cm aside from the reaction vials if necessary. naproxen (0.1 mmol, 1.0 equiv) and $NaNO_2$ were first dispersed in specific solvent and stirred for 1 min at room temperature. Specified acid was then added and the resulting mixture was vigorously stirred at room temperature (24 °C) with or without light irradiation for several hours. After removal of the solvent *in vacuo*, the resulting residue was dissolved in 1 mL of $CDCl_3$ along with $Cl_2CHCHCl_2$ (20 μL) as an internal standard for ^{1}H-NMR analysis.

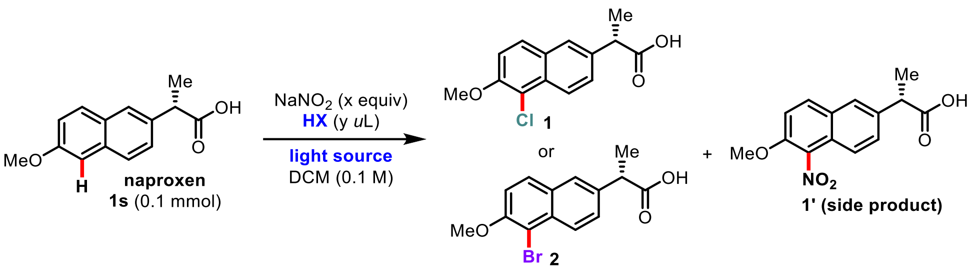

Entry	reagents (equiv), HCl (37% aq.) or HBr (40% aq.) (uL), light	**1** % or **2** %	**1’** %
1	NaNO2 (0.2), HCl (12), Blue LEDs (460-470nm)	26 (**1**)	<10
2	NaNO2 (0.5), HCl (30), Blue LEDs (460-470nm)	60 (**1**)	<10
3	NaNO2 (1.0), HCl (60), Blue LEDs (460-470nm)	86 (75[b]) (**1**)	<10
4	NaNO2 (1.0), HCl (50), Blue LEDs (460-470nm)	85 (75[b]) (**1**)	<10
5	NaNO2 (1.0), HCl (30), Blue LEDs (460-470nm)	29 (**1**)	<10
6	NaNO2 (1.0), HCl (10), Blue LEDs (460-470nm)	13 (**1**)	<10
7	NaNO2 (2.0), HCl (50), Blue LEDs (460-470nm)	51 (**1**)	24
8	NaNO2 (1.0), HCl (50), White CFL	68 (**1**)	<10
9	NaNO2 (1.0), HCl (50), Green LEDs (510-520nm)	69 (**1**)	<10
10	NaNO2 (1.0), HCl (50), Red LEDs (620-630nm)	37 (**1**)	<10
11	NaNO2 (1.0), HCl (50), UV (365 nm)	26 (**1**)	<10
12[c]	NaNO2 (1.0), HCl (50), in darkness	<10 (**1**)	11
13	HCl (60), 12 h, Blue LEDs (460-470nm)	0 (**1**)	0
14	NaNO2 (1.0), HBr (50) , Blue LEDs (460-470nm)	90 (80[b]) (**2**)	<10

[a]All reactions were carried out on a 0.1 mmol scale in solvent dichloromethane (DCM) (1 mL) at room temperature (24 °C) under light (24 W) irradiation for 10 hours, and yields are based on crude ^{1}H NMR analysis on a 0.1 mmol scale reaction mixture. HCl (37% aqueous) and HBr (40% aqueous). [b]Isolated yield. [c]In darkness.

Table S1. Reaction optimization for the chlorination and bromination of naproxen **1**

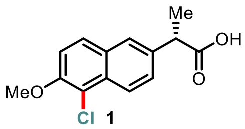

^{1}H NMR (500 MHz, $CDCl_3$) δ 8.18 (d, J = 8.7 Hz, 1H), 7.73 (d, J = 9.0 Hz, 1H), 7.70 (s, 1H), 7.54 (d, J = 8.9 Hz, 1H), 7.29 (d, J = 9.0 Hz, 1H), 4.02 (s, 3H), 3.90 (q, J = 7.2 Hz, 1H), 1.60 (d, J = 7.2 Hz, 3H). **^{13}C NMR** (126 MHz, $CDCl_3$) δ 180.57, 152.73, 135.77, 131.30, 129.57, 127.99, 127.51, 126.53, 124.25, 116.93, 114.13, 57.09, 45.23, 18.17. The spectra data are consistent with those reported in literature.[6]

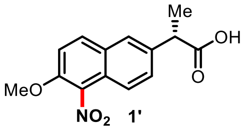

1'

^{1}H NMR (500 MHz, $CDCl_3$) δ 8.19 (d, J = 8.8 Hz, 1H), 7.75 (d, J = 8.8 Hz, 2H), 7.58 (d, J = 8.7 Hz, 1H), 7.30 (d, J = 9.0 Hz, 1H), 5.06 (q, J = 6.5 Hz, 1H), 4.03 (s, 3H), 1.57 (d, J = 6.4 Hz, 3H). Compound **1'** is known compounds and the spectra data are consistent with those reported in literature.[5]

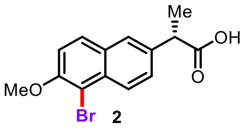

2

^{1}H NMR (500 MHz, $CDCl_3$) δ 8.18 (d, J = 8.8 Hz, 1H), 7.76 (d, J = 9.0 Hz, 1H), 7.69 (s, 1H), 7.53 (d, J = 8.8 Hz, 1H), 7.25 (d, J = 8.9 Hz, 1H), 4.01 (s, 3H), 3.90 (q, J = 7.1 Hz, 1H), 1.60 (d, J = 7.1 Hz, 3H). **^{13}C NMR** (126 MHz, $CDCl_3$) δ 179.98, 153.96, 135.85, 132.58, 129.87, 128.99, 127.77, 126.90, 126.54, 114.08, 108.65, 57.19, 45.12, 18.21. **HRMS** Calcd for $C_{14}H_{13}BrNaO_3^+$ [M+Na$^+$]: 330.9940; Found: 330.9941.

5. General procedures and substrate scope for C-H chlorination of quinones and (hetero)arenes

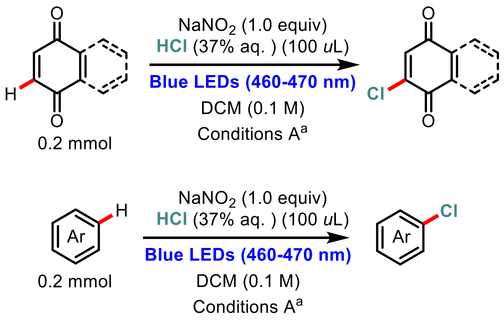

Scheme S5. C-H chlorination reactions of quinones and hetero(arenes)

General conditions A: Substrate (0.2 mmol, 1.0 equiv), $NaNO_2$ (0.2 mmol, 1.0 equiv) and 37% aqueous HCl (100 *u*L) were dispersed in CH_2Cl_2 (2.0 mL) in a 4 mL glass vial at room temperature. The reaction vial was sealed with a PTEF cap and the reaction mixture vigorously stirred at room temperature (24 °C) under the 24 W blue LEDs (460-470 nm) irradiation for 10 hours (It is worth noting that 24 W blue LEDs was positioned 5 cm aside from the reaction vials.). Then, the reaction mixture was extracted with CH_2Cl_2 (3 × 2 mL). The combined organic layer was dried over anhydrous Na_2SO_4, filtered and concentrated. The residue was purified by chromatography on silica gel to afford the desired chlorination products.

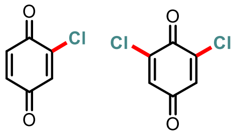

3 **3'**

Compound **3** was isolated in 56% yield and compound **3'** was isolated in 32% yield following the general conditions A. The spectra of compound **3:** **^{1}H NMR** (500 MHz, $CDCl_3$) δ 7.02 (s, 1H), 6.92 (d, *J* = 10.1 Hz, 1H), 6.81 (d, *J* = 10.1 Hz, 1H). **^{13}C NMR** (126 MHz, $CDCl_3$) δ 185.08, 179.38, 144.30, 136.93, 136.19, 133.86. The spectra data are consistent with those reported in literature.[7] The spectra of compound **3':** **^{1}H NMR** (500 MHz, $CDCl_3$) δ 7.03 (s, 2H). **^{13}C NMR** (126 MHz, $CDCl_3$) δ 182.63, 172.87, 143.75, 133.97. The spectra data are consistent with those reported in literature.[8]
However, upon precisely reducing the aqueous HCl loading to 50 uL (3.0 equiv), monochlorinated benzoquinone **3** is obtained as the sole isolable product in 87% isolated yield.

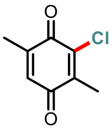

4

Compound **4** was isolated in 90% yield following the general conditions A. **^{1}H NMR** (500 MHz, $CDCl_3$) δ 6.64 (q, *J* = 1.7 Hz, 1H), 2.17 (s, 3H), 2.10 (d, *J* = 1.6 Hz, 3H). **^{13}C NMR** (126 MHz, $CDCl_3$) δ 184.98, 180.05, 145.66, 142.47, 140.93, 133.45, 16.34,

13.71. The spectra data are consistent with those reported in literature.[8]

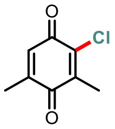

5

Compound **5** was isolated in 89% yield following the general conditions A. **^{1}H NMR** (500 MHz, $CDCl_3$) δ 6.70 (q, *J* = 1.6 Hz, 1H), 2.19 (s, 3H), 2.08 (d, *J* = 1.7 Hz, 3H). **^{13}C NMR** (126 MHz, $CDCl_3$) δ 185.39, 179.43, 146.32, 142.57, 140.78, 132.73, 16.14, 13.98. The spectra data are consistent with those reported in literature.[8]

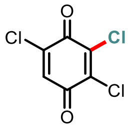

6

Compound **6** was isolated in 92% yield following the general conditions A. **^{1}H NMR** (500 MHz, $CDCl_3$) δ 7.18 (s, 1H). **^{13}C NMR** (126 MHz, $CDCl_3$) δ 175.53, 171.23, 144.07, 141.68, 140.76, 133.18. The spectra data are consistent with those reported in literature.[9]

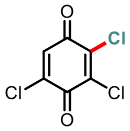

7

Compound **7** was isolated in 92% yield following the general conditions A. **^{1}H NMR** (500 MHz, $CDCl_3$) δ 7.17 (s, 1H). **^{13}C NMR** (126 MHz, $CDCl_3$) δ 175.51, 171.21, 144.05, 141.66, 140.75, 133.18. The spectra data are consistent with those reported in literature.[9]

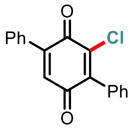

8

Compound **8** was isolated in 89% yield following the general conditions A. **^{1}H NMR** (500 MHz, $CDCl_3$) δ 7.55 (d, *J* = 7.2 Hz, 2H), 7.53 – 7.45 (m, 6H), 7.38 – 7.32 (m, 2H), 7.00 (s, 1H). The spectra data are consistent with those reported in literature.[10]

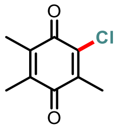

9

Compound **9** was isolated in 80% yield following the general conditions A. **^{1}H NMR** (500 MHz, $CDCl_3$) δ 2.18 (s, 3H), 2.08 (s, 3H), 2.05 (s, 3H). **^{13}C NMR** (126 MHz, $CDCl_3$) δ 185.15, 179.76, 142.09, 141.27, 140.79, 140.39, 13.98, 12.97, 12.80. The spectra data are consistent with those reported in literature.[11]

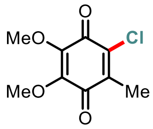

10

Compound **10** was isolated in 75% yield following the general conditions A. **^{1}H NMR** (500 MHz, $CDCl_3$) δ 4.02 (s, 3H), 3.99 (s, 3H), 2.16 (s, 3H). **^{13}C NMR** (126 MHz, $CDCl_3$) δ 181.67, 176.80, 145.38, 144.43, 140.24, 138.73, 61.65, 61.45, 13.65. **HRMS** Calcd for $C_9H_{10}ClO_4^+$ [$M+H^+$]: 217.0262; Found: 217.0263.

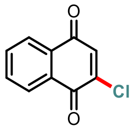

11

Compound **11** was isolated in 90% yield following the general conditions A. **^{1}H NMR** (500 MHz, $CDCl_3$) δ 8.21 – 8.13 (m, 1H), 8.11 – 8.06 (m, 1H), 7.82 – 7.73 (m, 2H), 7.22 (s, 1H). **^{13}C NMR** (126 MHz, $CDCl_3$) δ 182.80, 178.09, 146.47, 136.05, 134.64, 134.28, 131.89, 131.43, 127.65, 126.89. The spectra data are consistent with those reported in literature.[8]

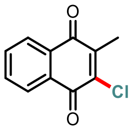

12

Compound **12** was isolated in 73% yield following the general conditions A. ^{1}**H NMR** (500 MHz, $CDCl_3$) δ 8.17 – 8.13 (m, 1H), 8.13 – 8.08 (m, 1H), 7.77 – 7.69 (m, 2H), 2.34 (s, 3H). ^{13}**C NMR** (126 MHz, $CDCl_3$) δ 182.71, 177.67, 145.00, 143.44, 134.27, 134.04, 131.81, 131.49, 127.29, 127.11, 14.60. The spectra data are consistent with those reported in literature.[12]

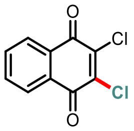

13

Compound **13** was isolated in 93% yield following the general conditions A. ^{1}**H NMR** (500 MHz, $CDCl_3$) δ 8.18 (dd, *J* = 5.7, 3.3 Hz, 2H), 7.80 (dd, *J* = 5.8, 3.3 Hz, 2H). ^{13}**C NMR** (126 MHz, $CDCl_3$) δ 176.16, 143.67, 134.80, 131.06, 127.96. **GC-MS** Calcd for $C_{10}H_4Cl_2O_2$ [M]: 225.9590; Found: 225.9000.

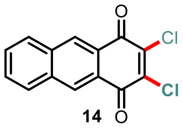

14

Compound **14** was isolated in 38% yield following the general conditions A (the recovery of starting material is 52%). ^{1}**H NMR** (500 MHz, $CDCl_3$) δ 8.83 (s, 2H), 8.14 (dd, *J* = 6.2, 3.3 Hz, 2H), 7.81 (dd, *J* = 6.3, 3.2 Hz, 2H). ^{13}**C NMR** (126 MHz, $CDCl_3$) δ 179.23, 135.65, 132.71, 130.99, 130.34, 124.92. The spectra data are consistent with those reported in literature.[13]

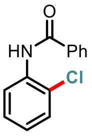

15

Compound **15** was isolated in 45% yield following the general conditions A (the recovery of starting material is 50%). ^{1}**H NMR** (500 MHz, $CDCl_3$) δ 8.60 – 8.52 (m,

1H), 8.42 (d, *J* = 32.8 Hz, 1H), 7.95 – 7.88 (m, 2H), 7.62 – 7.56 (m, 1H), 7.53 (t, *J* = 7.6 Hz, 2H), 7.42 (d, *J* = 8.1 Hz, 1H), 7.38 – 7.29 (m, 1H), 7.09 (t, *J* = 7.8 Hz, 1H). **^{13}C NMR** (126 MHz, $CDCl_3$) δ 165.43, 134.90, 134.78, 132.54, 132.35, 129.35, 129.17, 129.11, 128.90, 128.22, 128.05, 127.24, 127.23, 124.89, 123.63, 123.15, 122.32, 121.63. The spectra data are consistent with those reported in literature.[14]

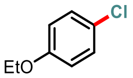

16

Compound **16** was isolated in 71% yield following the general conditions A. **^{1}H NMR** (500 MHz, $CDCl_3$) δ 7.22 (d, *J* = 8.7 Hz, 2H), 6.82 (d, *J* = 8.4 Hz, 2H), 4.00 (q, *J* = 7.0 Hz, 2H), 1.41 (t, *J* = 7.0 Hz, 3H). **^{13}C NMR** (126 MHz, $CDCl_3$) δ 157.69, 129.40, 125.47, 115.87, 63.87, 14.89. The spectra data are consistent with those reported in literature.[6]

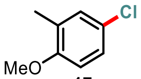

17

Compound **17** was isolated in 80% yield following the general conditions A. **^{1}H NMR** (500 MHz, $CDCl_3$) δ 7.14 – 7.09 (m, 2H), 6.73 (d, *J* = 8.7 Hz, 1H), 3.81 (s, 3H), 2.20 (s, 3H). **^{13}C NMR** (126 MHz, $CDCl_3$) δ 156.50, 130.48, 128.62, 126.45, 125.06, 111.07, 55.66, 16.21. The spectra data are consistent with those reported in literature.[6]

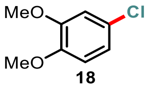

18

Compound **18** was isolated in 85% yield following the general conditions A. **^{1}H NMR** (500 MHz, $CDCl_3$) δ 6.90 – 6.82 (m, 2H), 6.76 (d, *J* = 8.5 Hz, 1H), 3.85 (s, 3H), 3.85 (s, 3H). **^{13}C NMR** (126 MHz, $CDCl_3$) δ 149.68, 147.95, 125.74, 120.39, 112.17, 112.05, 56.19, 56.15. The spectra data are consistent with those reported in literature.[6]

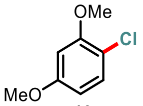

19

Compound **19** was isolated in 88% yield following the general conditions A. **^{1}H NMR** (500 MHz, $CDCl_3$) δ 7.24 (d, *J* = 8.7 Hz, 1H), 6.50 (s, 1H), 6.42 (d, *J* = 8.8 Hz, 1H), 3.87 (s, 3H), 3.79 (s, 3H). **^{13}C NMR** (126 MHz, $CDCl_3$) δ 159.62, 155.74, 130.25, 114.26, 105.28, 100.14, 56.17, 55.69. The spectra data are consistent with those reported in literature.[6]

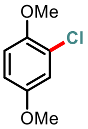

20

Compound **20** was isolated in 70% yield following the general conditions A. **^{1}H NMR** (500 MHz, $CDCl_3$) δ 6.95 (s, 1H), 6.86 (d, *J* = 8.9 Hz, 1H), 6.76 (d, *J* = 6.7 Hz, 1H), 3.85 (s, 3H), 3.76 (s, 3H). **^{13}C NMR** (126 MHz, $CDCl_3$) δ 153.95, 149.50, 123.08, 116.25, 113.33, 112.97, 56.88, 55.97. The spectra data are consistent with those reported in literature.[6]

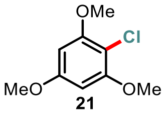

21

Compound **21** was isolated in 83% yield following the general conditions A. **^{1}H NMR** (500 MHz, $CDCl_3$) δ 6.18 (s, 2H), 3.88 (s, 6H), 3.81 (s, 3H). **^{13}C NMR** (126 MHz, $CDCl_3$) δ 159.56, 156.70, 102.87, 91.76, 56.45, 55.68. The spectra data are consistent with those reported in literature.[15]

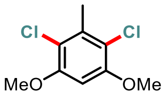

22

Compound **22** was isolated in 68% yield following the general conditions A. **^{1}H NMR** (500 MHz, $CDCl_3$) δ 6.45 (s, 1H), 3.90 (s, 6H), 2.48 (s, 3H). **^{13}C NMR** (126 MHz, $CDCl_3$) δ 154.28, 136.39, 115.37, 95.32, 56.62, 18.03. The spectra data are consistent

with those reported in literature.[16]

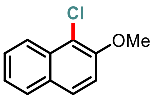

23

Compound **23** was isolated in 90% yield following the general conditions A. ^{1}**H NMR** (500 MHz, $CDCl_3$) δ 8.23 (d, J = 8.5 Hz, 1H), 7.82 – 7.76 (m, 2H), 7.58 (t, J = 7.1 Hz, 1H), 7.41 (t, J = 7.5 Hz, 1H), 7.30 (d, J = 9.0 Hz, 1H), 4.04 (s, 3H). ^{13}**C NMR** (126 MHz, $CDCl_3$) δ 152.69, 132.02, 129.66, 128.14, 128.10, 127.59, 124.45, 123.60, 117.03, 113.84, 57.11. The spectra data are consistent with those reported in literature.[6]

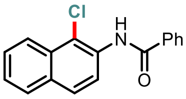

24

Compound **24** was isolated in 80% yield following the general conditions A. ^{1}**H NMR** (500 MHz, $CDCl_3$) δ 8.69 (d, J = 9.2 Hz, 2H), 8.19 (d, J = 8.4 Hz, 1H), 7.99 (d, J = 7.6 Hz, 2H), 7.84 (dd, J = 8.7, 5.1 Hz, 2H), 7.63 – 7.58 (m, 2H), 7.54 (t, J = 7.5 Hz, 2H), 7.49 (t, J = 7.5 Hz, 1H). ^{13}**C NMR** (126 MHz, $CDCl_3$) δ 165.61, 134.79, 133.05, 132.40, 131.42, 130.74, 129.11, 128.31, 127.79, 127.67, 127.34, 125.72, 123.94, 120.52, 118.69. **HRMS** Calcd for $C_{17}H_{12}ClNNaO^+$ [$M+Na^+$]: 304.0500; Found: 304.0493.

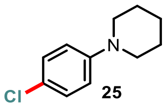

25

Compound **25** was isolated in 60% yield following the general conditions A. ^{1}**H NMR** (500 MHz, $CDCl_3$) δ 7.18 (d, J = 8.8 Hz, 2H), 6.84 (d, J = 8.5 Hz, 2H), 3.11 (t, J = 5.5 Hz, 4H), 1.72 – 1.67 (m, 4H), 1.59 – 1.56 (m, 2H). The spectra data are consistent with those reported in literature.[17]

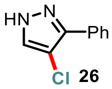

26

Compound **26** was isolated in 86% yield following the general conditions A. **^{1}H NMR** (500 MHz, $CDCl_3$) δ 7.77 (d, *J* = 6.6 Hz, 2H), 7.52 (s, 1H), 7.48 – 7.38 (m, 3H). **^{13}C NMR** (126 MHz, $CDCl_3$) δ 142.89, 134.48, 129.42, 128.92, 128.86, 127.39, 108.11. The spectra data are consistent with those reported in literature.[18]

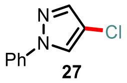

27

Compound **27** was isolated in 71% yield following the general conditions A. **^{1}H NMR** (500 MHz, $CDCl_3$) δ 7.91 (s, 1H), 7.67 – 7.60 (m, 3H), 7.46 (t, *J* = 8.0 Hz, 2H), 7.32 (t, *J* = 7.4 Hz, 1H). **^{13}C NMR** (126 MHz, $CDCl_3$) δ 139.85, 139.60, 129.67, 127.13, 124.96, 119.10, 112.51. **HRMS** Calcd for $C_9H_8ClN_2^+$ [M+H$^+$]: 179.0371; Found: 179.0372.

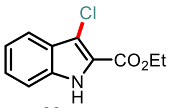

28

Compound **28** was isolated in 70% yield following the general conditions A. **^{1}H NMR** (500 MHz, $CDCl_3$) δ 9.01 (br, 1H), 7.72 (d, *J* = 8.2 Hz, 1H), 7.43 – 7.34 (m, 2H), 7.22 (t, *J* = 8.0 Hz, 1H), 4.47 (q, *J* = 7.1 Hz, 2H), 1.46 (t, *J* = 7.2 Hz, 3H). **^{13}C NMR** (126 MHz, $CDCl_3$) δ 161.16, 134.88, 126.71, 126.39, 122.56, 121.43, 120.38, 112.61, 112.19, 61.56, 14.50. The spectra data are consistent with those reported in literature.[6]

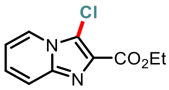

29

Compound **29** was isolated in 90% yield following the general conditions A. **^{1}H NMR** (500 MHz, $CDCl_3$) δ 8.18 (d, *J* = 7.0 Hz, 1H), 7.66 (d, *J* = 9.2 Hz, 1H), 7.34 – 7.27 (m, 1H), 6.98 (t, *J* = 6.9 Hz, 1H), 4.47 (q, *J* = 7.2 Hz, 2H), 1.44 (t, *J* = 7.1 Hz, 3H). **^{13}C NMR** (126 MHz, $CDCl_3$) δ 162.38, 145.22, 133.89, 126.65, 124.52, 119.25, 114.57, 100.29, 61.47, 14.45. **HRMS** Calcd for $C_{10}H_{10}ClN_2O_2^+$ [M+H$^+$]: 225.0425; Found: 225.0431.

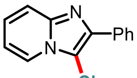

30

Compound **30** was isolated in 90% yield following the general conditions A. ^{1}**H NMR** (500 MHz, $CDCl_3$) δ 8.14 (d, *J* = 7.4 Hz, 2H), 8.09 (d, *J* = 6.8 Hz, 1H), 7.63 (d, *J* = 9.1 Hz, 1H), 7.48 (t, *J* = 7.7 Hz, 2H), 7.38 (t, *J* = 7.4 Hz, 1H), 7.23 (t, *J* = 7.3 Hz, 1H), 6.91 (t, *J* = 6.7 Hz, 1H). ^{13}**C NMR** (126 MHz, $CDCl_3$) δ 143.78, 139.83, 132.57, 128.66, 128.38, 127.59, 125.02, 122.79, 117.73, 113.05, 105.81. The spectra data are consistent with those reported in literature.[19]

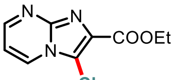

31

Compound **31** was isolated in 63% yield following the general conditions A. ^{1}**H NMR** (500 MHz, $CDCl_3$) δ 8.72 (s, 1H), 8.50 (d, *J* = 17.0 Hz, 1H), 7.09 (s, 1H), 4.50 (q, *J* = 7.1 Hz, 2H), 1.46 (t, *J* = 7.2 Hz, 3H). ^{13}**C NMR** (126 MHz, $CDCl_3$) δ 152.74, 152.58, 132.47, 131.24, 110.80, 110.59, 61.83, 14.47. The spectra data are consistent with those reported in literature.[6]

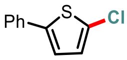

32

Compound **32** was isolated in 86% yield following the general conditions A. ^{1}**H NMR** (500 MHz, $CDCl_3$) δ 7.52 (d, *J* = 7.1 Hz, 2H), 7.38 (t, *J* = 7.6 Hz, 2H), 7.30 (t, *J* = 7.4 Hz, 1H), 7.08 (d, *J* = 3.8 Hz, 1H), 6.90 (d, *J* = 3.9 Hz, 1H). ^{13}**C NMR** (126 MHz, $CDCl_3$) δ 143.08, 133.83, 129.27, 129.15, 127.97, 127.24, 125.69, 122.38. The spectra data are consistent with those reported in literature.[20]

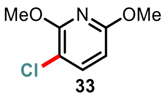

33

Compound **33** was isolated in 34% yield following the general conditions A (the recovery of starting material is 50%). ^{1}**H NMR** (500 MHz, $CDCl_3$) δ 7.50 (d, *J* = 8.4

Hz, 1H), 6.27 (d, J = 8.3 Hz, 1H), 4.01 (s, 3H), 3.90 (s, 3H). **^{13}C NMR** (126 MHz, CDCl3) δ 161.40, 157.72, 140.75, 107.99, 102.10, 77.41, 77.16, 76.91, 54.20, 53.94. The spectra data are consistent with those reported in literature.[6]

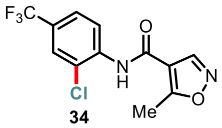

34

Compound **34** was isolated in 63% yield following the general conditions A. **^{1}H NMR** (500 MHz, $CDCl_3$) δ 8.63 (d, J = 8.7 Hz, 1H), 8.51 (s, 1H), 8.04 (br, 1H), 7.69 (s, 1H), 7.58 (d, J = 8.7 Hz, 1H), 2.81 (d, J = 1.4 Hz, 3H). **^{13}C NMR** (126 MHz, $CDCl_3$) δ 173.58, 159.05, 147.91, 137.26, 126.46, 126.43, 125.36, 125.33, 122.74, 121.27, 112.18, 12.92. **^{13}C NMR** (126 MHz, $CDCl_3$) δ 173.58, 159.05, 147.91, 137.26, 127.25, 126.98, 126.47, 126.43, 126.40, 125.39, 125.36, 125.33, 125.30, 124.41, 122.74, 122.24, 121.27, 112.18, 12.92. **^{19}F NMR** (471 MHz, $CDCl_3$) δ -62.35 (s, 3F). The spectra data are consistent with those reported in literature.[21]

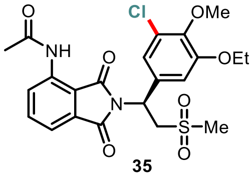

35

Compound **35** was isolated in 67% yield following the general conditions A. **^{1}H NMR** (500 MHz, $CDCl_3$) δ 9.45 (s, 1H), 8.76 (d, J = 8.8 Hz, 1H), 7.65 (t, J = 7.9 Hz, 1H), 7.50 (d, J = 7.3 Hz, 1H), 7.28 (s, 1H), 6.86 (s, 1H), 6.34 (d, J = 11.8 Hz, 1H), 4.50 (t, J = 13.2 Hz, 1H), 4.12 – 4.06 (m, 3H), 3.84 (s, 3H), 3.48 (dd, J = 14.8, 2.8 Hz, 1H), 3.01 (s, 3H), 2.25 (s, 3H), 1.45 (t, J = 7.0 Hz, 3H). **^{13}C NMR** (126 MHz, $CDCl_3$) δ 169.69, 169.30, 168.00, 150.29, 147.68, 137.77, 136.26, 131.18, 126.07, 125.10, 124.15, 118.36, 115.17, 113.48, 112.77, 65.00, 56.28, 53.71, 45.86, 41.03, 25.03, 14.68. The spectra data are consistent with those reported in literature.[6]

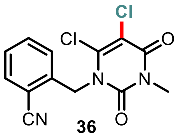

36

Compound **36** was isolated in 71% yield following the general conditions A. **^{1}H NMR** (500 MHz, $CDCl_3$) δ 7.72 (d, *J* = 7.7 Hz, 1H), 7.61 (t, *J* = 7.8 Hz, 1H), 7.44 (t, *J* = 7.7 Hz, 1H), 7.22 (d, *J* = 7.9 Hz, 1H), 5.57 (s, 2H), 3.46 (s, 3H). **^{13}C NMR** (126 MHz, $CDCl_3$) δ 157.58, 150.24, 142.67, 138.79, 133.66, 133.53, 128.74, 126.65, 116.84, 111.28, 109.67, 49.32, 29.95. **HRMS** Calcd for $C_{13}H_9Cl_2N_3NaO_2^+$ [M+H$^+$]: 331.9964; Found: 331.9963.

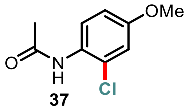

37

Compound **37** was isolated in 80% yield following the general conditions A. **^{1}H NMR** (500 MHz, $CDCl_3$) δ 8.14 (d, *J* = 9.1 Hz, 1H), 7.40 (br, 1H), 6.91 (s, 1H), 6.81 (d, *J* = 9.2 Hz, 1H), 3.78 (s, 3H), 2.21 (s, 3H). **^{13}C NMR** (126 MHz, $CDCl_3$) δ 168.20, 156.43, 128.05, 124.18, 123.45, 114.62, 113.36, 55.82, 24.72. The spectra data are consistent with those reported in literature.[22]

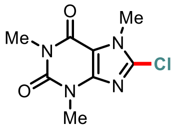

38

Compound **38** was isolated in 90% yield following the general conditions A. **^{1}H NMR** (500 MHz, $CDCl_3$) δ 3.96 (s, 3H), 3.55 (s, 3H), 3.40 (s, 3H). **^{13}C NMR** (126 MHz, $CDCl_3$) δ 154.66, 151.38, 147.17, 139.08, 108.34, 32.79, 29.92, 28.10. The spectra data are consistent with those reported in literature.[23]

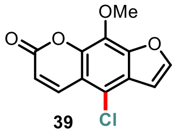

39

Compound **39** was isolated in 86% yield following the general conditions A. **^{1}H NMR**

(500 MHz, $CDCl_3$) δ 8.16 (d, *J* = 9.8 Hz, 1H), 7.71 (d, *J* = 2.3 Hz, 1H), 6.93 (d, *J* = 2.3 Hz, 1H), 6.46 (d, *J* = 9.9 Hz, 1H), 4.28 (s, 3H). **^{13}C NMR** (126 MHz, $CDCl_3$) δ 159.77, 147.19, 147.05, 143.71, 140.31, 132.04, 125.75, 116.29, 115.63, 114.34, 105.87, 61.58. The spectra data are consistent with those reported in literature.[24]

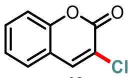

40

Compound **40** was isolated in 69% yield following the general conditions A. **^{1}H NMR** (500 MHz, $CDCl_3$) δ 7.88 (s, 1H), 7.55 (t, *J* = 7.1 Hz, 1H), 7.47 (d, *J* = 7.8 Hz, 1H), 7.35 (d, *J* = 8.3 Hz, 1H), 7.32 (t, *J* = 7.5 Hz, 1H). **^{13}C NMR** (126 MHz, $CDCl_3$) δ 157.42, 152.85, 140.18, 132.03, 127.40, 125.19, 122.54, 118.97, 116.94. The spectra data are consistent with those reported in literature.[8]

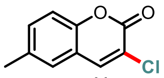

41

Compound **41** was isolated in 64% yield following the general conditions A. **^{1}H NMR** (500 MHz, $CDCl_3$) δ 7.80 (s, 1H), 7.34 (d, *J* = 8.6 Hz, 1H), 7.26 – 7.21 (m, 2H), 2.41 (s, 3H). **^{13}C NMR** (126 MHz, $CDCl_3$) δ 157.61, 150.98, 140.16, 135.03, 133.06, 127.13, 122.36, 118.70, 116.61, 20.88. The spectra data are consistent with those reported in literature.[8]

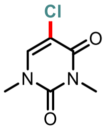

42

Compound **42** was isolated in 86% yield following the general conditions A. **^{1}H NMR** (500 MHz, $CDCl_3$) δ 7.43 (s, 1H), 3.41 (s, 3H), 3.37 (s, 3H). **^{13}C NMR** (126 MHz, $CDCl_3$) δ 159.61, 150.94, 140.05, 107.97, 37.34, 29.05. The spectra data are consistent with those reported in literature.[23]

6. General procedures and substrate scope for C-H bromination of quinones and

(hetero)arenes

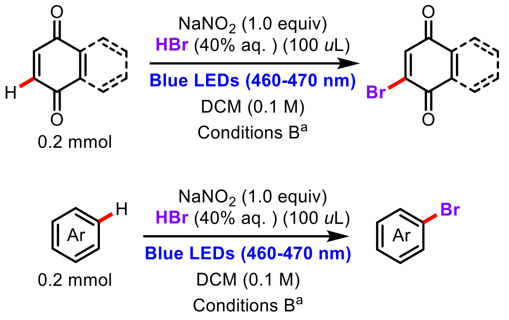

Scheme S6. C-H bromination reactions of quinones and hetero(arenes)

General conditions B: Substrate (0.2 mmol, 1.0 equiv), $NaNO_2$ (0.2 mmol, 1.0 equiv) and 40% aqueous HBr (100 *u*L) were dispersed in CH_2Cl_2 (2.0 mL) in a 4 mL glass vial at room temperature. The reaction vial was sealed with a PTEF cap and the reaction mixture vigorously stirred at room temperature (24 °C) under the 24 W blue LEDs (460-470 nm) irradiation for 10 hours (It is worth noting that 24 W blue LEDs was positioned 5 cm aside from the reaction vials.). Then, the reaction mixture was extracted with CH_2Cl_2 (3 × 2 mL). The combined organic layer was dried over anhydrous Na_2SO_4, filtered and concentrated. The residue was purified by chromatography on silica gel to afford the desired bromination products.

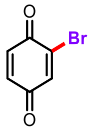

43

Compound **43** was isolated in 76% yield following the general conditions B. ^{1}**H NMR** (500 MHz, $CDCl_3$) δ 7.30 (s, 1H), 6.96 (d, *J* = 10.1 Hz, 1H), 6.83 (d, *J* = 10.1 Hz, 1H). The spectra data are consistent with those reported in literature.[25]

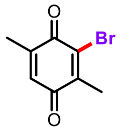

44

Compound **44** was isolated in 77% yield following the general conditions B. ^{1}**H NMR** (500 MHz, $CDCl_3$) δ 6.63 (q, *J* = 1.6 Hz, 1H), 2.19 (s, 3H), 2.10 (d, *J* = 1.6 Hz, 3H).

^{13}**C NMR** (126 MHz, $CDCl_3$) δ 184.32, 179.96, 146.15, 145.67, 136.06, 133.25, 77.41, 77.16, 76.91, 16.95, 16.65. The spectra data are consistent with those reported in literature.[8]

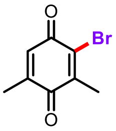

45

Compound **45** was isolated in 70% yield following the general conditions B. ^{1}**H NMR** (500 MHz, $CDCl_3$) δ 6.76 (q, J = 1.7 Hz, 1H), 2.23 (s, 3H), 2.08 (d, J = 1.7 Hz, 3H). ^{13}**C NMR** (126 MHz, $CDCl_3$) δ 184.62, 179.36, 146.18, 146.06, 136.05, 132.65, 17.23, 16.13. The spectra data are consistent with those reported in literature.[8]

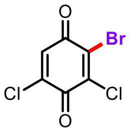

46

Compound **46** was isolated in 60% yield following the general conditions B. ^{1}**H NMR** (500 MHz, $CDCl_3$) δ 7.23 (s, 1H). ^{13}**C NMR** (126 MHz, $CDCl_3$) δ 175.79, 170.67, 144.03, 143.91, 136.51, 133.16. **HRMS** Calcd for $C_{10}H_{10}ClN_2O_2^+$ [M+K$^+$]: 292.8169; Found: 292.8198.

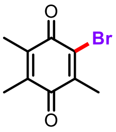

47

Compound **47** was isolated in 88% yield following the general conditions B. ^{1}**H NMR** (500 MHz, $CDCl_3$) δ 2.20 (s, 3H), 2.07 (s, 3H), 2.03 (s, 3H). ^{13}**C NMR** (126 MHz, $CDCl_3$) δ 184.40, 179.55, 145.74, 141.02, 140.76, 135.58, 17.20, 13.29, 12.76. The spectra data are consistent with those reported in literature.[26]

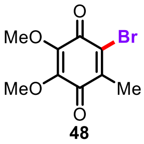

48

Compound **48** was isolated in 62% yield following the general conditions B. ^{1}**H NMR** (500 MHz, $CDCl_3$) δ 4.02 (s, 3H), 3.99 (s, 3H), 2.19 (s, 3H). ^{13}**C NMR** (126 MHz, $CDCl_3$) δ 181.10, 176.76, 145.29, 144.21, 143.89, 133.70, 61.66, 61.41, 16.82. The spectra data are consistent with those reported in literature.[8]

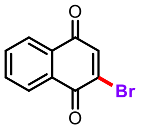

49

Compound **49** was isolated in 85% yield following the general conditions B. ^{1}**H NMR** (500 MHz, $CDCl_3$) δ 8.15 (d, *J* = 8.8 Hz, 1H), 8.06 (d, *J* = 6.7 Hz, 1H), 7.76 (t, *J* = 5.8 Hz, 2H), 7.50 (s, 1H). ^{13}**C NMR** (126 MHz, $CDCl_3$) δ 182.47, 177.93, 140.44, 140.22, 134.53, 134.22, 131.79, 131.02, 127.89, 126.96. The spectra data are consistent with those reported in literature.[8]

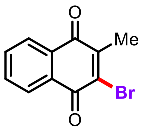

50

Compound **50** was isolated in 92% yield following the general conditions B. ^{1}**H NMR** (500 MHz, $CDCl_3$) δ 8.12 (d, *J* = 6.8 Hz, 1H), 8.08 (d, *J* = 6.9 Hz, 1H), 7.77 – 7.67 (m, 2H), 2.37 (s, 3H). ^{13}**C NMR** (126 MHz, $CDCl_3$) δ 181.97, 177.54, 148.55, 139.11, 134.19, 133.97, 131.58, 131.22, 127.56, 127.16, 17.94. The spectra data are consistent with those reported in literature.[12]

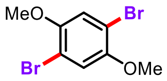

51

Compound **51** was isolated in 92% yield following the general conditions B. ^{1}**H NMR** (500 MHz, $CDCl_3$) δ 7.10 (s, 2H), 3.85 (s, 6H). ^{13}**C NMR** (126 MHz, $CDCl_3$) δ 150.68, 117.28, 110.64, 57.17. The spectra data are consistent with those reported in literature.[27]

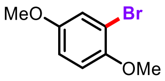

51'

By precisely reducing the aqueous HBr loading to 50 uL (1.7 equiv), 1,4-dimethoxybenzene provided the monobrominated product **51’** in 90 % isolated yield. **^{1}H NMR** (400 MHz, $CDCI_3$) δ 7.12 (dd, J = 2.5, 0.7 Hz, 1H), 6.84 – 6.81 (m, 2H), 3.84 (d, J = 1.3 Hz, 4H), 3.76 (s, 4H). **^{13}C NMR** (101 MHz, $CDCI_3$) δ 154.14, 150.41, 119.11, 117.25, 113.79, 113.03, 112.07, 56.97, 56.02. The spectra data are consistent with those reported in literature.[27]

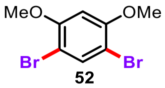

52

Compound **52** was isolated in 93% yield following the general conditions B. **^{1}H NMR** (500 MHz, $CDCl_3$) δ 7.62 (s, 1H), 6.45 (s, 1H), 3.87 (s, 6H). **^{13}C NMR** (126 MHz, $CDCl_3$) δ 156.20, 135.87, 102.42, 97.43, 56.57. The spectra data are consistent with those reported in literature.[28]

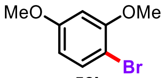

52'

By precisely reducing the aqueous HBr loading to 50 uL (1.7 equiv), 1,3-dimethoxybenzene provided the monobrominated product **52’** in 90 % isolated yield. **^{1}H NMR** (400 MHz, $CDCI_3$) δ 7.39 (d, J = 8.7 Hz, 1H), 6.48 (d, J = 2.8 Hz, 1H), 6.39 (dd, J = 8.7, 2.7 Hz, 1H), 3.86 (s, 3H), 3.78 (s, 3H). **^{13}C NMR** (101 MHz, $CDCI_3$) δ 160.31, 156.60, 133.21, 105.98, 102.48, 100.03, 56.20, 55.63. The spectra data are consistent with those reported in literature.[28]

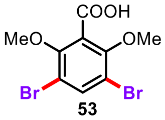

53

Compound **53** was isolated in 90% yield following the general conditions B. **^{1}H NMR** (500 MHz, $CDCl_3$) δ 7.84 (s, 1H), 3.95 (s, 6H). **^{13}C NMR** (126 MHz, $CDCl_3$) δ 169.02,

154.51, 138.33, 125.36, 112.97, 62.69. **HRMS** Calcd for $C_9H_8Br_2NaO_4^+$ [M+Na$^+$]: 360.8682; Found: 360.8682.

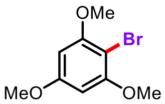

54

Compound **54** was isolated in 83% yield following the general conditions B. **^{1}H NMR** (500 MHz, $CDCl_3$) δ 6.16 (s, 2H), 3.86 (s, 6H), 3.81 (s, 3H). **^{13}C NMR** (126 MHz, $CDCl_3$) δ 160.58, 157.58, 92.11, 91.76, 56.46, 55.63. The spectra data are consistent with those reported in literature.[29]

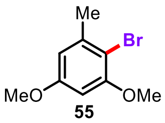

55

Compound **55** was isolated in 76% yield following the general conditions B. **^{1}H NMR** (500 MHz, $CDCl_3$) δ 6.42 (s, 1H), 6.35 (s, 1H), 3.86 (s, 3H), 3.79 (s, 3H), 2.39 (s,3H). **^{13}C NMR** (126 MHz, $CDCl_3$) δ 159.45, 156.77, 139.95, 107.36, 105.25, 97.38, 56.41, 55.60, 23.70. The spectra data are consistent with those reported in literature.[30]

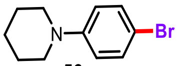

56

Compound **56** was isolated in 78% yield following the general conditions B. **^{1}H NMR** (500 MHz, $CDCl_3$) δ 7.32 (d, J = 8.6 Hz, 2H), 6.80 (d, J = 8.5 Hz, 2H), 3.12 (t, J = 5.5 Hz, 4H), 1.74 – 1.65 (m, 4H), 1.61 – 1.54 (m, 2H). **^{13}C NMR** (126 MHz, $CDCl_3$) δ 151.28, 131.85, 118.13, 111.20, 50.56, 25.78, 24.29. The spectra data are consistent with those reported in literature.[31a]

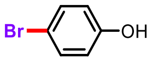

56'

Compound **56'** was isolated in 78% yield following the general conditions B. **^{1}H NMR** (500 MHz, $CDCl_3$) δ 7.37 – 7.30 (m, 2H), 6.75 – 6.69 (m, 2H), 5.05 (s, 1H). **^{13}C NMR** (126 MHz, $CDCl_3$) δ 154.89, 132.61, 117.36, 112.95. The spectra data are consistent with those reported in literature.[31b]

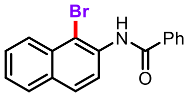

57

Compound **57** was isolated in 84% yield following the general conditions B. **^{1}H NMR** (500 MHz, $CDCl_3$) δ 8.74 (s, 1H), 8.67 (d, *J* = 8.9 Hz, 1H), 8.17 (d, *J* = 8.5 Hz, 1H), 8.00 (d, *J* = 7.4 Hz, 2H), 7.85 (d, *J* = 9.0 Hz, 1H), 7.81 (d, *J* = 8.1 Hz, 1H), 7.62 – 7.51 (m, 4H), 7.47 (t, *J* = 7.5 Hz, 1H). **^{13}C NMR** (126 MHz, $CDCl_3$) δ 165.52, 134.68, 134.52, 132.36, 132.04, 131.70, 129.06, 128.54, 128.30, 127.89, 127.31, 126.64, 125.69, 120.82, 112.18. The spectra data are consistent with those reported in literature.[32]

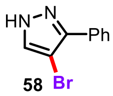

58

Compound **58** was isolated in 93% yield following the general conditions B. **^{1}H NMR** (500 MHz, $CDCl_3$) δ 7.76 (d, *J* = 6.7 Hz, 2H), 7.53 (s, 1H), 7.47 – 7.39 (m, 3H). The spectra data are consistent with those reported in literature.[33]

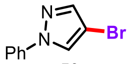

59

Compound **59** was isolated in 93% yield following the general conditions B. **^{1}H NMR** (500 MHz, $CDCl_3$) δ 7.93 (s, 1H), 7.68 (s, 1H), 7.64 (d, *J* = 8.0 Hz, 2H), 7.45 (t, *J* = 7.8 Hz, 2H), 7.31 (t, *J* = 7.4 Hz, 1H). **^{13}C NMR** (126 MHz, $CDCl_3$) δ 141.63, 139.76, 129.66, 127.16, 127.13, 119.14, 95.75. The spectra data are consistent with those reported in literature.[34]

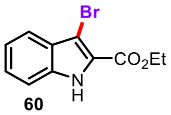

60

Compound **60** was isolated in 86% yield following the general conditions B. **^{1}H NMR** (500 MHz, $CDCl_3$) δ 9.32 (br, 1H), 7.70 (d, *J* = 8.1 Hz, 1H), 7.46 – 7.37 (m, 2H), 7.30 – 7.22 (m, 1H), 4.50 (q, *J* = 7.1 Hz, 2H), 1.49 (t, *J* = 7.2 Hz, 3H). **^{13}C NMR** (126 MHz, $CDCl_3$) δ 161.31, 135.54, 128.11, 126.67, 124.24, 121.56, 121.42, 112.17, 98.39, 61.64, 14.46. The spectra data are consistent with those reported in literature.[34]

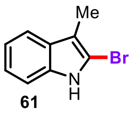

61

Compound **61** was isolated in 70% yield following the general conditions B. **^{1}H NMR** (500 MHz, $CDCl_3$) δ 7.97 – 7.83 (br, 1H), 7.49 (d, *J* = 7.8 Hz, 1H), 7.29 – 7.26 (m, 1H), 7.17 (t, *J* = 7.5 Hz, 1H), 7.12 (t, *J* = 7.5 Hz, 1H), 2.27 (s, 3H). **^{13}C NMR** (126 MHz, $CDCl_3$) δ 136.13, 128.50, 122.33, 119.98, 118.37, 111.57, 110.41, 108.07, 9.53. The spectra data are consistent with those reported in literature.[35]

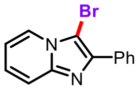

62

Compound **62** was isolated in 90% yield following the general conditions B. **^{1}H NMR** (500 MHz, $CDCl_3$) δ 8.17 (d, *J* = 6.9 Hz, 1H), 8.13 (d, *J* = 8.0 Hz, 2H), 7.65 (d, *J* = 9.1 Hz, 1H), 7.49 (t, *J* = 7.4 Hz, 2H), 7.39 (t, *J* = 7.2 Hz, 1H), 7.26 (t, *J* = 7.9 Hz, 1H), 6.93 (t, *J* = 6.8 Hz, 1H). **^{13}C NMR** (126 MHz, $CDCl_3$) δ 145.47, 142.62, 132.76, 128.62, 128.50, 128.05, 125.44, 124.11, 117.67, 113.32, 91.94. The spectra data are consistent with those reported in literature.[36]

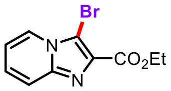

63

Compound **63** was isolated in 90% yield following the general conditions B. **^{1}H NMR** (500 MHz, $CDCl_3$) δ 8.18 (d, *J* = 6.9 Hz, 1H), 7.67 (d, *J* = 9.2 Hz, 1H), 7.31 (t, *J* = 7.0

Hz,1H), 6.99 (t, J = 6.9 Hz, 1H), 4.47 (q, J = 7.1 Hz, 2H), 1.44 (t, J = 7.1 Hz, 3H). **^{13}C NMR** (126 MHz, $CDCl_3$) δ 162.41, 145.22, 133.84, 126.70, 124.54, 119.26, 114.60, 100.35, 61.51, 14.47. The spectra data are consistent with those reported in literature.[37]

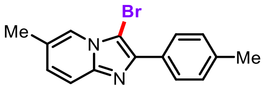

64

Compound **64** was isolated in 83% yield following the general conditions B. **^{1}H NMR** (500 MHz, $CDCl_3$) δ 8.01 (d, J = 7.8 Hz, 2H), 7.93 (s, 1H), 7.52 (d, J = 9.1 Hz, 1H), 7.28 (d, J = 7.8 Hz, 2H), 7.08 (d, J = 9.1 Hz, 1H), 2.40 (s, 3H), 2.38 (s, 3H). **^{13}C NMR** (126 MHz, $CDCl_3$) δ 144.57, 142.59, 138.11, 130.27, 129.27, 128.23, 127.77, 122.86, 121.72, 116.94, 91.05, 21.46, 18.48. **HRMS** Calcd for $C_{15}H_{14}BrN_2^+$ [M+H$^+$]: 301.0331; Found: 301.0339.

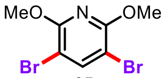

65

Compound **65** was isolated in 62% yield following the general conditions B. **^{1}H NMR** (500 MHz, $CDCl_3$) δ 7.86 (s, 1H), 3.99 (s, 6H). **^{13}C NMR** (126 MHz, $CDCl_3$) δ 157.83, 145.74, 95.74, 54.81. The spectra data are consistent with those reported in literature.[38]

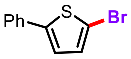

66

Compound **66** was isolated in 80% yield following the general conditions B. **^{1}H NMR** (500 MHz, $CDCl_3$) δ 7.52 (d, J = 7.7 Hz, 2H), 7.38 (t, J = 7.6 Hz, 2H), 7.30 (t, J = 7.4 Hz, 1H), 7.06 (d, J = 3.9 Hz, 1H), 7.03 (d, J = 3.7 Hz, 1H). **^{13}C NMR** (126 MHz, $CDCl_3$) δ 146.01, 133.78, 130.97, 129.15, 128.02, 125.75, 123.36, 111.52. The spectra data are consistent with those reported in literature.[39]

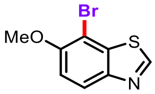

67

Compound **67** was isolated in 64% yield following the general conditions B. ^{1}**H NMR** (500 MHz, $CDCl_3$) δ 8.87 (s, 1H), 8.01 (d, *J* = 8.8 Hz, 1H), 7.14 (d, *J* = 8.8 Hz, 1H), 3.98 (s, 3H). ^{13}**C NMR** (126 MHz, $CDCl_3$) δ 154.34, 152.30, 147.23, 138.96, 122.92, 111.77, 102.45, 57.28. **HRMS** Calcd for $C_8H_7BrNOS^+$ [M+H$^+$]: 243.9426; Found: 243.9431.

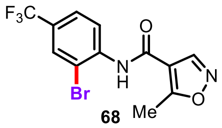

68

Compound **68** was isolated in 48% yield following the general conditions B. ^{1}**H NMR** (500 MHz, $CDCl_3$) δ 8.63 (d, *J* = 8.7 Hz, 1H), 8.52 (s, 1H), 8.06 (br, 1H), 7.86 (s, 1H), 7.63 (d, *J* = 8.7 Hz, 1H), 2.82 (s, 3H). ^{13}**C NMR** (126 MHz, $CDCl_3$) δ 173.67, 159.06, 147.90, 138.37, 129.62, 129.58, 126.03, 126.00, 121.30, 113.03, 112.17, 13.00. ^{19}**F NMR** (471 MHz, $CDCl_3$) δ -62.32 (s, 3F). The spectra data are consistent with those reported in literature.[21]

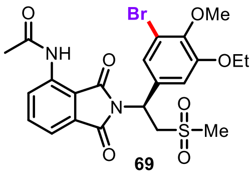

69

Compound **69** was isolated in 91% yield following the general conditions B. ^{1}**H NMR** (500 MHz, $CDCl_3$) δ 9.43 (s, 1H), 8.73 (d, *J* = 8.5 Hz, 1H), 7.63 (t, *J* = 7.9 Hz, 1H), 7.47 (d, *J* = 7.2 Hz, 1H), 7.32 (s, 1H), 6.99 (s, 1H), 6.29 (dd, *J* = 11.8, 3.0 Hz, 1H), 4.48 (t, *J* = 13.1 Hz, 1H), 4.09 – 4.02 (m, 3H), 3.81 (s, 3H), 3.46 (dd, *J* = 14.6, 3.0 Hz, 1H), 2.99 (s, 3H), 2.23 (s, 3H), 1.42 (t, *J* = 7.0 Hz, 3H). ^{13}**C NMR** (126 MHz, $CDCl_3$) δ 169.82, 169.35, 168.16, 150.30, 148.35, 137.78, 136.30, 131.20, 128.08, 125.14, 118.42, 115.79, 115.19, 113.60, 113.17, 64.91, 56.31, 53.76, 48.83, 41.07, 25.04, 14.66. **HRMS** Calcd for $C_{22}H_{23}BrN_2NaO_7S^+$ [M+Na$^+$]: 561.0302; Found: 561.0302.

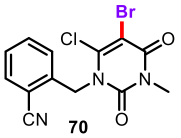

70

Compound **70** was isolated in 76% yield following the general conditions B. ^{1}**H NMR** (500 MHz, $CDCl_3$) δ 7.72 (d, *J* = 7.7 Hz, 1H), 7.61 (t, *J* = 7.7 Hz, 1H), 7.45 (t, *J* = 7.6 Hz, 1H), 7.22 (d, *J* = 7.9 Hz, 1H), 5.61 (s, 2H), 3.47 (s, 3H). ^{13}**C NMR** (126 MHz, $CDCl_3$) δ 157.84, 150.57, 144.47, 138.84, 133.68, 133.54, 128.74, 126.64, 116.86, 111.30, 99.63, 49.84, 30.24. The spectra data are consistent with those reported in literature.[21]

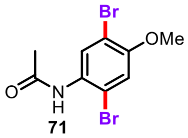

71

Compound **71** was isolated in 83% yield following the general conditions B. ^{1}**H NMR** (500 MHz, $CDCl_3$) δ 8.48 (s, 1H), 7.34 (br, 1H), 7.04 (s, 1H), 3.87 (s, 3H), 2.22 (s, 3H). ^{13}**C NMR** (126 MHz, $CDCl_3$) δ 168.14, 152.98, 129.96, 126.95, 115.30, 112.76, 111.34, 56.84, 24.74. **HRMS** Calcd for $C_9H_{10}Br_2NO_2^+$ [M+H$^+$]: 321.9073; Found: 321.9074.

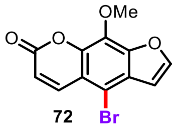

72

Compound **72** was isolated in 76% yield following the general conditions B. ^{1}**H NMR** (500 MHz, $CDCl_3$) δ 8.12 (d, *J* = 9.8 Hz, 1H), 7.73 (d, *J* = 2.2 Hz, 1H), 6.88 (d, *J* = 2.2 Hz, 1H), 6.45 (d, *J* = 9.8 Hz, 1H), 4.28 (s, 3H). ^{13}**C NMR** (126 MHz, $CDCl_3$) δ 159.89, 147.04, 146.83, 143.91, 142.76, 132.60, 128.13, 116.00, 115.83, 107.61, 105.70, 61.58. The spectra data are consistent with those reported in literature.[40]

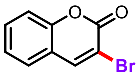

73

Compound **73** was isolated in 66% yield following the general conditions B. ^{1}**H NMR** (500 MHz, $CDCl_3$) δ 8.11 (s, 1H), 7.57 (t, *J* = 8.1 Hz, 1H), 7.47 (d, *J* = 7.7 Hz, 1H), 7.35 (d, *J* = 8.3 Hz, 1H), 7.32 (t, *J* = 7.5 Hz, 1H). ^{13}**C NMR** (126 MHz, $CDCl_3$) δ 157.21, 153.37, 144.52, 132.22, 127.27, 125.12, 119.50, 116.99, 112.06. The spectra data are consistent with those reported in literature.[8]

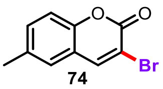

74

Compound **74** was isolated in 61% yield following the general conditions B. ^{1}**H NMR** (500 MHz, $CDCl_3$) δ 8.04 (s, 1H), 7.36 (d, *J* = 8.4 Hz, 1H), 7.26 – 7.21 (m, 2H), 2.41 (s, 3H). ^{13}**C NMR** (126 MHz, $CDCl_3$) δ 157.42, 151.52, 144.51, 134.96, 133.27, 126.99, 119.24, 116.67, 111.92, 20.88. The spectra data are consistent with those reported in literature.[40]

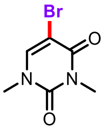

75

Compound **75** was isolated in 90% yield following the general conditions B. ^{1}**H NMR** (500 MHz, $CDCl_3$) δ 7.55 (s, 1H), 3.41 (s, 3H), 3.37 (s, 3H). ^{13}**C NMR** (126 MHz, $CDCl_3$) δ 159.60, 151.16, 142.59, 95.69, 37.33, 29.23. The spectra data are consistent with those reported in literature.[23]

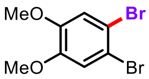

76

Compound **76** was isolated in 86% yield following the general conditions B. ^{1}**H NMR** (500 MHz, $CDCl_3$) δ 7.05 (s, 2H), 3.85 (s, 6H). ^{13}**C NMR** (126 MHz, $CDCl_3$) δ 149.02, 116.07, 114.90, 56.41. The spectra data are consistent with those reported in literature.[41]

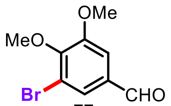

77

Compound **77** was isolated in 56% yield following the general conditions B. ^{1}**H NMR** (500 MHz, $CDCl_3$) δ 10.18 (s, 1H), 7.40 (s, 1H), 7.05 (s, 1H), 3.96 (s, 3H), 3.91 (s, 3H). ^{13}**C NMR** (126 MHz, $CDCl_3$) δ 190.93, 154.66, 149.05, 126.71, 120.54, 115.61, 110.61, 56.66, 56.32. The spectra data are consistent with those reported in literature.[42]

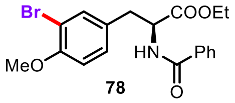

78

Compound **78** was isolated in 78% yield following the general conditions B. ^{1}**H NMR** (500 MHz, $CDCl_3$) δ 7.74 (d, *J* = 7.0 Hz, 2H), 7.50 (t, *J* = 7.4 Hz, 1H), 7.42 (t, *J* = 7.5 Hz, 2H), 7.33 (d, *J* = 2.2 Hz, 1H), 7.05 (dd, *J* = 8.4, 2.2 Hz, 1H), 6.80 (d, *J* = 8.4 Hz, 1H), 6.70 (d, *J* = 7.4 Hz, 1H), 5.00 (q, *J* = 5.5 Hz, 1H), 4.27 – 4.16 (m, 2H), 3.85 (s, 3H), 3.25 – 3.04 (m, 2H), 1.29 (t, *J* = 7.1 Hz, 3H). ^{13}**C NMR** (126 MHz, $CDCl_3$) δ 171.53, 166.98, 155.09, 134.33, 133.98, 131.87, 129.64, 129.45, 128.71, 127.09, 112.00, 111.59, 61.86, 56.27, 53.72, 36.76, 14.27. **HRMS** Calcd for $C_{19}H_{21}BrNO_4^+$ $[M+H^+]$: 406.0648; Found: 406.0648.

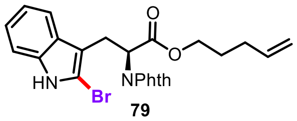

79

Compound **79** was isolated in 90% yield following the general conditions B. ^{1}**H NMR** (500 MHz, $CDCl_3$) δ 8.52 (d, *J* = 15.2 Hz, 1H), 7.72 – 7.67 (m, 2H), 7.63 – 7.57 (m, 2H), 7.44 (d, *J* = 7.9 Hz, 1H), 7.07 (d, *J* = 8.0 Hz, 1H), 7.00 (t, *J* = 7.5 Hz, 1H), 6.94 (t, *J* = 7.5 Hz, 1H), 5.79 – 5.69 (m, 1H), 5.26 – 5.19 (m, 1H), 5.01 – 4.91 (m, 2H), 4.29 – 4.17 (m, 2H), 3.72 – 3.62 (m, 2H), 2.06 (d, *J* = 8.6 Hz, 2H), 1.78 – 1.68 (m, 2H). ^{13}**C NMR** (126 MHz, $CDCl_3$) δ 169.01, 167.57, 137.29, 135.98, 134.06, 131.69, 127.55, 123.38, 122.26, 120.12, 117.65, 115.50, 110.66, 109.21, 65.48, 52.01, 29.97, 27.67, 24.68. **HRMS** Calcd for $C_{24}H_{21}BrKN_2O_4^+$ $[M+K^+]$: 519.0316; Found: 519.0316.

7. Visible-light-promoted $C(sp^2)$-H chlorination and bromination of electron-poor arenes and benzene

We have now carried out additional experiments with benzene and a set of representative electron-deficient arenes under our standard conditions. The results are summarized in Scheme S7.

Key observations:

1) Electron-poor arenes and hetero-arenes such as benzoic acid, nitrobenzene, pyridine, quinolone, and isoquinoline were completely unreactive under our standard chlorination conditions (Scheme S7, A).
2) When the electron-deficient heterocycle carries an electron-donating group, e.g., 2,6-dimethoxypyridine, the chlorination can proceed to give the corresponding selective chlorinated product in 34% yield (Scheme S7, A).
3) Benzene itself gave 46% mono-chlorination product under our standard chlorinated conditions (Scheme S7, A).
4) Electron-poor arenes and hetero-arenes such as benzoic acid, nitrobenzene, pyridine, quinolone, and isoquinoline were completely unreactive under our standard bromination conditions (Scheme S7, B).
5) When the electron-deficient heteroarenes or arenes carry electron-donating groups, e.g., 2,6-dimethoxypyridine, 2,6-dimethoxybenzoic acid or 3,4-dimethoxybenzaldehyde, the bromination can proceed to give the corresponding selective brominated products in moderate to good yields (Scheme S7, B).
6) Benzene itself gave 60% mono-bromination product under our standard chlorinated conditions (Scheme S7, B).

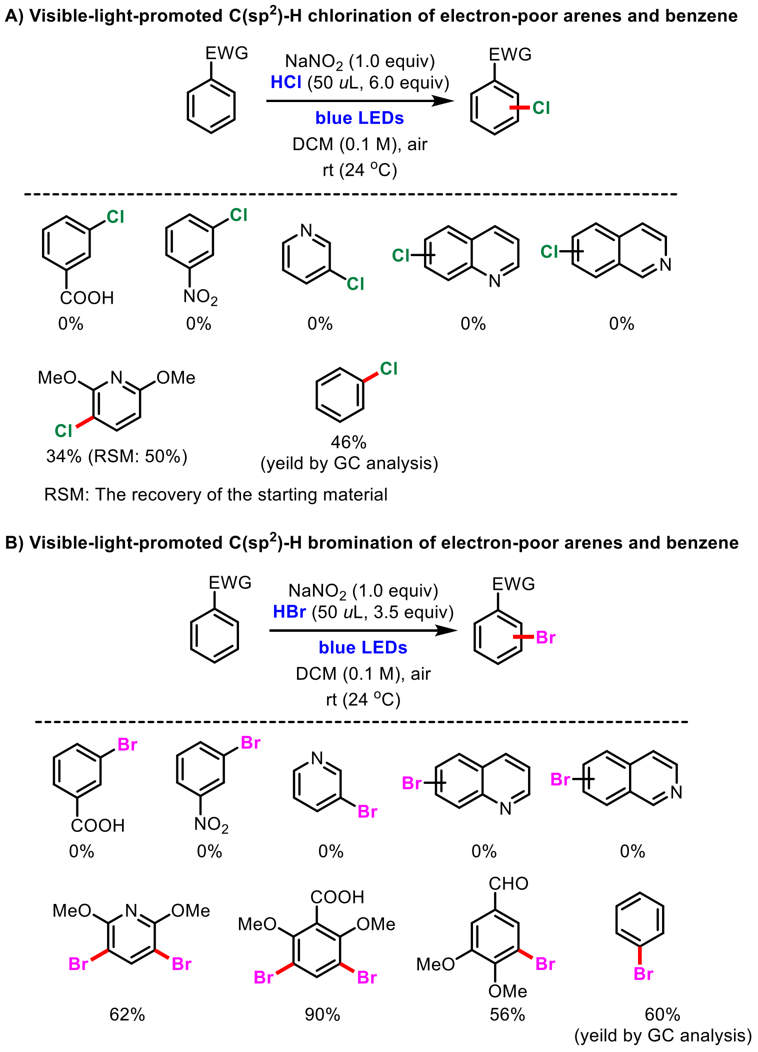

Scheme S7. Visible-light-promoted C(sp^2)-H chlorination and bromination of electron-poor arenes and benzene

8. Synthetic applications

8.1 Gram-scale continuous flow aromatic C(sp^2)-H chlorination of naproxen

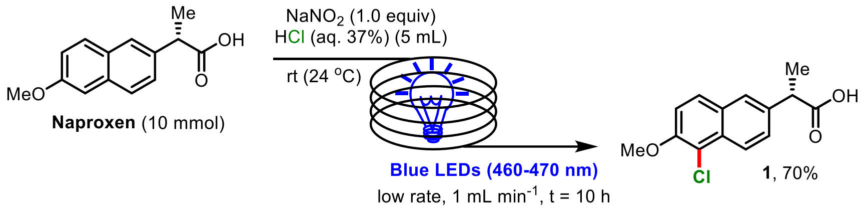

Scheme S8. Gram-scale continuous flow aromatic C(sp^2)-H chlorination of naproxen

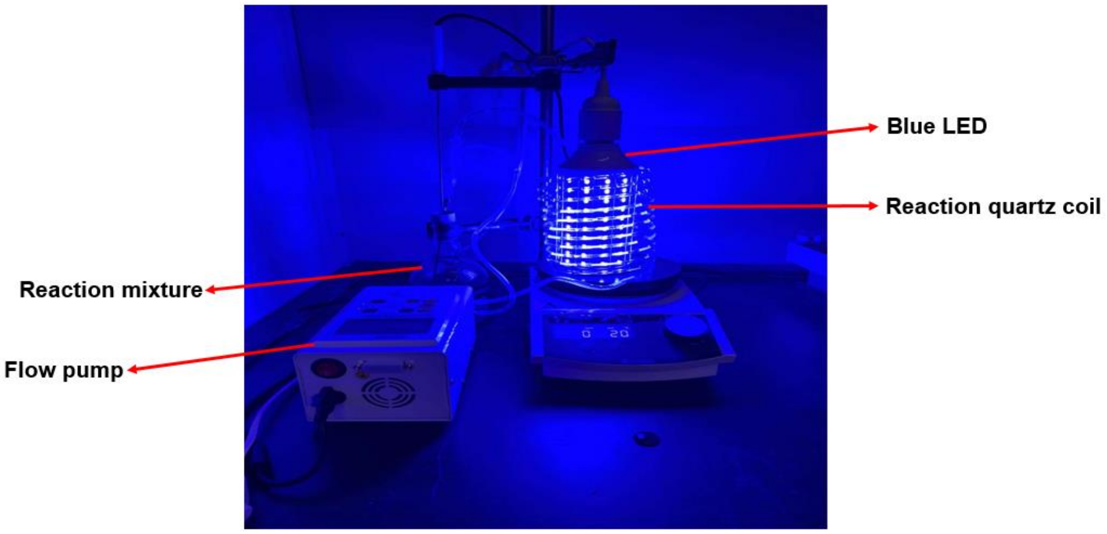

Figure S1. Gram-scale continuous flow aromatic C(sp^2)-H chlorination of naproxen

Naproxen (10.0 mmol, 1.0 equiv) and $NaNO_2$ (10.0 mmol, 1.0 equiv) were dispersed in CH_2Cl_2 (100.0 mL) in a 250 mL round bottom flask. Then HCl (5 mL, aq. 37% in water) was added at 0 °C. As shown in Figure S1, a flow pump was employed to pump the reaction mixture, and then attached to the flow apparatus was placed into a platform equipped with a 24 W blue LEDs (460-470 nm) and quartz reaction coil (The reaction coil is made of quartz, O.D. = 2 mm, I.D. = 1 mm). The flow apparatus itself was set up with flow rate = 1 mL min^{-1}. The flow reaction mixture irradiated with blue LEDs (460-470 nm) at room temperature (24 °C) for 10 hours until the reaction was complete (monitored by TLC). The reaction mixture was concentrated under reduced pressure, and then the resulting crude product was purified by column chromatography (eluent: *n*-hexane/AcOEt) to provide the chlorination product **1** in 70% isolated yield (1.85 g).

8.2 Gram-scale continuous flow aromatic C(sp^2)-H bromination of naproxen

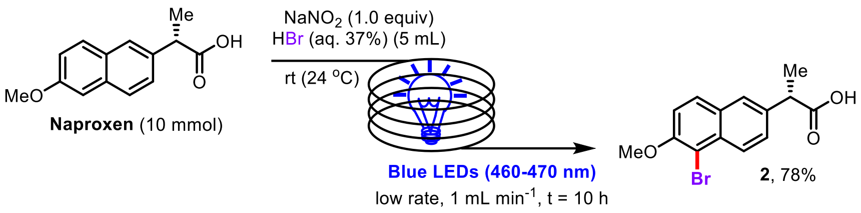

Scheme S9. Gram-scale continuous flow aromatic C(sp^2)-H bromination of naproxen

Naproxen (10.0 mmol, 1.0 equiv) and $NaNO_2$ (10.0 mmol, 1.0 equiv) were dispersed in CH_2Cl_2 (100.0 mL) in a 250 mL round bottom flask. Then HBr (5 mL, aq. 40% in water) was added at 0 °C. As shown in Figure S1, a flow pump was employed to pump the reaction mixture, and then attached to the flow apparatus was placed into a platform equipped with a 24 W blue LEDs (460-470 nm) and quartz reaction coil (The reaction coil is made of quartz, O.D. = 2 mm, I.D. = 1 mm). The flow apparatus itself was set up with flow rate = 1 mL min^{-1}. The flow reaction mixture irradiated with blue LEDs (460-470 nm) at room temperature (24 °C) for 10 hours until the reaction was complete (monitored by TLC). The reaction mixture was concentrated under reduced pressure, and then the resulting crude product was purified by column chromatography (eluent: *n*-hexane/AcOEt) to provide the bromination product **2** in 78% isolated yield (2.41 g).

9. Mechanistic studies

9.1 Mechanistic studies of aromatic C(sp^2)-H chlorination reaction

Radical scavenger experiment:

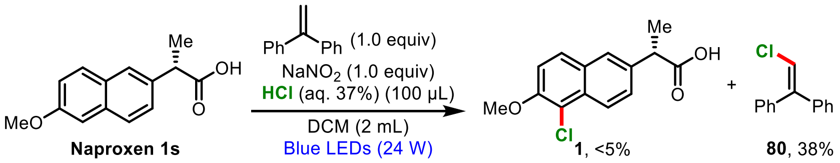

Scheme S10. Radical scavenger experiment

Radical scavenger 1,1-Diphenylethylene (0.2 mmol, 1.0 equiv), Naproxen (0.2 mmol, 1.0 equiv), $NaNO_2$ (0.2 mmol, 1.0 equiv) and HCl (100 *u*L, aq. 37% in water) were dispersed in CH_2Cl_2 (2.0 mL) in a 4 mL glass vial at room temperature. The reaction

vial was sealed with a PTEF cap and the reaction mixture vigorously stirred at 24 ℃ under the 24 W blue LEDs irradiation for 10 hours. Then, the reaction mixture was extracted with CH_2Cl_2 (3 × 2 mL). The combined organic layer was dried over anhydrous Na_2SO_4, filtered and concentrated. The residue was purified by chromatography on silica gel to afford the 2-chloro-1,1-diphenylethylene 16 mg (38% isolated yield).

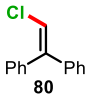

80

^{1}H NMR (500 MHz, $CDCl_3$) δ 7.43 – 7.38 (m, 2H), 7.38 – 7.30 (m, 6H), 7.24 – 7.19 (m, 2H), 6.60 (s, 1H). Compound **80** is known compounds and its spectra data is consistent with those reported in the literature.[43]

Hydrogen abstraction by chlorine atom:

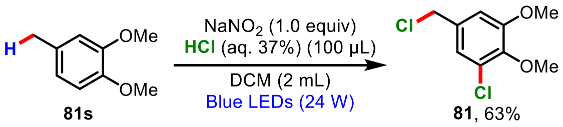

Scheme S11. Hydrogen abstraction by chlorine atom

Substrate 1,2-dimethoxy-4-methylbenzene (0.2 mmol, 1.0 equiv), $NaNO_2$ (0.2 mmol, 1.0 equiv) and HCl (100 uL, aq. 37% in water) were dispersed in CH_2Cl_2 (2.0 mL) in a 4 mL glass vial at room temperature. The reaction vial was sealed with a PTEF cap and the reaction mixture vigorously stirred at 24 ℃ under the 24 W blue LEDs irradiation for 10 hours. Then, the reaction mixture was extracted with CH_2Cl_2 (3 × 2 mL). The combined organic layer was dried over anhydrous Na_2SO_4, filtered and concentrated. The residue was purified by chromatography on silica gel to afford the 1-chloro-2-(chloromethyl)-4,5-dimethoxybenzene 27 mg (63%).

Notably, benzylic and aliphatic C-H bonds could be activated by the chlorine radical via hydrogen atom abstraction, leading to the corresponding chlorination product in 63% isolated yield.

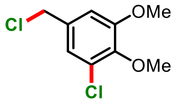

81

¹H NMR (500 MHz, $CDCl_3$) δ 6.93 (s, 1H), 6.88 (s, 1H), 4.67 (s, 2H), 3.89 (s, 3H), 3.88 (s, 3H). **¹³C NMR** (126 MHz, $CDCl_3$) δ 149.39, 149.29, 130.16, 121.29, 111.88, 111.16, 56.08, 56.04, 46.81. **GC-MS** Calcd for $C_9H_{10}Cl_2O_2$ [M]: 220.0058; Found: 220.0060.

Radical clock experiment:

Compounds **82s** and **83s** were examined for the chlorination reaction in order to prove that chlorine radical was produced under our chlorination standard conditions and carbon radical intermediate was involved in this chlorination process.

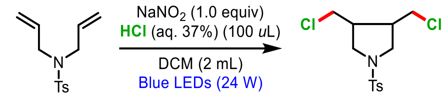

Scheme S12. Radical clock experiment of *N*-tosyldiallyamine

N-tosyldiallyamine (0.2 mmol, 1.0 equiv), $NaNO_2$ (0.2 mmol, 1.0 equiv) and HCl (100 *u*L, aq. 37% in water) were dispersed in CH_2Cl_2 (2.0 mL) in a 4 mL glass vial at room temperature. The reaction vial was sealed with a PTEF cap and the reaction mixture vigorously stirred at 24 ℃ under the 24 W blue LEDs (460-470 nm) irradiation for 10 hours. Then, the reaction mixture was extracted with CH_2Cl_2 (3 × 2 mL). The combined organic layer was dried over anhydrous Na_2SO_4, filtered and concentrated. The residue was purified by chromatography on silica gel to afford the cyclization-derived pyrrolidine 43 mg (67% isolated yield).

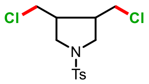

82

^{1}**H NMR** (500 MHz, $CDCl_3$) δ 7.71 (t, *J* = 8.8 Hz, 2H), 7.34 (d, *J* = 8.2 Hz, 2H), 3.54 – 3.37 (m, 5H), 3.31 – 3.24 (m, 2H), 3.12 (dd, *J* = 10.3, 6.1 Hz, 1H), 2.66 – 2.58 (m, 1H), 2.44 (s, 3H), 2.42 – 2.34 (m, 1H). Compound **82** is known and spectra data are consistent with those reported in the literature.[44]

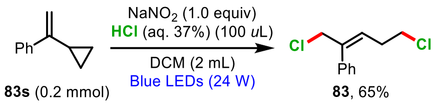

Scheme S13. Radical clock experiment of (1-cyclopropylvinyl)benzene

Cyclopropane-containing alkene (1-cyclopropylvinyl)benzene (0.2 mmol, 1.0 equiv), $NaNO_2$ (0.2 mmol, 1.0 equiv) and HCl (100 *u*L, aq. 37% in water) were dispersed in CH_2Cl_2 (2.0 mL) in a 4 mL glass vial at room temperature. The reaction vial was sealed with a PTEF cap and the reaction mixture vigorously stirred at 24 ℃ under the 24 W blue LEDs (460-470 nm) irradiation for 10 hours. Then, the reaction mixture was extracted with CH_2Cl_2 (3 × 2 mL). The combined organic layer was dried over anhydrous Na_2SO_4, filtered and concentrated. The residue was purified by chromatography on silica gel to afford the ring-opening product 28 mg (65%).

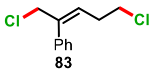

83

^{1}**H NMR** (500 MHz, $CDCl_3$) δ 7.46 (d, *J* = 7.6 Hz, 2H), 7.37 (t, *J* = 7.4 Hz, 2H), 7.34 – 7.28 (m, 1H), 6.01 (t, *J* = 7.4 Hz, 1H), 4.48 (s, 2H), 3.68 (t, *J* = 6.7 Hz, 2H), 2.81 (q, *J* = 6.9 Hz, 2H). Compound **83** is known and spectra data are consistent with those reported in the literature.[45]

Light/dark experiment of chlorination of compound of 42:

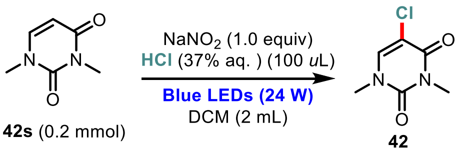

Scheme S14. Light/dark experiment of chlorination of **42**

Six vials were equipped with a stir bar and charged with **42** (0.2 mmol, 1.0 equiv), $NaNO_2$ (0.2 mmol, 1.0 equiv) and HCl (100 *u*L, aq. 37% in water) were dispersed in CH_2Cl_2 (2.0 mL) in a 4 mL glass vial at room temperature. The reaction vial was sealed with a PTEF cap and the reactions was alternatively irradiated with a Blue LEDs (24 W) and kept in the dark in 1 h intervals. After each interval, one vial was take out, the solvent was removed under reduced pressure, and the yield was determined by ^{1}H NMR based on a $Cl_2CHCHCl_2$ as an internal standard. These results indicated that continuous irradiation with light was essential for promoting the reaction.

Vial	Time (h)/condition						Yield (%)[a]
1	0-1/hv						14
2	0-1/hv	1-2/dark					20
3	0-1/hv	1-2/dark	2-3/hv				38
4	0-1/hv	1-2/dark	2-3/hv	3-4/dark			48
5	0-1/hv	1-2/dark	2-3/hv	3-4/dark	4-5/hv		62
6	0-1/hv	1-2/dark	2-3/hv	3-4/dark	4-5/hv	5-6/dark	69

a) ^{1}H-NMR yield, average of three experiments.

Table S2. Light/Dark experiment of chlorination of **42**.

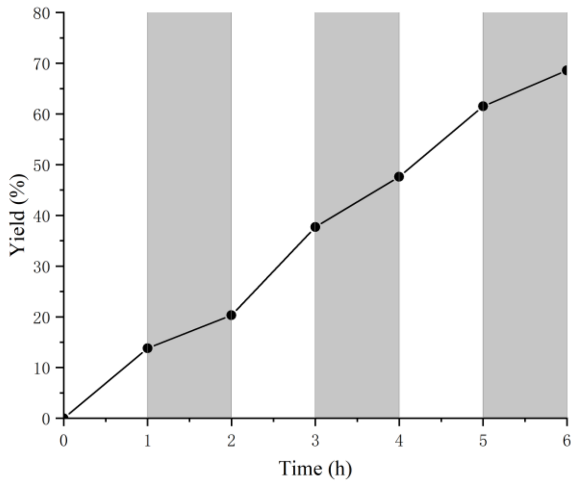

Figure S2. Light/dark experiment of chlorination of **42**

UV-vis experiment:

Ultraviolet–visible absorption experiments were performed using a Unicosh UV-4800 UV-visible spectrophotometer. Six samples were prepared, H_2O (2.0 mL); H_2O (2.0 mL) and mixtures of HCl (aq. 37% in water, 5 μL); H_2O (2.0 mL) and mixtures of $NaNO_2$ (0.1 mmol); H_2O (2.0 mL) and mixtures of $NaNO_2$ (0.1 mmol), HCl (aq. 37% in water, 5 μL); H_2O (2.0 mL) and mixtures of $NaNO_2$ (0.1 mmol), HCl (aq. 37% in water, 10 μL) and H_2O (2.0 mL) and mixtures of $NaNO_2$ (0.1 mmol), HCl (aq. 37% in water, 60 μL), a series of aliquots was transferred into the 2.0 mL quartz tube under an air atmosphere and measured directly.

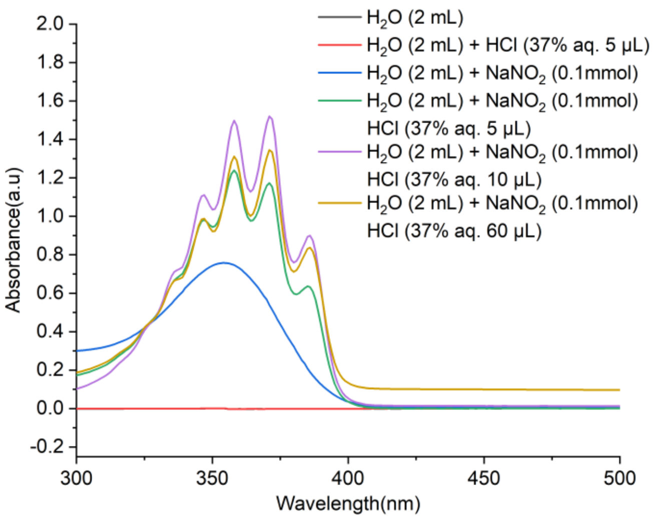

Figure S3. Ultraviolet–visible absorption spectrum

Proposed mechanism of C(sp^2)-H chlorination

Classic photochemistry mechanism (Figure S4, A):

We propose a plausible pathway for $NaNO_2$/HCl-mediated photochemical aromatic C(sp^2)-H chlorination. The radical process starts with the formation of nitrous acid (HNO_2) by a reaction between $NaNO_2$ and HCl. Then, nucleophilic substitution reaction between **Int I** (protonated HNO_2) and aqueous HCl *in situ* generates chlorinating reagent nitrosyl chloride (Cl–N=O). Under light irradiation, nitrosyl chloride undergoes homolytic cleavage to generate chlorine radical and nitric oxide radical. Subsequently, the chlorine radical selectively adds to the aromatic π-bond of the substrate, forming a carbon-centered radical intermediate (**Int II**). **Int II** undergoes a single-electron transfer (SET) with nitrosyl chloride to generate carbocation intermediate (**Int III**). A trace amount of H_2O present in aqueous HCl then acts as a base to facilitate proton elimination of **Int III**, furnishing the aromatic C(sp^2)–H chlorinated product; the concomitant restoration of aromaticity provides the principal thermodynamic driving force for this deprotonation event. Meanwhile, the one-electron-reduced nitrosyl chloride undergoes fragmentation to afford a chloride anion and nitric oxide. The latter is subsequently scavenged by air(O_2) and H_2O to regenerate nitrous acid (HNO_2), thereby re-entering the cycle.

The clear self-sustained component points toward a radical chain process being involved (Figure S4, B). Firstly, the chlorine radical generated through the photolysis of nitrosyl chloride react with the π-system of the arene to afford the corresponding carbon-centered radical intermediate (**Int II**). **Int II** undergoes a single-electron transfer (SET) with nitrosyl chloride to generate carbocation intermediate (**Int III**). A trace amount of H_2O present in aqueous HCl then acts as a base to facilitate proton elimination of **Int III**, furnishing the aromatic C(sp²)–H chlorinated product. Meanwhile, the one-electron-reduced nitrosyl chloride fragments to afford a nitric oxide anion (NO^-) and a chlorine radical (Cl•); the regenerated Cl• then propagates the radical chain. Thermodynamically, the cleavage of the one-electron-reduced nitrosyl chloride to generate nitric oxide anion (NO^-) and a chlorine radical (Cl•) is highly disfavoured; nevertheless, rapid downstream consumption of the nascent Cl• would shift the equilibrium toward chloride formation, rendering the fragmentation kinetically feasible.

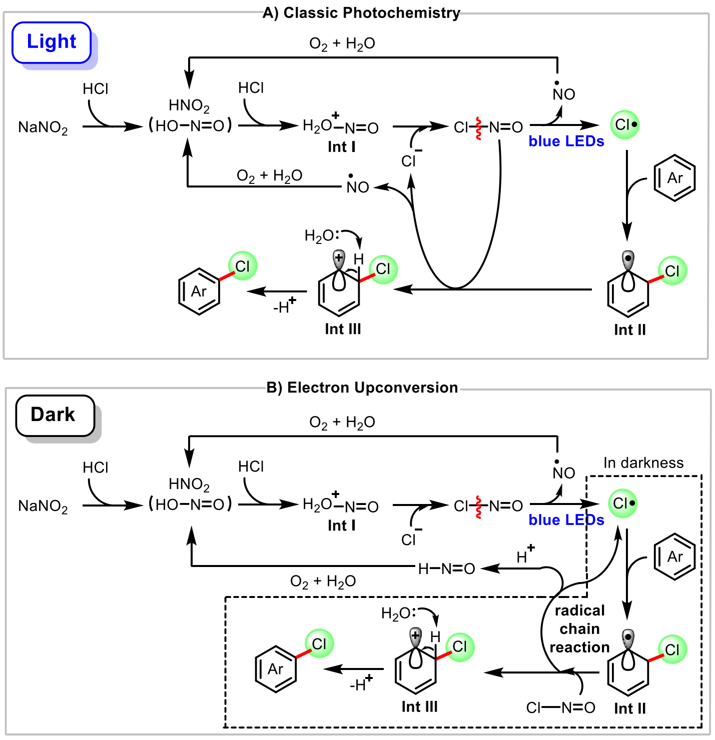

Figure S4. Proposed mechanism of C(sp²)-H chlorination

9.2 Mechanistic studies of aromatic C(sp²)-H bromination reaction

Radical scavenger experiment:

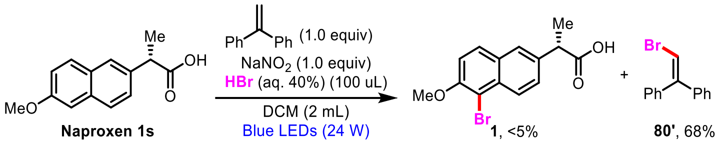

Scheme S15. Radical scavenger experiment

Radical scavenger 1,1-Diphenylethylene (0.2 mmol, 1.0 equiv), Naproxen (0.2 mmol, 1.0 equiv), $NaNO_2$ (0.2 mmol, 1.0 equiv) and HBr (100 *u*L, aq. 40% in water) were dispersed in CH_2Cl_2 (2.0 mL) in a 4 mL glass vial at room temperature. The reaction vial was sealed with a PTEF cap and the reaction mixture vigorously stirred at 24 ℃ under the 24 W blue LEDs (460-470 nm) irradiation for 10 hours. Then, the reaction mixture was extracted with CH_2Cl_2 (3 × 2 mL). The combined organic layer was dried over anhydrous Na_2SO_4, filtered and concentrated. The residue was purified by chromatography on silica gel to afford the 2-Bromo-1,1-diphenylethylene 35 mg (68% isolated yield).

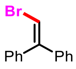

80'

^{1}H NMR (500 MHz, $CDCl_3$) δ 7.45 – 7.38 (m, 3H), 7.35 – 7.30 (m, 5H), 7.25 – 7.22 (m, 2H), 6.80 (s, 1H). Compound **80'** is known and spectra data are consistent with those reported in the literature.[44]

Radical clock experiment:

Compound (1-cyclopropylvinyl)benzene was examined for the bromination reaction in order to prove that bromine radical was produced under our bromination standard conditions and carbon radical intermediate was involved in this bromination process.

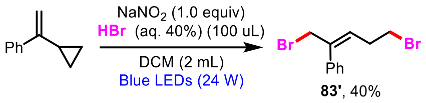

Scheme S16. Radical clock experiment of (1-cyclopropylvinyl)benzene

Cyclopropane-containing alkene (1-cyclopropylvinyl)benzene (0.2 mmol, 1.0 equiv), $NaNO_2$ (0.2 mmol, 1.0 equiv) and HBr (100 *u*L, aq. 40% in water) were dispersed in CH_2Cl_2 (2.0 mL) in a 4 mL glass vial at room temperature. The reaction vial was sealed with a PTEF cap and the reaction mixture vigorously stirred at 24 ℃ under the 24 W blue LEDs (460-470 nm) irradiation for 12 h. Then, the reaction mixture was extracted with CH_2Cl_2 (3 × 2 mL). The combined organic layer was dried over anhydrous Na_2SO_4, filtered and concentrated. The residue was purified by chromatography on silica gel to afford the ring-opening product 24 mg (40% isolated yield).

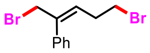

83'

^{1}H NMR (500 MHz, $CDCl_3$) δ 7.47 (d, J = 7.6 Hz, 2H), 7.37 (t, J = 7.4 Hz, 2H), 7.34 – 7.29 (m, 1H), 5.97 (t, J = 7.3 Hz, 1H), 4.36 (s, 2H), 3.54 (t, J = 6.9 Hz, 2H), 2.90 (q, J = 7.0 Hz, 2H).**^{13}C NMR** (126 MHz, $CDCl_3$) δ 140.05, 138.91, 130.76, 128.70, 128.02, 126.20, 32.11, 31.32, 28.78. **HRMS** Calcd for $C_{11}H_{12}{}^{79}Br_2K$ [M+K$^+$]: 340.8937; Found: 340.8947.

10. References

[1] Huang, J.; Sun, F.; Liu, W., Manganese-catalyzed deoxygenation of secondary and tertiary amides under mild conditions. *Journal of Catalysis* **2023,** *423*, 19-25.

[2] Schmidt, B.; Berger, R.; Hoelter, F., Functionalized alkoxy arene diazonium salts from paracetamol. *Organic & Biomolecular Chemistry* **2010,** *8* (6), 1406-1414.

[3] Liu, F.; Bian, Q.; Mao, J.; Gao, Z.; Liu, D.; Liu, S.; Wang, X.; Wang, Y.; Wang, M.; Zhong, J., Asymmetric cross-coupling of racemic α-bromo esters with aryl Grignard reagents catalyzed by cyclopropane-based bisoxazolines cobalt complexes. *Tetrahedron: Asymmetry* **2016,** *27* (14), 663-669.

[4] Adamek, J.; Mazurkiewicz, R.; Węgrzyk, A.; Erfurt, K., 1-Imidoalkylphosphonium salts with modulated C_{α}–P^{+} bond strength: synthesis and application as new active α-imidoalkylating agents. *Beilstein Journal of Organic Chemistry* **2017,** *13*, 1446-1455.

[5] Zheng, Y.; Hu, Q.-Q.; Huang, Q.; Xie, Y., Late-Stage C–H Nitration of Unactivated Arenes by $Fe(NO3)_3$·9H2O in Hexafluoroisopropanol. *Organic letters* **2024,** *26* (15), 3316-3320.

[6] Song, S.; Li, X. Y.; Wei, J. L.; Wang, W. J.; Zhang, Y. Q.; Ai, L. S.; Zhu, Y. C.; Shi, X. M.; Zhang, X. H.; Jiao, N., DMSO-catalysed late-stage chlorination of (hetero)arenes. *Nat Catal* **2020,** *3* (2), 107-115.

[7] Shimizu, A.; Hayashi, R.; Ashikari, Y.; Nokami, T.; Yoshida, J.-i., Switching the reaction pathways of electrochemically generated β-haloalkoxysulfonium ions – synthesis of halohydrins and epoxides. *Beilstein Journal of Organic Chemistry* **2015,** *11*, 242-248.

[8] Yu, D.; Ji, R.; Sun, Z.; Li, W.; Liu, Z.-Q., Electrochemical chlorination and bromination of electron-deficient C H bonds in quinones, coumarins, quinoxalines and 1,3-diketones. *Tetrahedron Letters* **2021,** *86*.

[9] Singh, P. K.; Khanna, R. N., Selective Halogenation of 1,4-Benzoquinones and 1,4-Naphthoquinones with Copper(II) Halide Adsorbed on Alumina. *Synthetic Communications* **1993,** *23* (15), 2083-2089.

[10] Nakazaki, A.; Huang, W.-Y.; Koga, K.; Yingyongnarongkul, B.-e.; Boonsombat, J.; Sawayama, Y.; Tsujimoto, T.; Nishikawa, T., Structural Study on a Naturally Occurring Terphenyl Quinone. *Bioscience, Biotechnology, and Biochemistry* **2014,** *77* (7), 1529-1532.

[11] McComas, C. C.; Perales, J. B.; Van Vranken, D. L., Synthesis of (+/-)-madindolines and chemical models. Studies of chemical reactivity. *Organic letters* **2002,** *4* (14), 2337-40.

[12] Xu, Z.; Jia, R.; Ma, Z.; Cao, S.; Shen, L.; Ji, H., Iron-Catalyzed Radical Methylation of Activated Alkenes with tert-Butanol as the Methyl Source. *Synlett* **2019**.

[13] Pochorovski, I.; Boudon, C.; Gisselbrecht, J. P.; Ebert, M. O.; Schweizer, W. B.;

Diederich, F., Quinone-based, redox-active resorcin[4]arene cavitands. *Angewandte Chemie (International ed. in English)* **2012,** *51* (1), 262-6.
[14] Prasad, P. K.; Sudalai, A., Copper(I) Bromide-Catalyzed Carbonylative Coupling of Aryl Halides with Phenols, Alcohols and Amines using Sodium Cyanide as C1 Source: A Synthesis of Carboxylic Acid Derivatives. *Advanced Synthesis & Catalysis* **2014,** *356* (10), 2231-2238.
[15] Ghosh, I.; Khamrai, J.; Savateev, A.; Shlapakov, N.; Antonietti, M.; König, B., Organic semiconductor photocatalyst can bifunctionalize arenes and heteroarenes. *Science* **2019,** *365* (6451), 360-366.
[16] Monde, K.; Satoh, H.; Nakamura, M.; Tamura, M.; Takasugi, M., Organochlorine Compounds from a Terrestrial Higher Plant: Structures and Origin of Chlorinated Orcinol Derivatives from Diseased Bulbs of Lilium maximowiczii. *Journal of Natural Products* **1998,** *61* (7), 913-921.
[17] Vantourout, J. C.; Law, R. P.; Isidro-Llobet, A.; Atkinson, S. J.; Watson, A. J. B., Chan–Evans–Lam Amination of Boronic Acid Pinacol (BPin) Esters: Overcoming the Aryl Amine Problem. *The Journal of Organic Chemistry* **2016,** *81* (9), 3942-3950.
[18] Zhang, J.; Peng, J. F.; Wang, T.; Kang, Y.; Jing, S.; Zhang, Z. T., Synthesis and biological evaluation of arylpyrazoles as fungicides against phytopathogenic fungi. *Molecular diversity* **2017,** *21* (2), 317-323.
[19] Yuan, Y.; Yao, A.; Zheng, Y.; Gao, M.; Zhou, Z.; Qiao, J.; Hu, J.; Ye, B.; Zhao, J.; Wen, H.; Lei, A., Electrochemical Oxidative Clean Halogenation Using HX/NaX with Hydrogen Evolution. *iScience* **2019,** *12*, 293-303.
[20] Luo, B.-T.; Liu, H.; Lin, Z.-J.; Jiang, J.; Shen, D.-S.; Liu, R.-Z.; Ke, Z.; Liu, F.-S., Aerobic and Efficient Direct Arylation of Five-Membered Heteroarenes and Their Benzocondensed Derivatives with Aryl Bromides by Bulky α-Hydroxyimine Palladium Complexes. *Organometallics* **2015,** *34* (20), 4881-4894.
[21] Wang, W.; Yang, X.; Dai, R.; Yan, Z.; Wei, J.; Dou, X.; Qiu, X.; Zhang, H.; Wang, C.; Liu, Y.; Song, S.; Jiao, N., Catalytic Electrophilic Halogenation of Arenes with Electron-Withdrawing Substituents. *Journal of the American Chemical Society* **2022,**

144 (29), 13415-13425.

[22] Li, Z. L.; Sun, K. K.; Cai, C., Cobalt(ii)-catalyzed regioselective C-H halogenation of anilides. *Organic & Biomolecular Chemistry* **2018,** *16* (30), 5433-5440.

[23] Fosu, S. C.; Hambira, C. M.; Chen, A. D.; Fuchs, J. R.; Nagib, D. A., Site-Selective C-H Functionalization of (Hetero)Arenes via Transient, Non-symmetric lodanes. *Chem* **2019,** *5* (2), 417-428.

[24] Mondal, H.; Patra, S.; Saha, S.; Nayak, T.; Sengupta, U.; Sudan Maji, M., Late-Stage Halogenation of Peptides, Drugs and (Hetero)aromatic Compounds with a Nucleophilic Hydrazide Catalyst. *Angewandte Chemie International Edition* **2023,** *62* (51).

[25] Kim, S.; Matsubara, R.; Hayashi, M., Activated Carbon-Promoted Dehydrogenation of Hydroquinones to Benzoquinones, Naphthoquinones, and Anthraquinones under Molecular Oxygen Atmosphere. *The Journal of Organic Chemistry* **2019,** *84* (5), 2997-3003.

[26] Sprang, F.; Herszman, J. D.; Waldvogel, S. R., Electrochemical oxidation of phenols in flow: a versatile and scalable access topara-benzoquinones. *Green Chemistry* **2022,** *24* (13), 5116-5124.

[27] (a) Royuela, S.; Almarza, J.; Mancheño, M. J.; Pérez-Flores, J. C.; Michel, E. G.; Ramos, M. M.; Zamora, F.; Ocón, P.; Segura, J. L., Synergistic Effect of Covalent Bonding and Physical Encapsulation of Sulfur in the Pores of a Microporous COF to Improve Cycling Performance in Li-S Batteries. *Chemistry – A European Journal* **2019,** *25* (53), 12394-12404. (b) Tania, L.; David J. D. W.; Jason L. D., On the potential intermediacy of PhIBr2 as a brominating agent. *Org. Biomol. Chem*, **2022**, *20*, 8454-8460.

[28] (a) Petzold, D.; König, B., Photocatalytic Oxidative Bromination of Electron-Rich Arenes and Heteroarenes by Anthraquinone. *Advanced Synthesis & Catalysis* **2017,** *360* (4), 626-630. (b) Li, C.; Cheng, Y.; Pang, F.; Yan, X.; Huang, Z.; Wang, X.; Li, Y.; Wang, H.; Xu, H., Halogenation of arenes using alkali metal halides/$Fe(NO_3)_3 \cdot 9H_2O$ at room temperature. *RSC Adv.* **2025,** *15,* 8523-8528.

[29] Kikushima, K.; Moriuchi, T.; Hirao, T., Vanadium-catalyzed oxidative bromination promoted by Bronsted acid or Lewis acid. *Tetrahedron* **2010,** *66* (34), 6906-6911.
[30] Mondal, M.; Puranik, V. G.; Argade, N. P., A Facile Phenol-Driven Intramolecular Diastereoselective Thermal/Base-Catalyzed Dipolar [2 + 2] Annulation Reactions: An Easy Access to Complex Bioactive Natural and Unnatural Benzopyran Congeners. *The Journal of Organic Chemistry* **2007,** *72* (6), 2068-2076.
[31] (a) Zou, Q.; Wang, C.; Smith, J.; Xue, D.; Xiao, J., Alkylation of Amines with Alcohols and Amines by a Single Catalyst under Mild Conditions. *Chemistry – A European Journal* **2015,** *21* (27), 9656-9661. (b) Ma, X.; Yu, J.; Jiang, M.; Wang, M.;Tang, L.; Wei, M.; Zhou, Q., Mild and Regioselective Bromination of Phenols with TMSBr. *Eur. J. Org. Chem.* **2019**, *28*, 4593-4596.
[32] Du, Y. B.; Xi, Z. G.; Guo, L. R.; Lu, H. F.; Feng, L.; Gao, H. Y., Practical bromination of arylhydroxylamines with $SOBr_2$ towards ortho-bromo-anilides. *Tetrahedron Letters* **2021,** *72*.
[33] Kumar, R.; Turcaud, S.; Micouin, L., The Reaction of Dimethylalkynylaluminum Reagents with Trimethylsilyldiazomethane: Original Reactivity Leading to New α-Silylated Alkynyl Hydrazones. *Organic letters* **2014,** *16* (23), 6192-6195.
[34] Jiang, P.-P.; Yang, X.-J., A quick, mild and efficient bromination using a CFBSA/KBr system. *Rsc Advances* **2016,** *6* (93), 90031-90034.
[35] Wang, N.-X.; Li, J.-H.; Tang, S.; Xie, Y.-X., Halogenation of Indoles with Copper(II) Halides: Selective Synthesis of 2-Halo-, 3-Halo-, and 2,3-Dibromoindoles. *Synthesis* **2007,** *2007* (10), 1535-1541.
[36] Katrun, P.; Kuhakarn, C., K2S2O8-Mediated halogenation of 2-arylimidazo[1,2-a]pyridines using sodium halides as the halogen sources. *Tetrahedron Letters* **2019,** *60* (14), 989-993.
[37] Patil, S. M.; Mascarenhas, M.; Sharma, R.; Mohana Roopan, S.; Roychowdhury, A., Microwave-Assisted One-Pot Synthesis of Substituted 3-Bromoimidazo[1,2-a]pyridines and Imidazoheterocycles. *Journal of Heterocyclic Chemistry* **2014,** *51* (5), 1509-1515.

[38] Lam, K. H.; Gambari, R.; Yuen, M. C. W.; Kan, C. W.; Chan, P.; Xu, L.; Tang, W.; Chui, C. H.; Cheng, G. Y. M.; Wong, R. S. M.; Lau, F. Y.; Tong, C. S. W.; Chan, A. K. W.; Lai, P. B. S.; Kok, S. H. L.; Cheng, C. H.; Chan, A. S. C.; Tang, J. C. O., The preparation of 2,6-disubstituted pyridinyl phosphine oxides as novel anti-cancer agents. *Bioorganic & Medicinal Chemistry Letters* **2009,** *19* (8), 2266-2269.

[39] Lu, H.-K.; Liu, T.; Shi, Z.; Yan, H.; Li, Z.; Ye, K.-Y., Electrochemical Bromination of Substituted Thiophenes in Batch and Continuous Flow. *European Journal of Organic Chemistry* **2023,** *26* (7), e202200963.

[40] Zhang, M.; Su, J.; Zhang, Y.; Chen, M.; Li, W.; Qin, X.; Xie, Y.; Qin, L.; Huang, S., A Copper Halide Promoted Regioselective Halogenation of Coumarins Using N-Halosuccinimide as Halide Source. *Synlett* **2019,** *30* (05), 630-634.

[41] Maegawa, T.; Shibata, A.; Kitamoto, S.; Fujimura, K.; Hirose, Y.; Hamamoto, H.; Nakamura, A.; Miki, Y., Dehydroxymethyl Bromination of Alkoxybenzyl Alcohols by Using a Hypervalent Iodine Reagent and Lithium Bromide. *Synlett* **2018,** *29* (17), 2275-2278.

[42] Ren, H.; Zhang, Z.; Xu, L.; Chen, Z.; Liu, Z.; Miao, M.; Song, J., Nickel-Catalyzed Regioselective Reductive Cross-Coupling of Aryl Halides with Polysubstituted Allyl Halides in the Presence of Imidazolium Salts. *Synlett* **2015,** *26* (20), 2784-2788.

[43] Zhang, S.; Ullah, A.; Yamamoto, Y.; Almansour, A. I.; Arumugam, N.; Kumar, R. S.; Bao, M., Copper(II)-Catalyzed and Chelation-Induced Remote C-H Halogenation of Quinolines under Neutral Conditions. *ChemistrySelect* **2017,** *2* (12), 3414-3418.

[44] Fu, N.; Sauer, G. S.; Lin, S., Electrocatalytic Radical Dichlorination of Alkenes with Nucleophilic Chlorine Sources. *Journal of the American Chemical Society* **2017,** *139* (43), 15548-15553.

[45] Tan, Y.-F.; Zhao, Y.-N.; Yang, D.; Lv, J.-F.; Guan, Z.; He, Y.-H., Electrochemical Synthesis of β-Iodoesters by 1,2-Iodoesterization of Unactivated Alkenes with Carboxylic Acids and Tetrabutylammonium Iodide. *The Journal of Organic Chemistry* **2023,** *88* (8), 5161-5171.
