# Supplementary material for: Visible-light promoted late-stage chlorination and bromination of quinones and (hetero)arenes utilizing aqueous HCl or HBr as halogen donors
Source: Commun Chem. 2025 Dec 11;9:23. doi: 10.1038/s42004-025-01831-5 (PMC12804898; doi:10.1038/s42004-025-01831-5)
Supplement: Supplementary file 3 — Description of Additional Supplementary Files [file 42004_2025_1831_MOESM3_ESM.pdf]

Description of Additional Supplementary Files:

File: Supplementary Data 1

Description: Copies of NMR spectra**.**

File: Supplementary Data 2

Description: Source data for Table 1.

File: Supplementary Data 3

Description: Source data for Figure 3C.
