# Supplementary material for: Visible-light promoted late-stage chlorination and bromination of quinones and (hetero)arenes utilizing aqueous HCl or HBr as halogen donors
Source: Commun Chem. 2025 Dec 11;9:23. doi: 10.1038/s42004-025-01831-5 (PMC12804898; doi:10.1038/s42004-025-01831-5)
Supplement: Supplementary file 4 — Dataset 1 [file 42004_2025_1831_MOESM4_ESM.pdf]

^{1}H NMR, ^{13}C NMR and ^{19}F NMR spectra

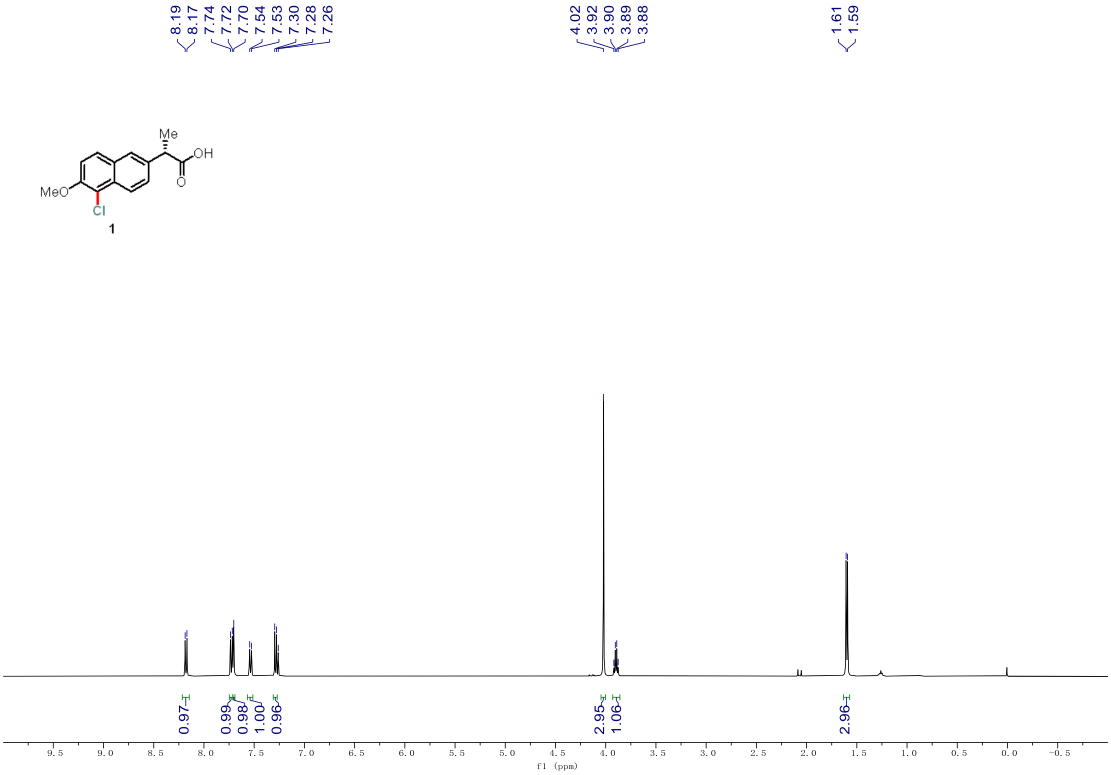

^{1}H NMR of compound **1**

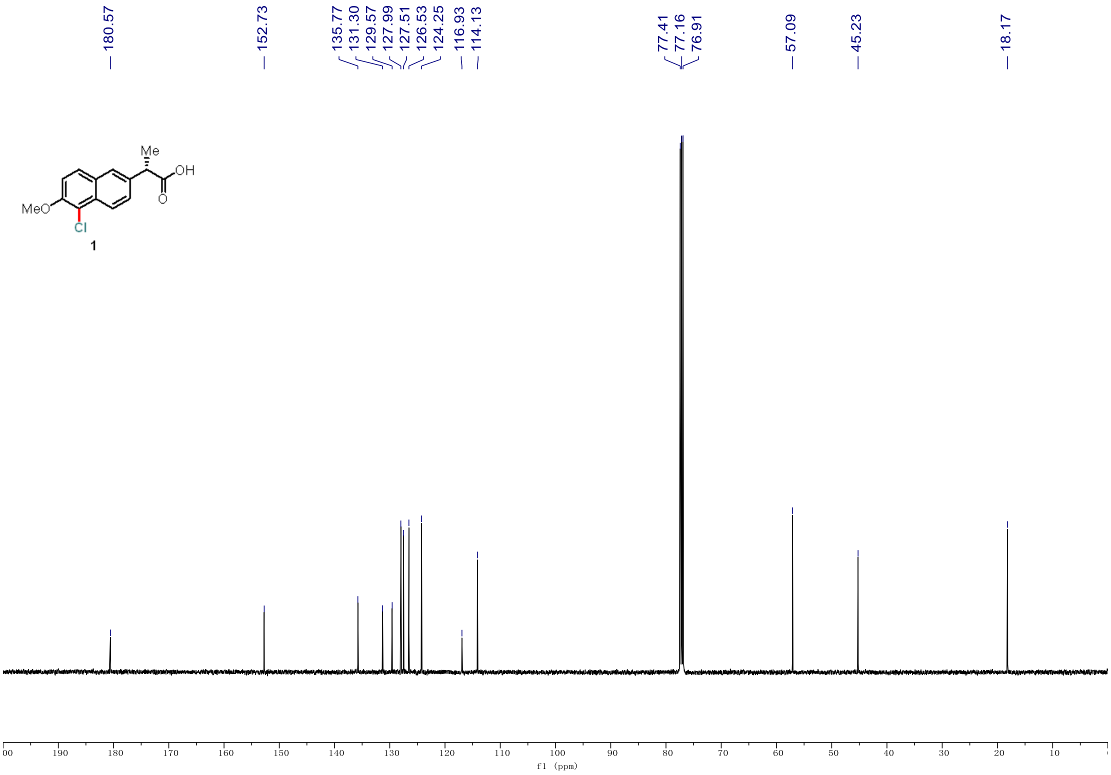

^{13}C NMR of compound **1**

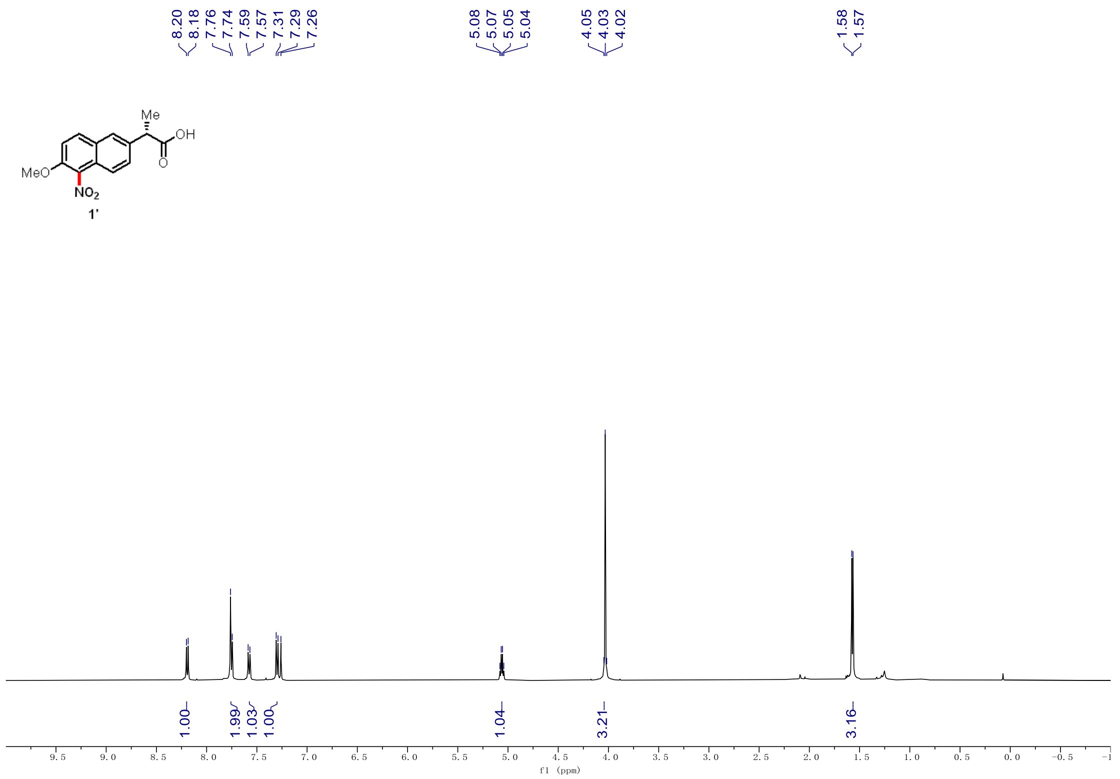

^{1}H NMR of compound **1'**

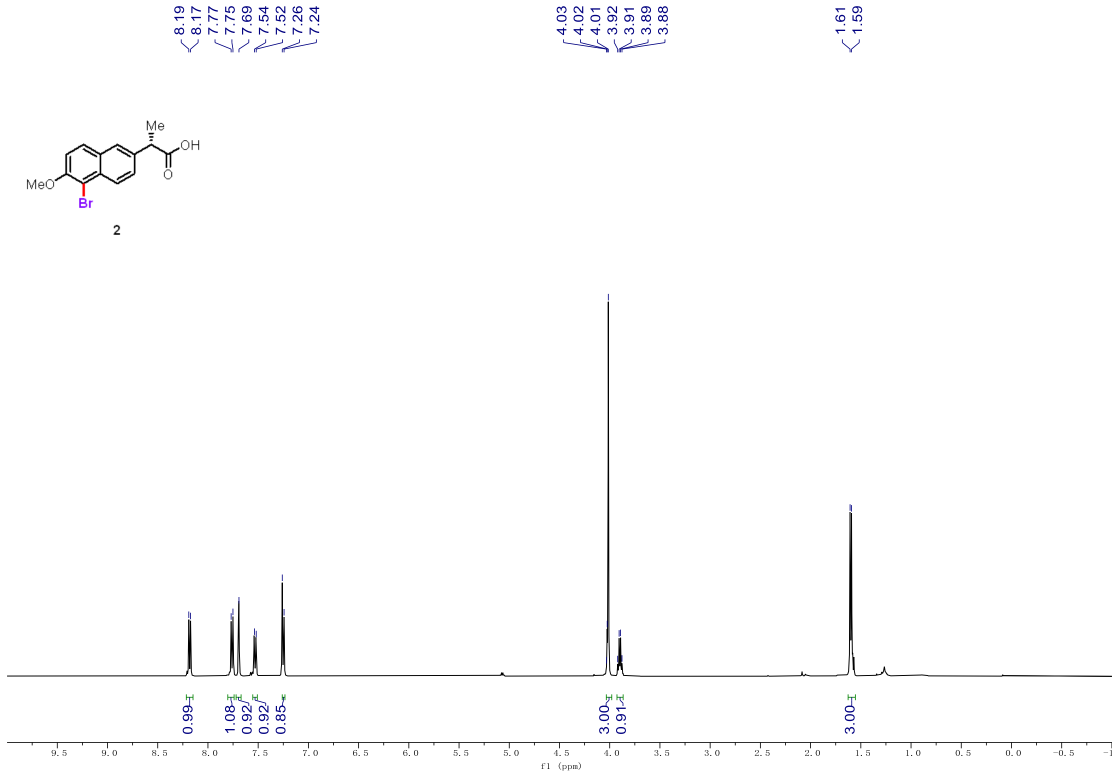

^{1}H NMR of compound **2**

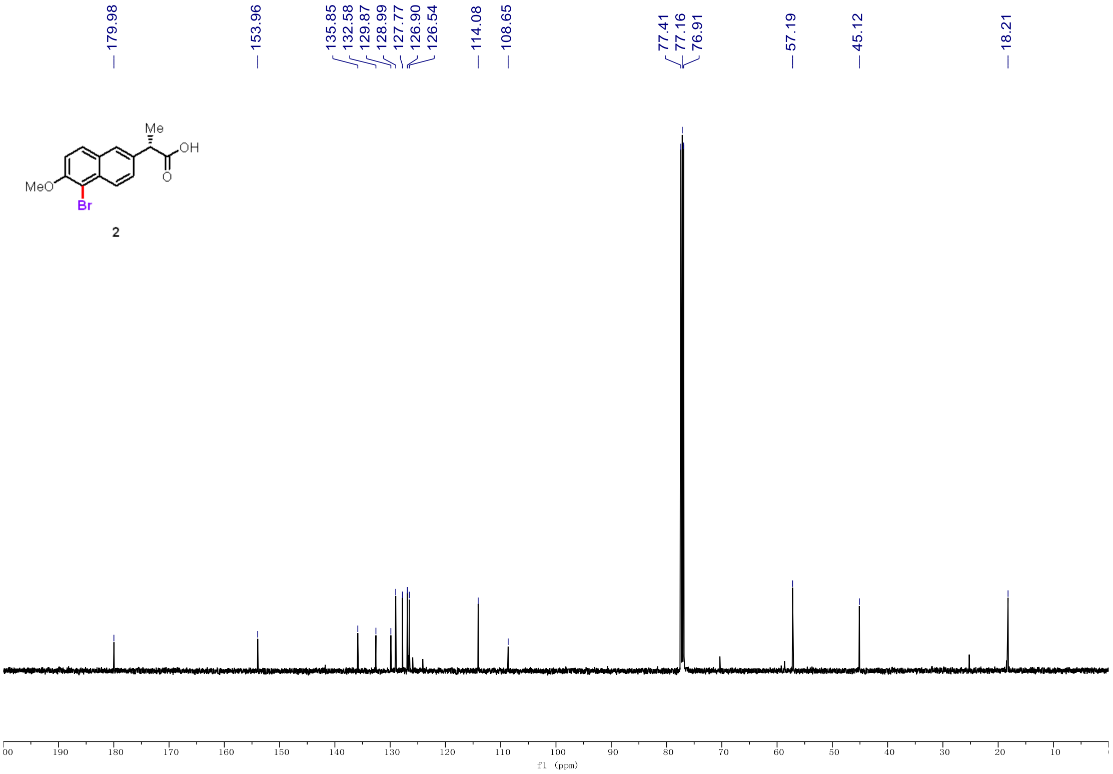

^{13}C NMR of compound **2**

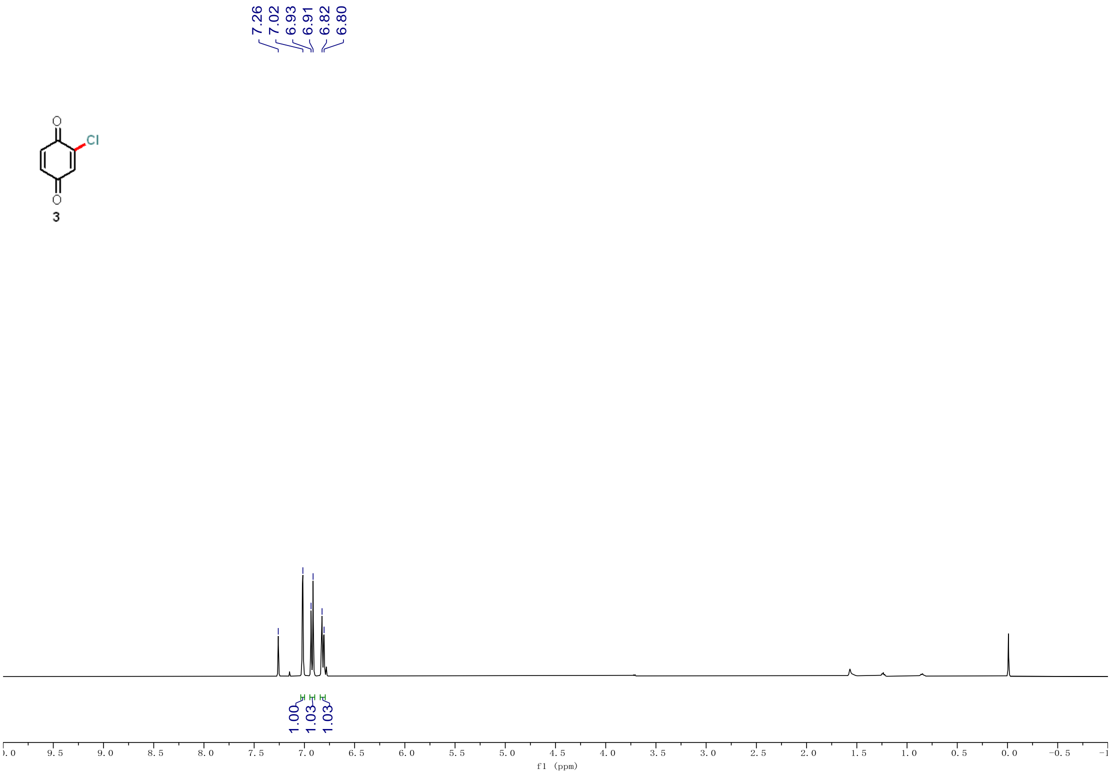

^{1}H NMR of compound **3**

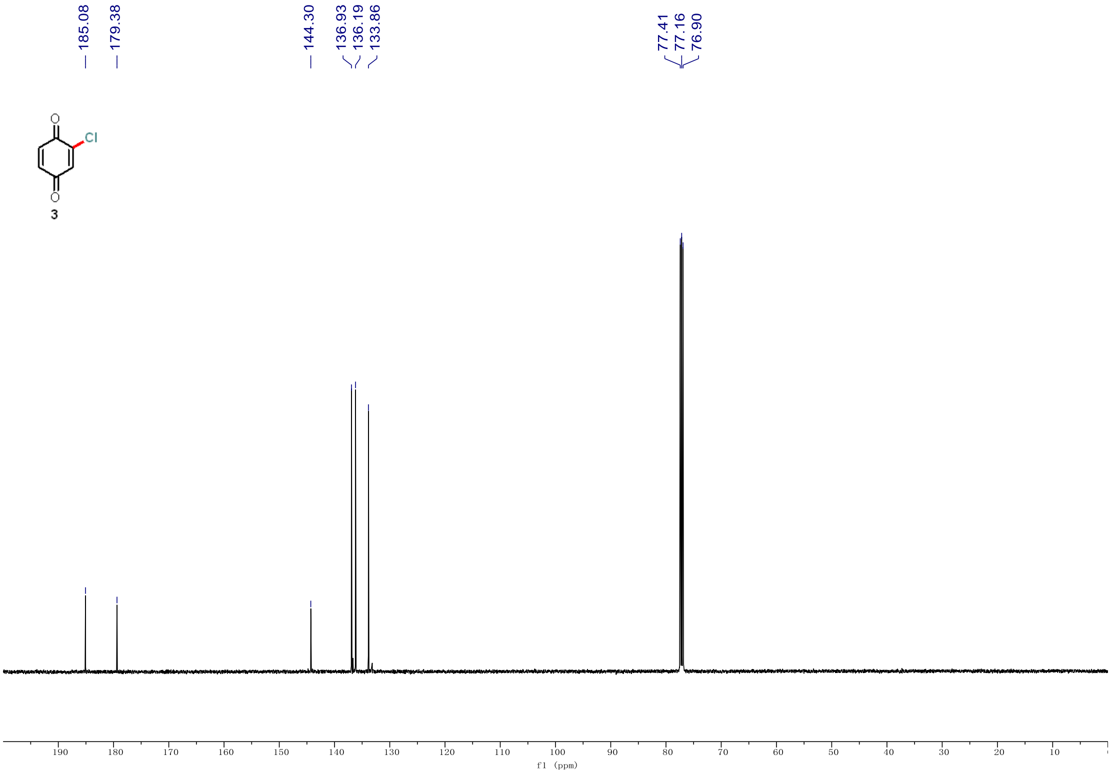

^{13}C NMR of compound **3**

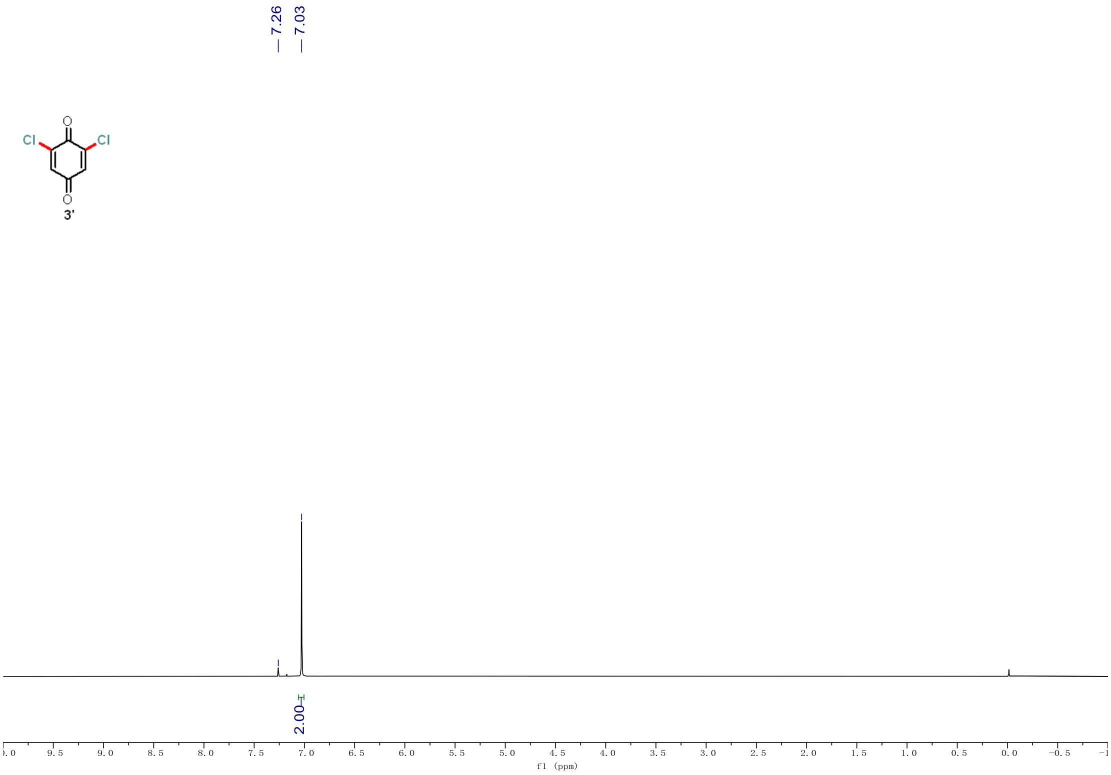

^{1}H NMR of compound **3'**

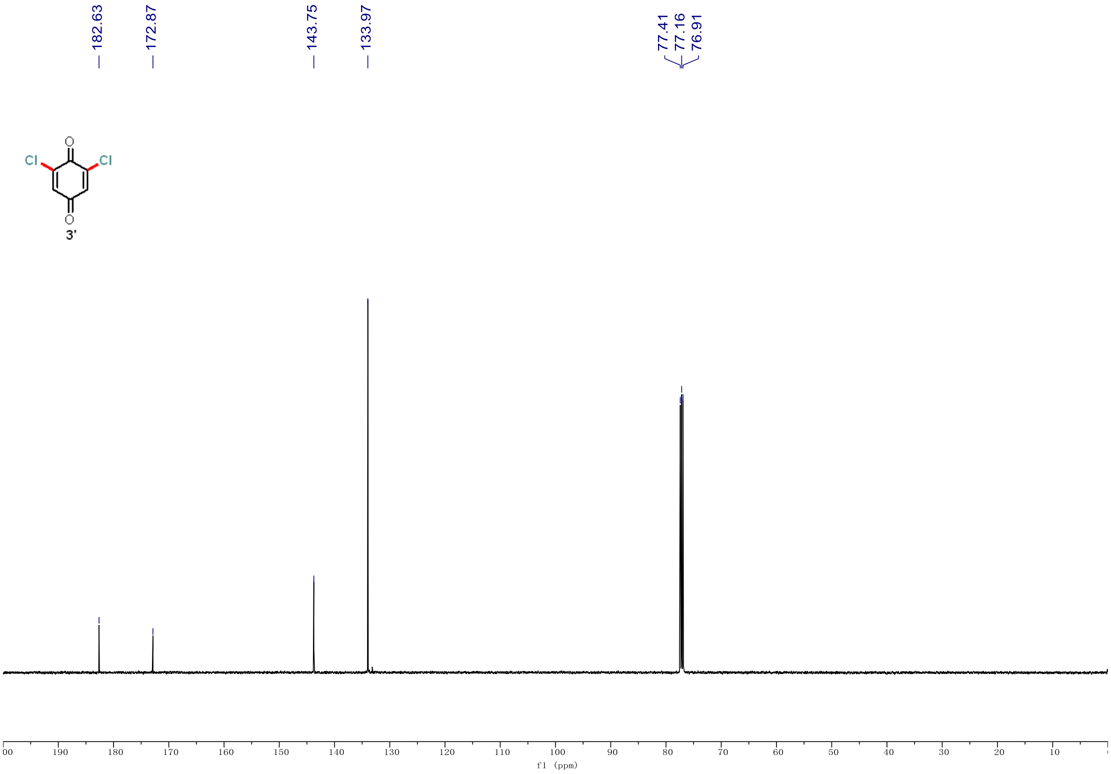

^{13}C NMR of compound **3'**

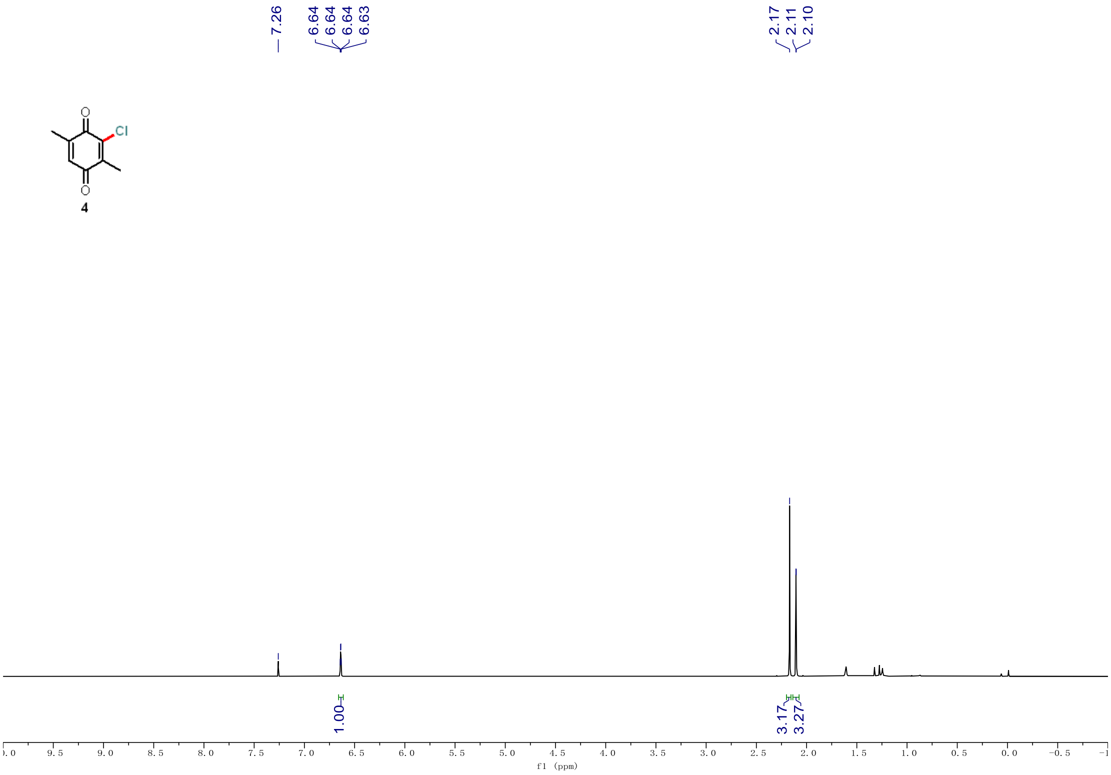

^{1}H NMR of compound **4**

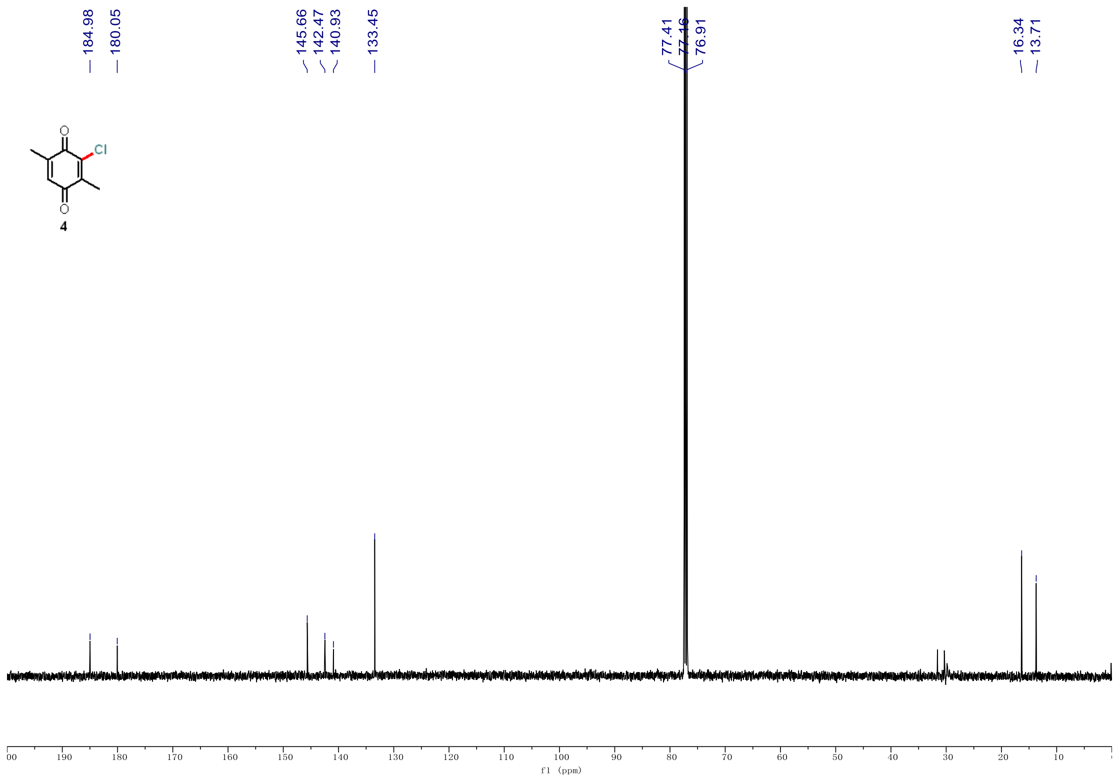

^{13}C NMR of compound **4**

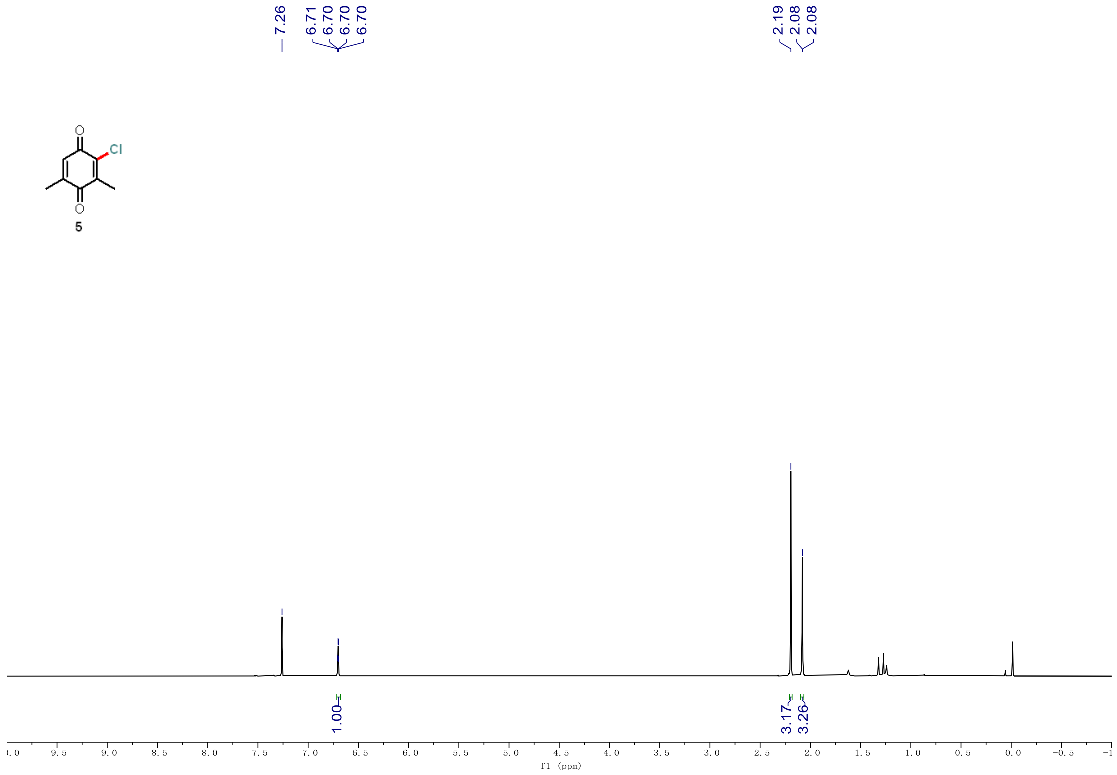

^{1}H NMR of compound **5**

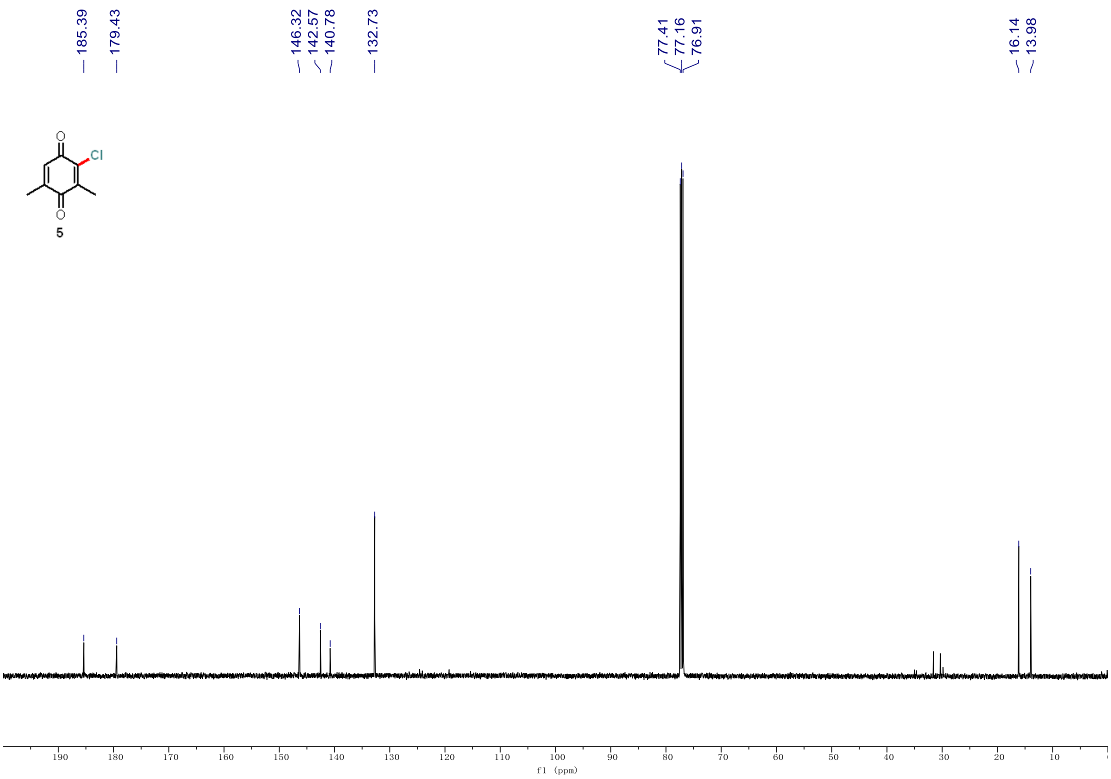

^{13}C NMR of compound **5**

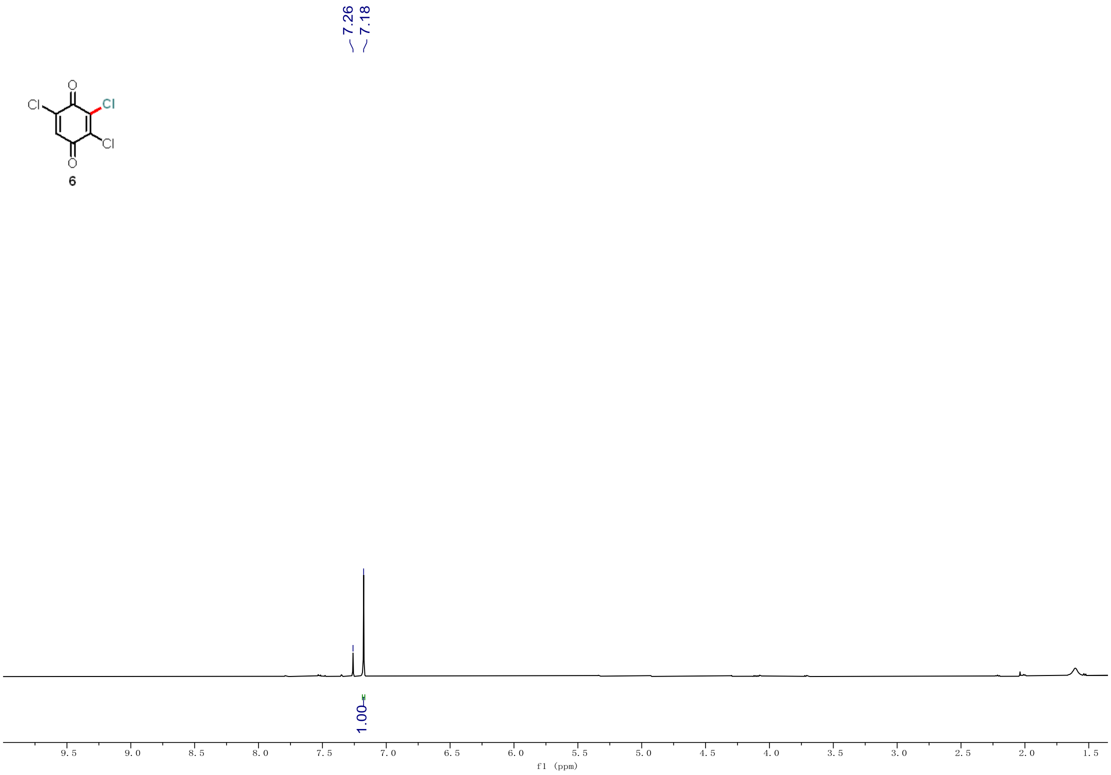

^{1}H NMR of compound **6**

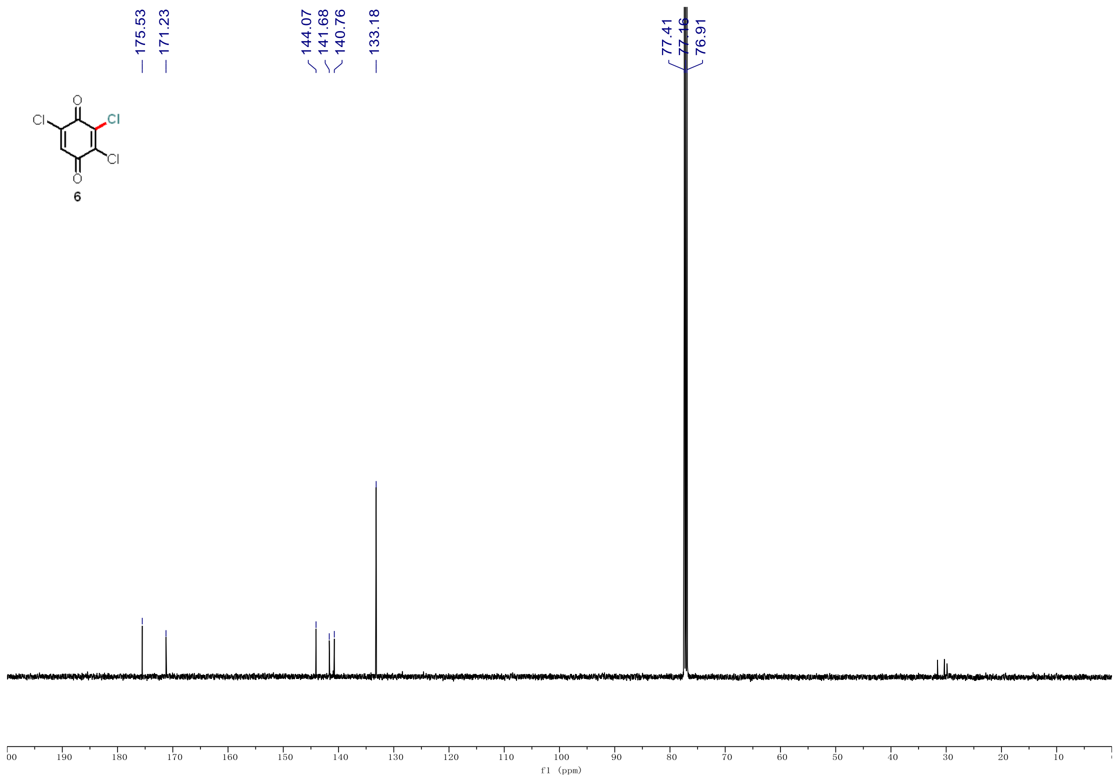

^{13}C NMR of compound **6**

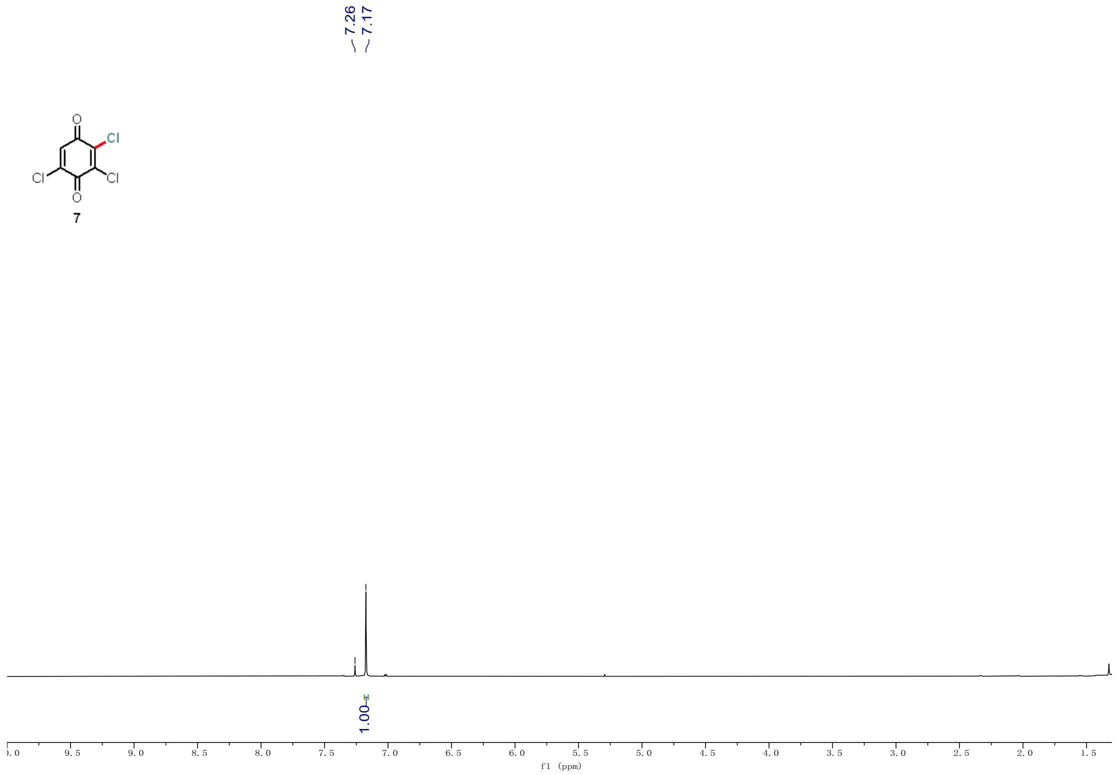

^{1}H NMR of compound **7**

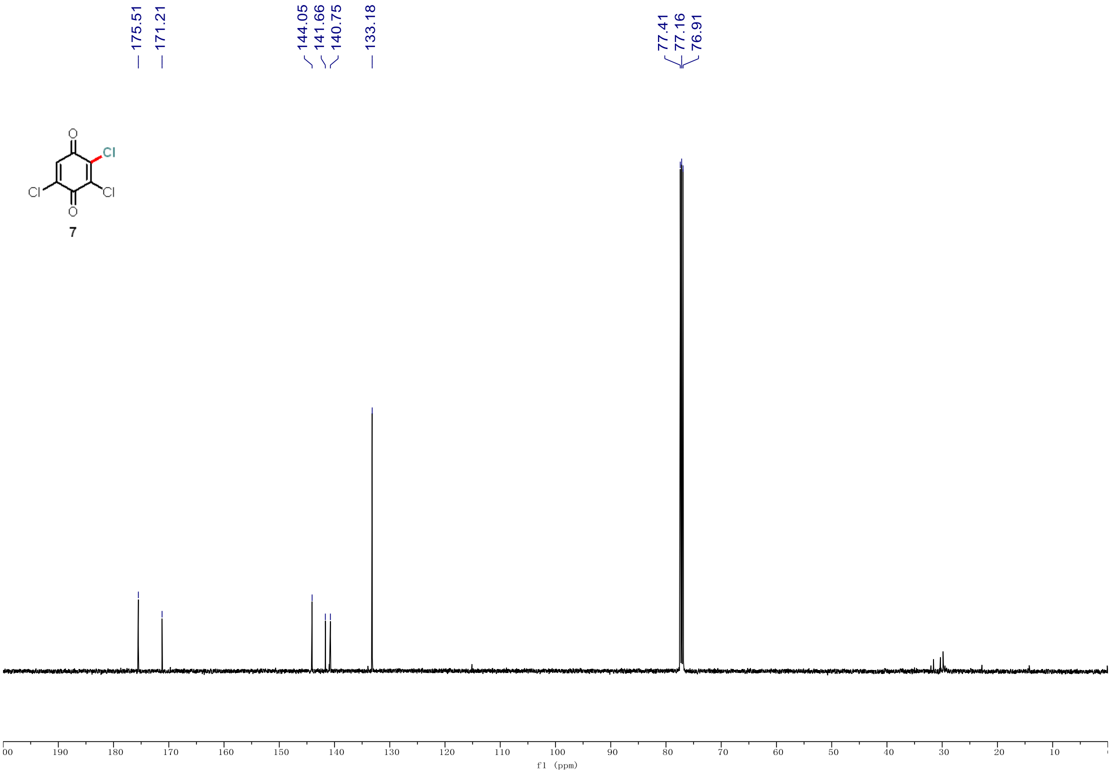

^{13}C NMR of compound **7**

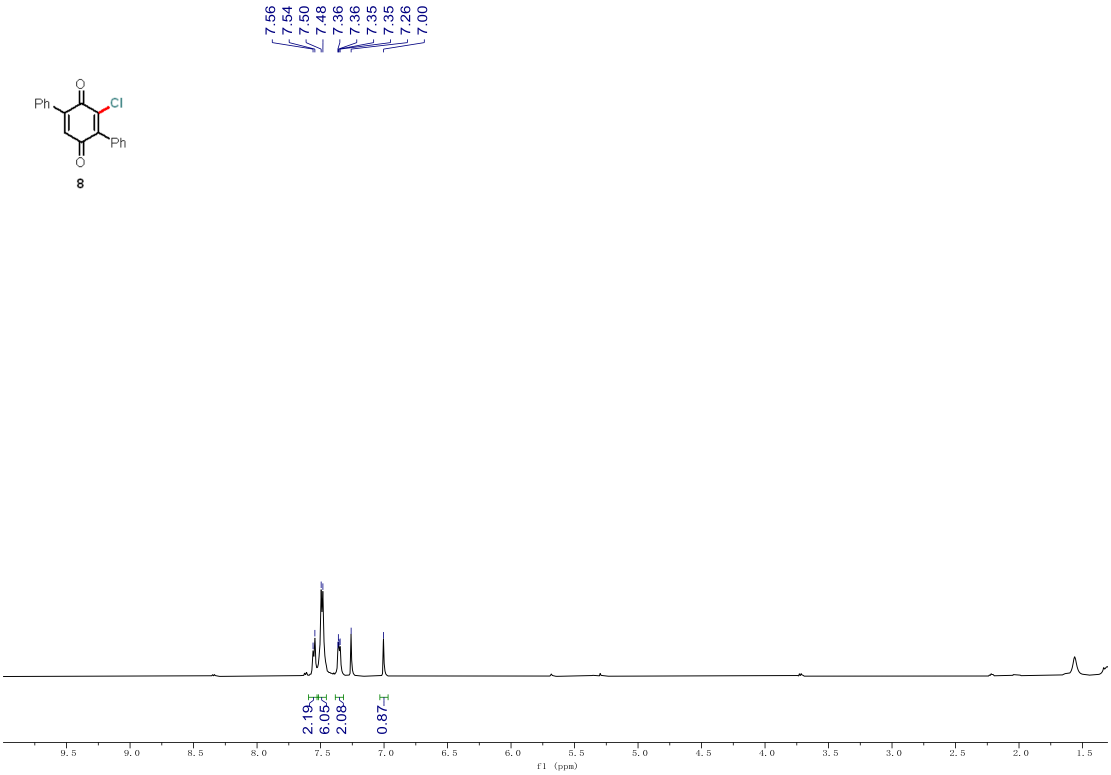

^{1}H NMR of compound **8**

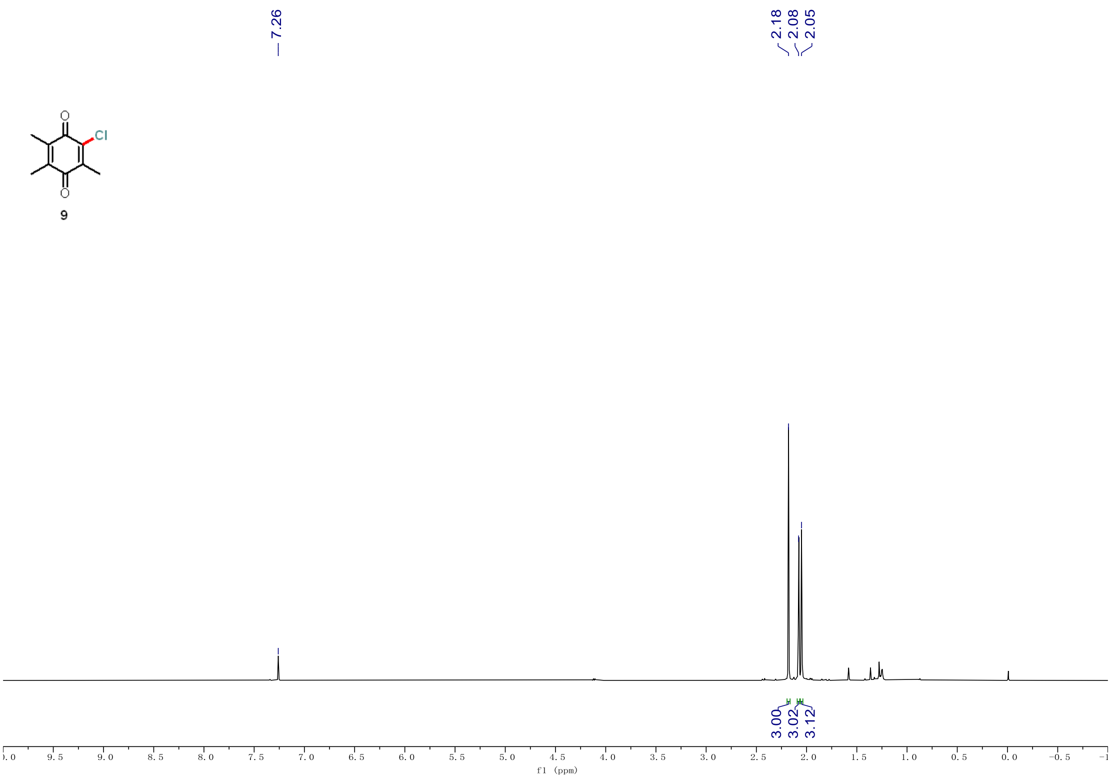

^{1}H NMR of compound **9**

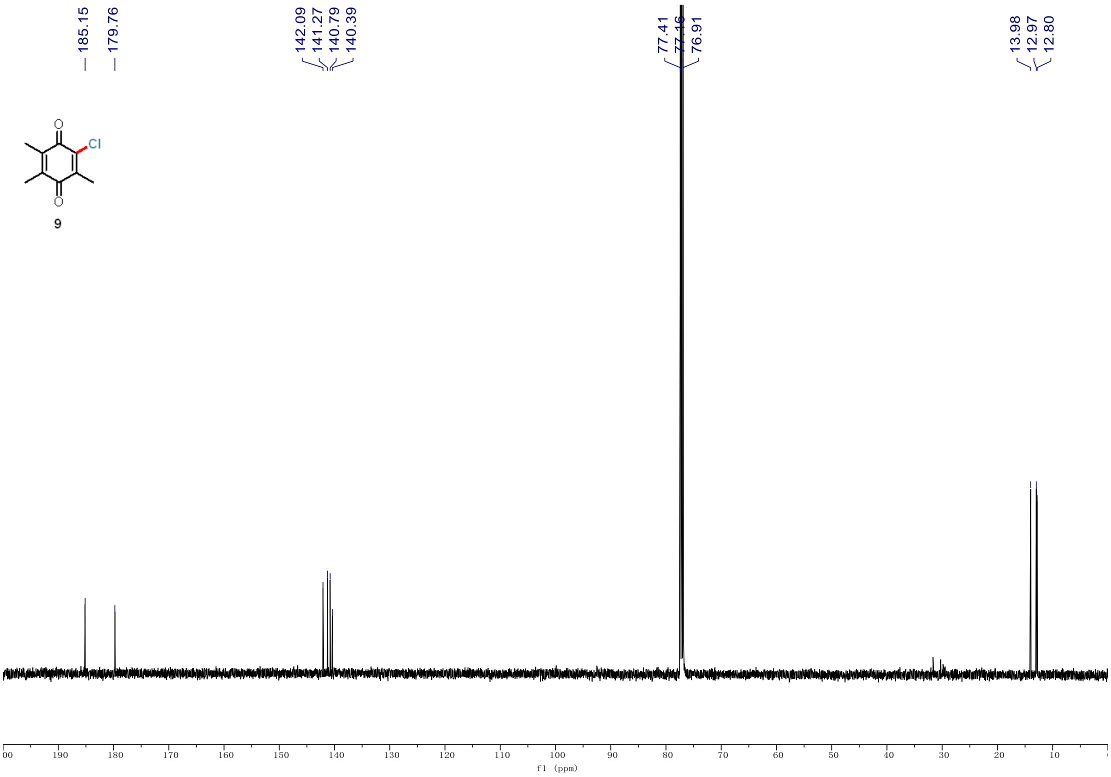

^{13}C NMR of compound **9**

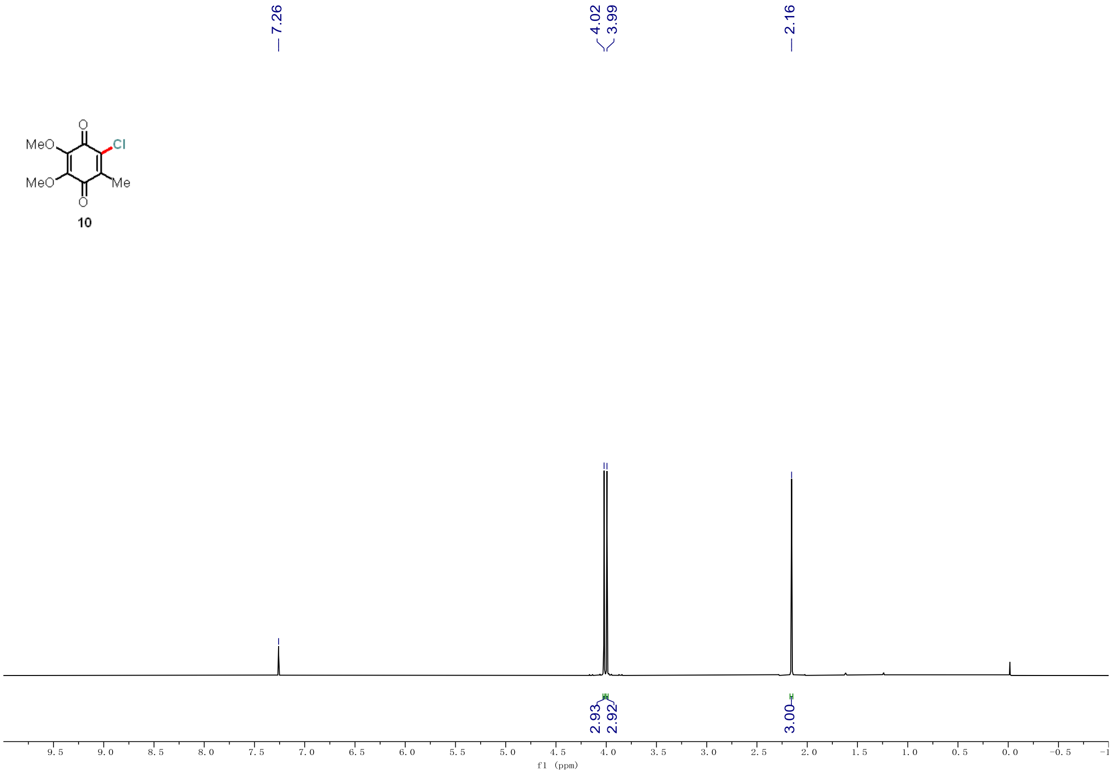

^{1}H NMR of compound **10**

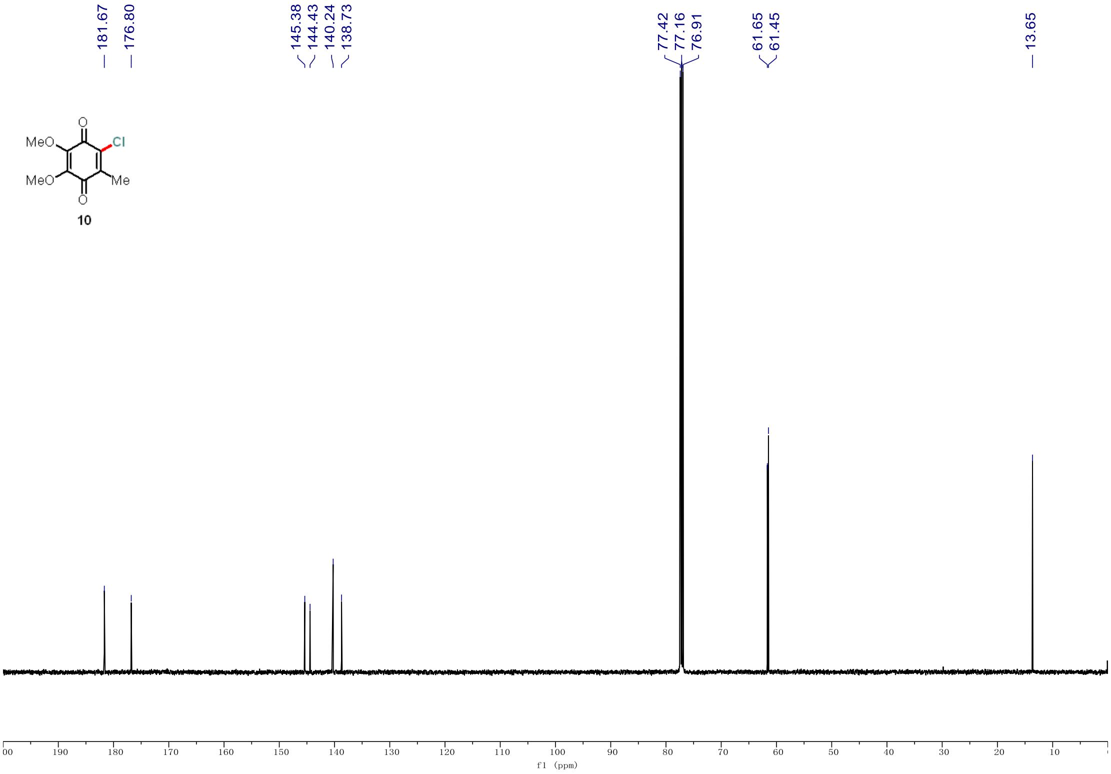

^{13}C NMR of compound **10**

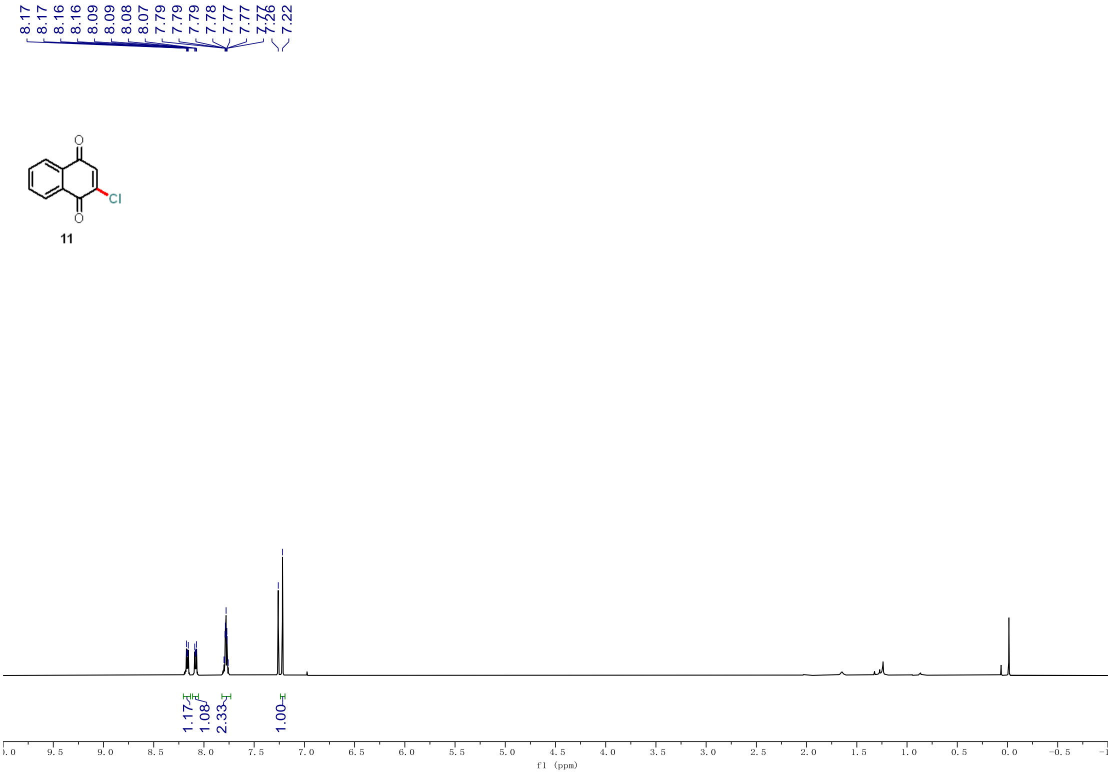

^{1}H NMR of compound **11**

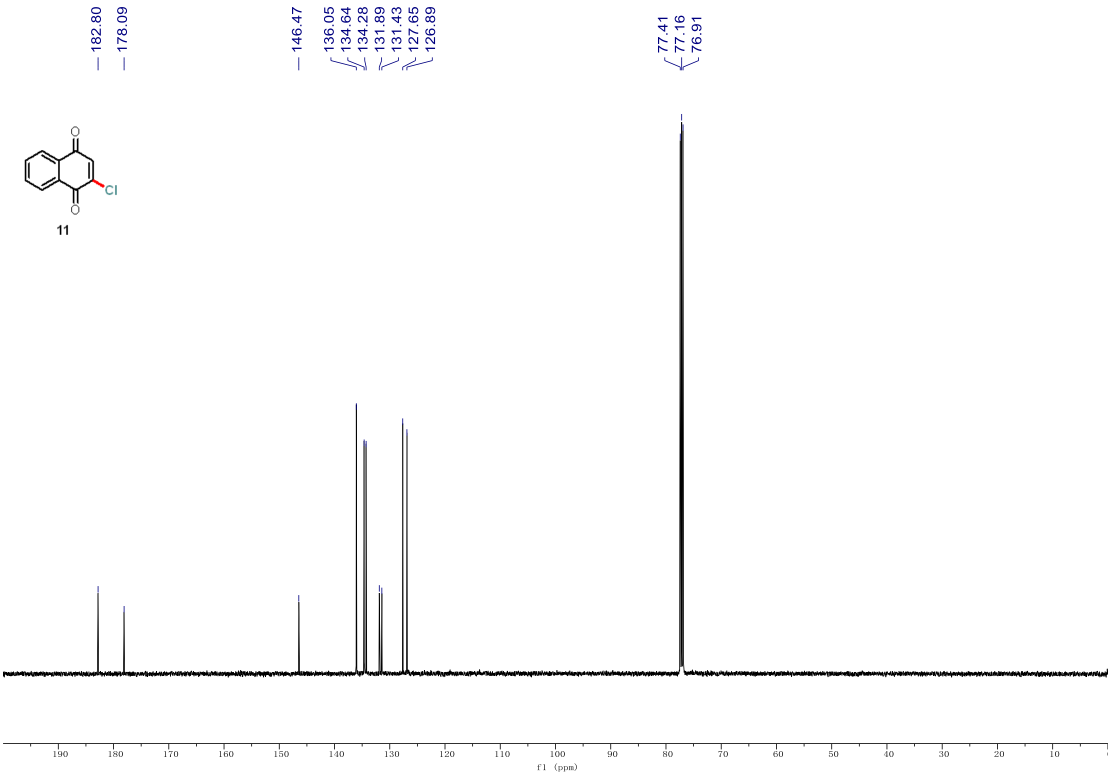

^{13}C NMR of compound **11**

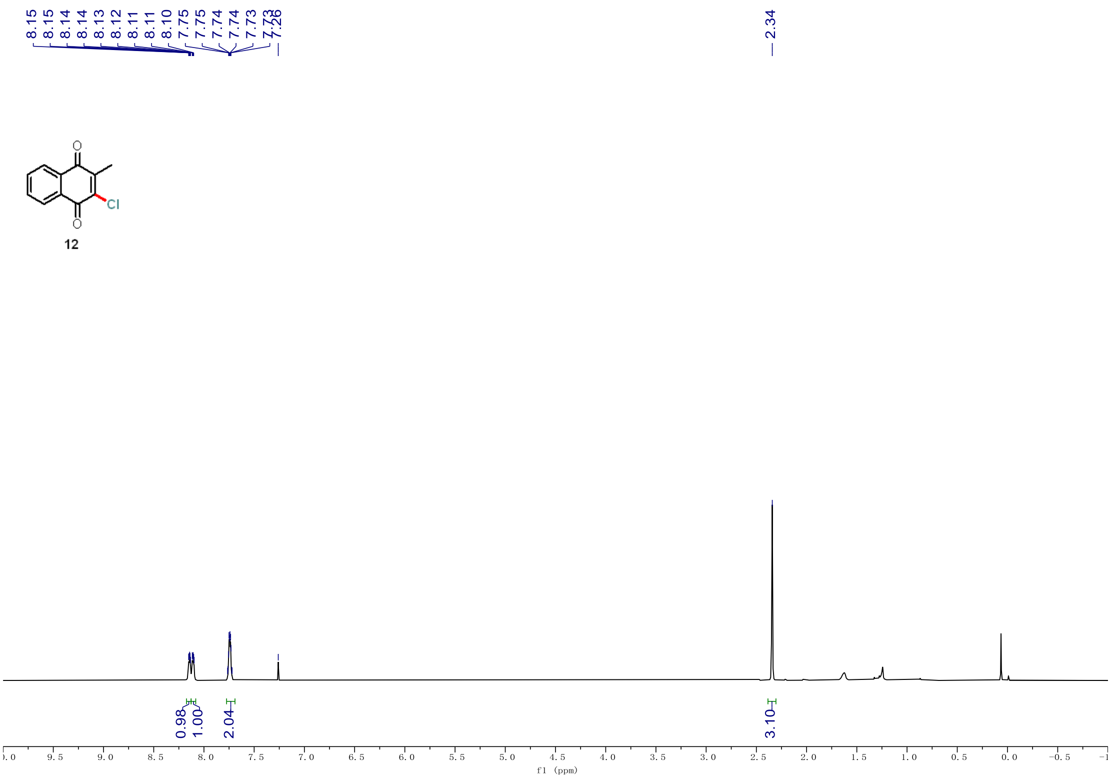

^{1}H NMR of compound **12**

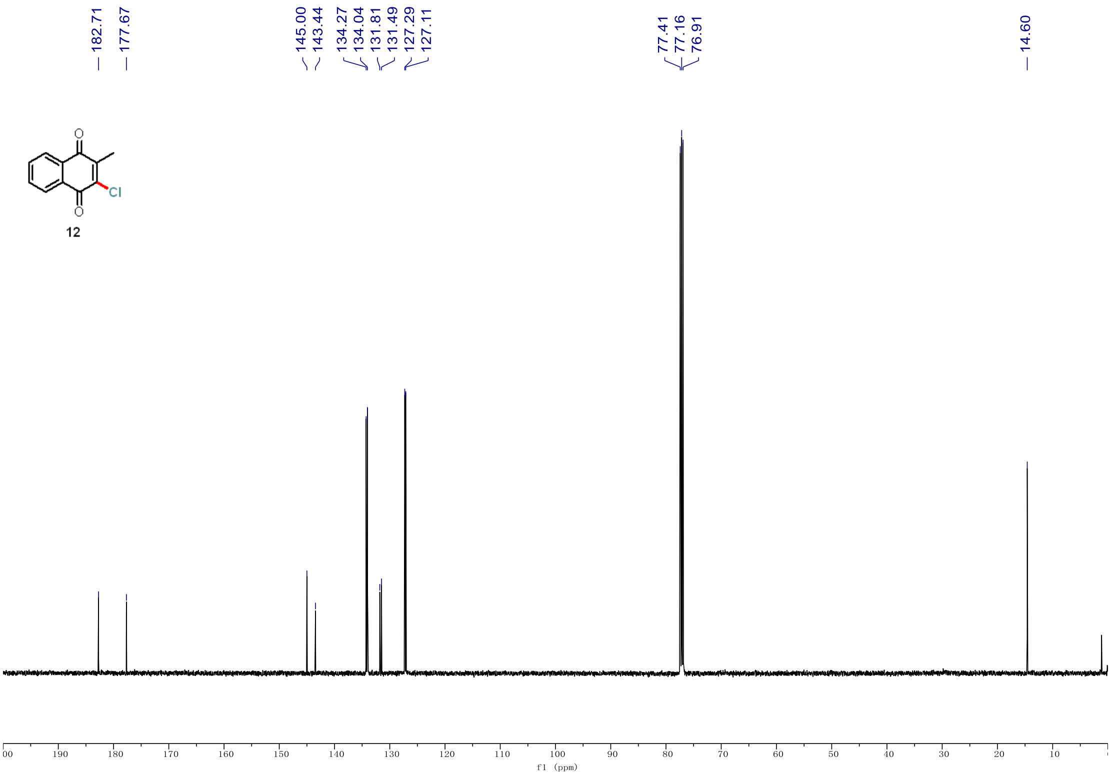

^{13}CNMR of compound **12**

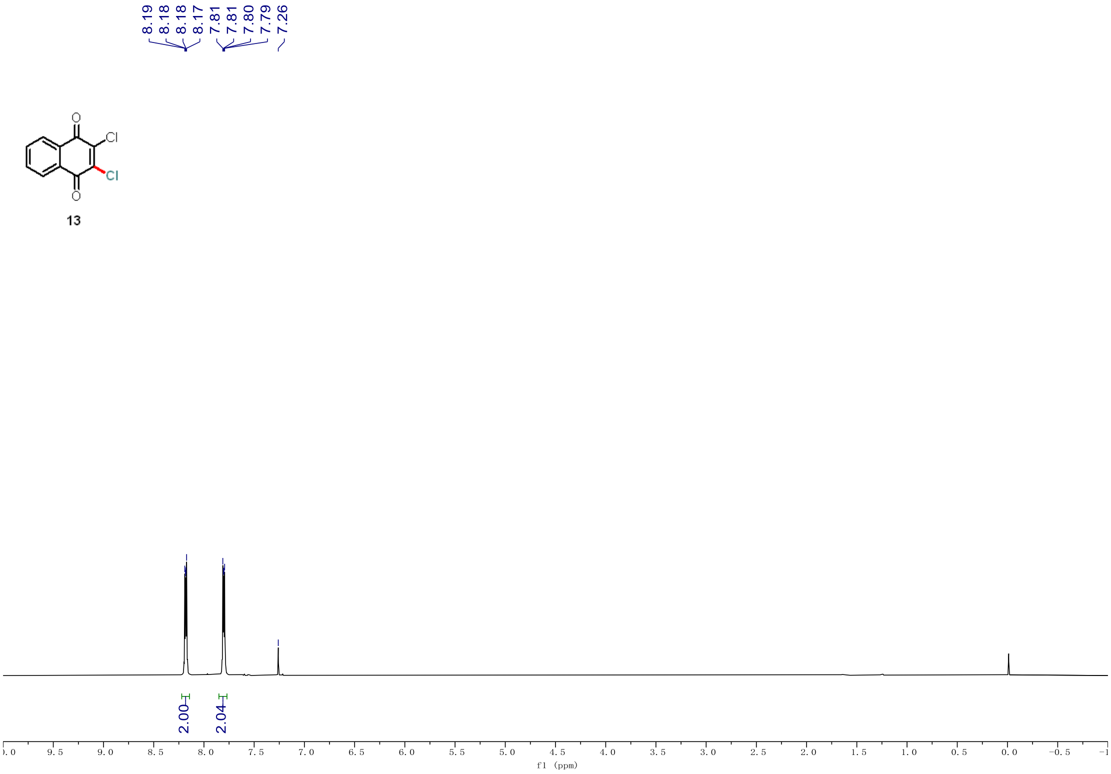

^{1}H NMR of compound **13**

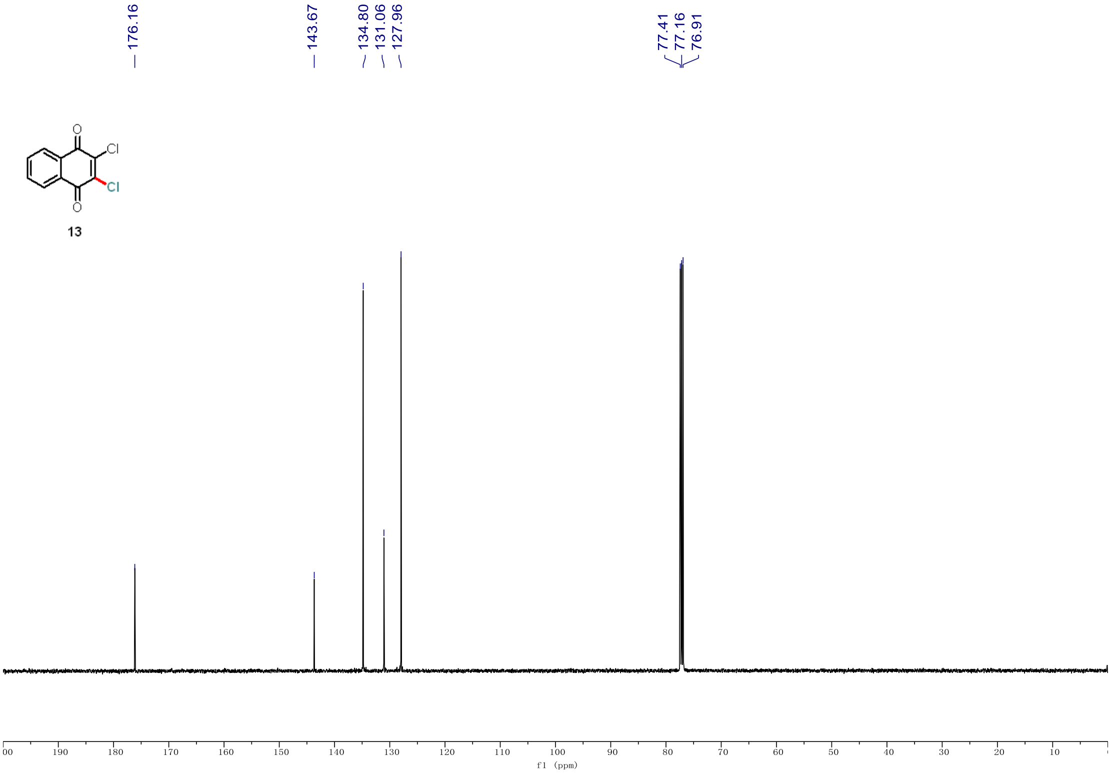

^{13}C NMR of compound **13**

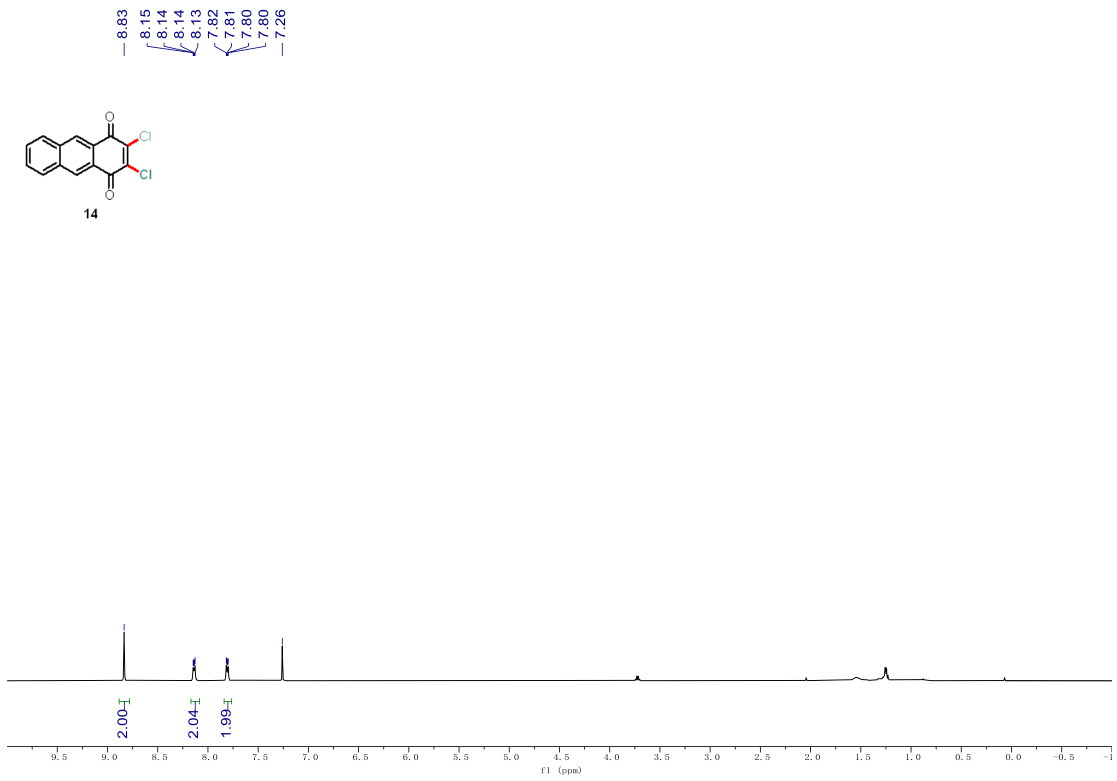

^1H NMR of compound **14**

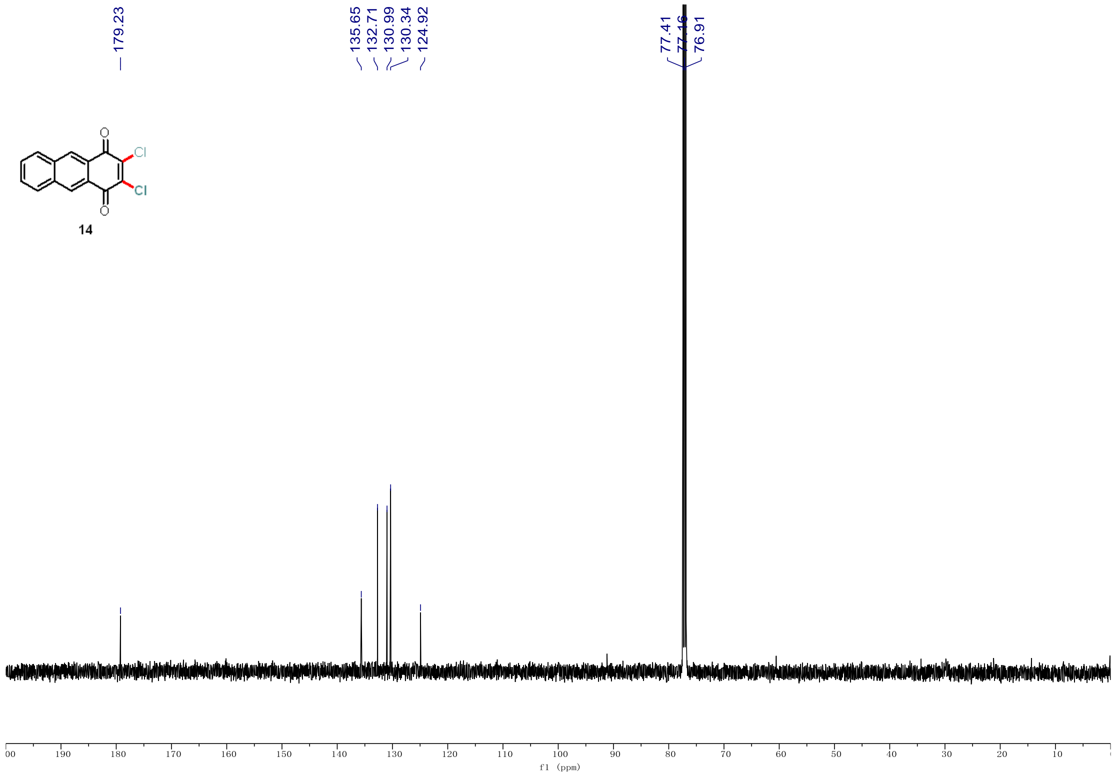

^{13}C NMR of compound **14**

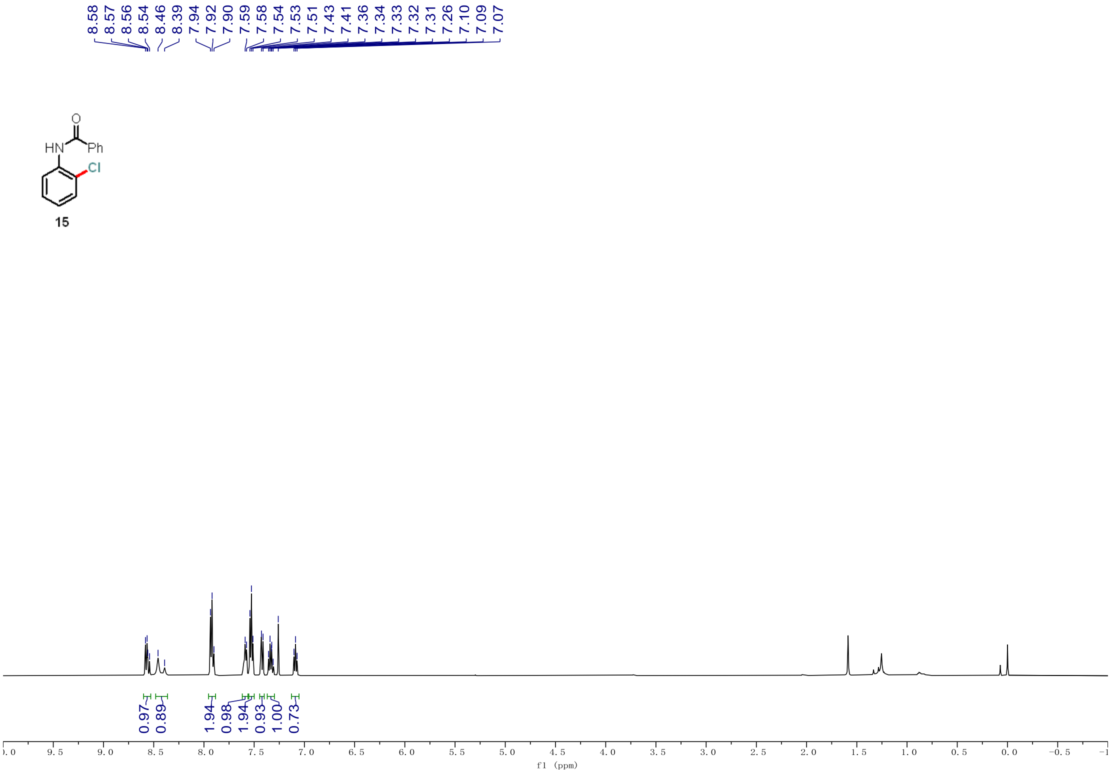

^{1}H NMR of compound **15**

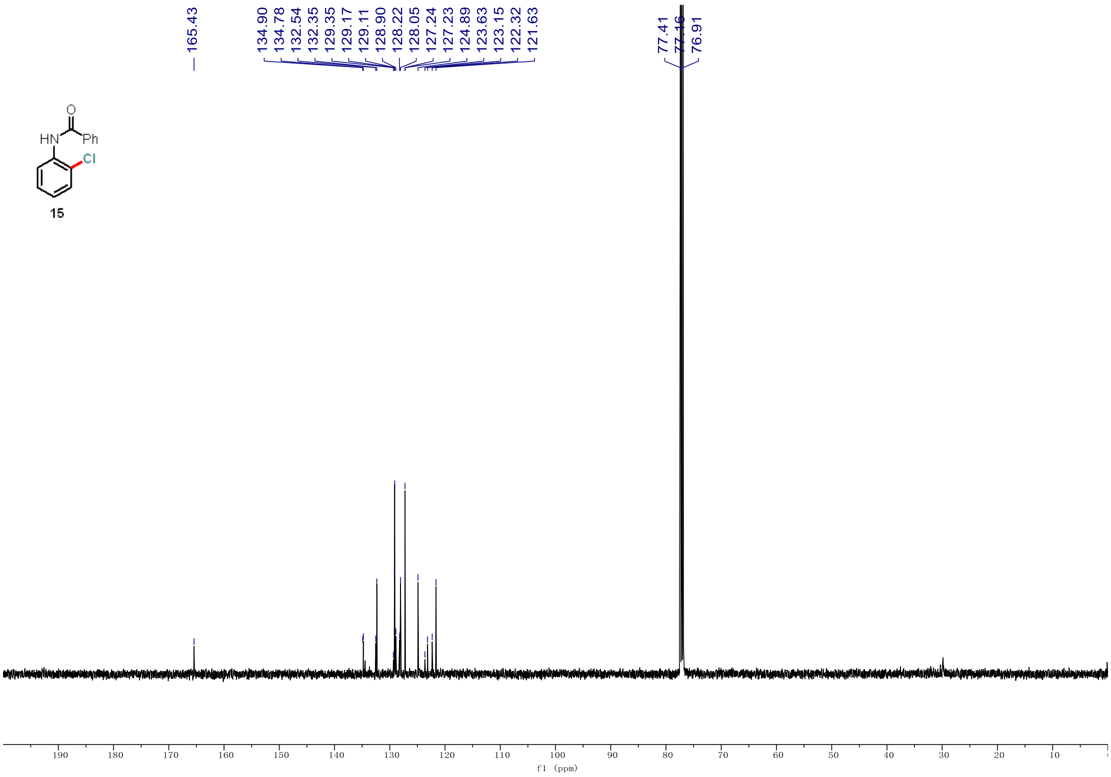

^{13}C NMR of compound **15**

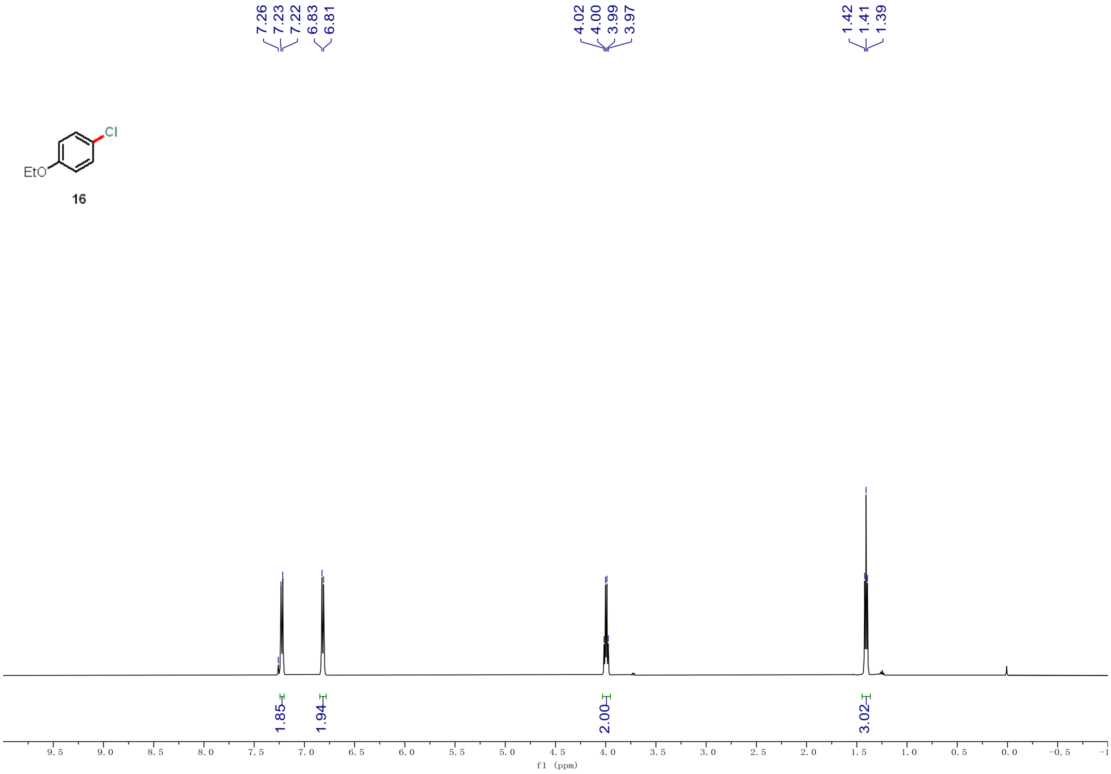

^{1}H NMR of compound **16**

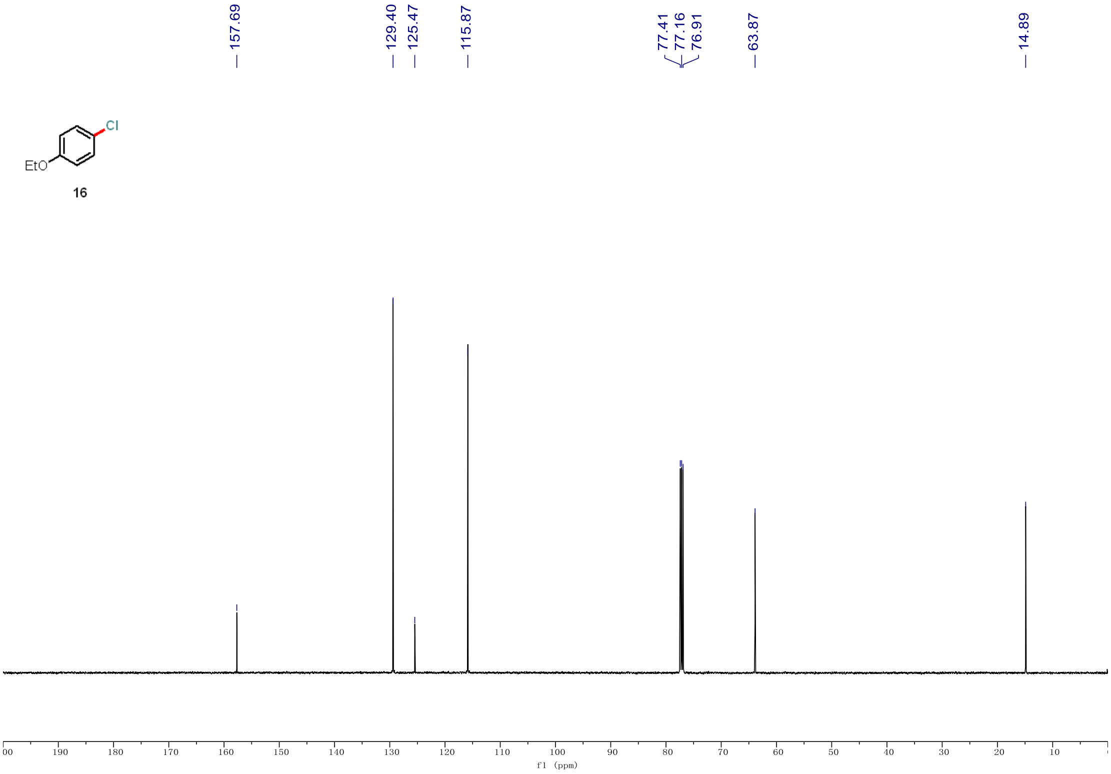

^{13}C NMR of compound **16**

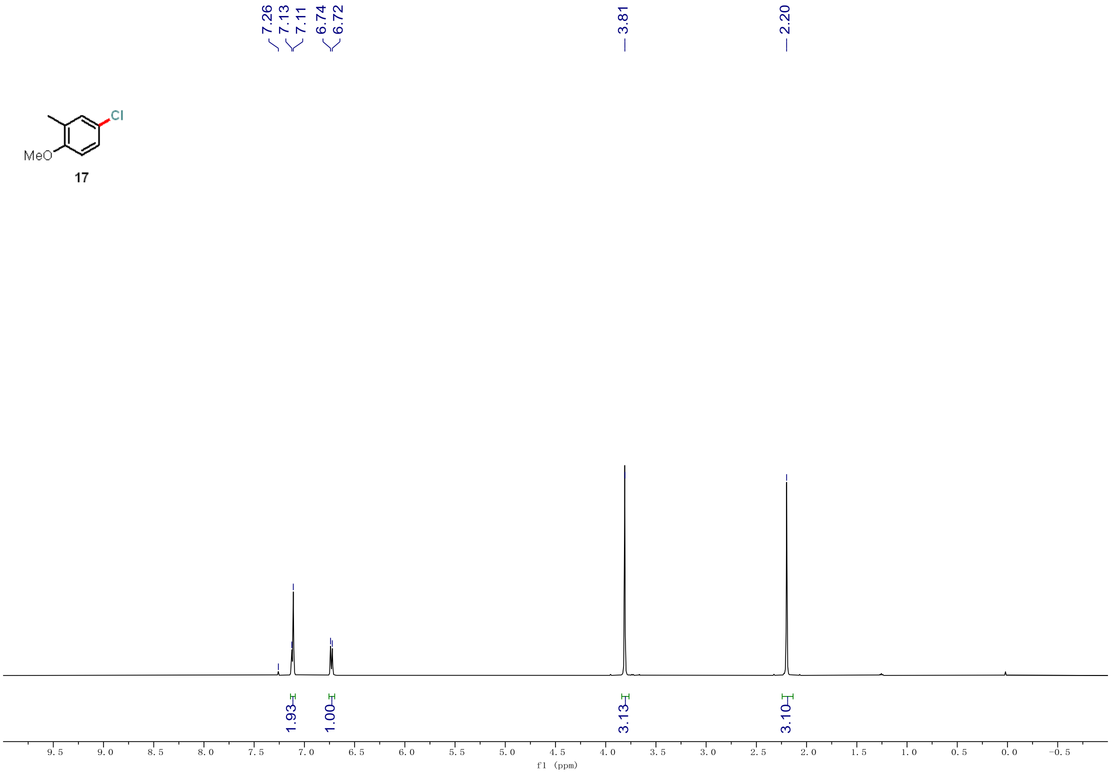

^{1}H NMR of compound **17**

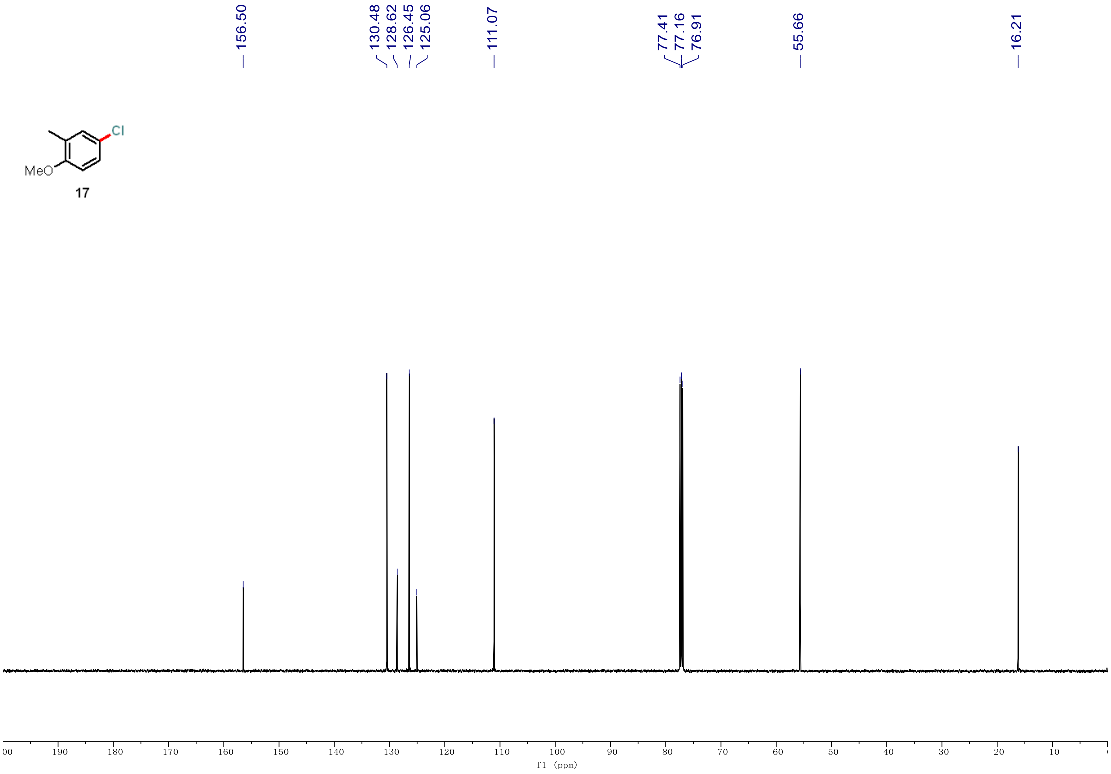

^{13}C NMR of compound **17**

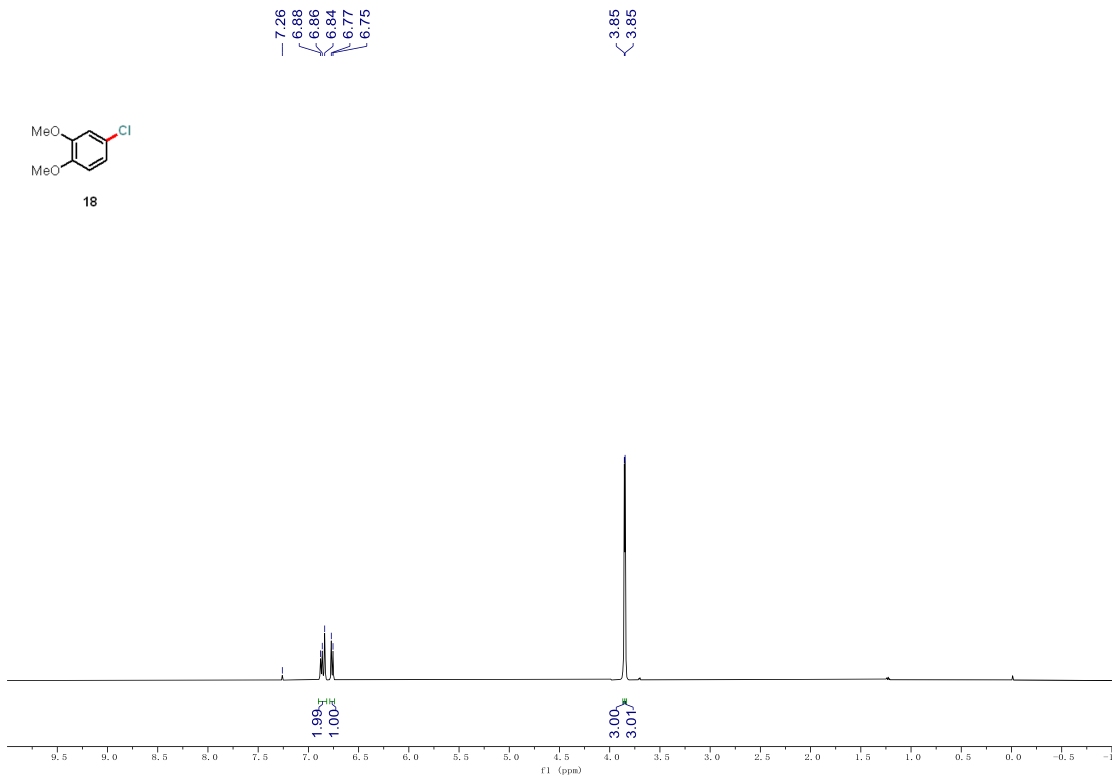

^{1}H NMR of compound **18**

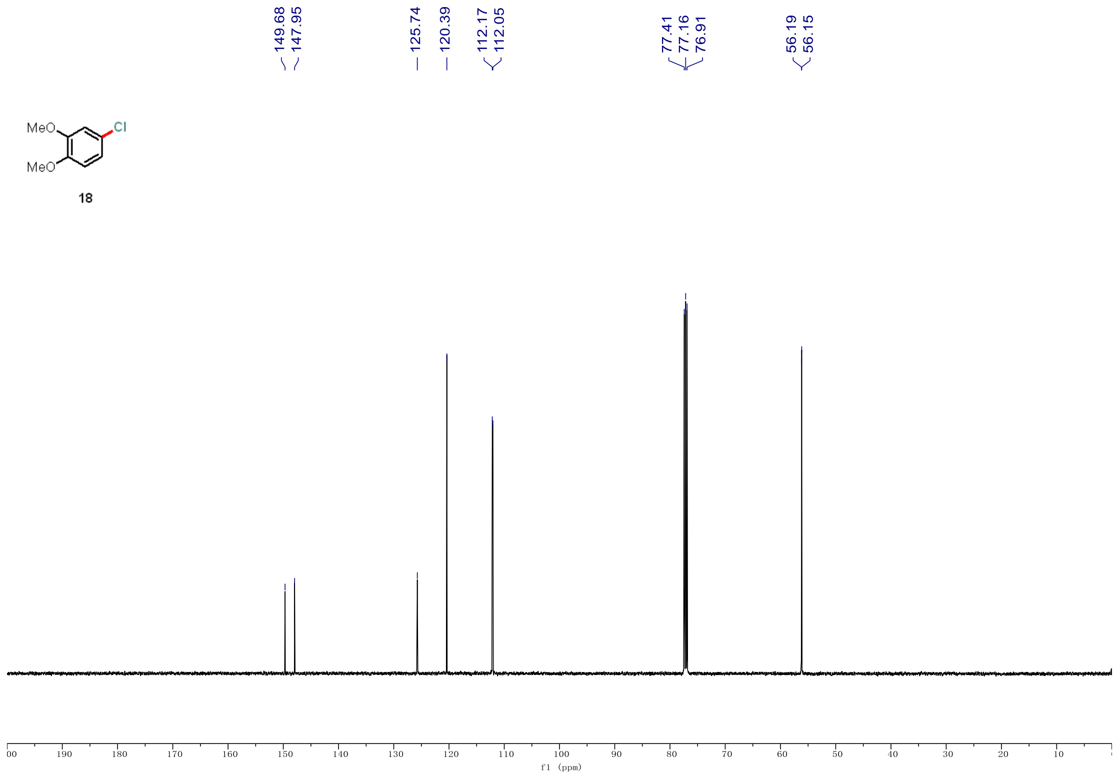

^{13}C NMR of compound **18**

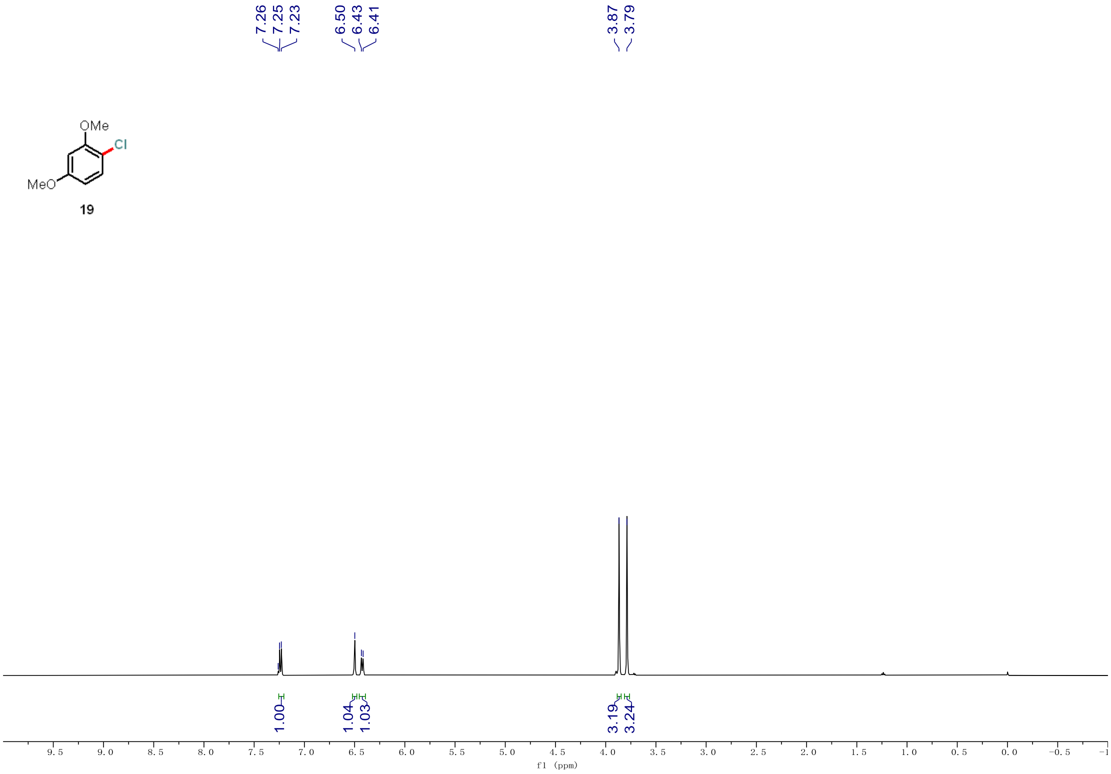

^{1}H NMR of compound **19**

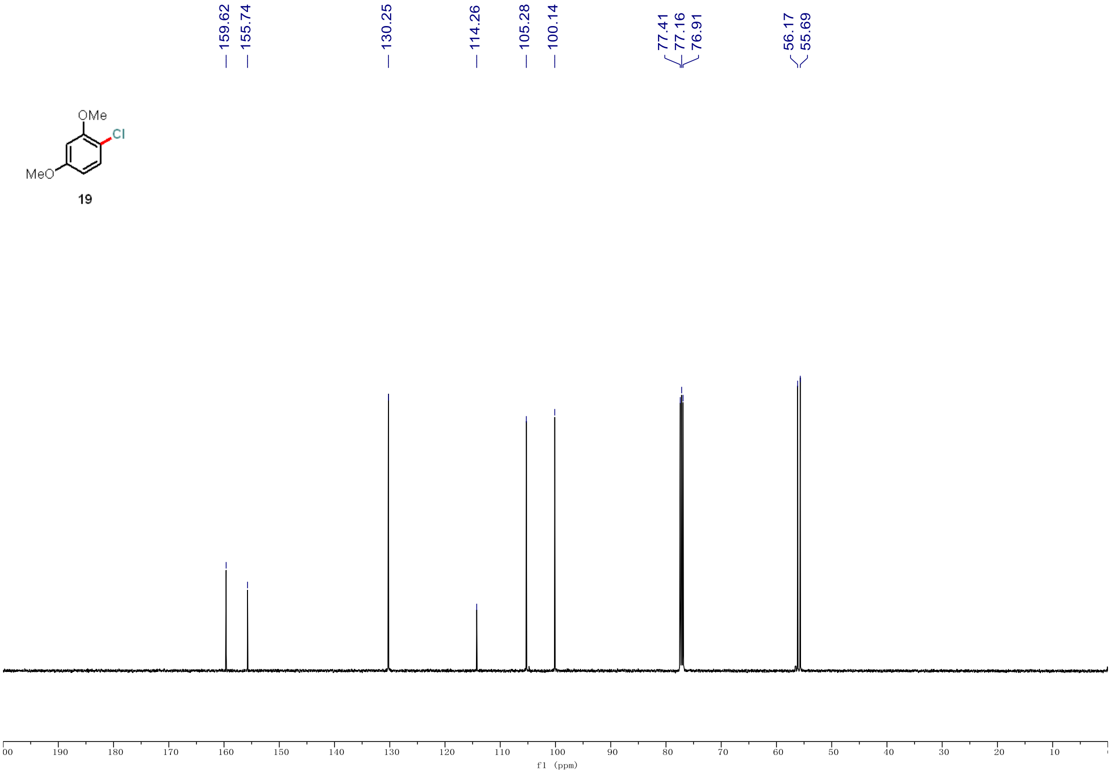

^{13}C NMR of compound **19**

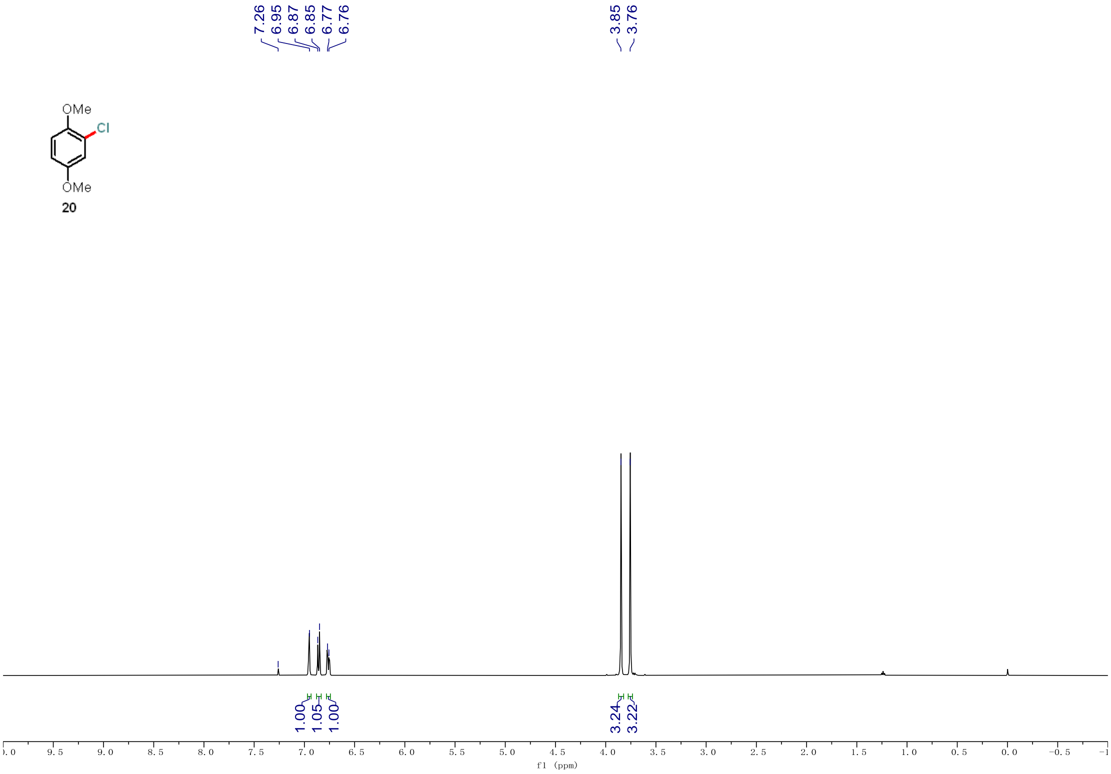

^{1}H NMR of compound **20**

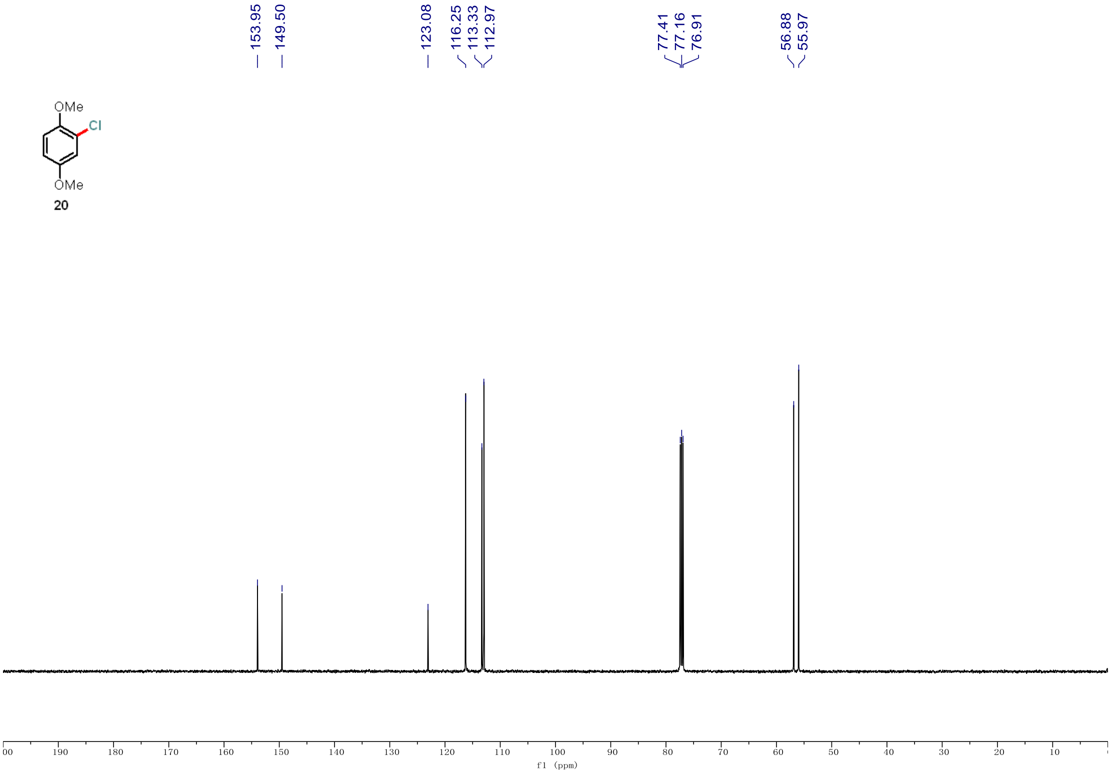

^{13}C NMR of compound **20**

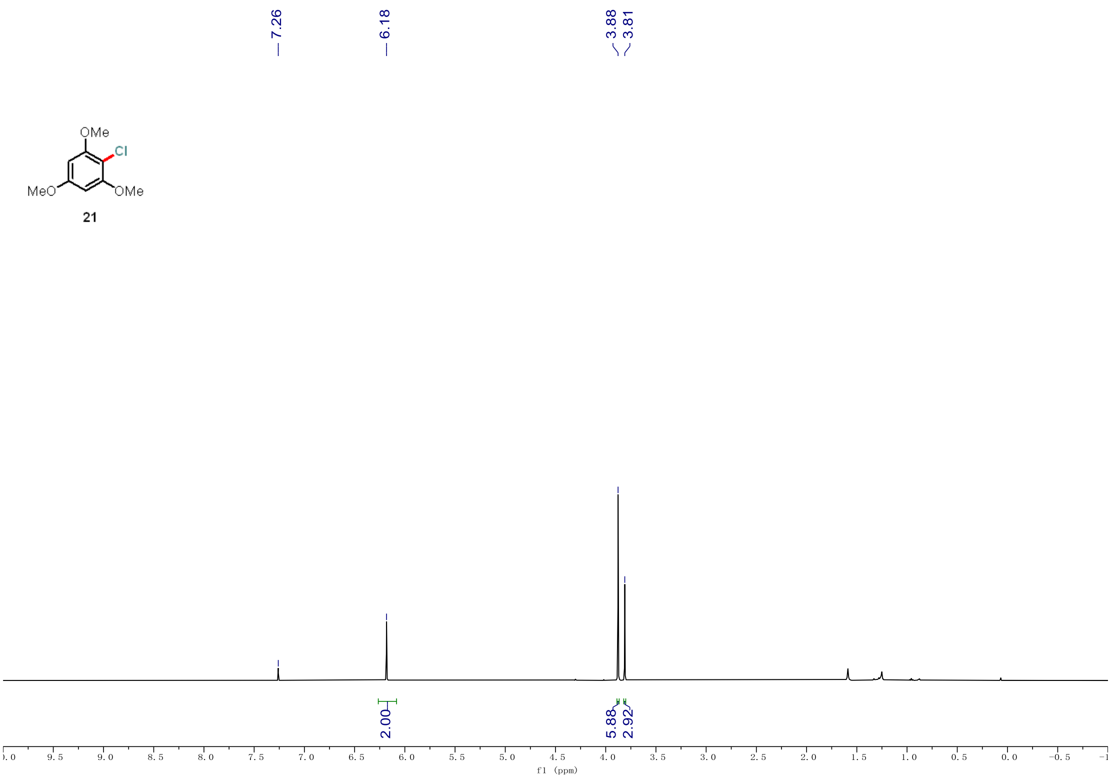

^{1}H NMR of compound **21**

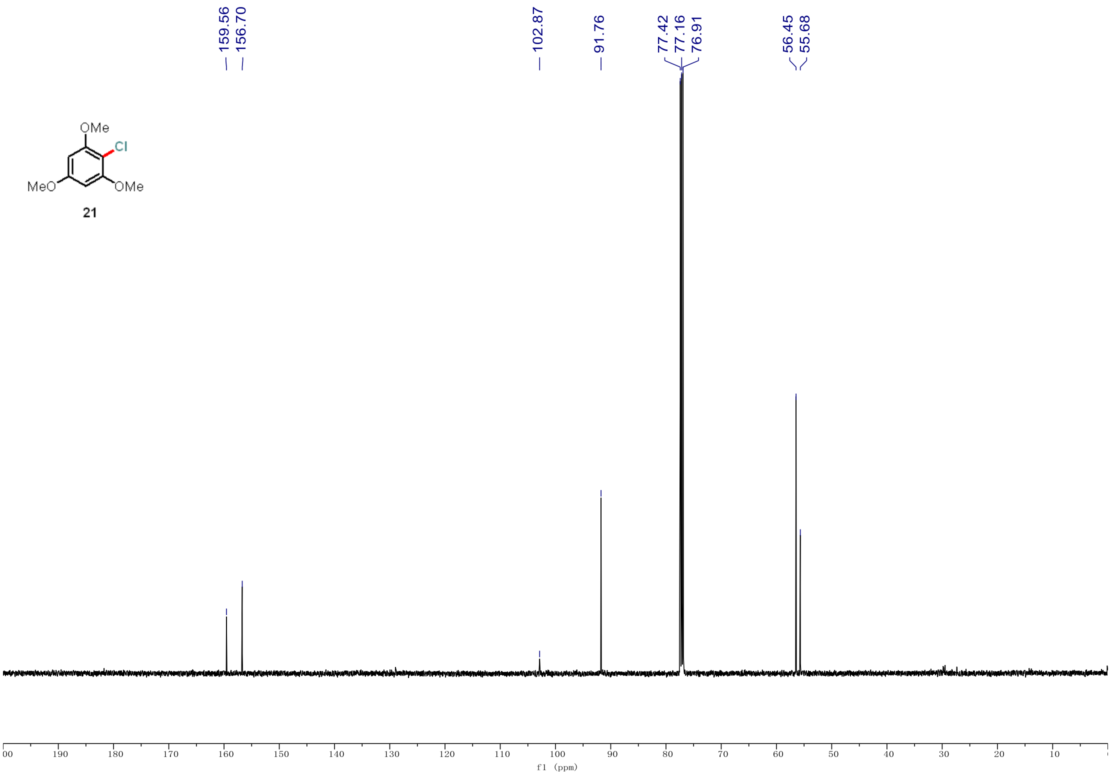

^{13}C NMR of compound **21**

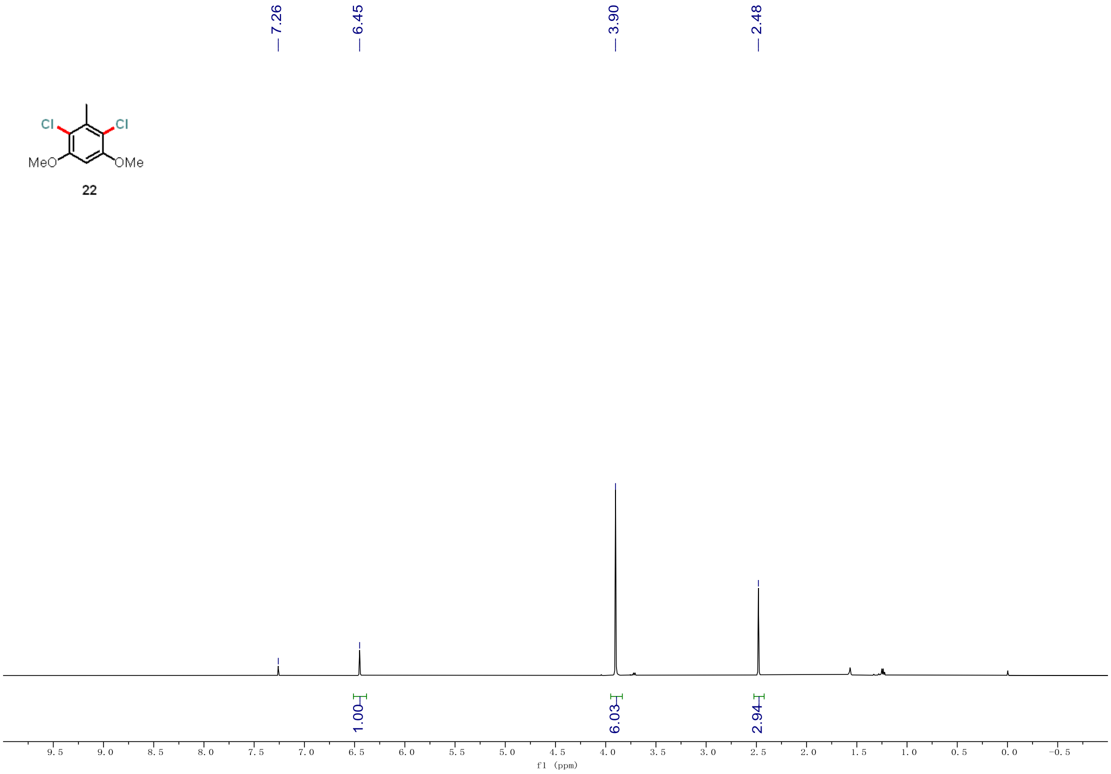

^{1}H NMR of compound **22**

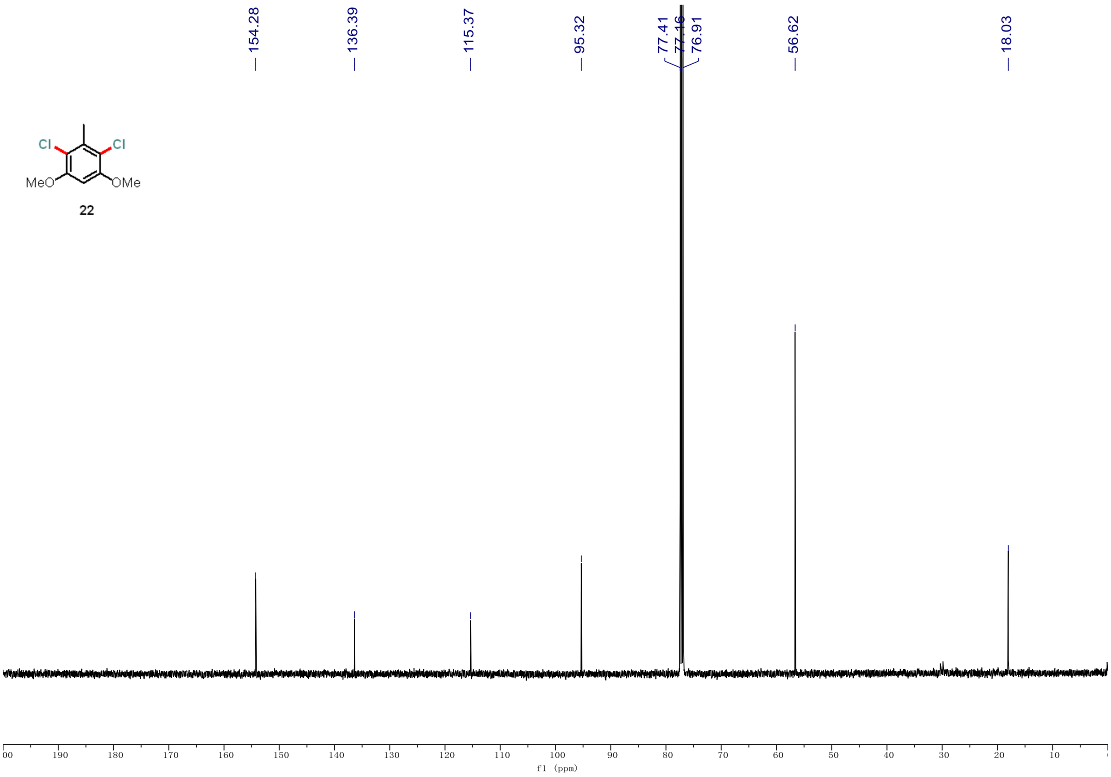

^{13}C NMR of compound **22**

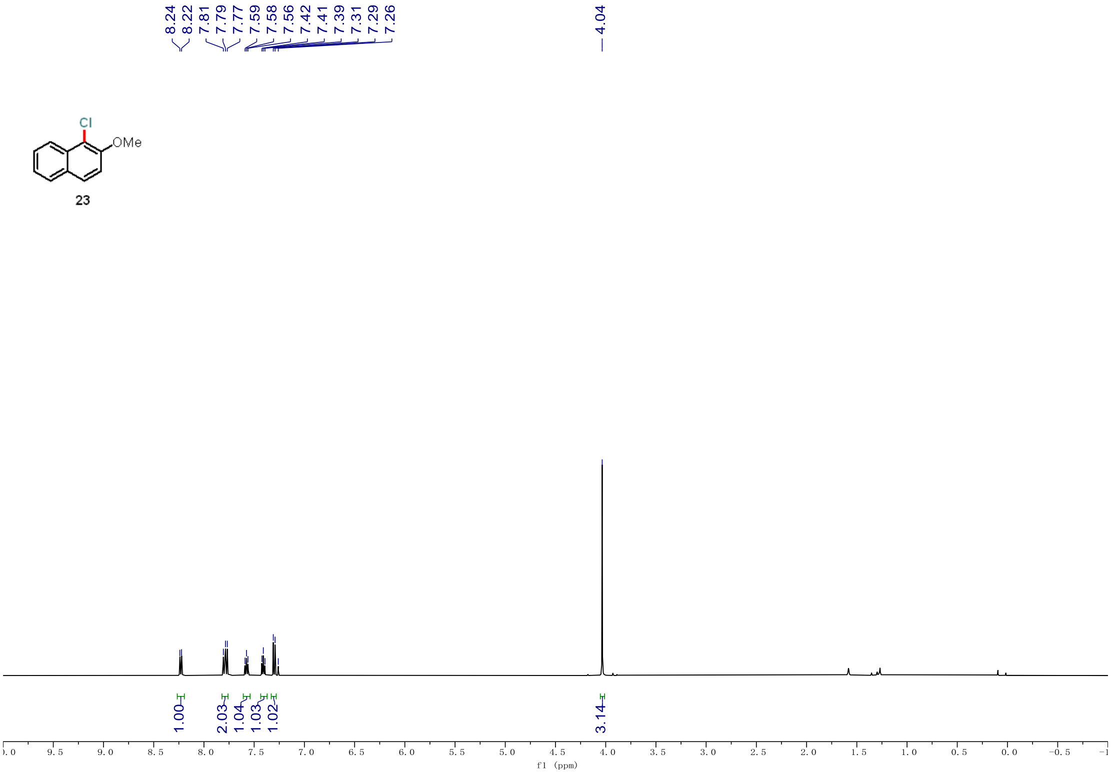

^{1}H NMR of compound **23**

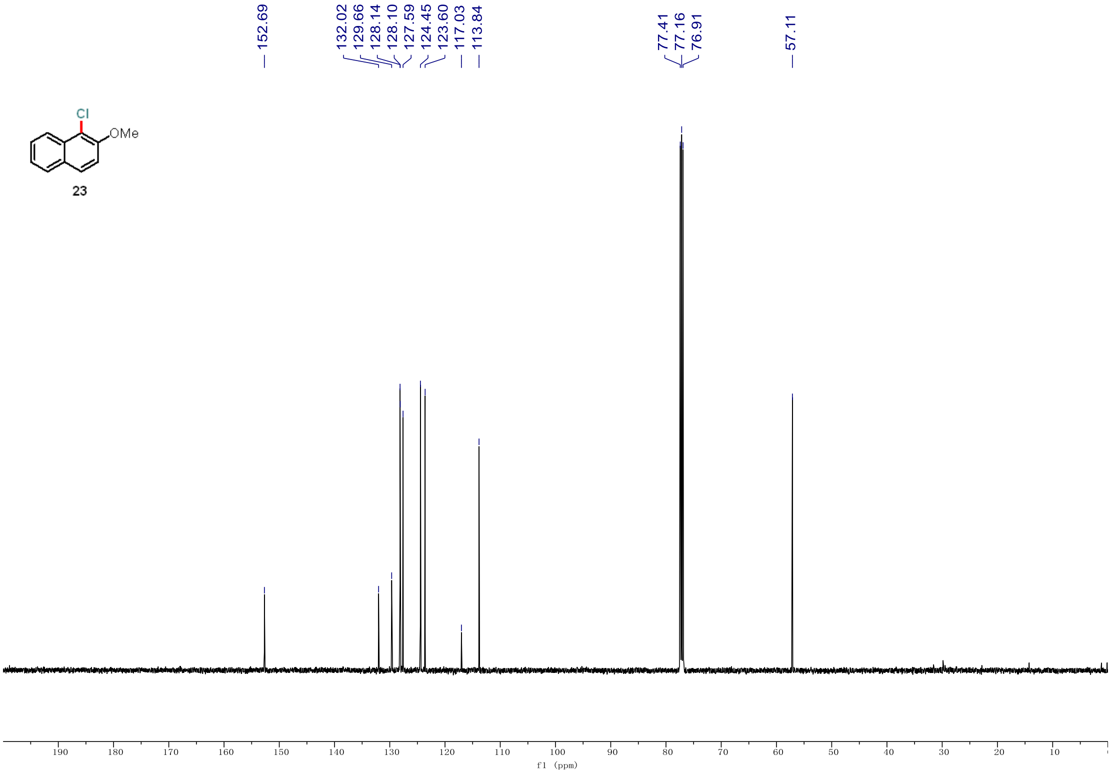

^{13}C NMR of compound **23**

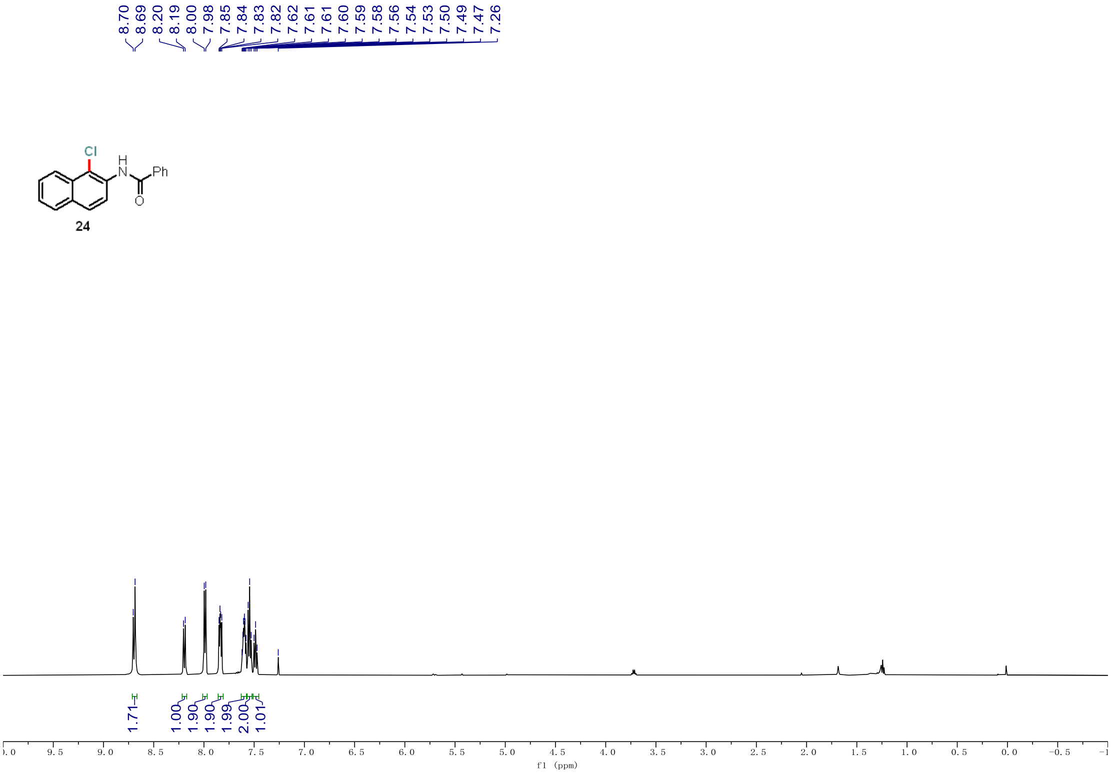

^{1}H NMR of compound **24**

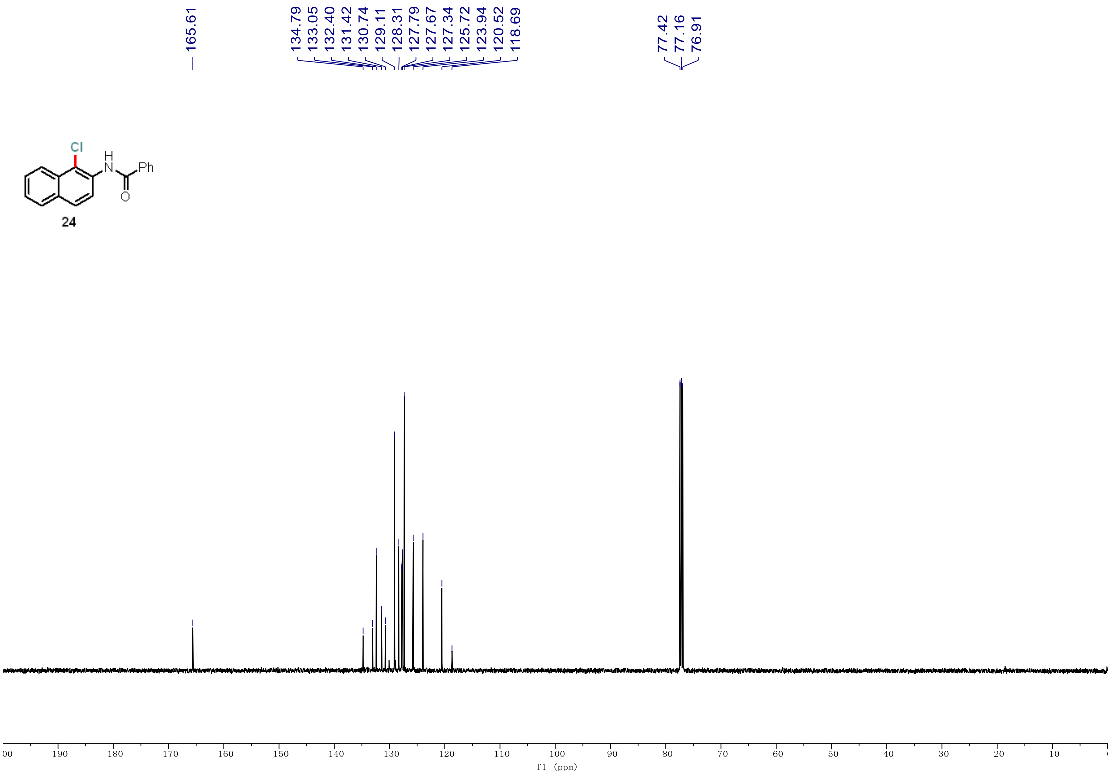

^{13}C NMR of compound **24**

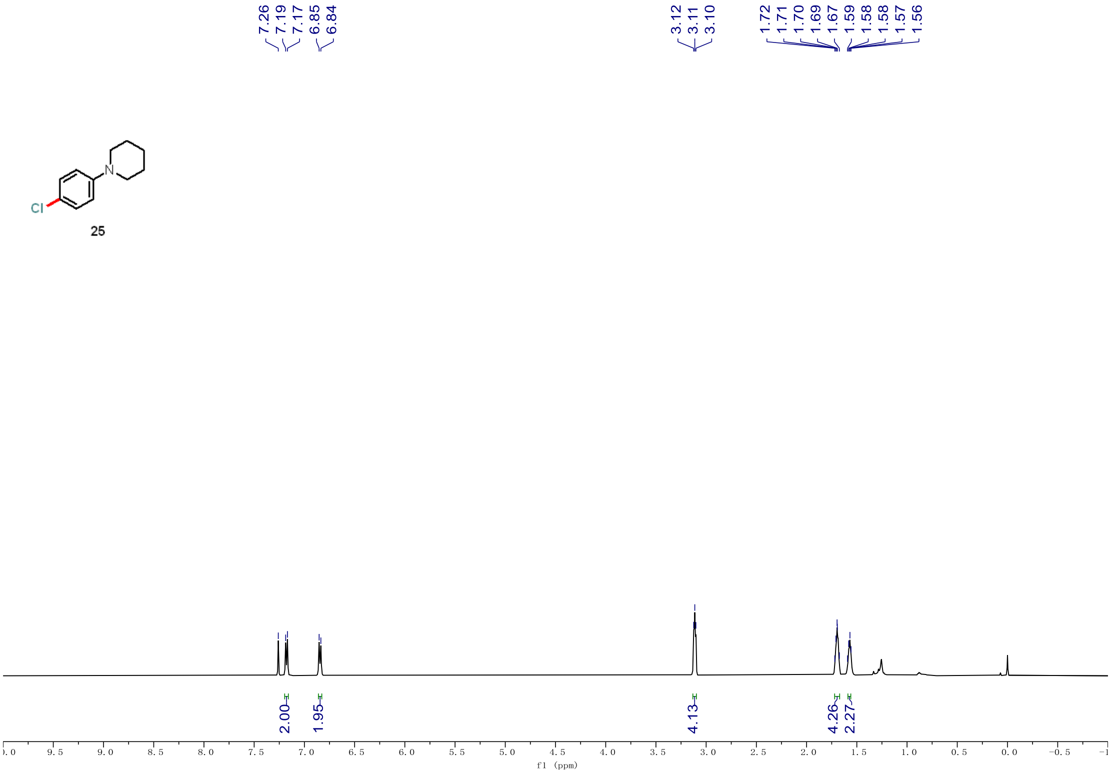

^{1}H NMR of compound **25**

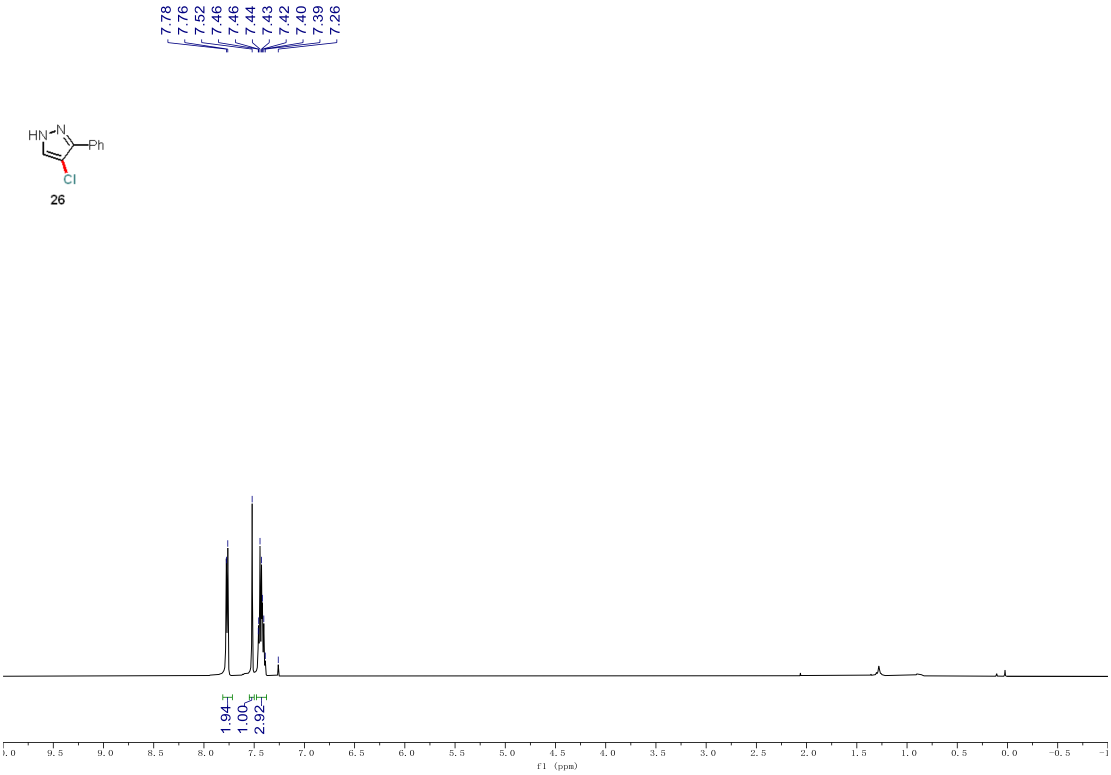

^{1}H NMR of compound **26**

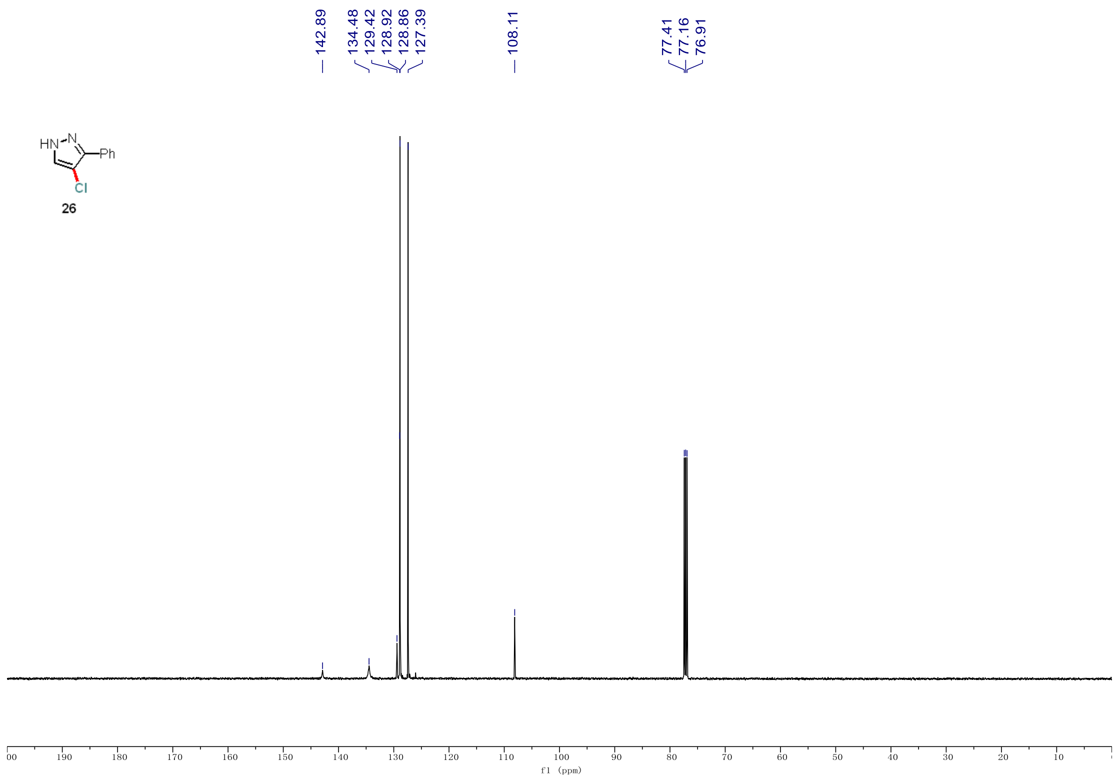

^{13}C NMR of compound **26**

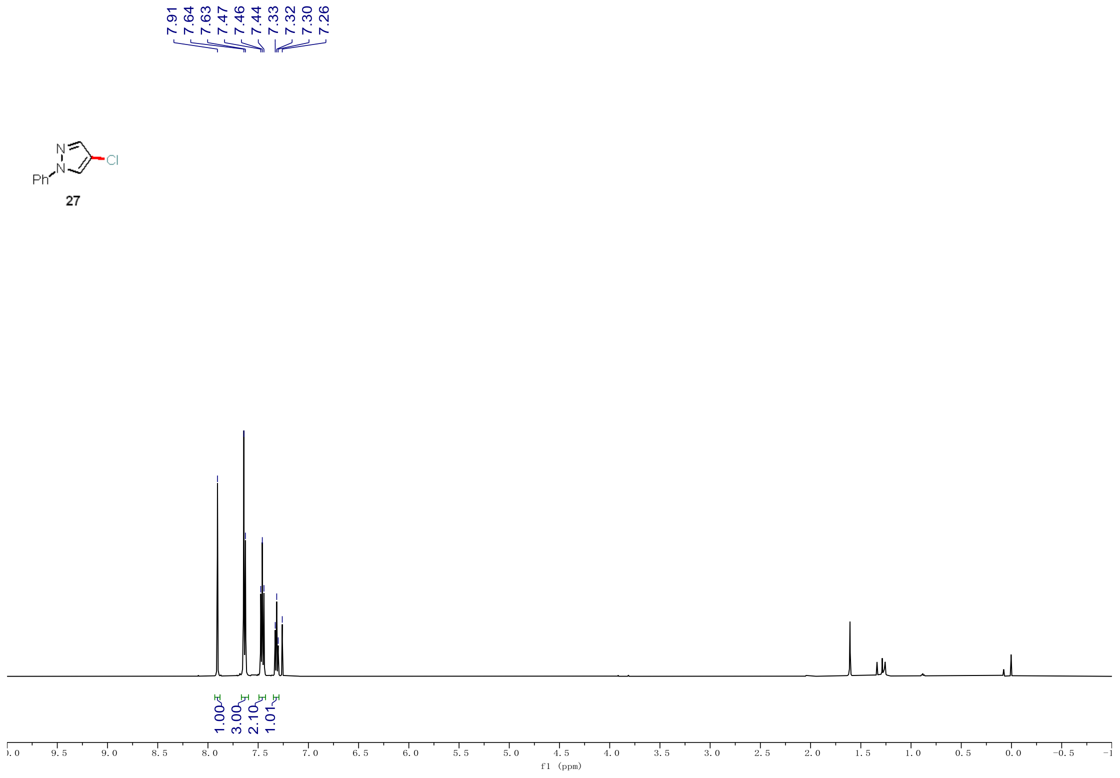

^{1}H NMR of compound **27**

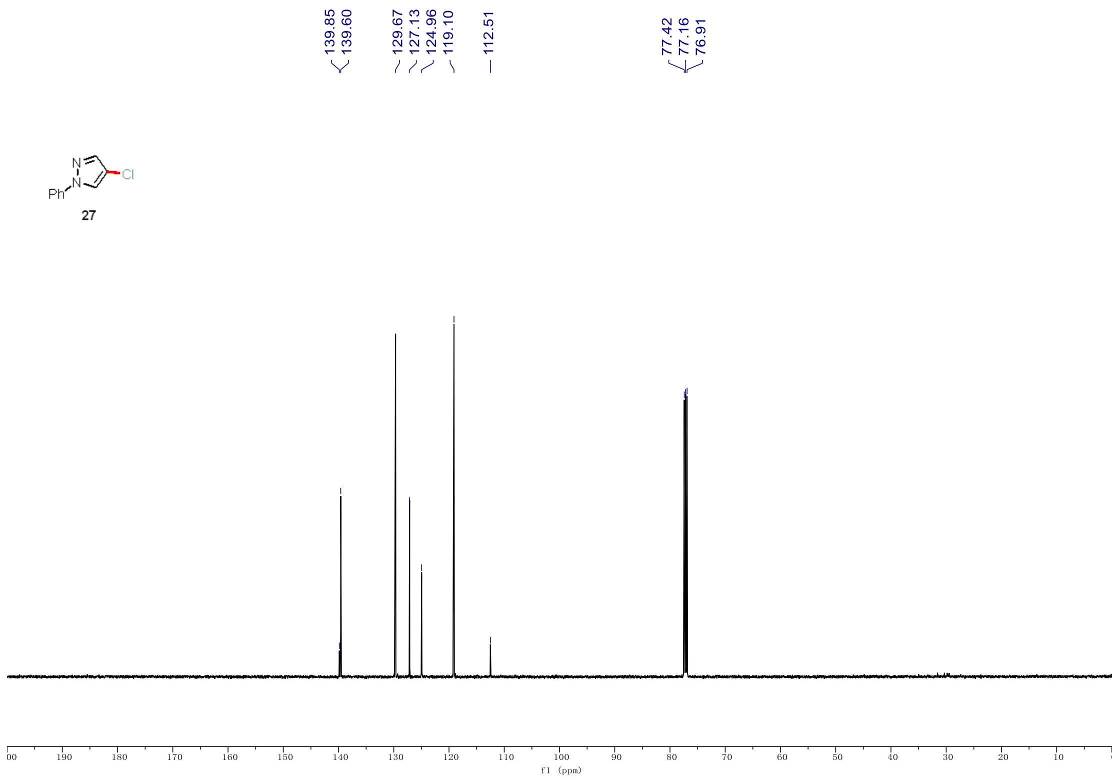

^{13}C NMR of compound **27**

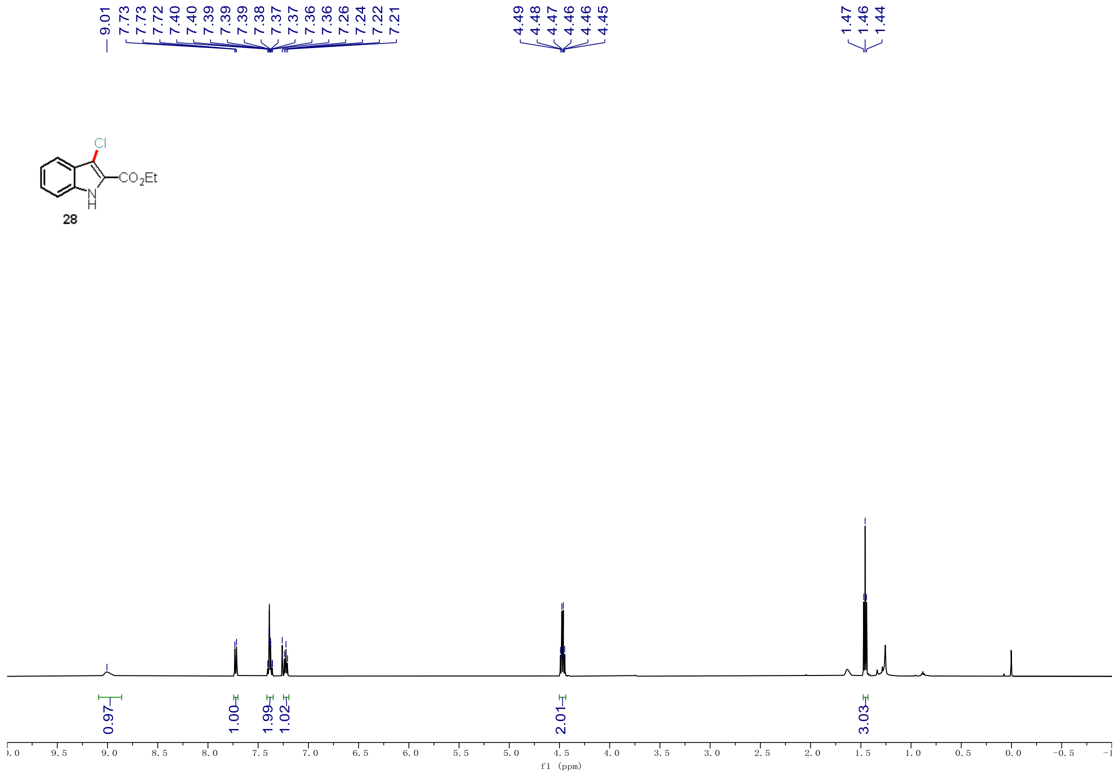

^{1}H NMR of compound **28**

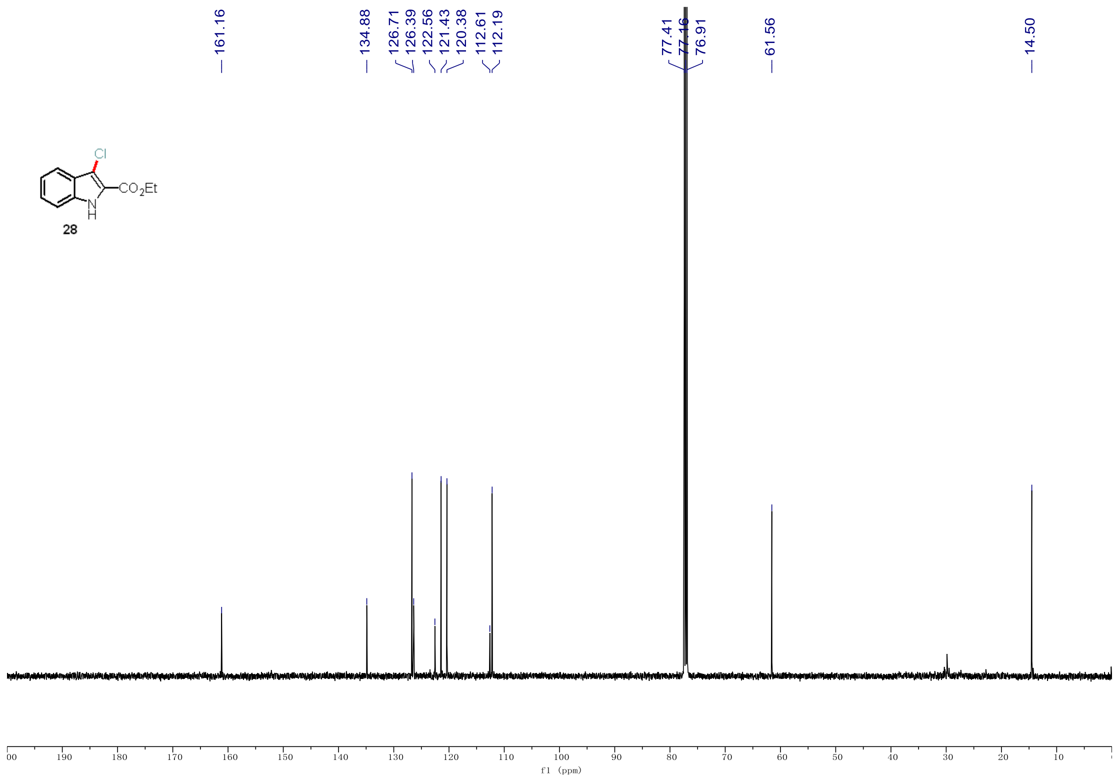

^{13}C NMR of compound **28**

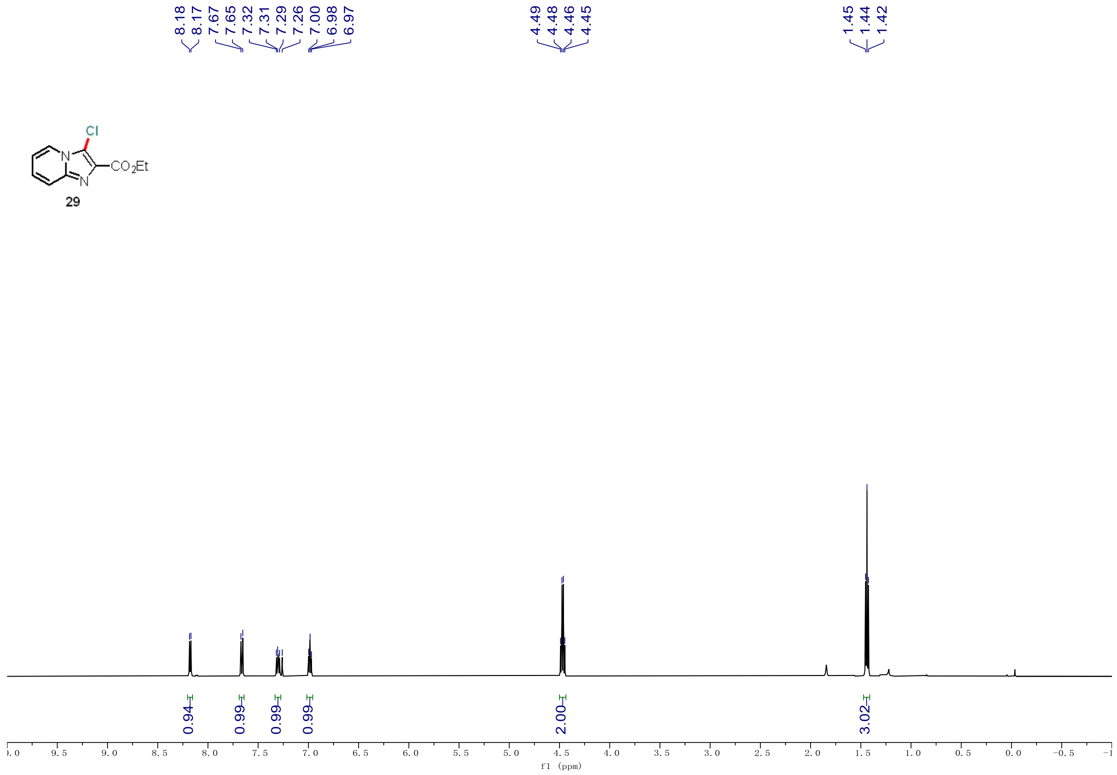

^{1}H NMR of compound **29**

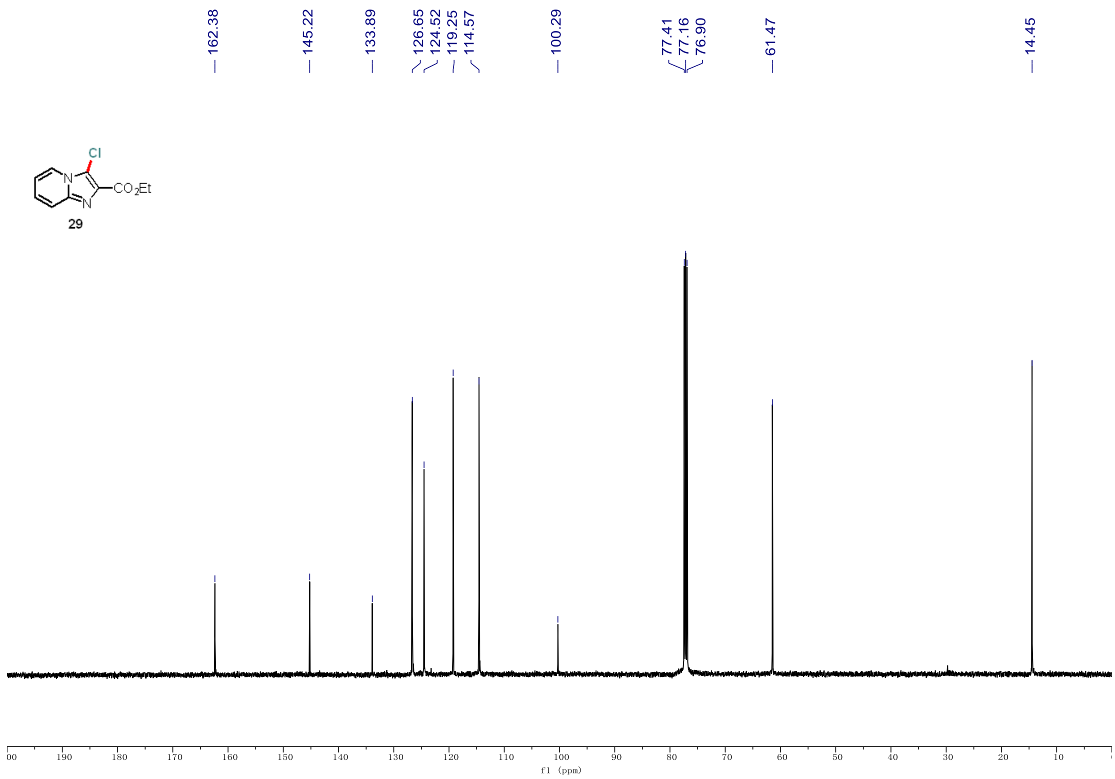

^{13}C NMR of compound **29**

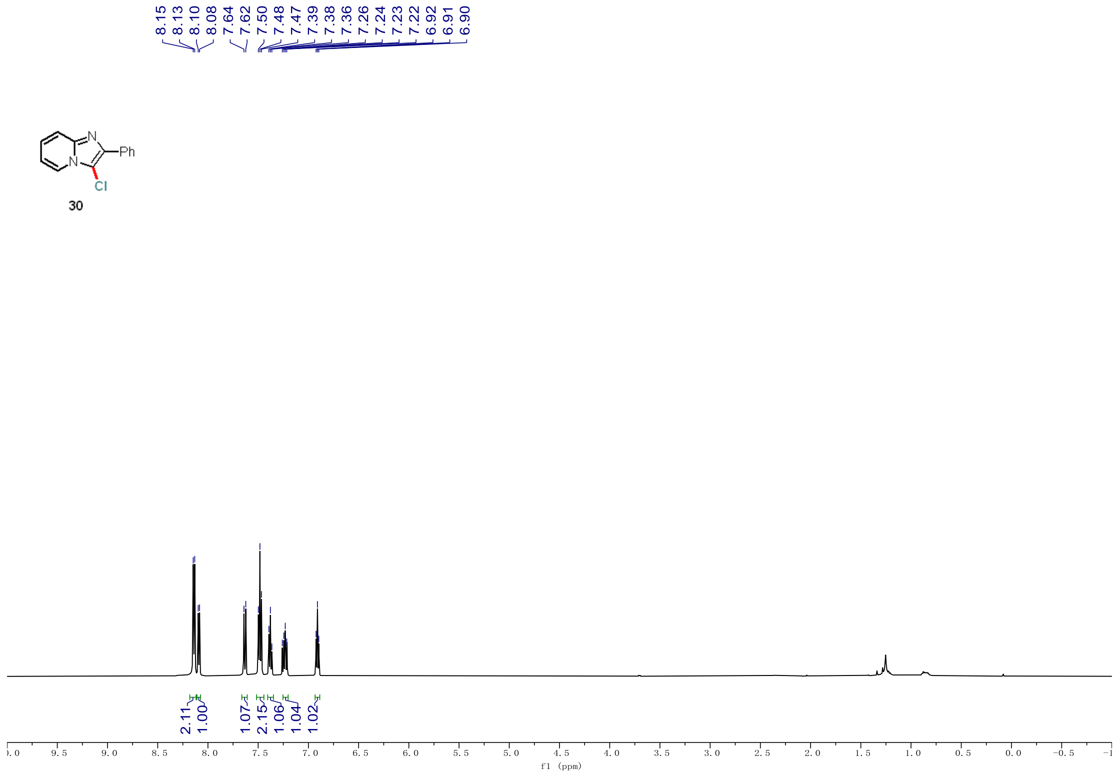

^{1}H NMR of compound **30**

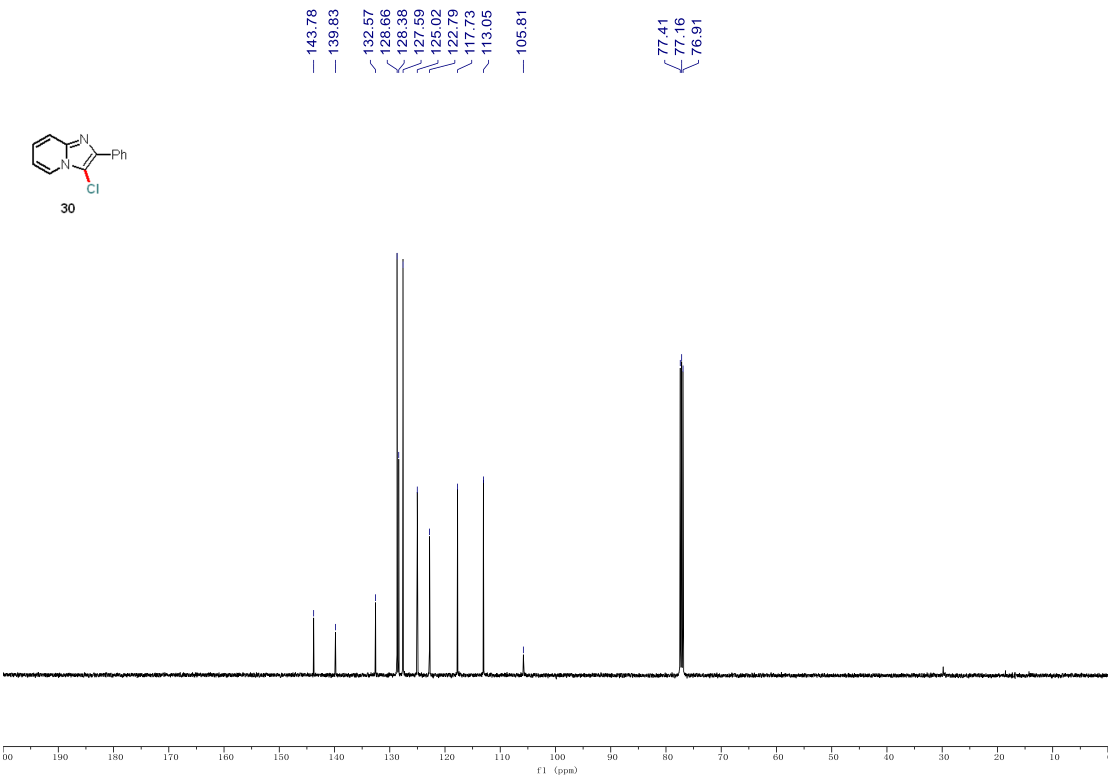

^{13}C NMR of compound **30**

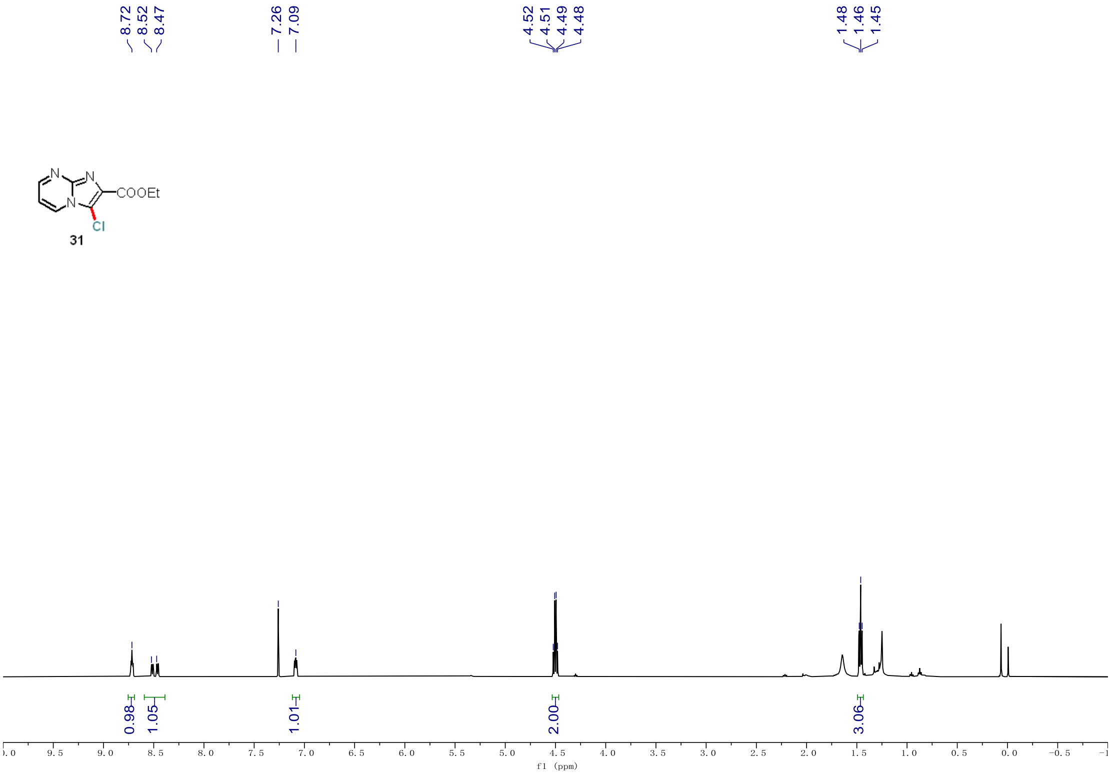

^{1}H NMR of compound **31**

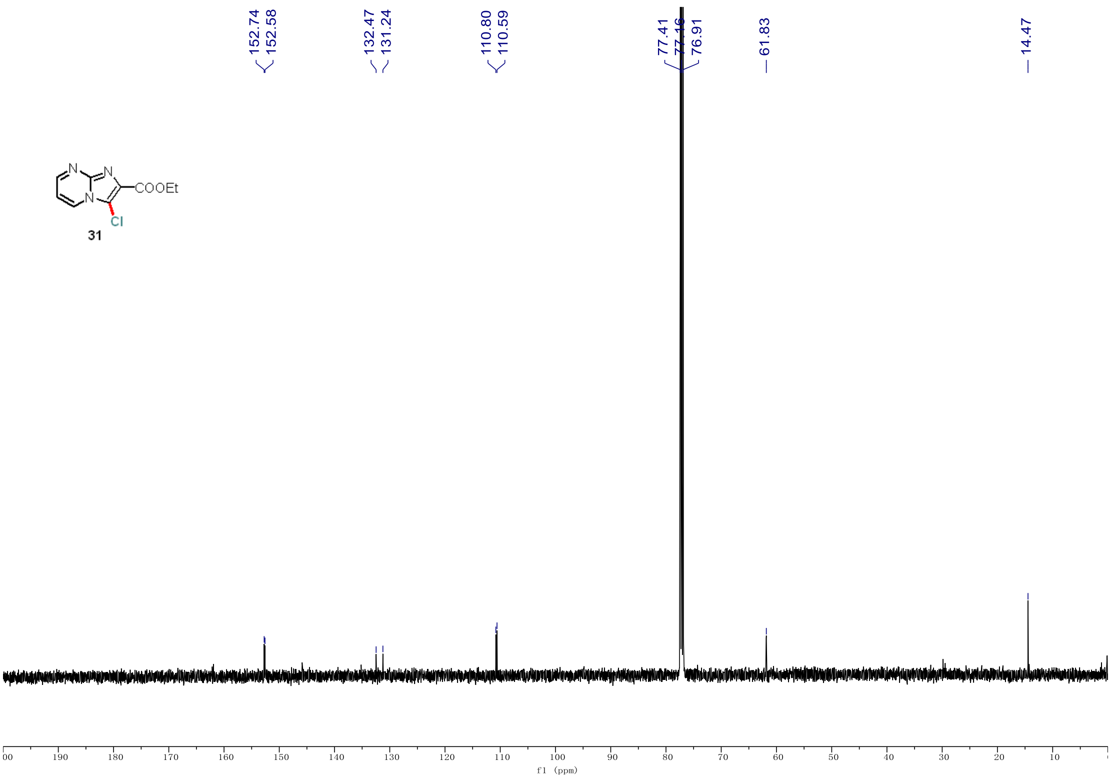

^{13}C NMR of compound **31**

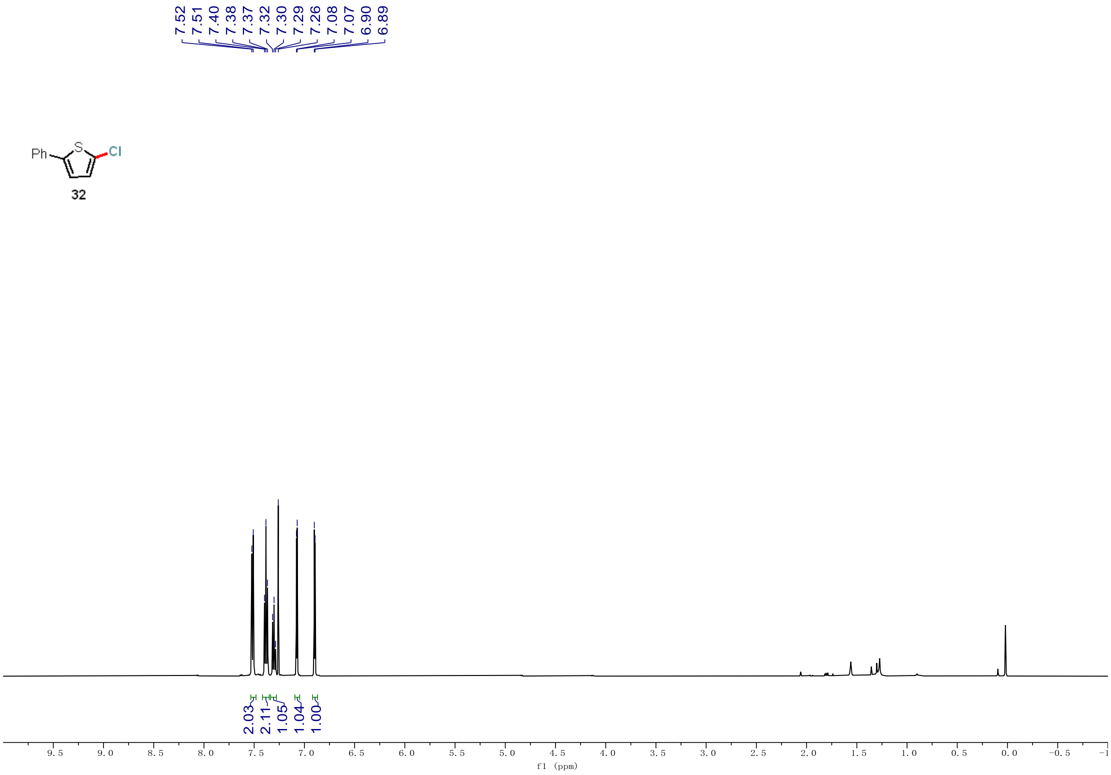

^{1}H NMR of compound **32**

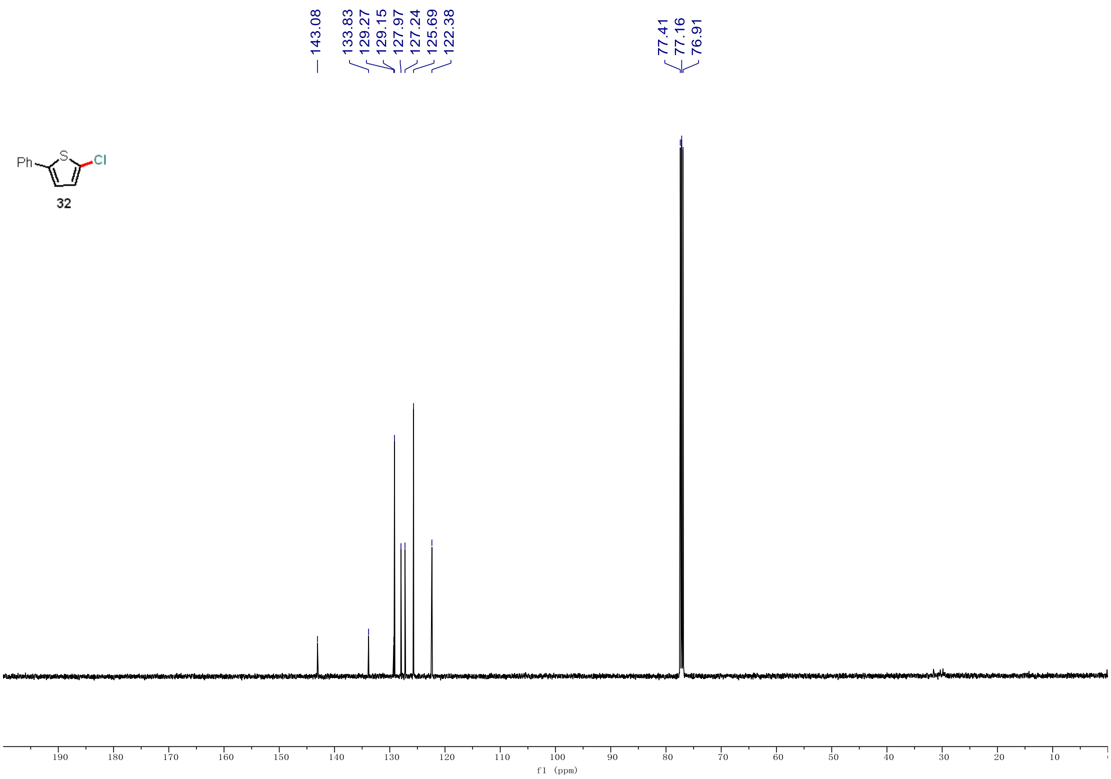

^{13}C NMR of compound **32**

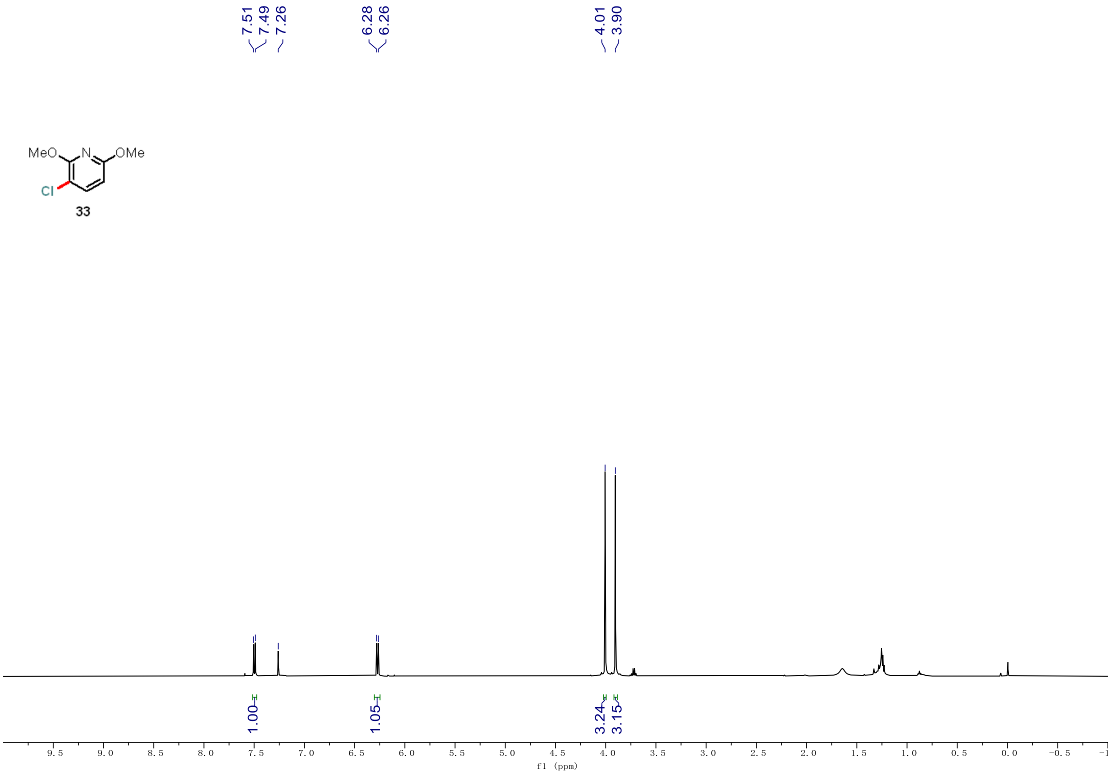

^{1}H NMR of compound **33**

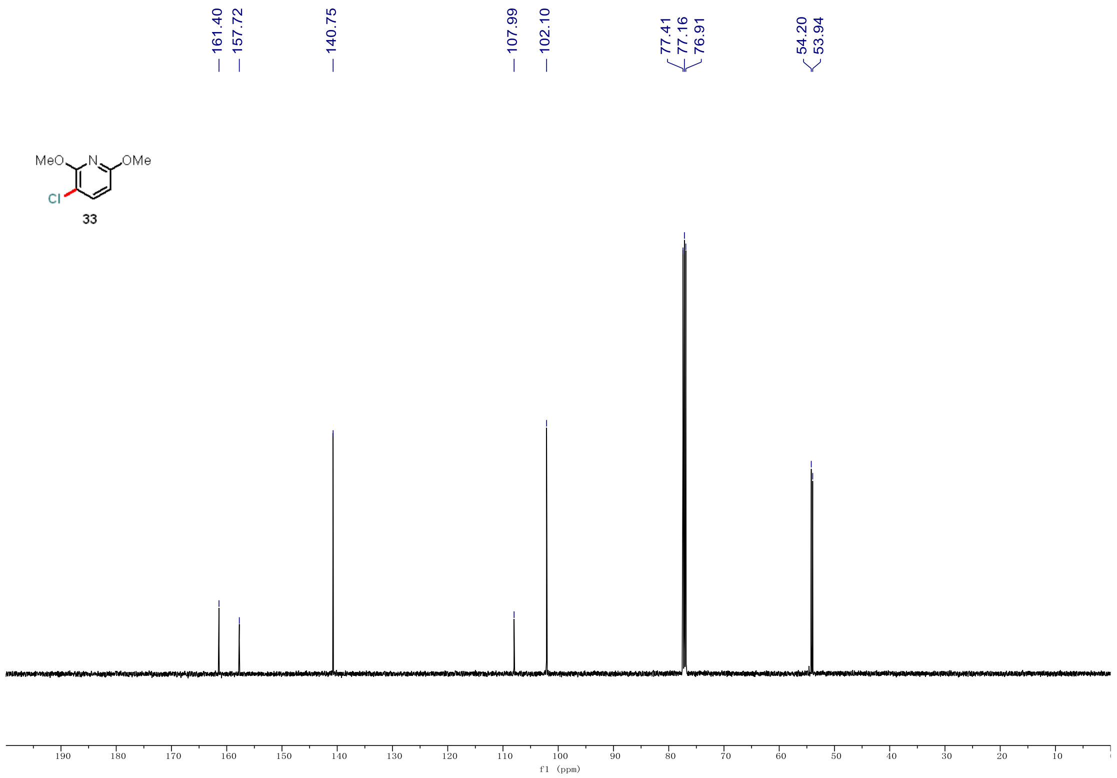

^{13}C NMR of compound **33**

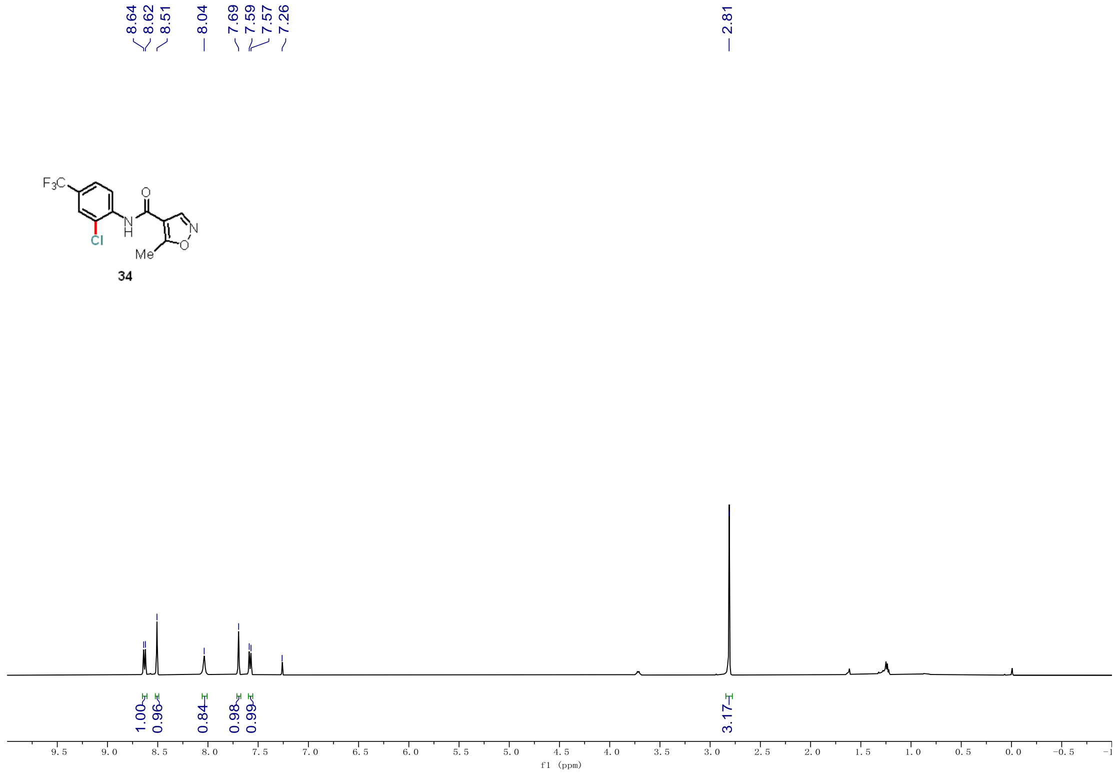

^{1}H NMR of compound **34**

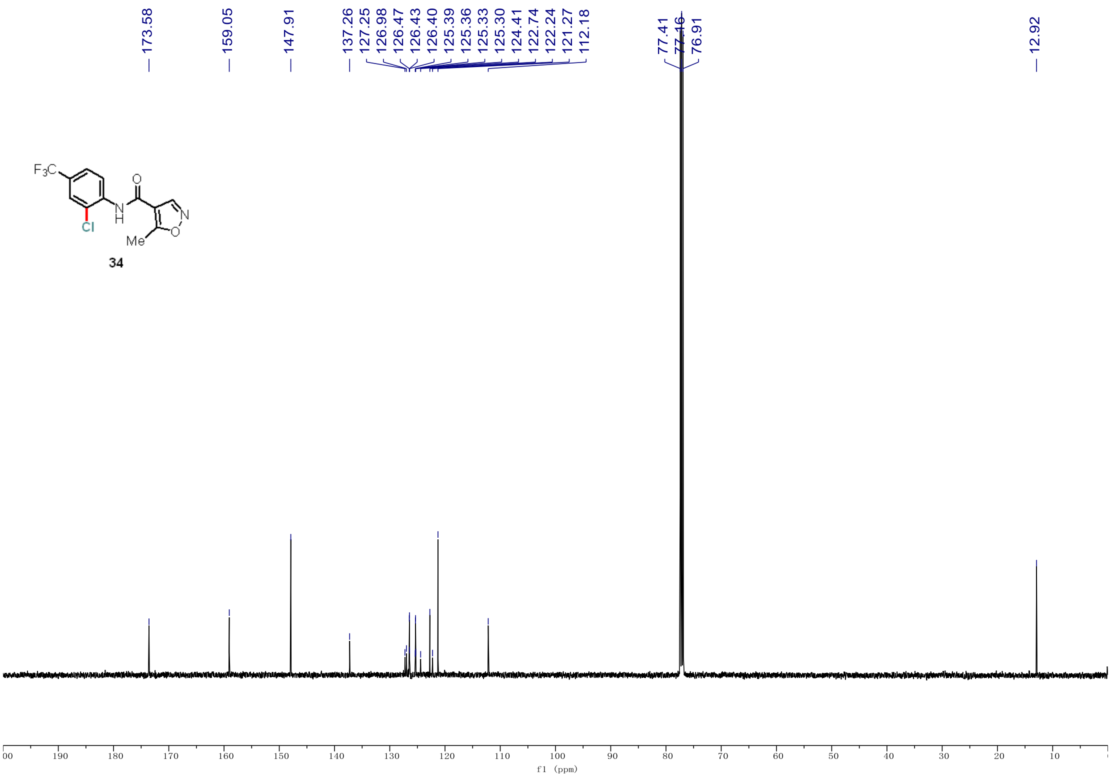

^{13}C NMR of compound **34**

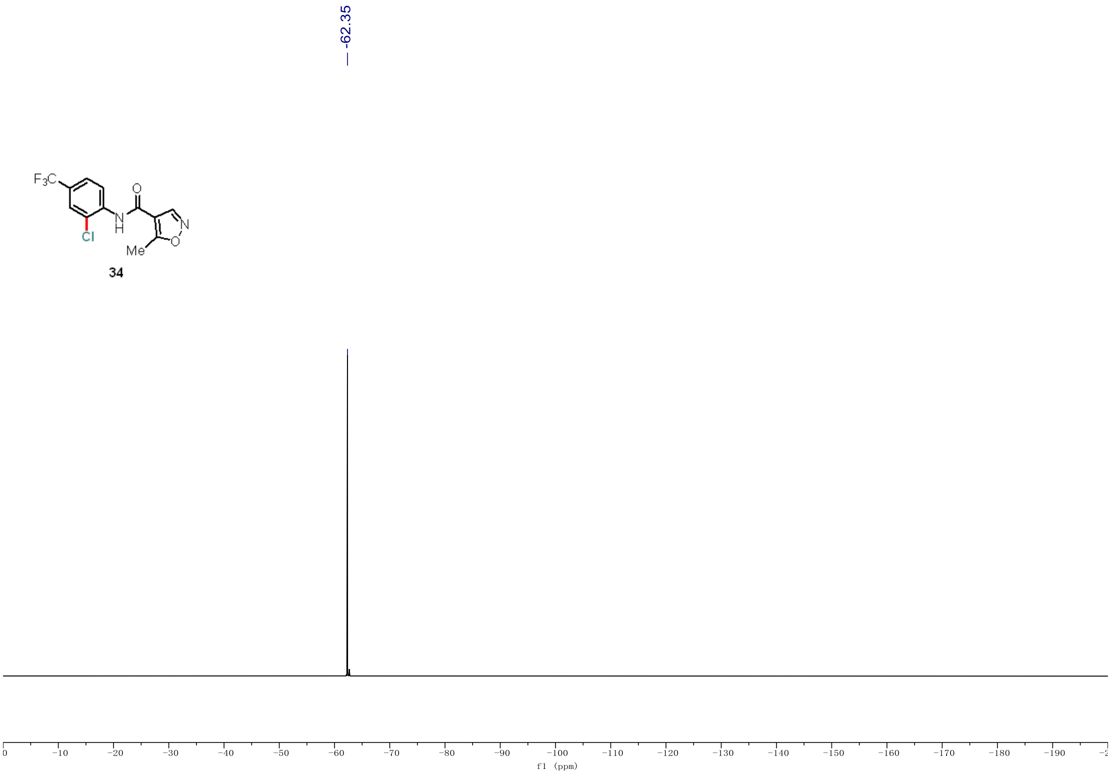

^{19}F NMR of compound **34**

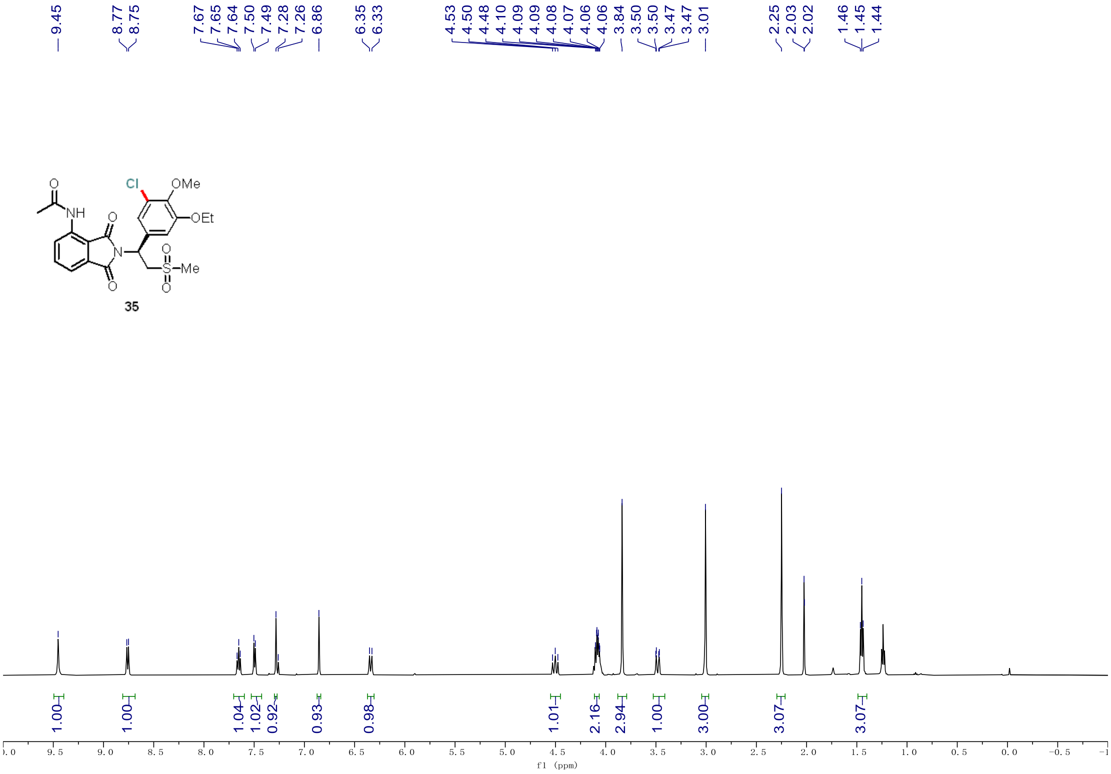

^{1}H NMR of compound **35**

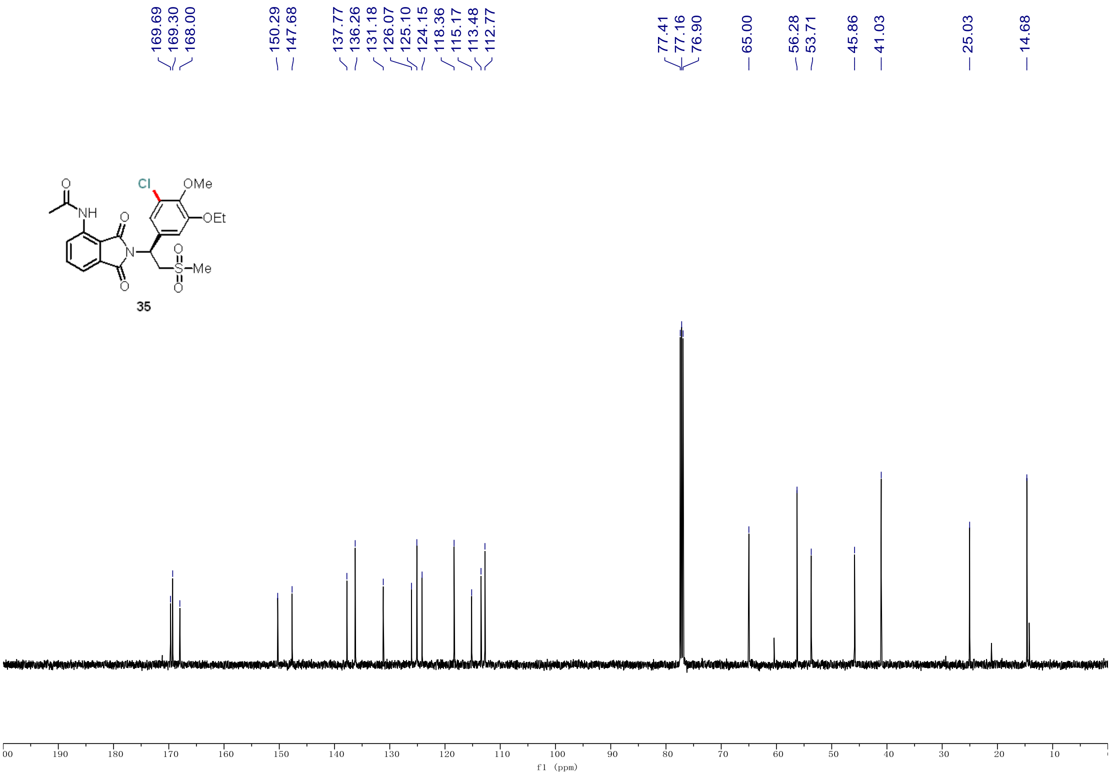

^{13}C NMR of compound **35**

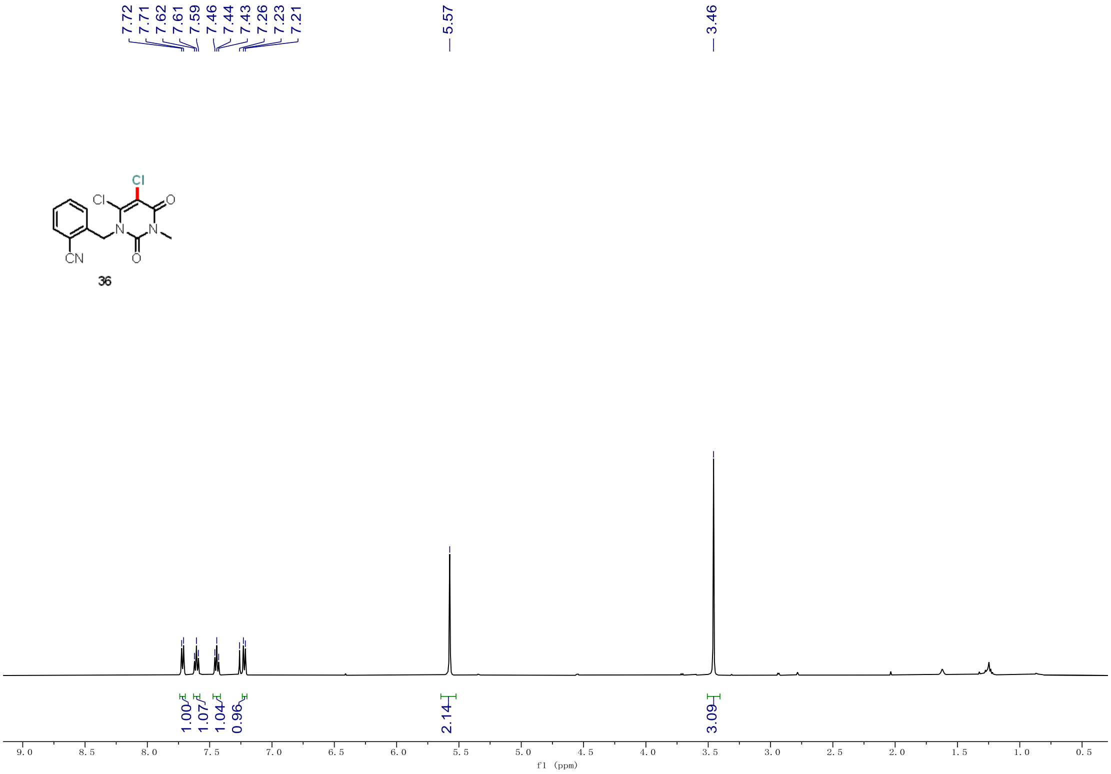

^{1}H NMR of compound **36**

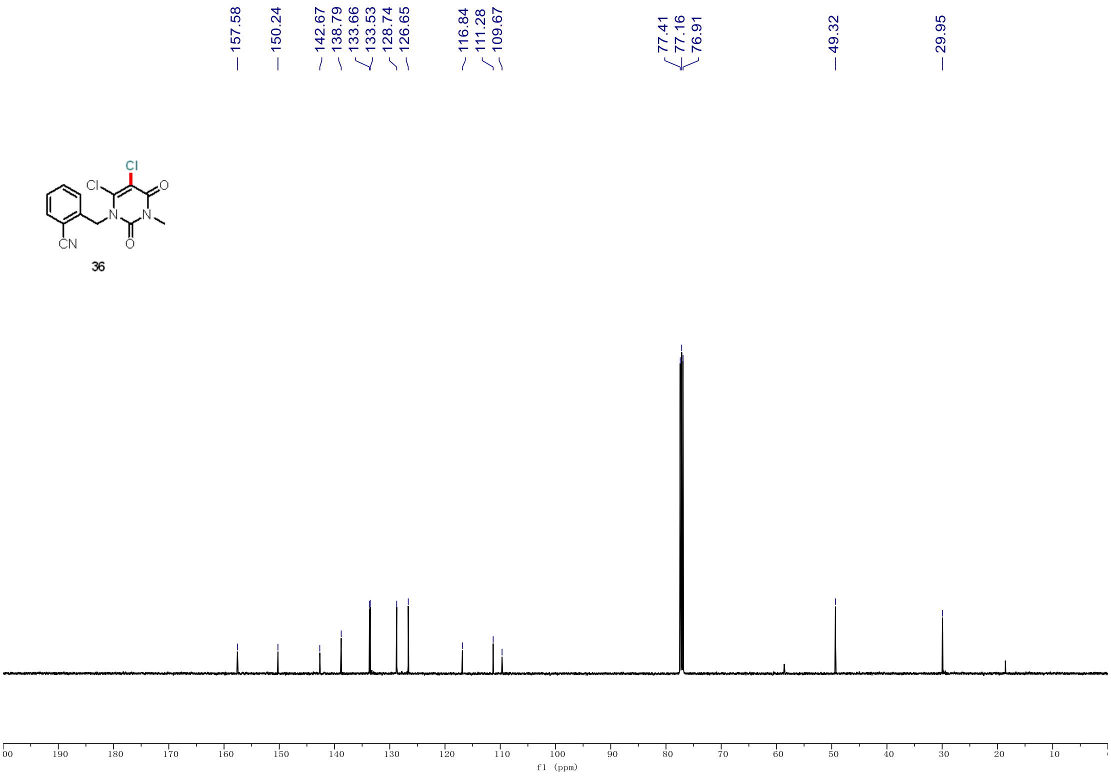

^{13}C NMR of compound **36**

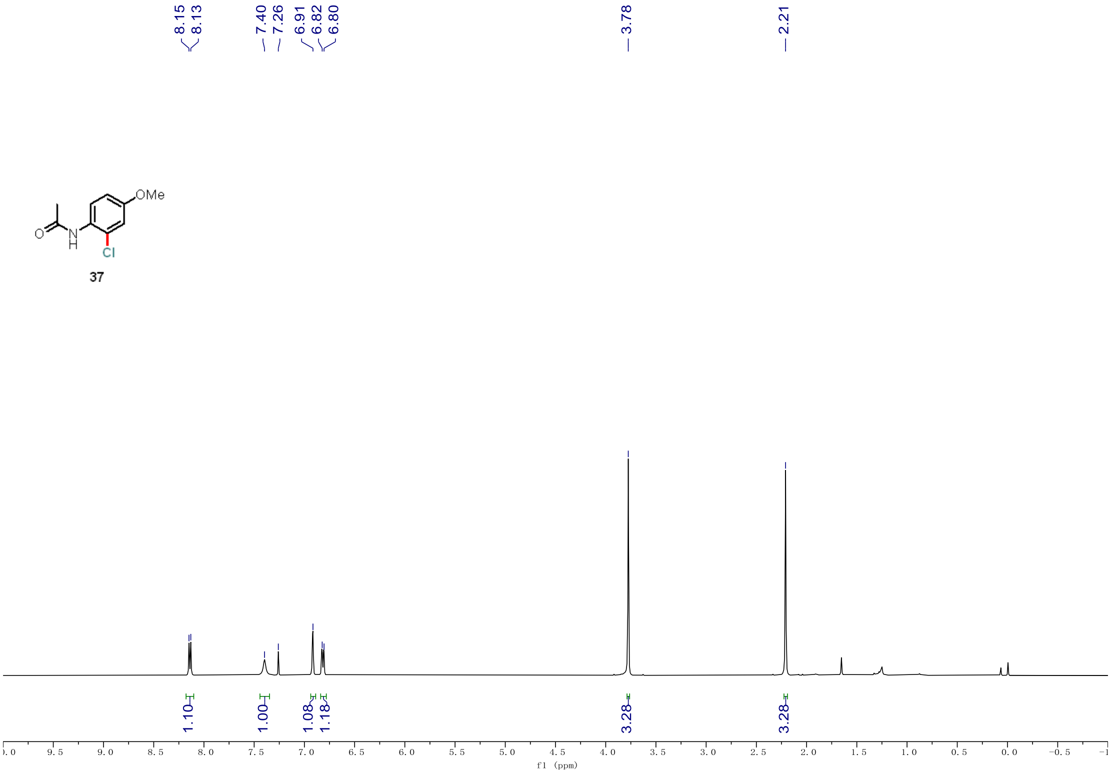

^{1}H NMR of compound **37**

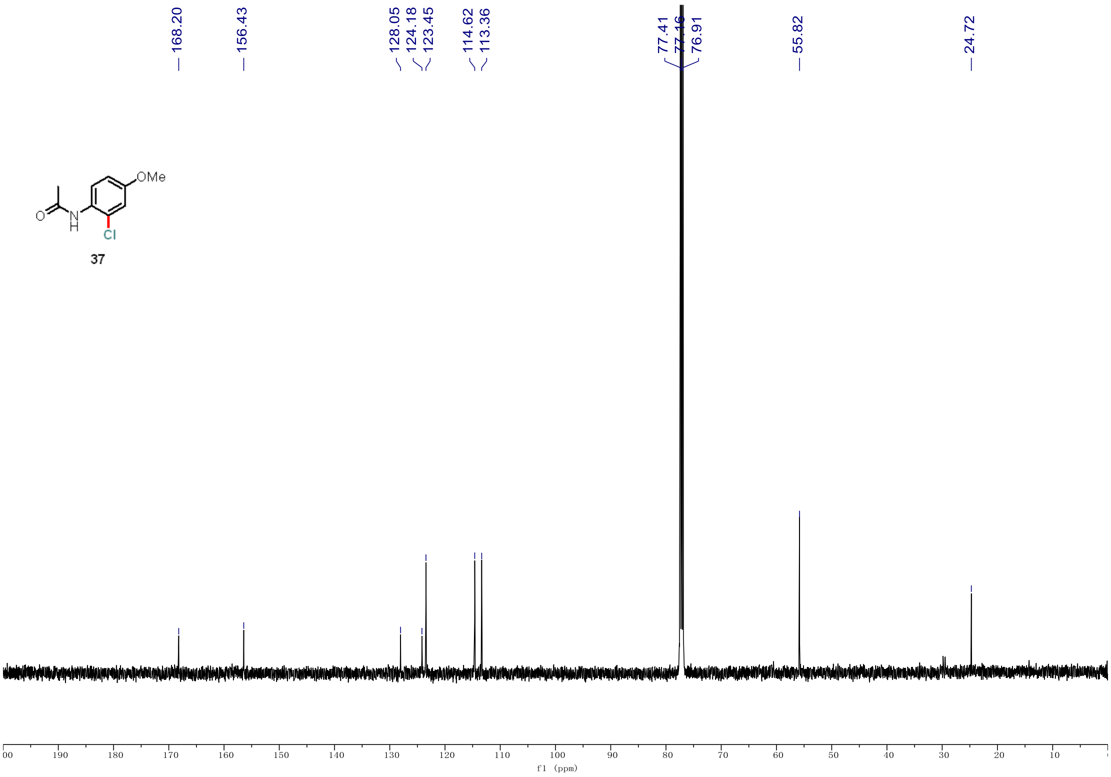

^{13}C NMR of compound **37**

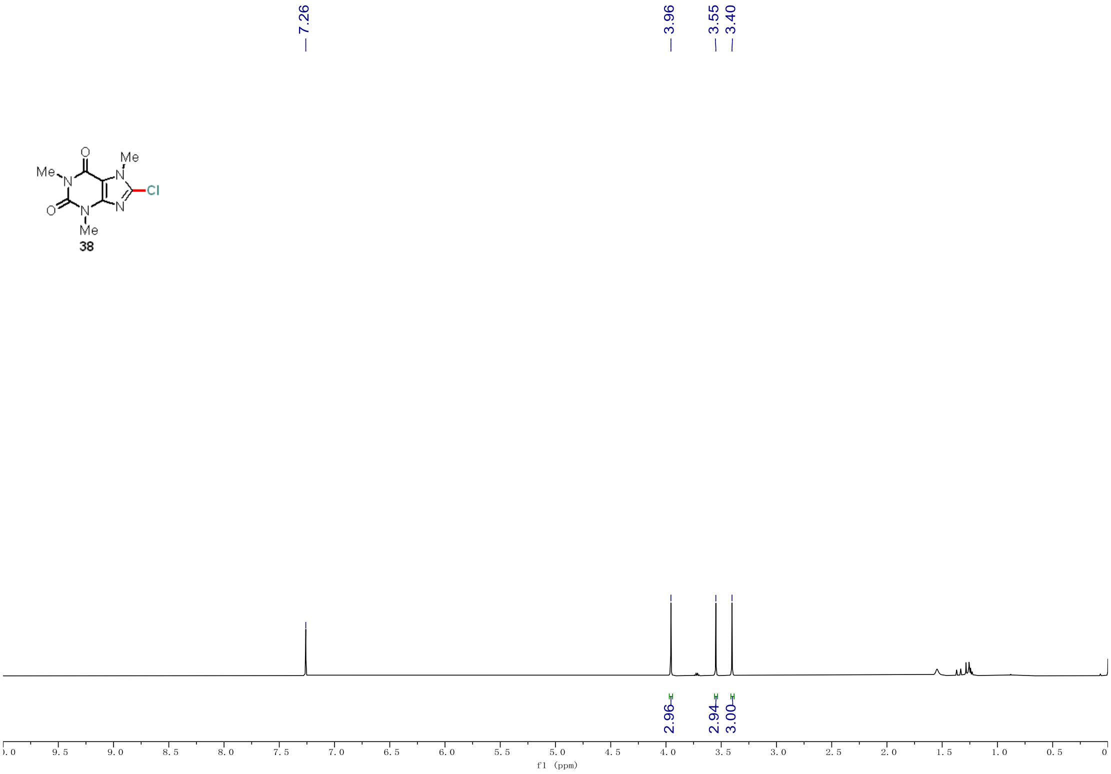

^{1}H NMR of compound **38**

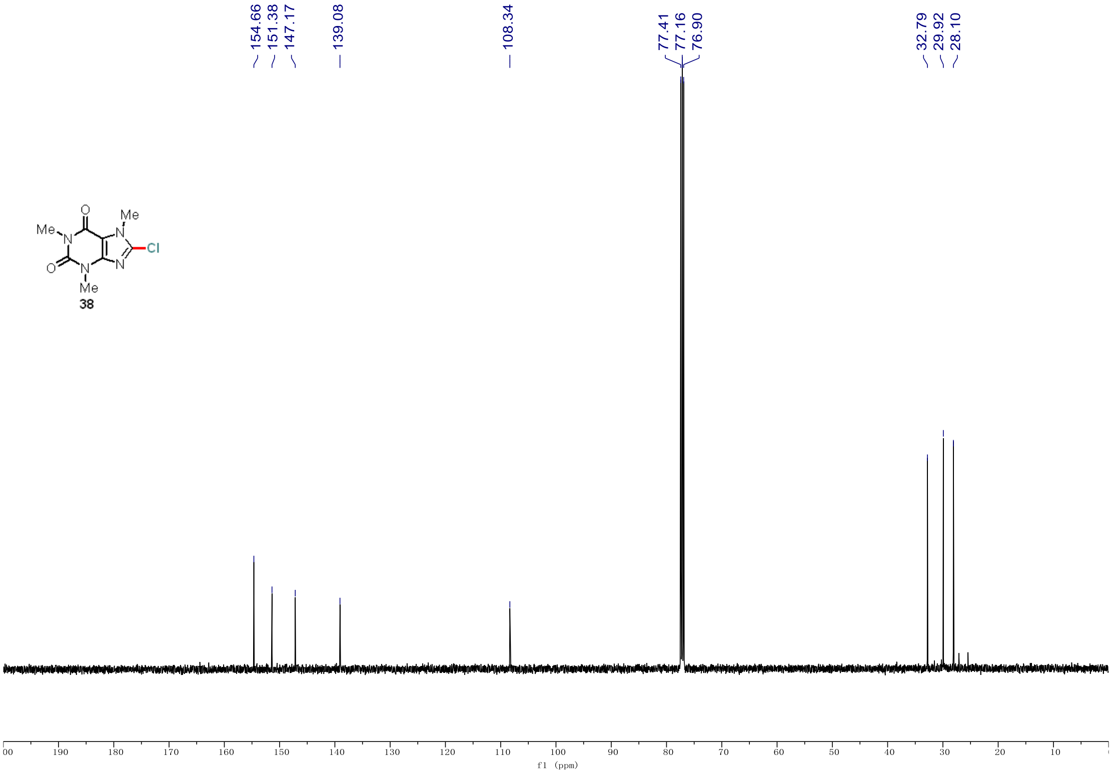

^{13}C NMR of compound **38**

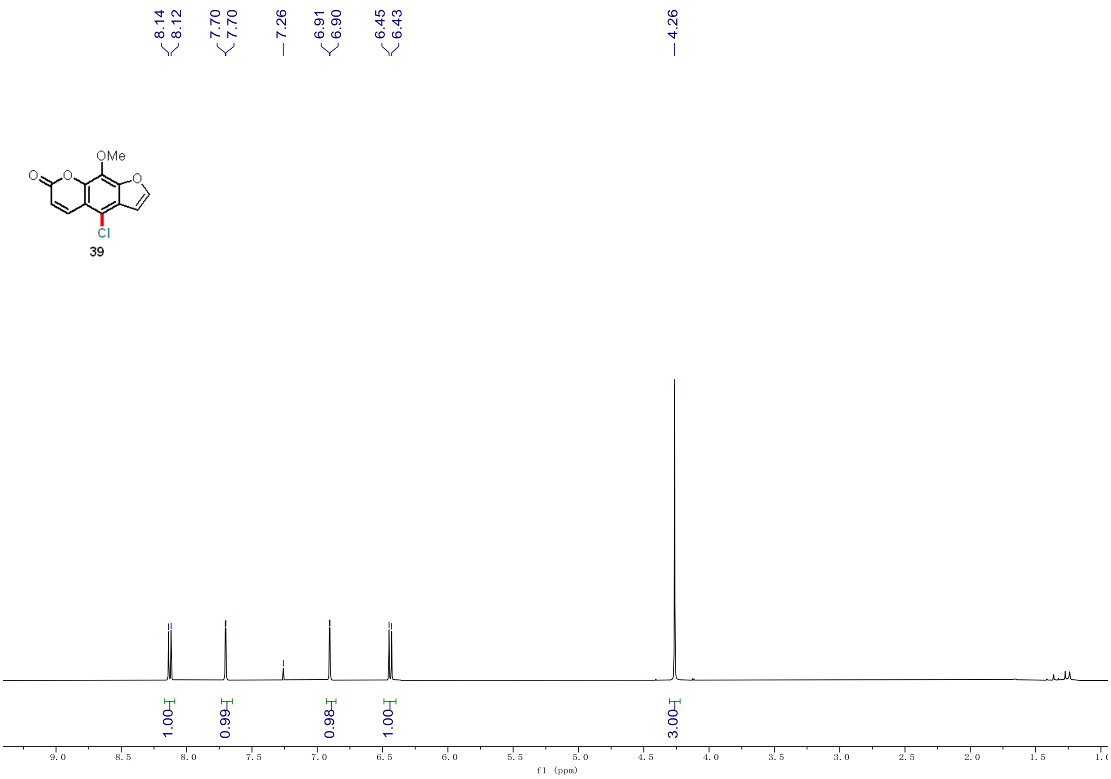

^{1}H NMR of compound **39**

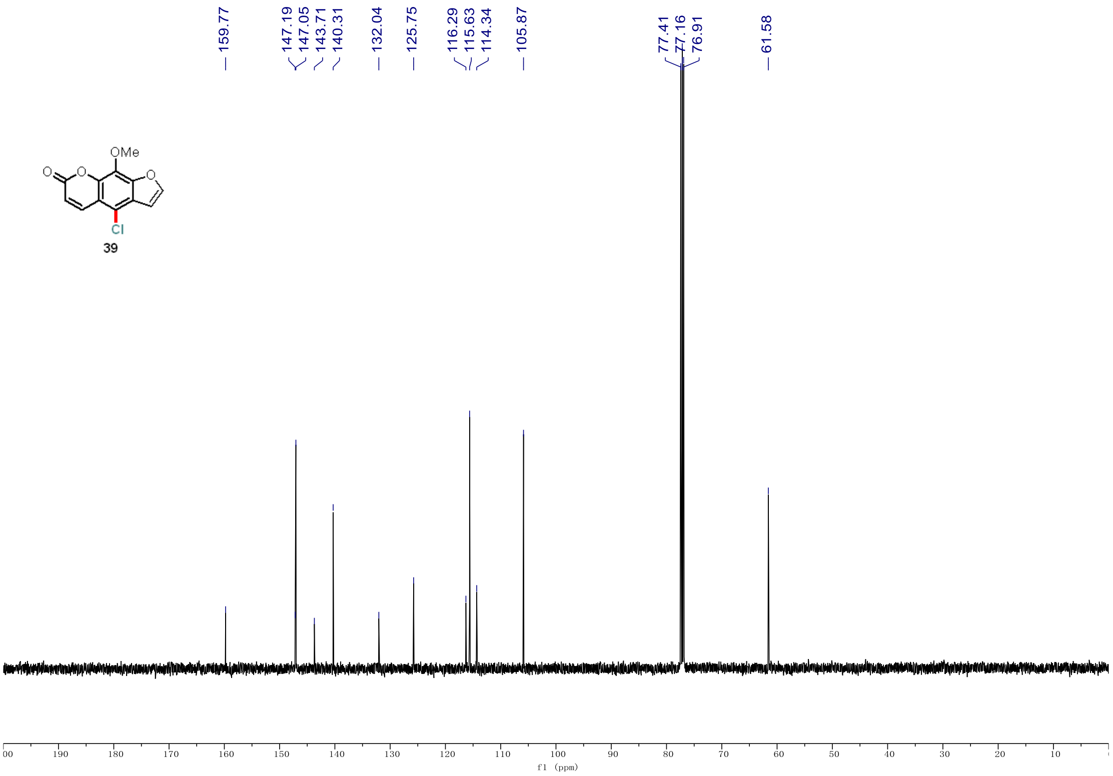

^{13}C NMR of compound **39**

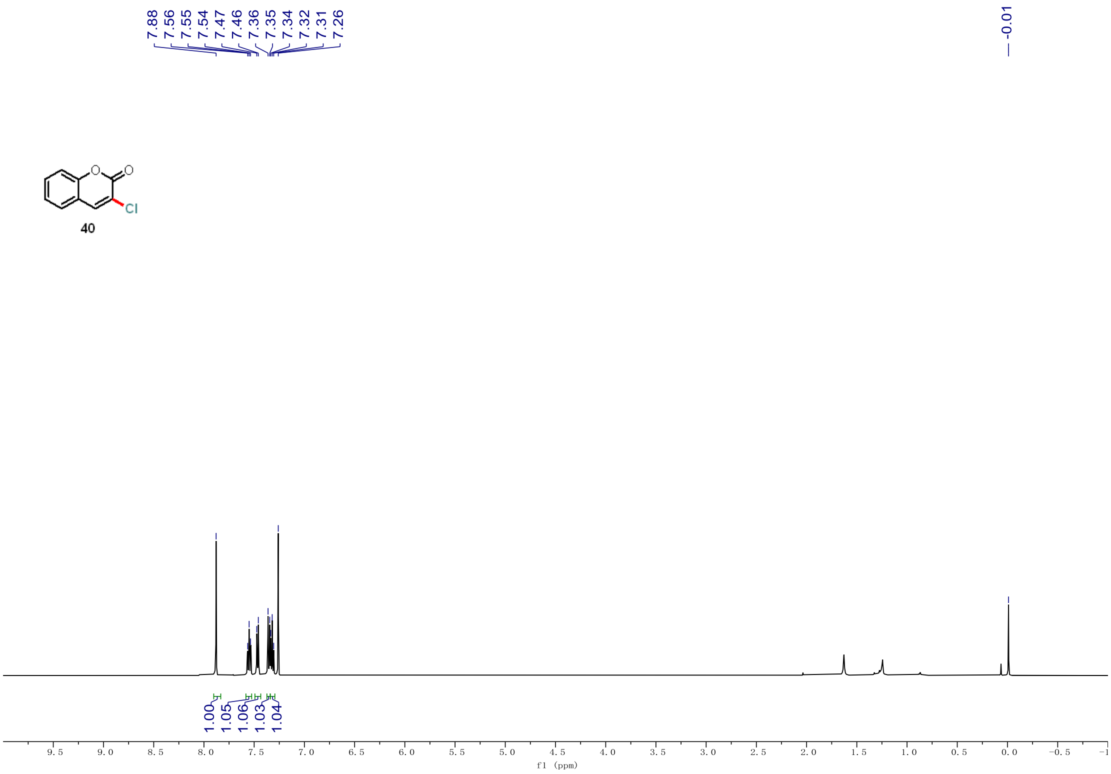

^{1}H NMR of compound **40**

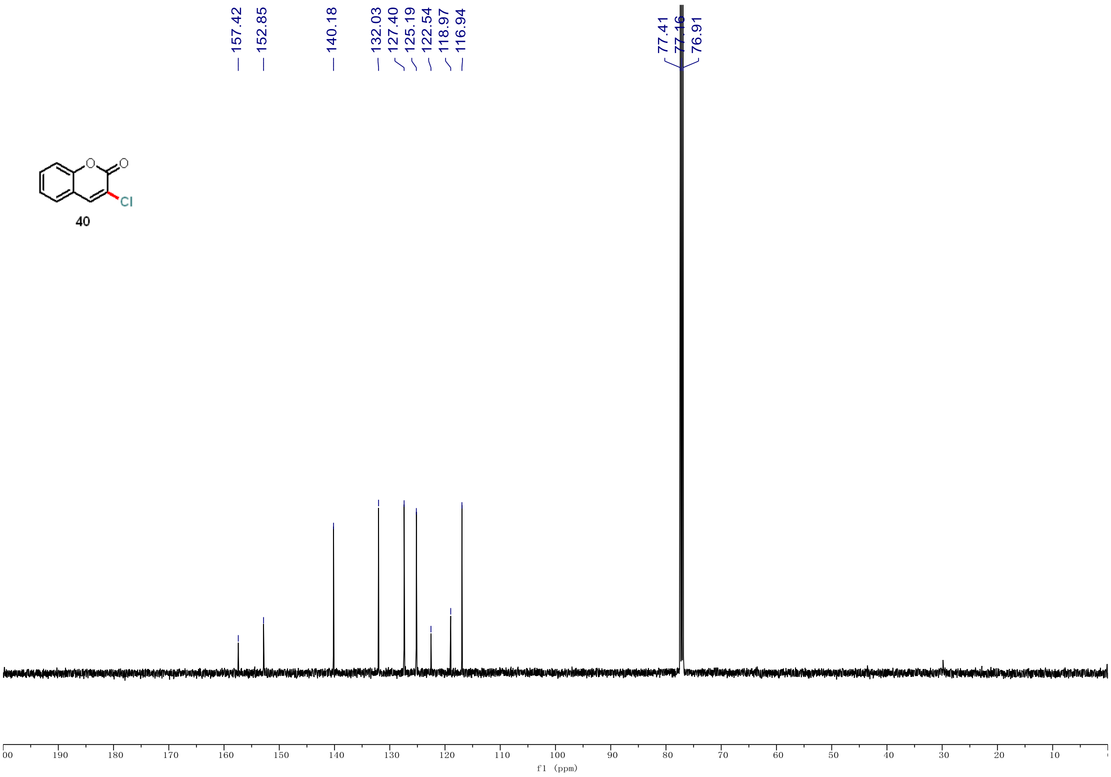

^{13}C NMR of compound **40**

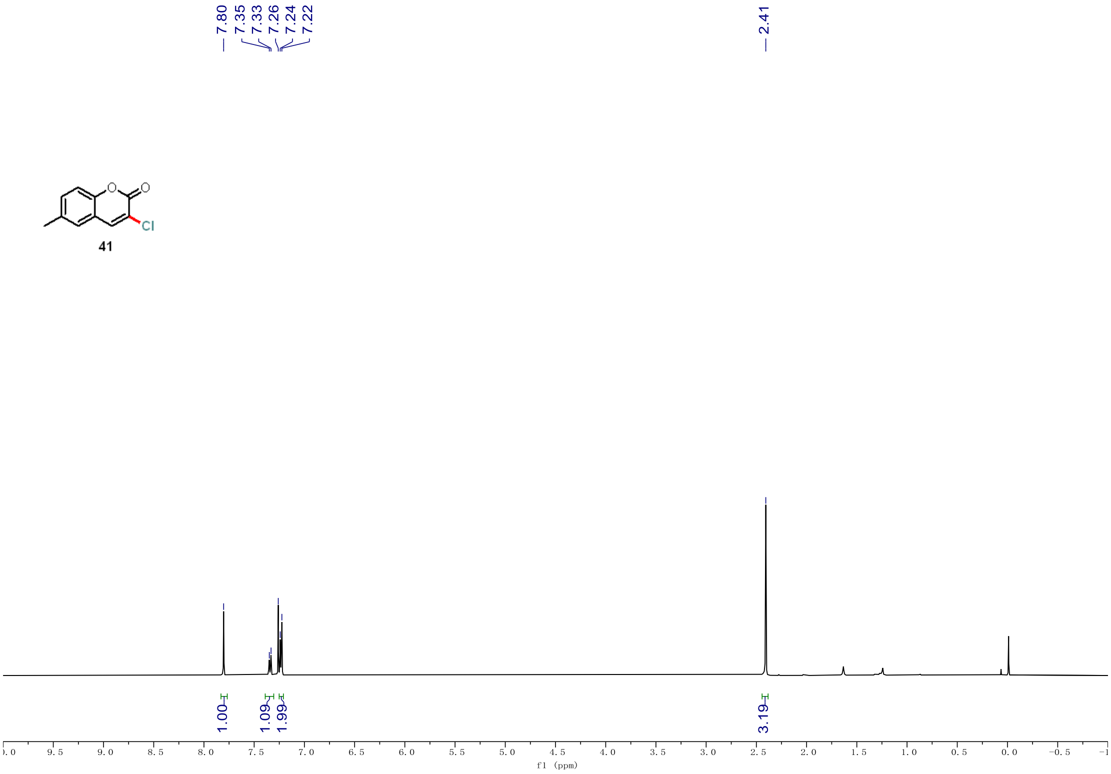

^{1}H NMR of compound **41**

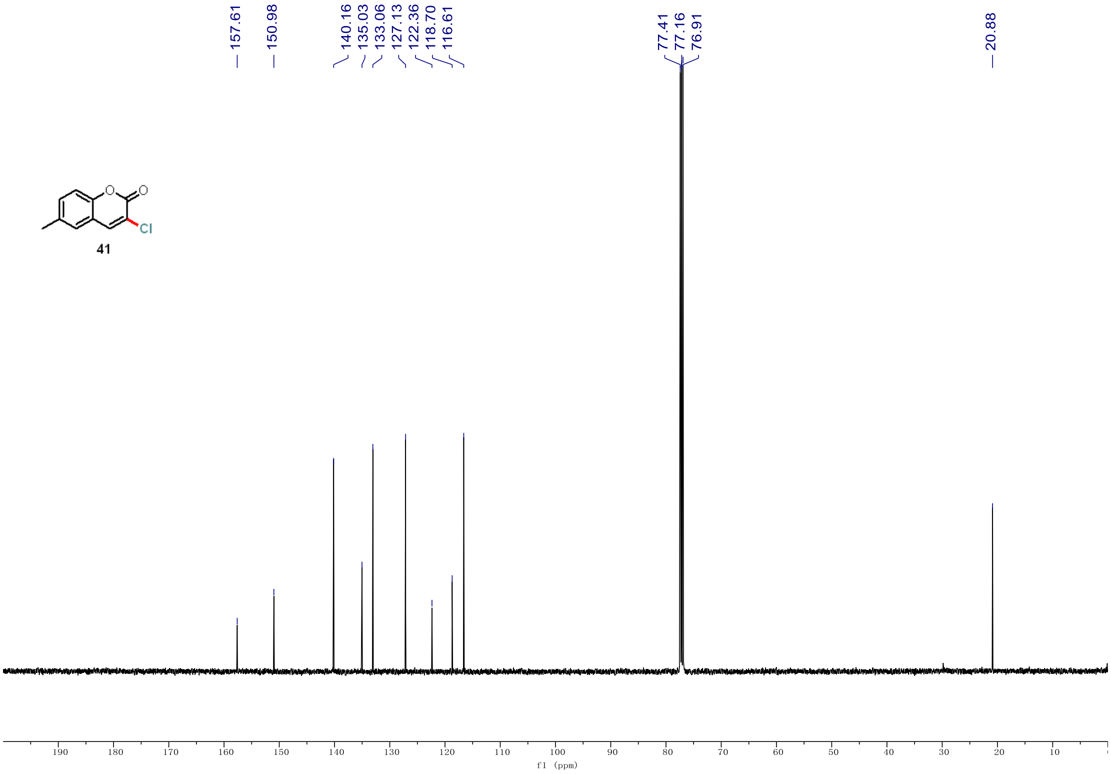

^{13}C NMR of compound **41**

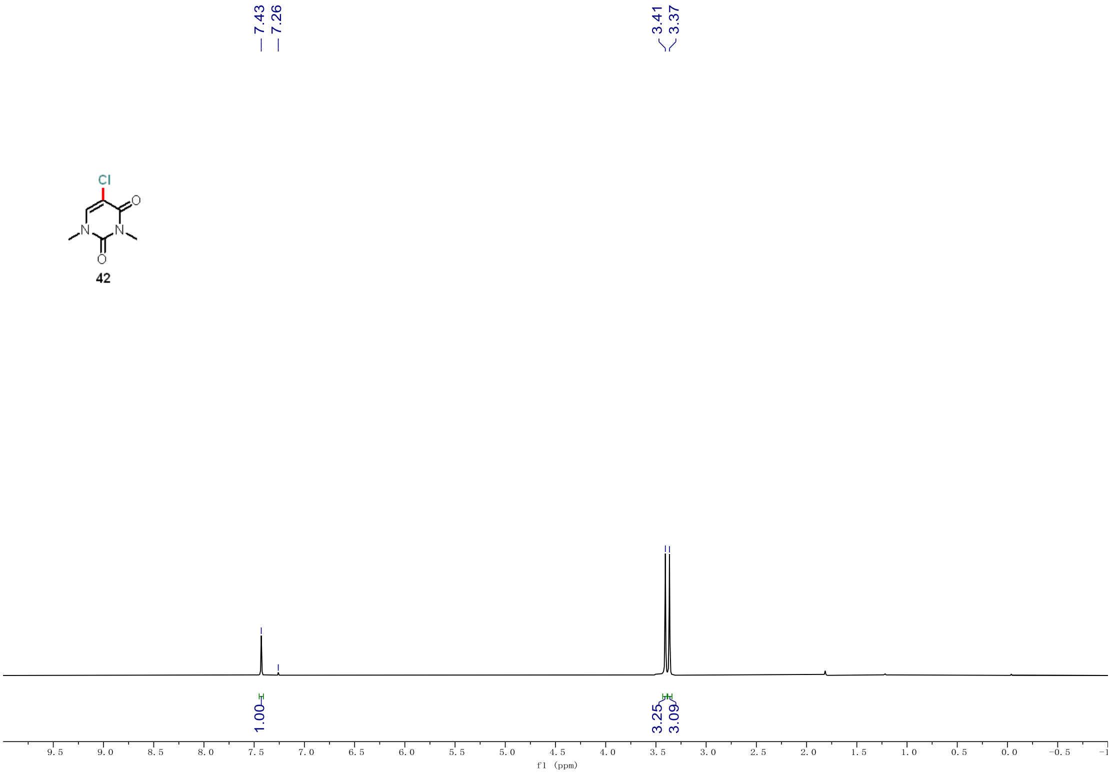

^{1}H NMR of compound **42**

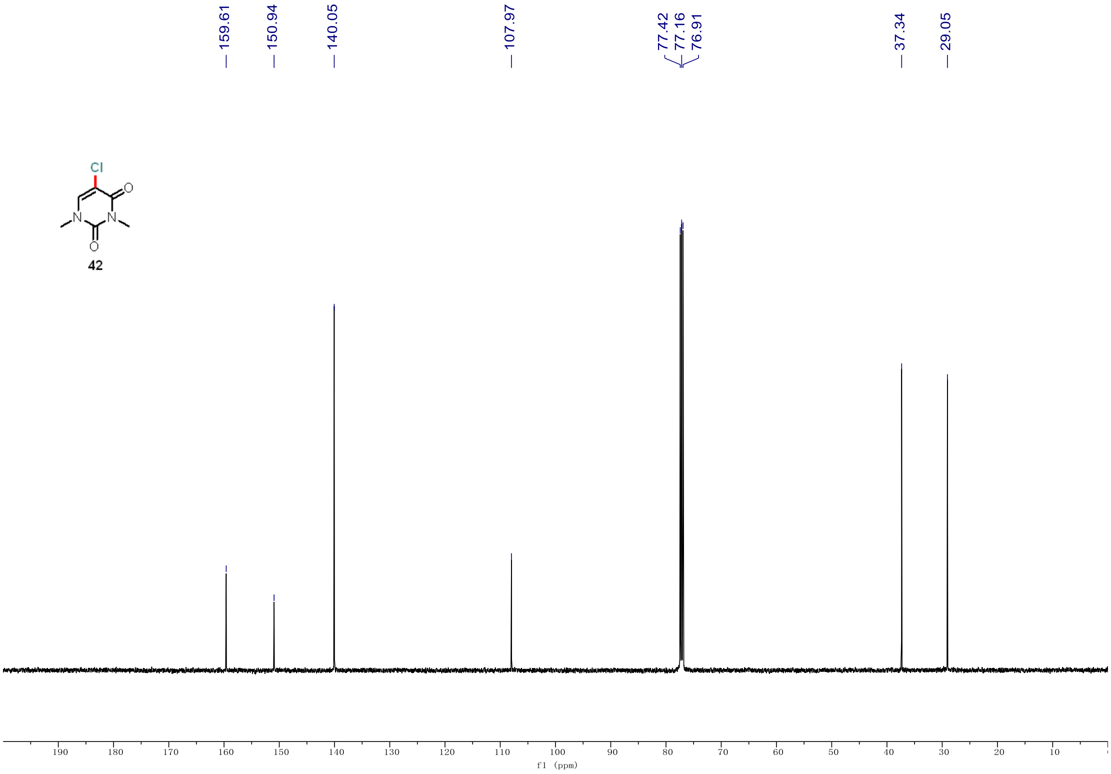

^{13}C NMR of compound **42**

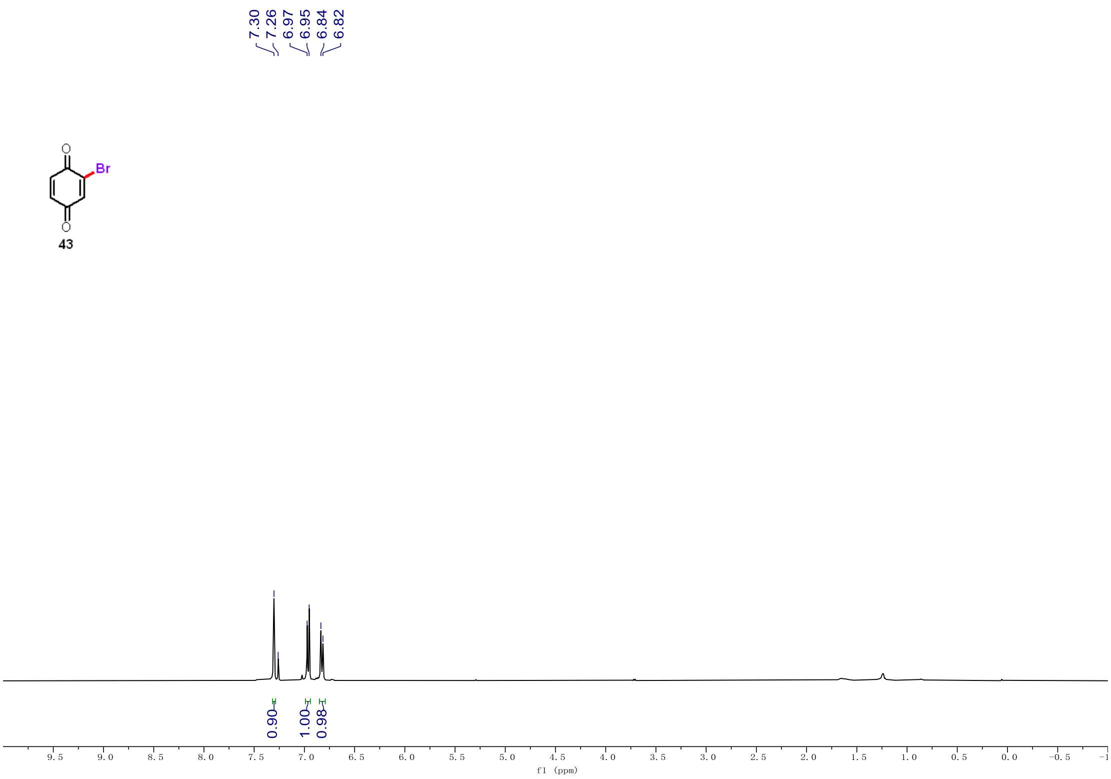

^{1}H NMR of compound **43**

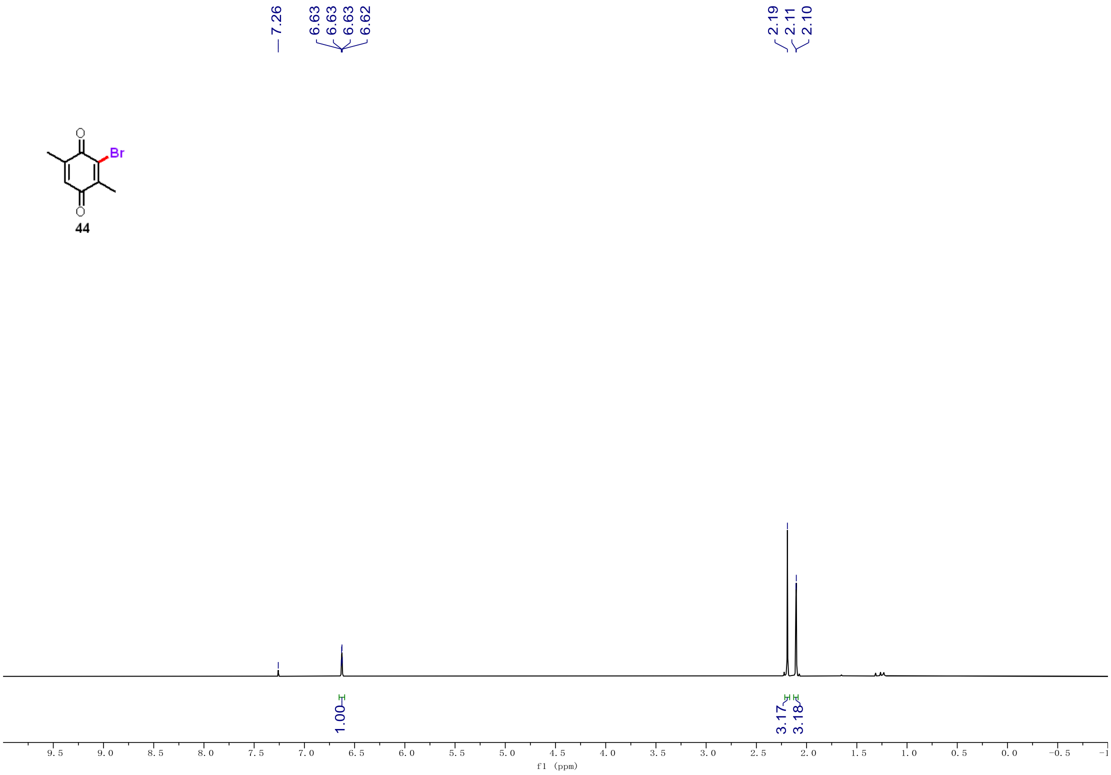

^{1}H NMR of compound **44**

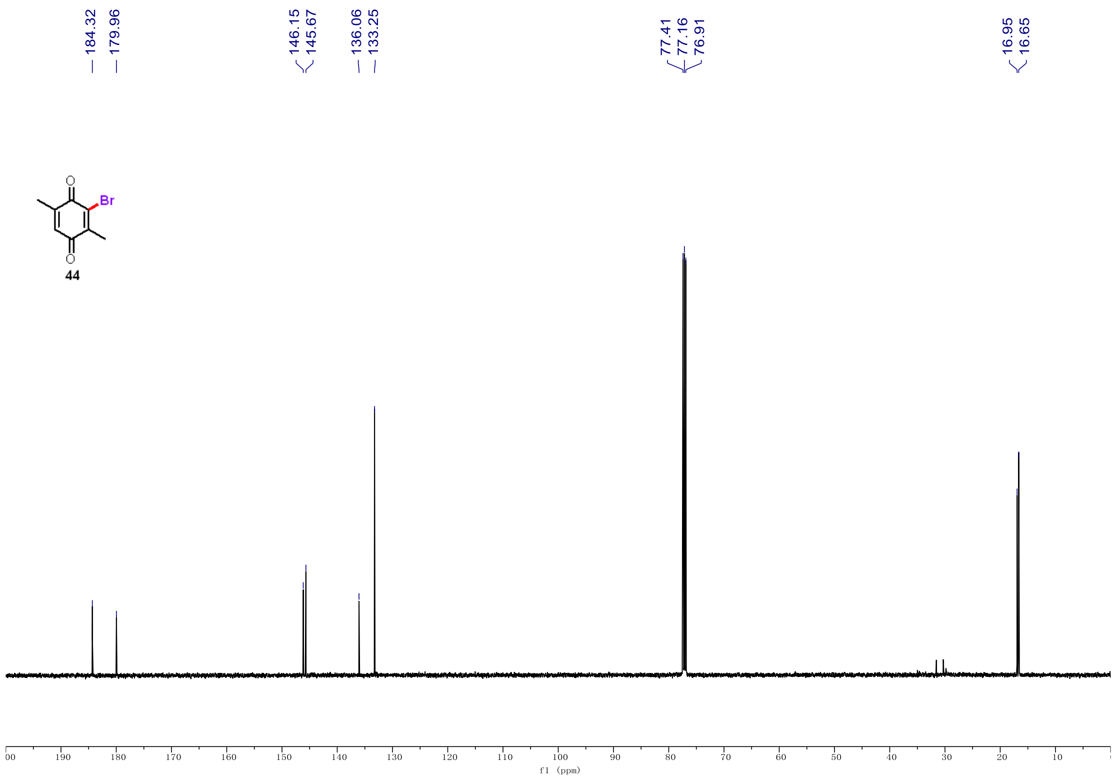

^{13}C NMR of compound **44**

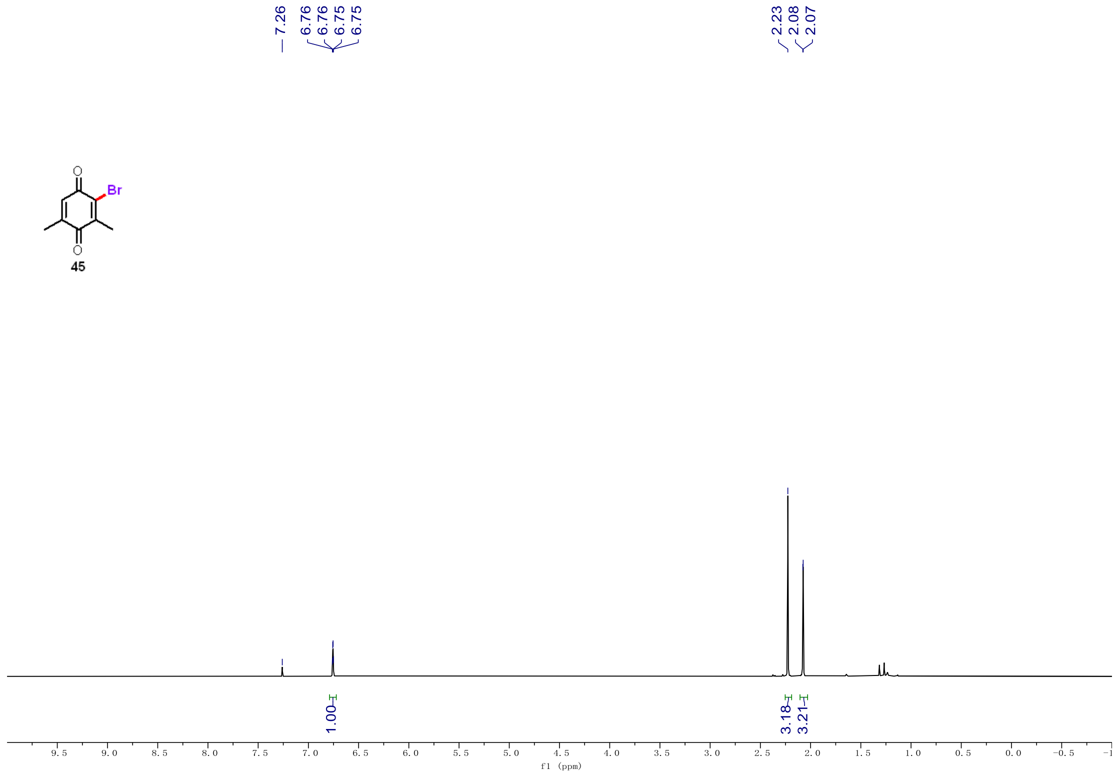

^{1}H NMR of compound **45**

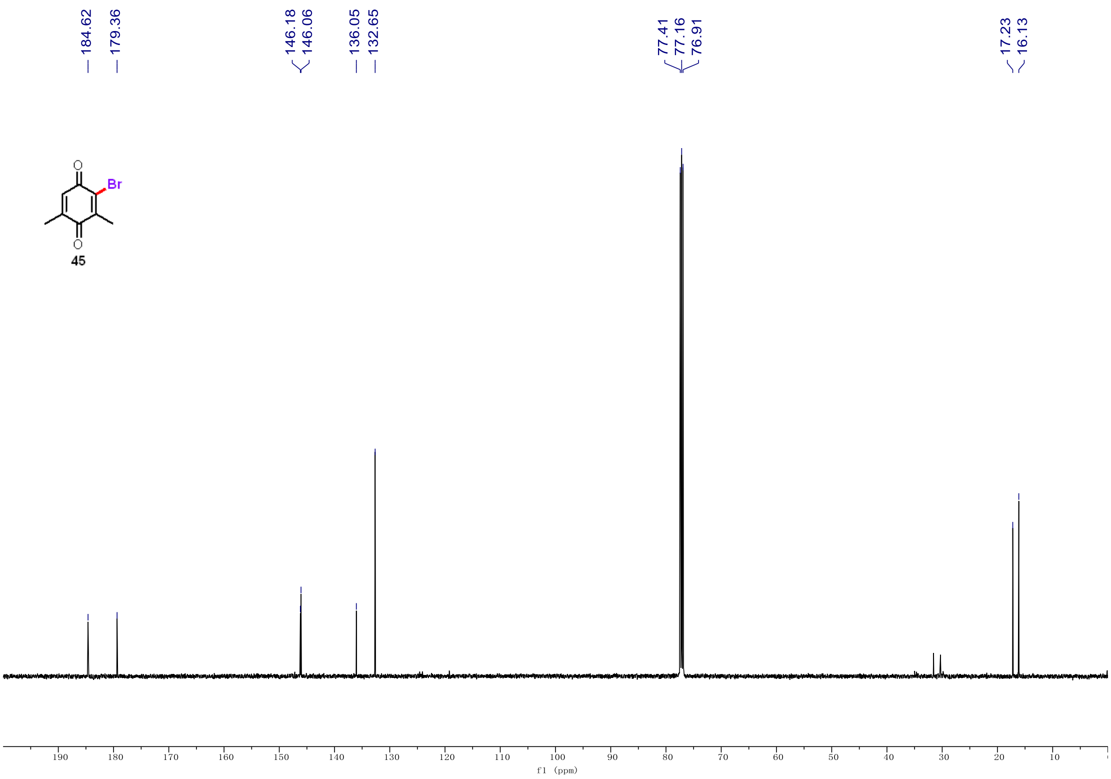

^{13}C NMR of compound **45**

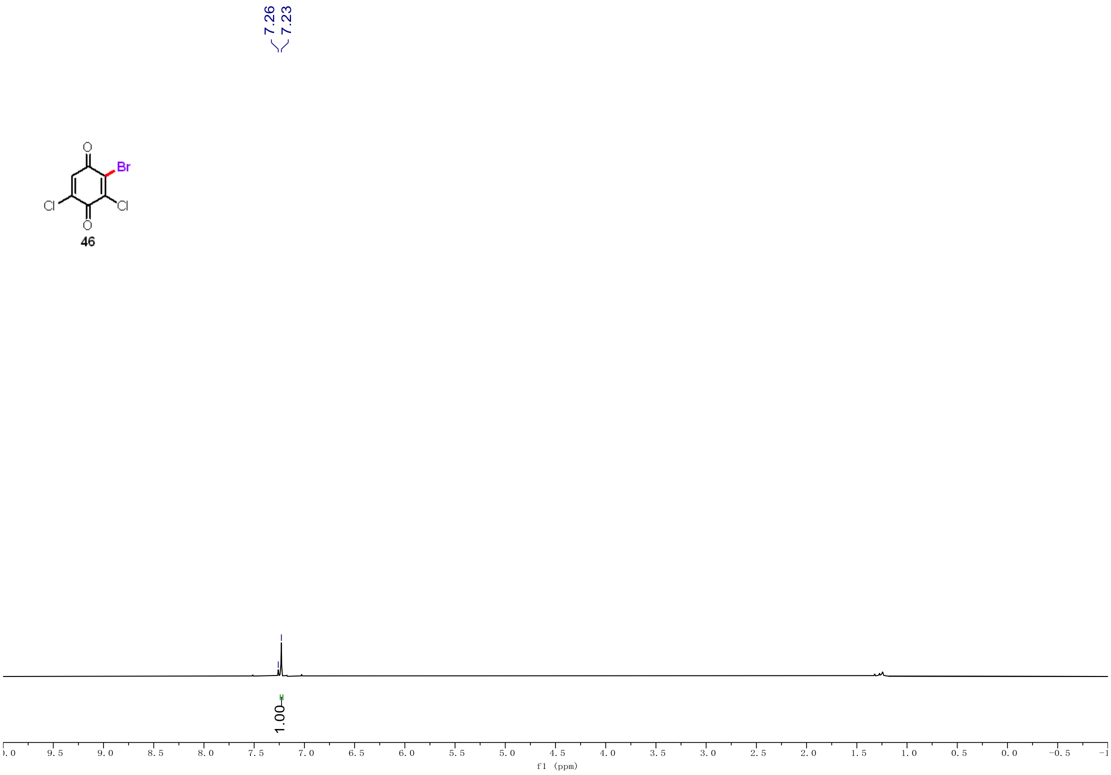

^{1}H NMR of compound **46**

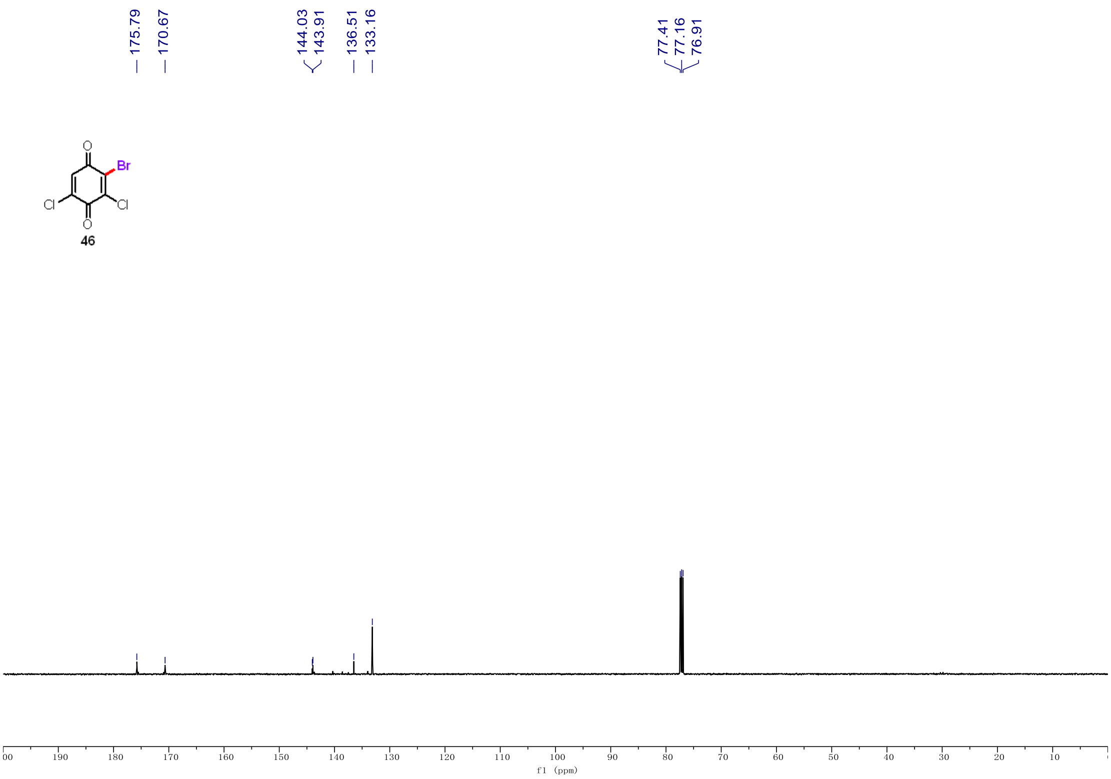

^{13}C NMR of compound **46**

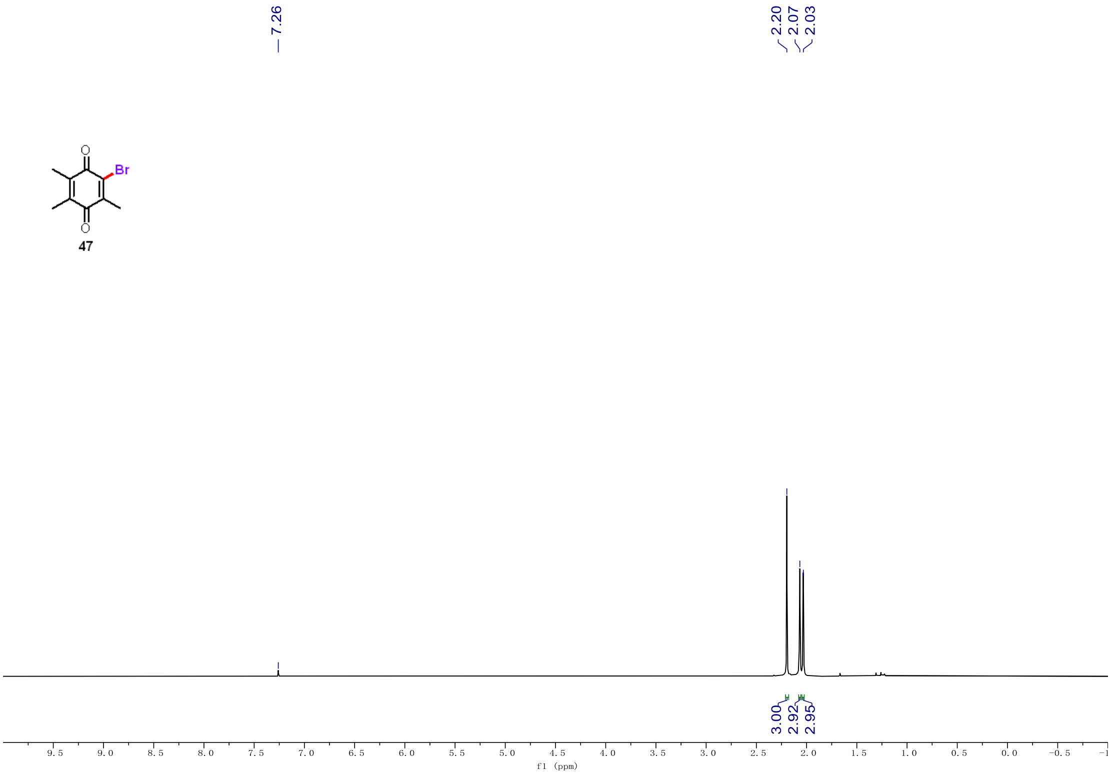

^{1}H NMR of compound **47**

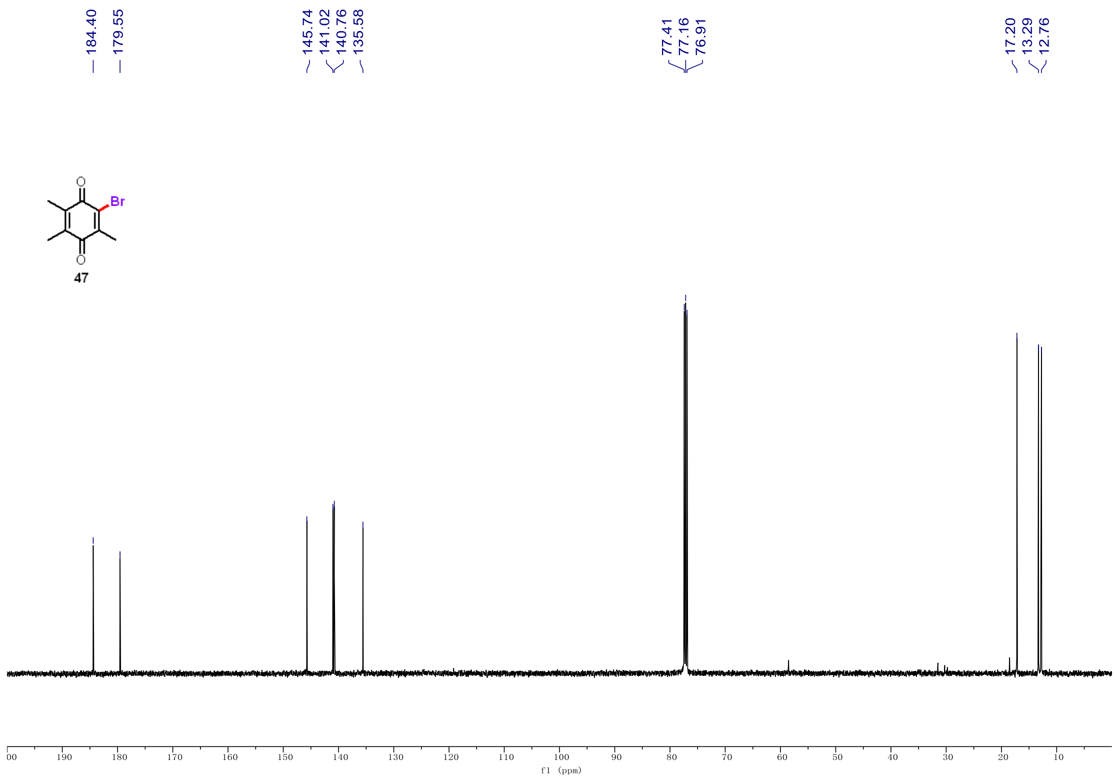

^{13}C NMR of compound **47**

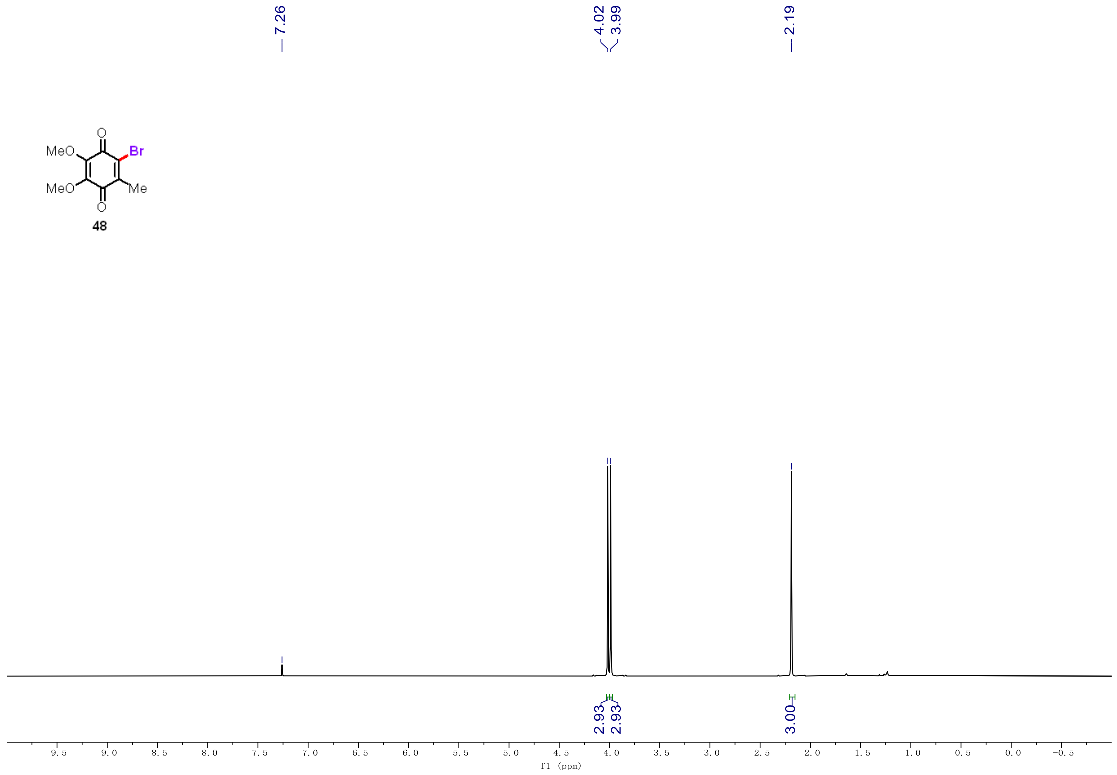

^{1}H NMR of compound **48**

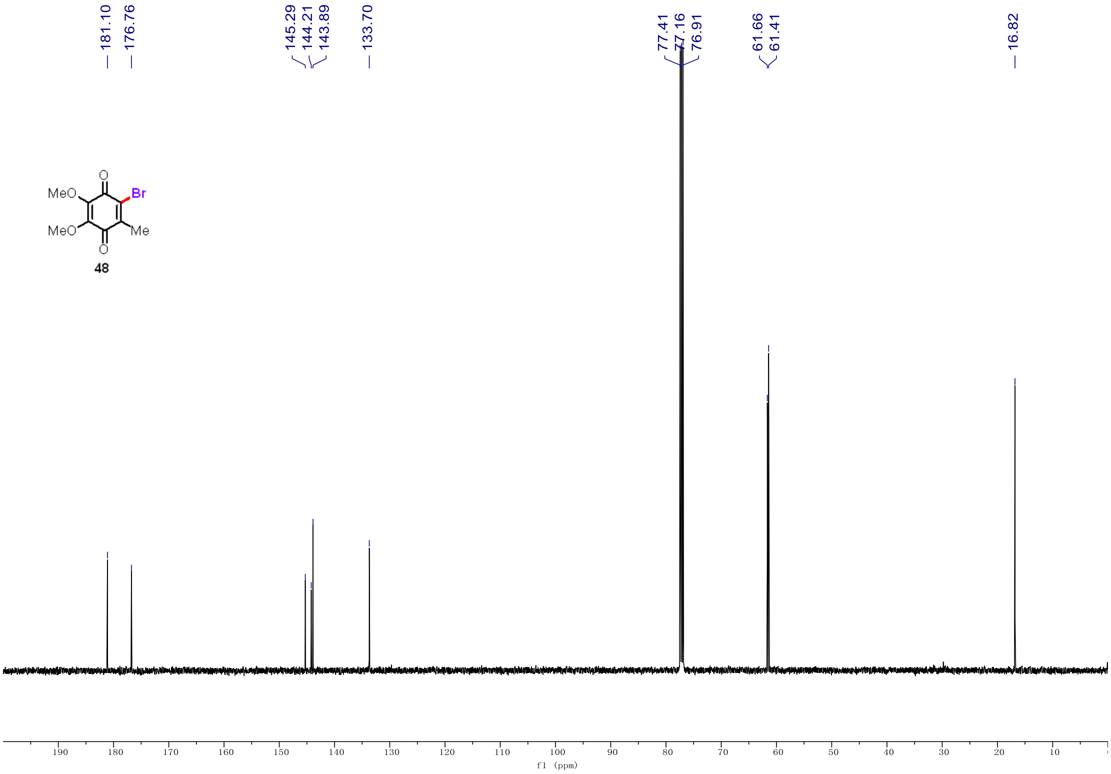

^{13}C NMR of compound **48**

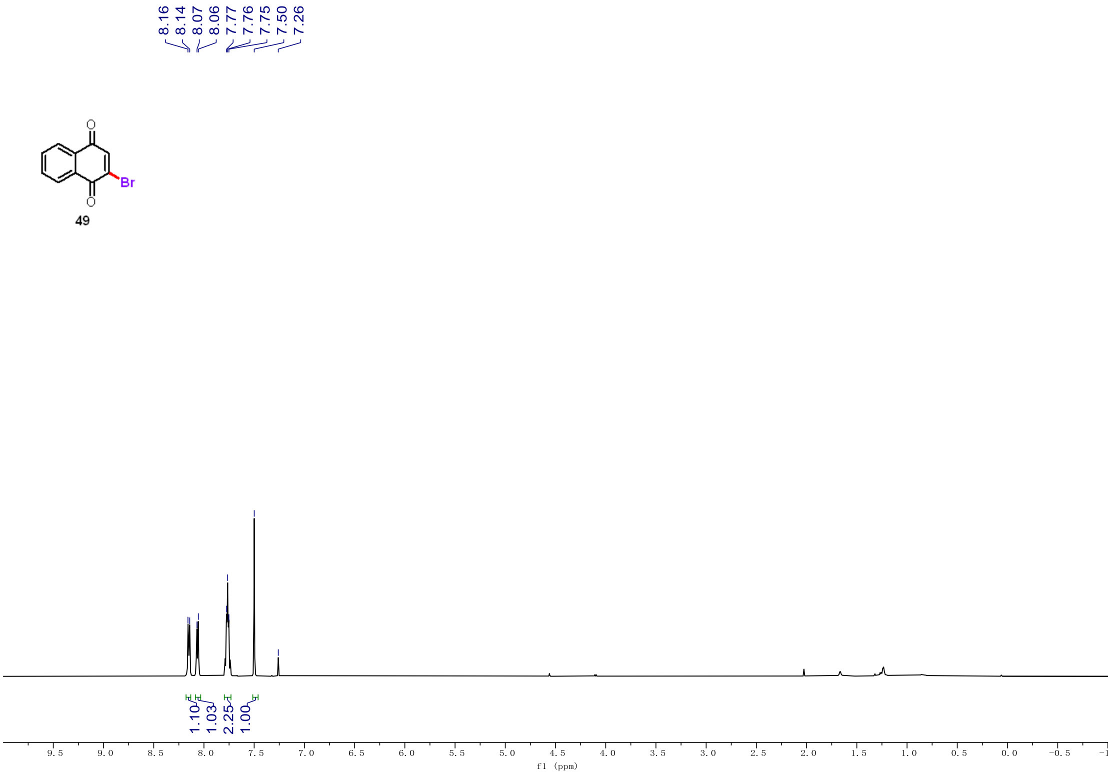

^{1}H NMR of compound **49**

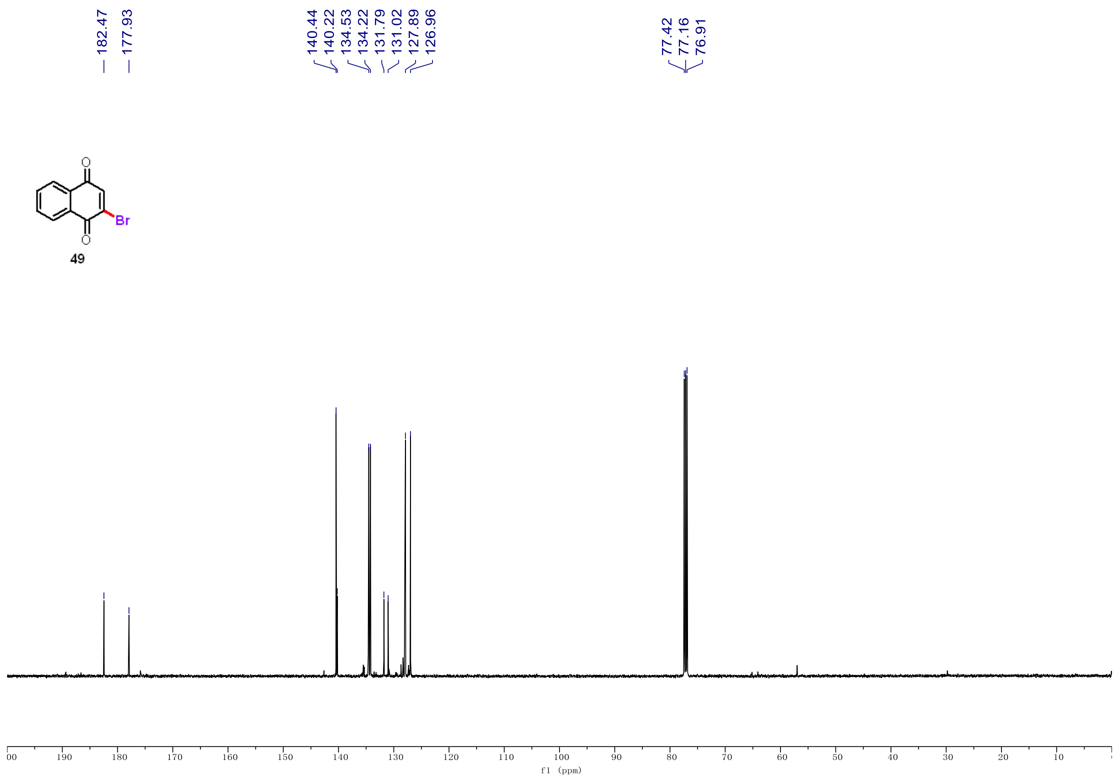

^{13}C NMR of compound **49**

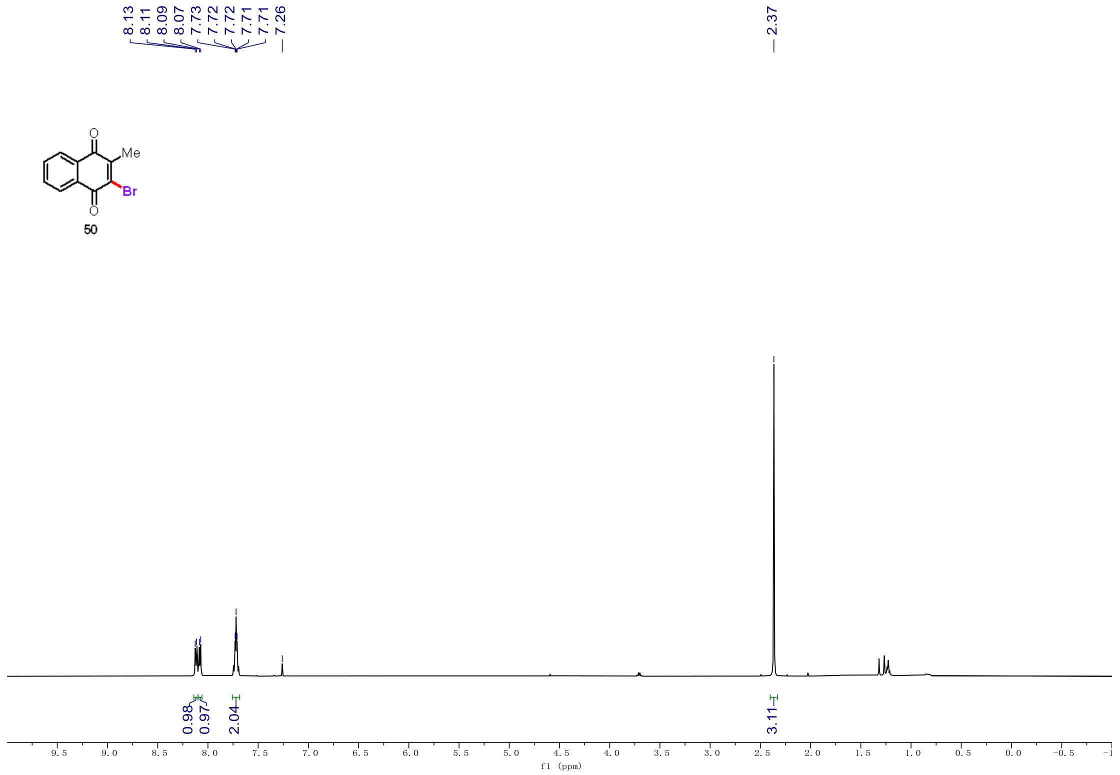

^{1}H NMR of compound **50**

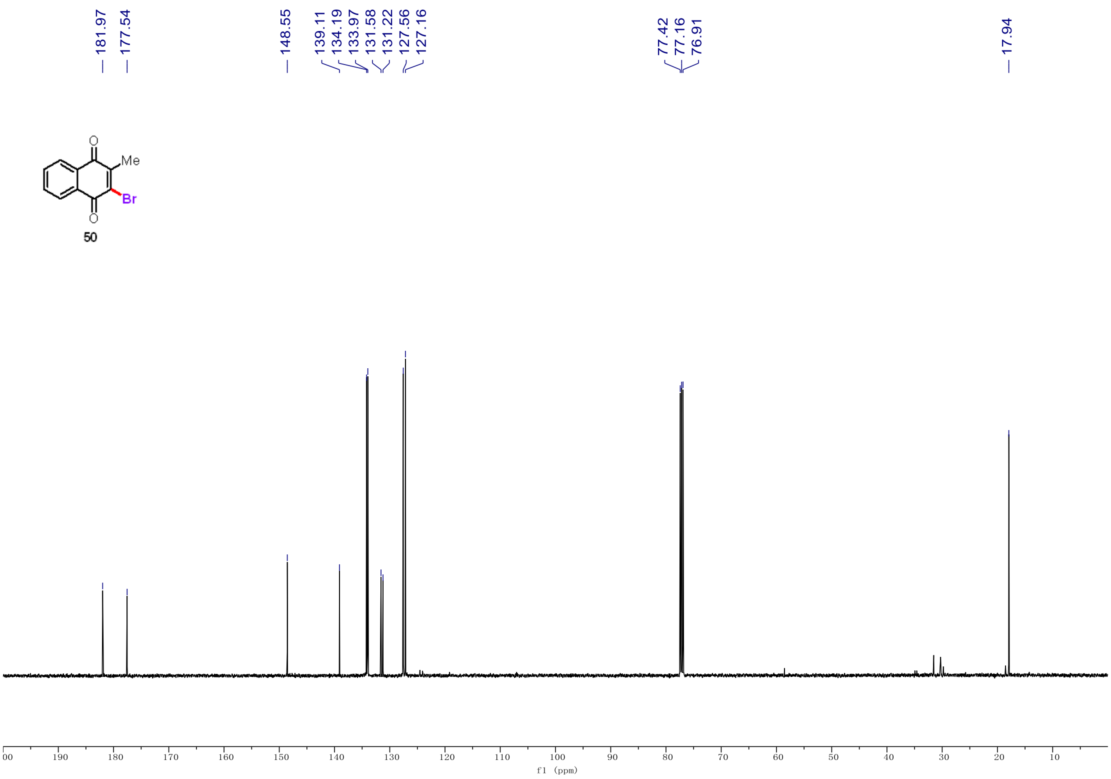

^{13}C NMR of compound **50**

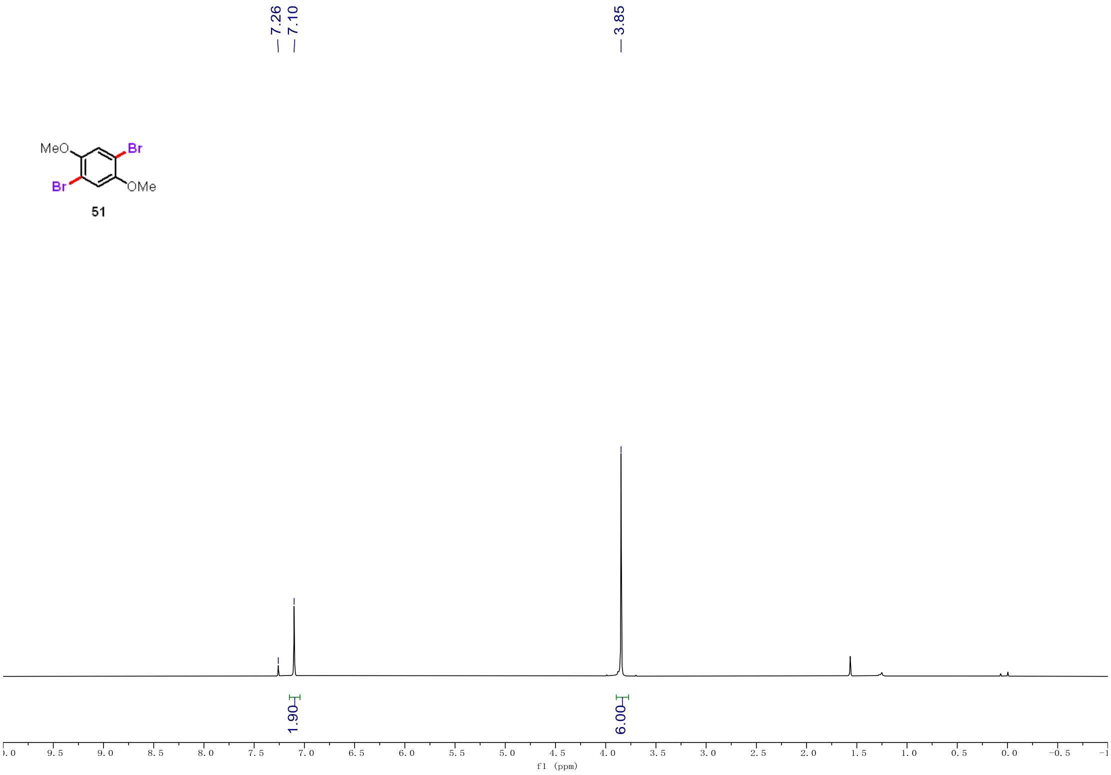

^{1}H NMR of compound **51**

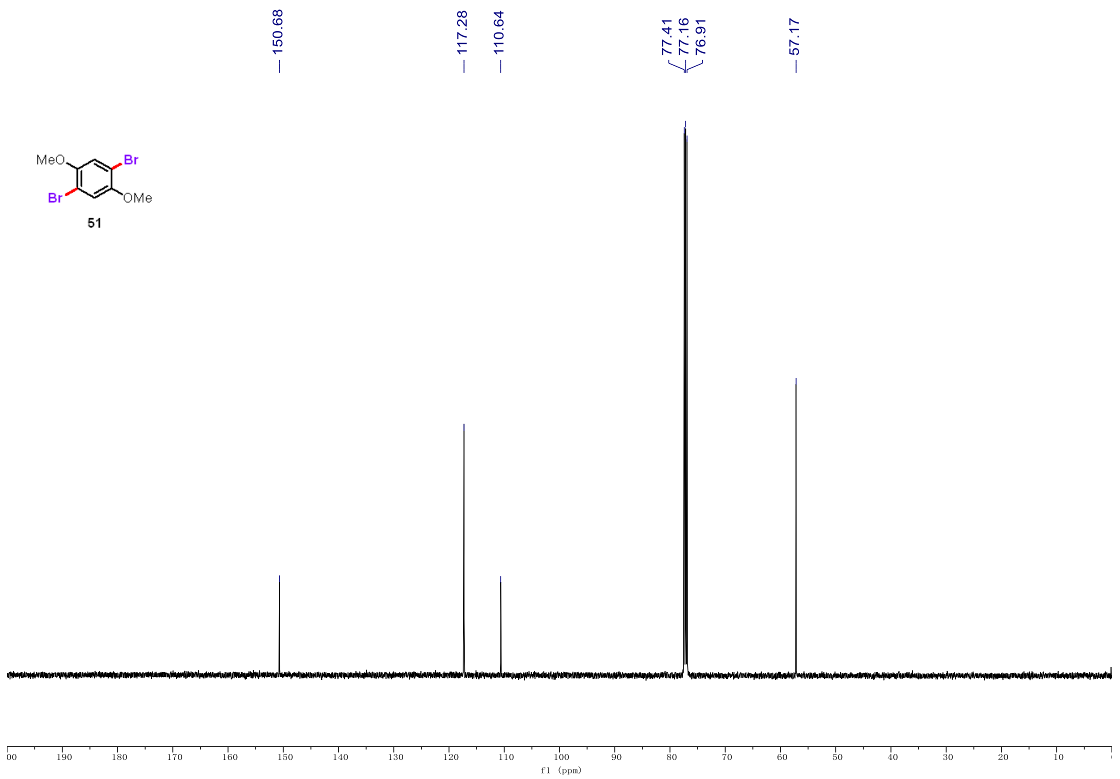

^{13}C NMR of compound **51**

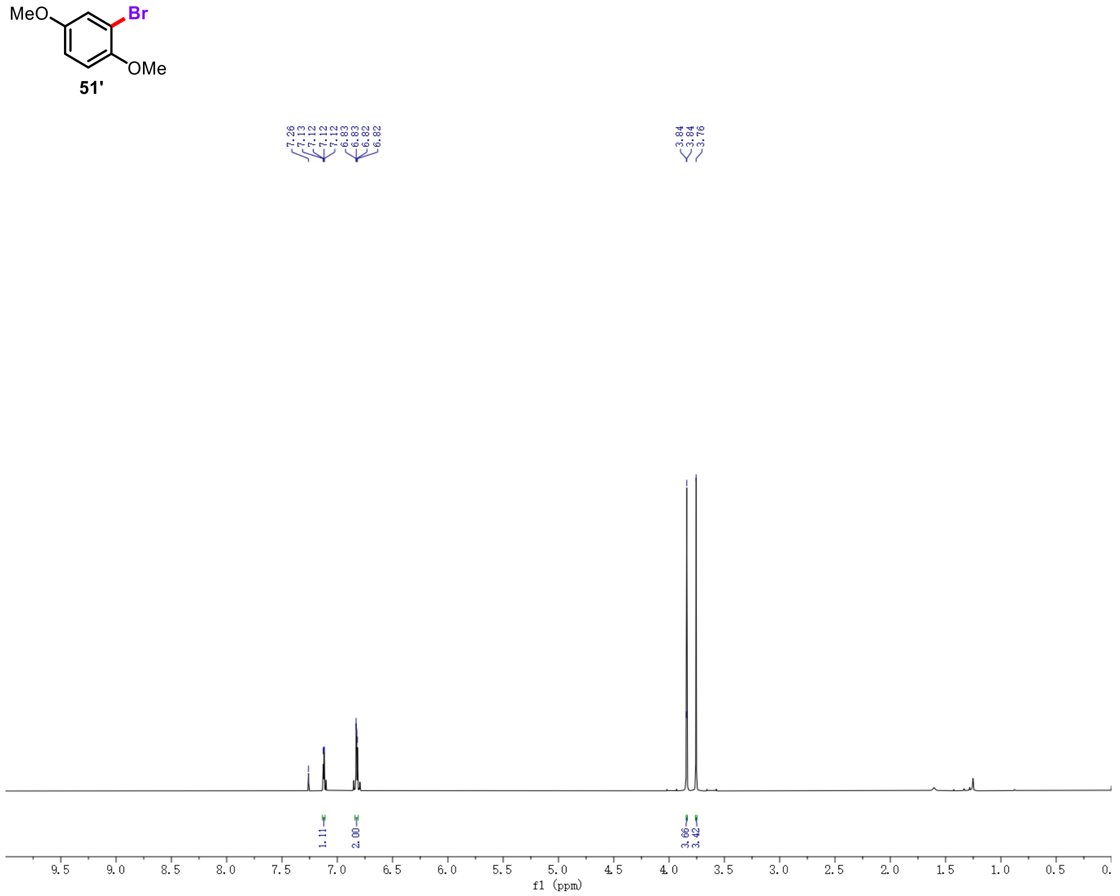

^{1}H NMR of compound **51’**

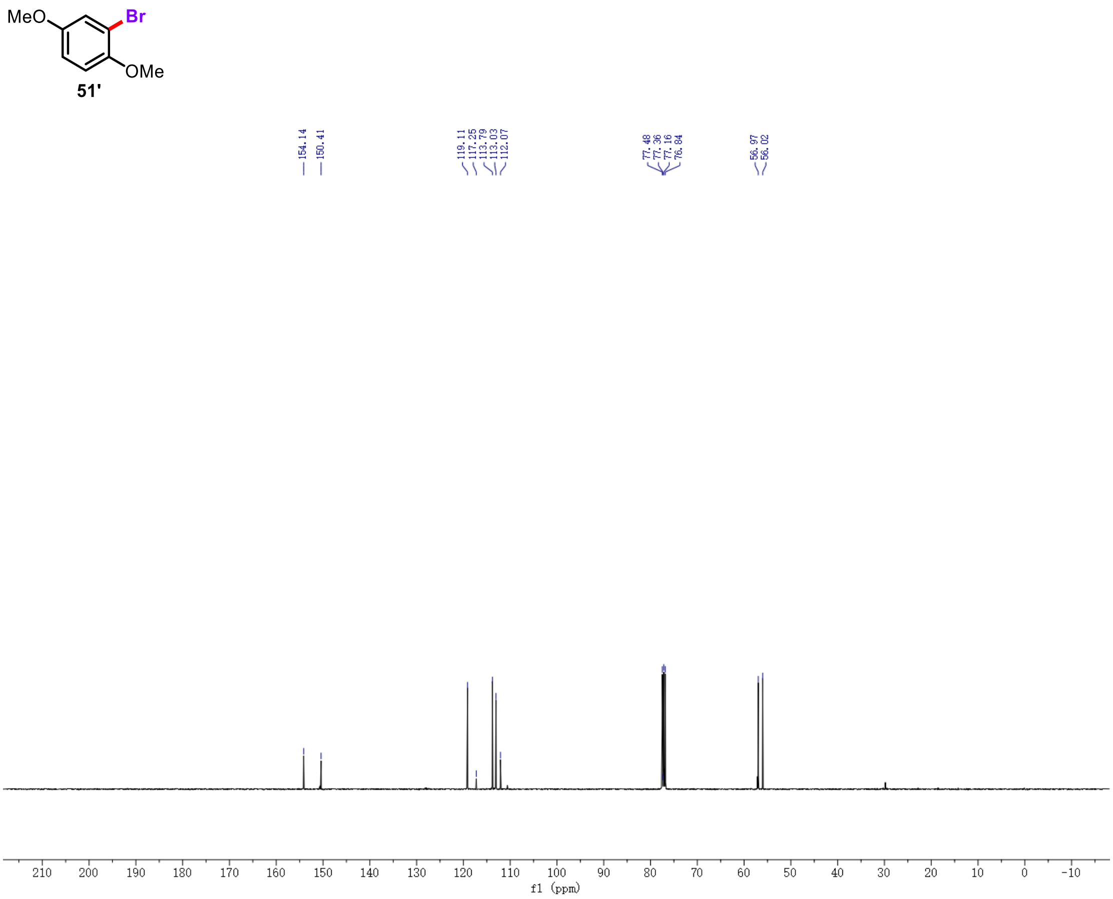

^{13}C NMR of compound **51’**

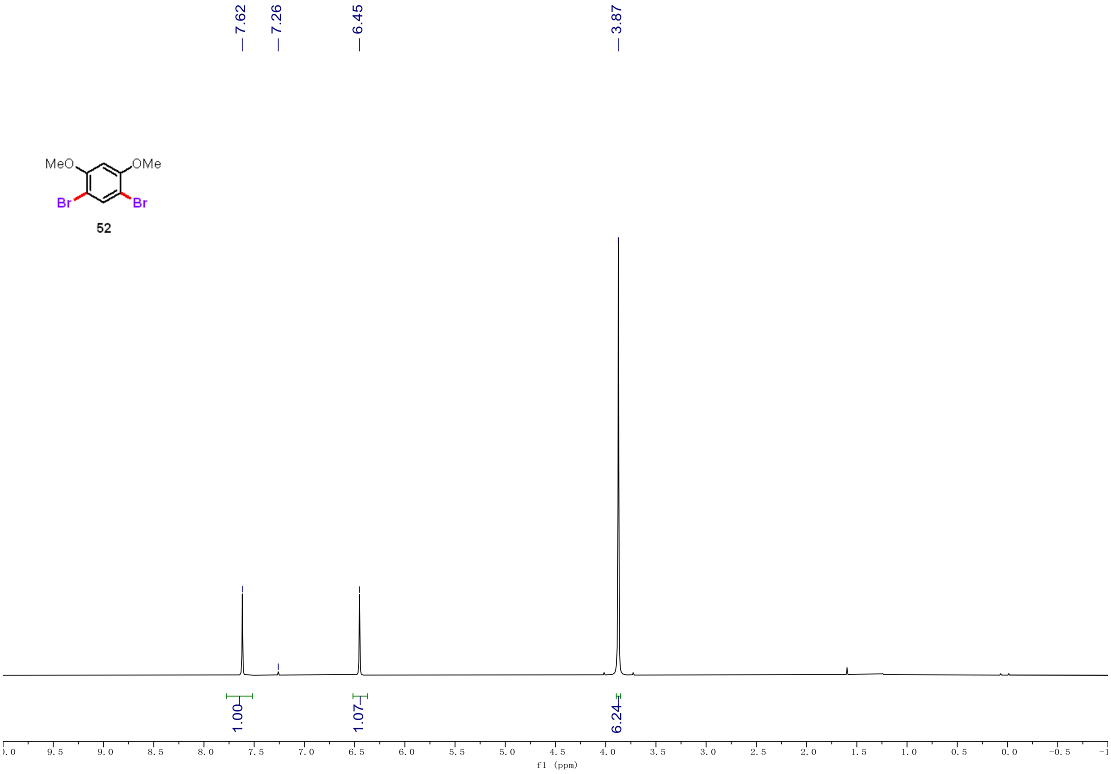

^{1}H NMR of compound **52**

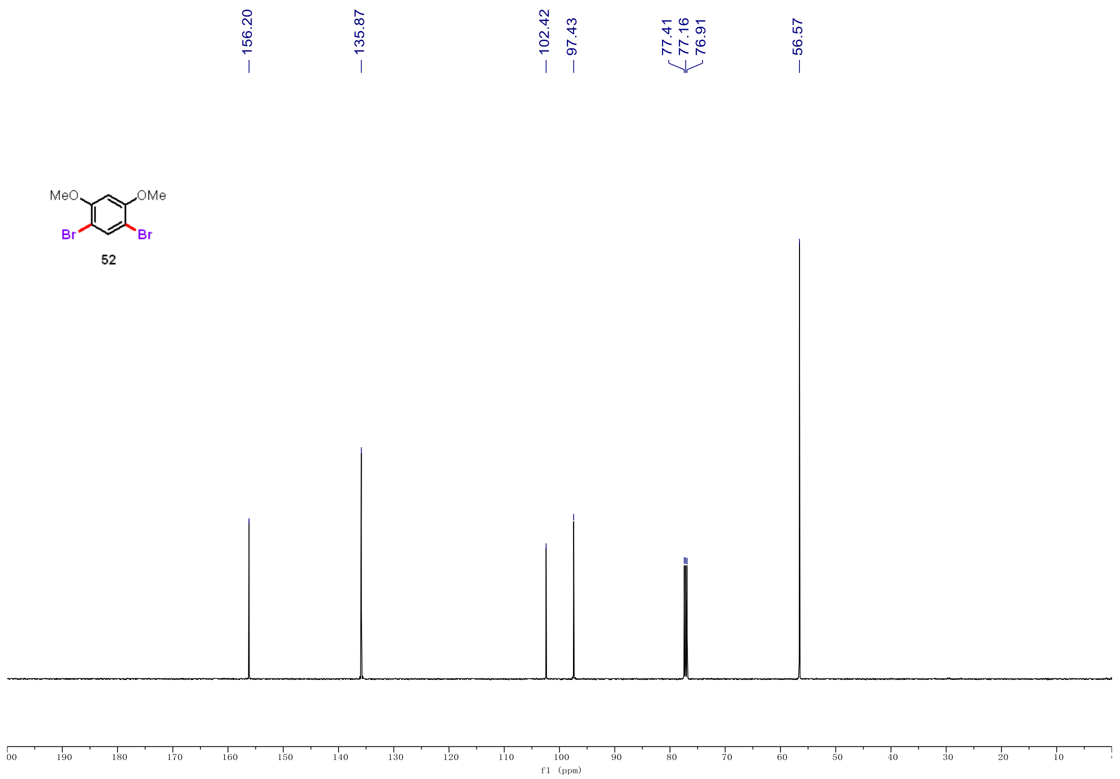

^{13}C NMR of compound **52**

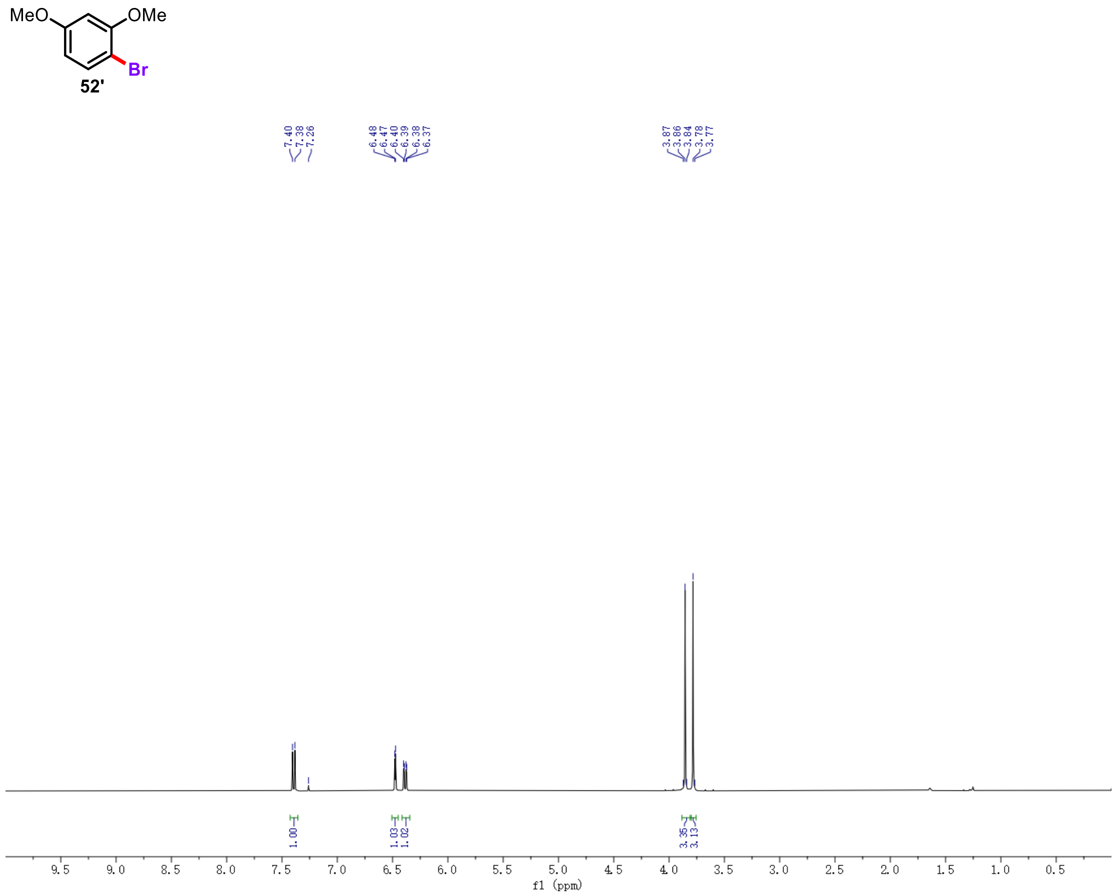

^{1}H NMR of compound **52’**

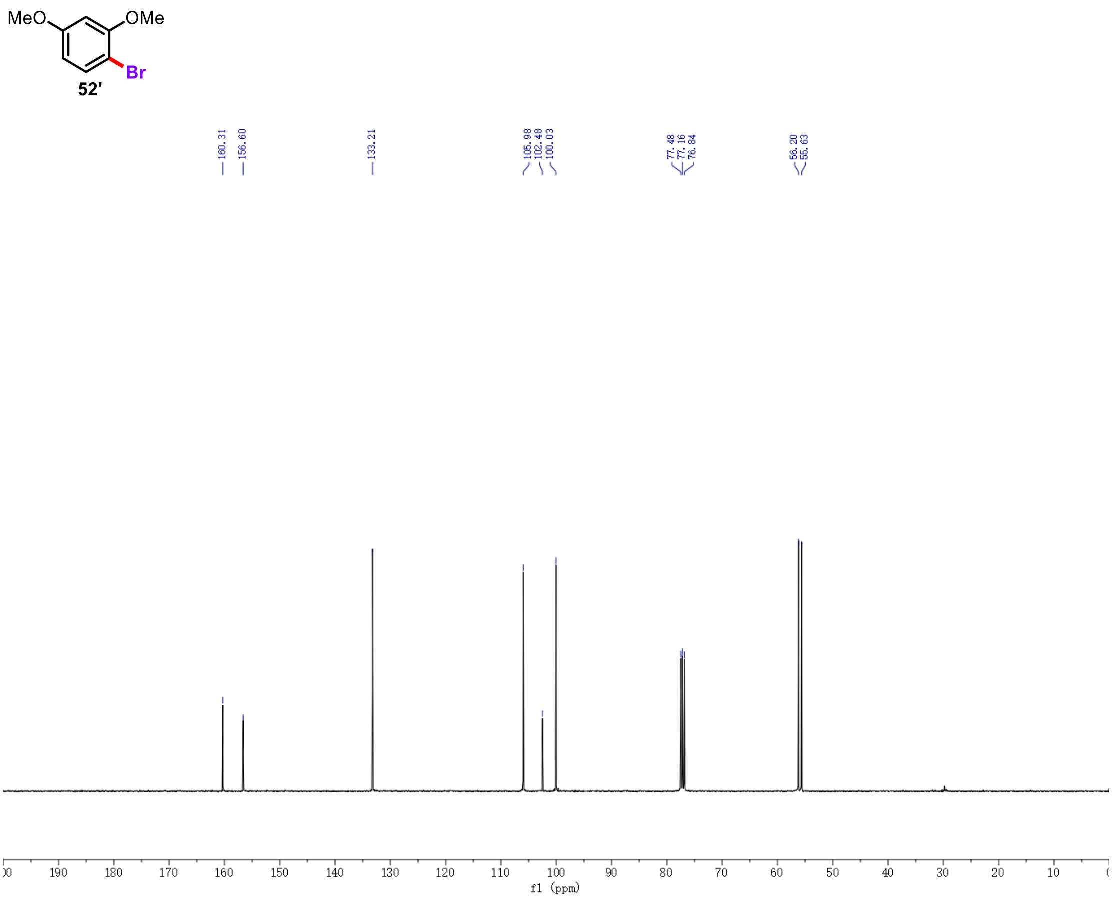

^{13}C NMR of compound **52’**

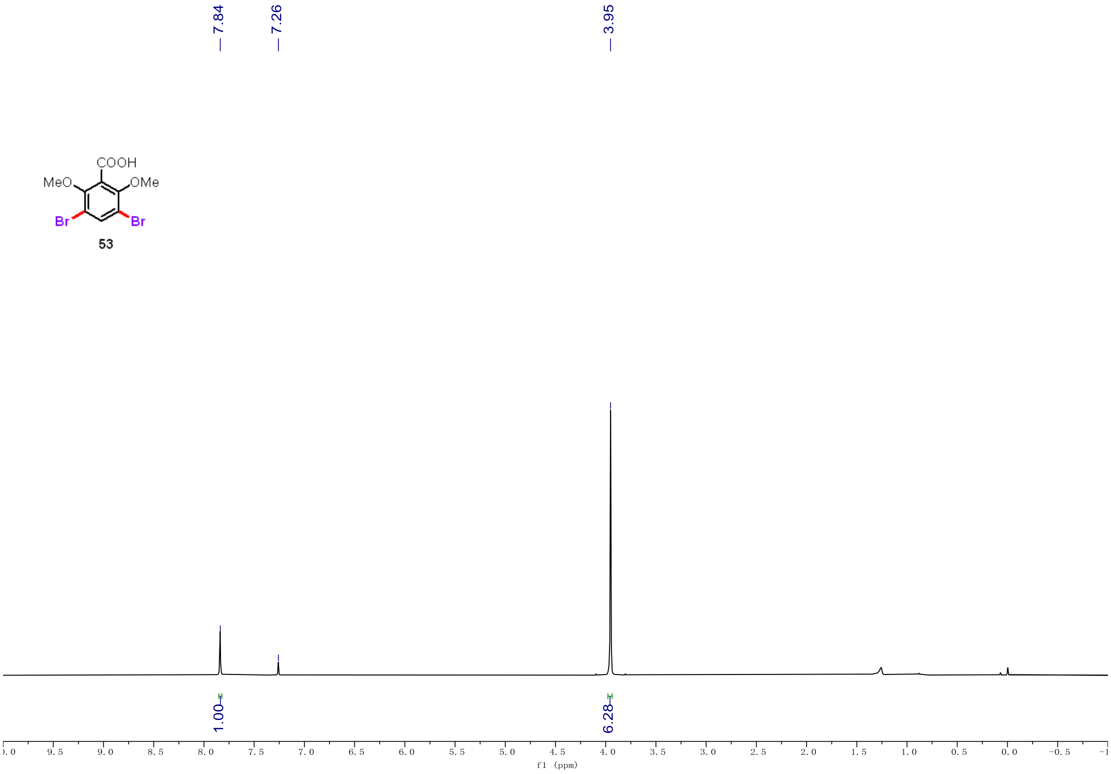

^{1}H NMR of compound **53**

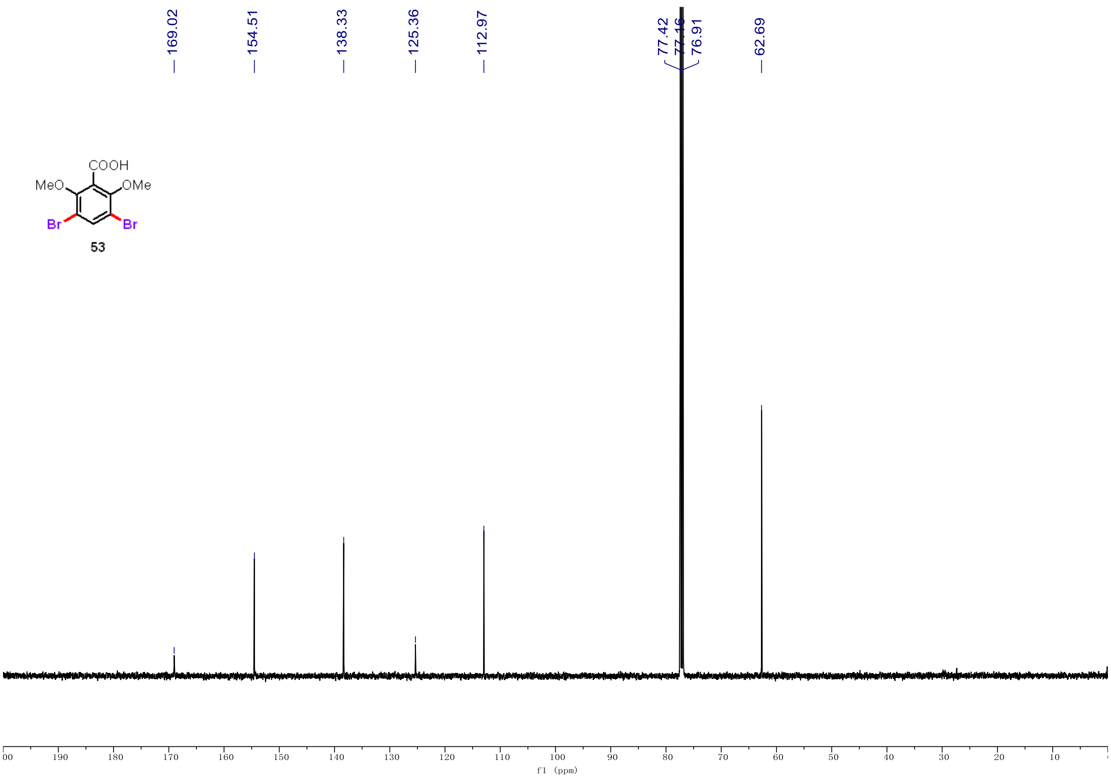

^{13}C NMR of compound **53**

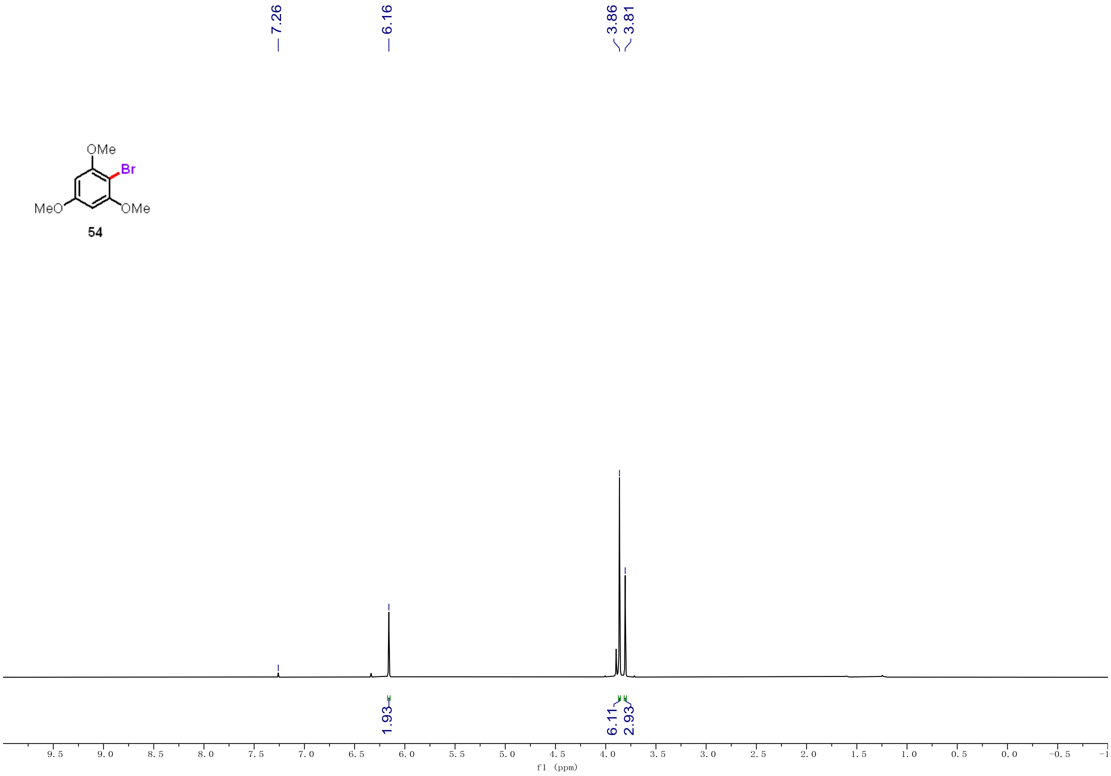

^{1}H NMR of compound **54**

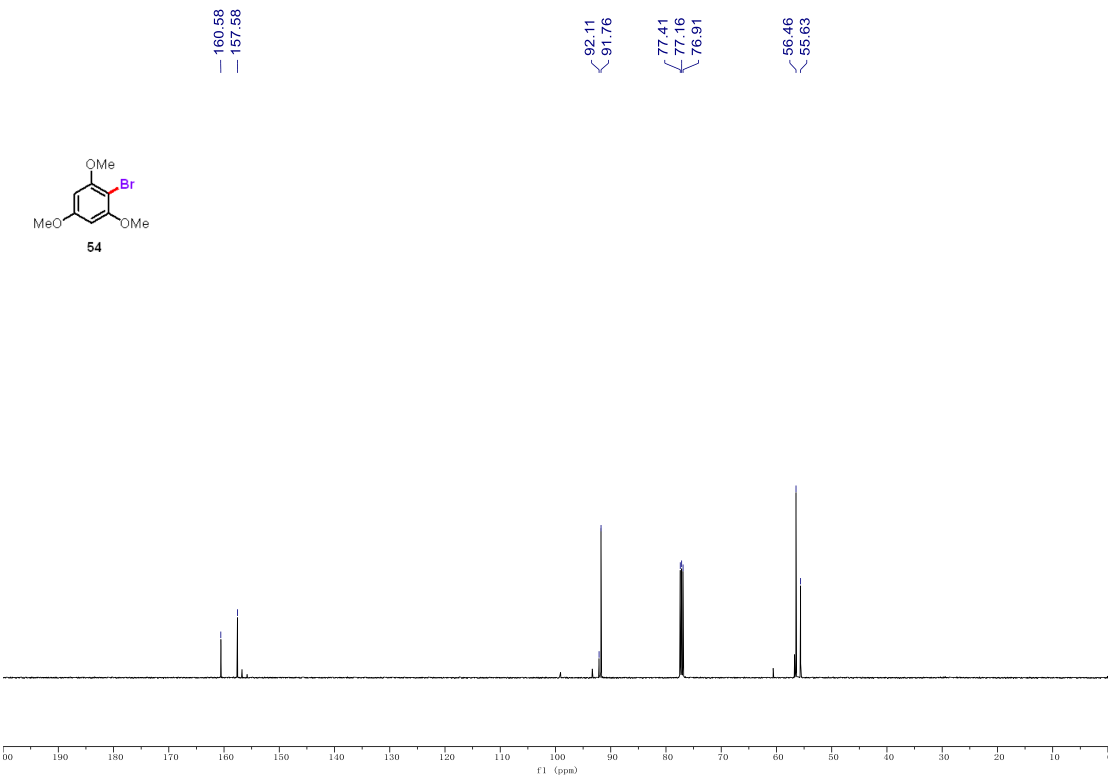

^{13}C NMR of compound **54**

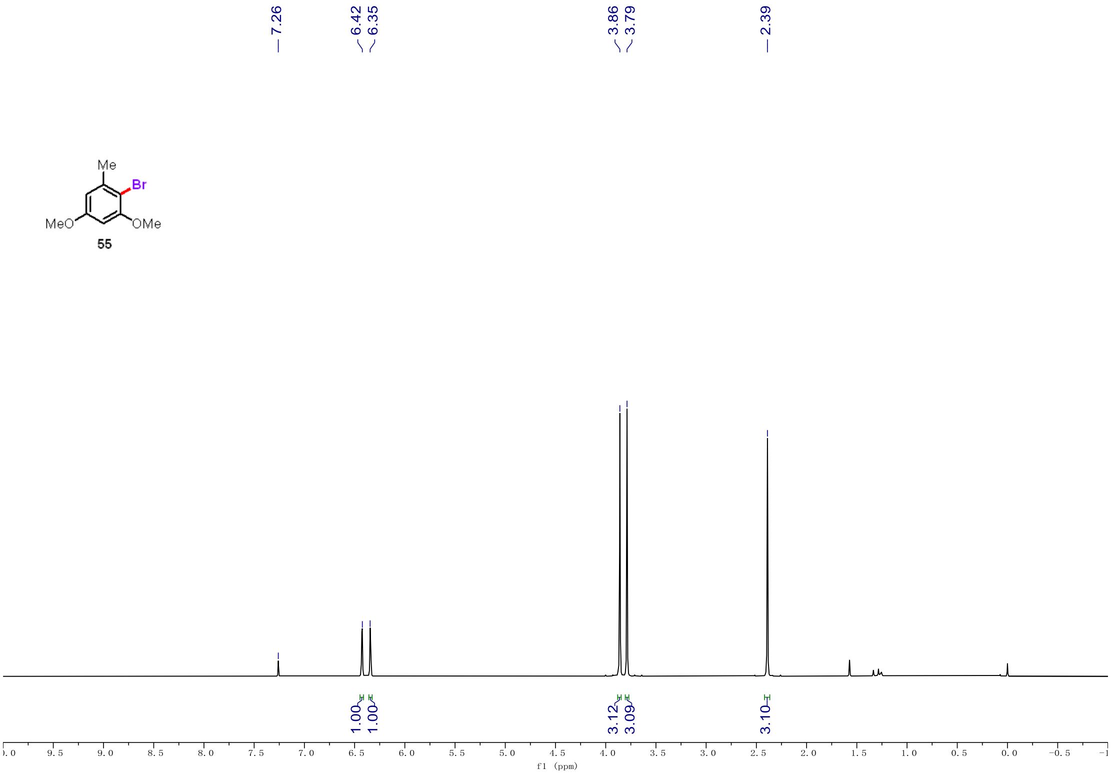

^{1}H NMR of compound **55**

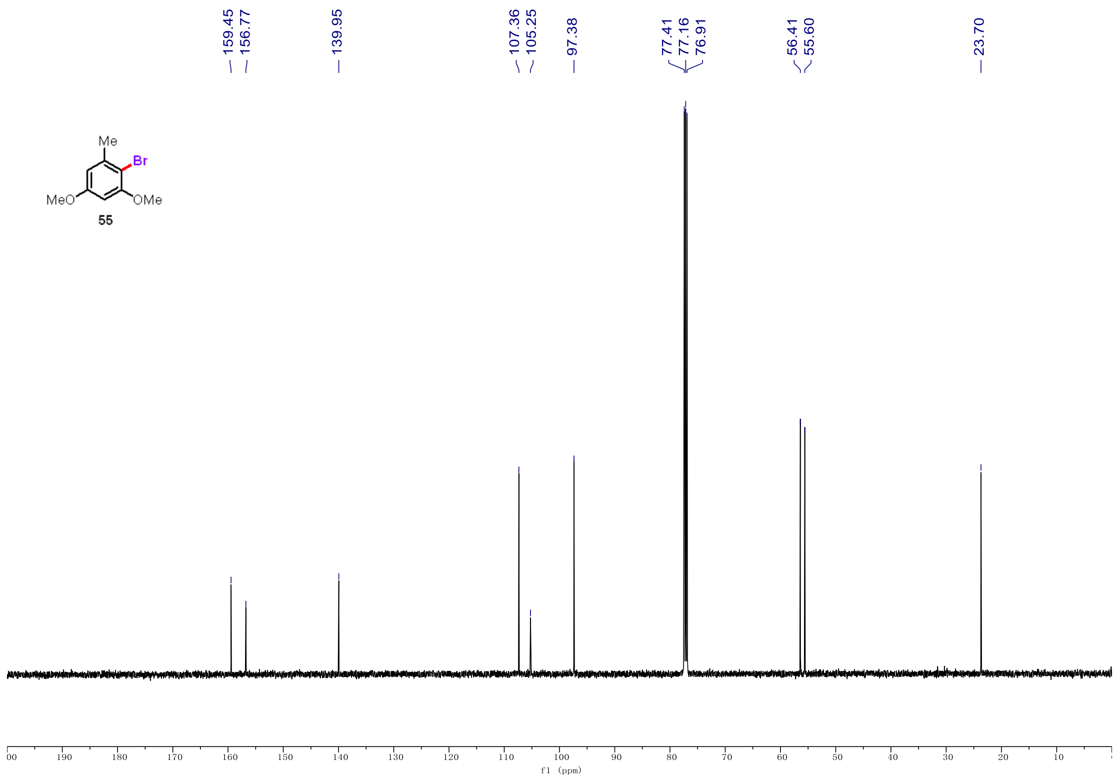

^{13}C NMR of compound **55**

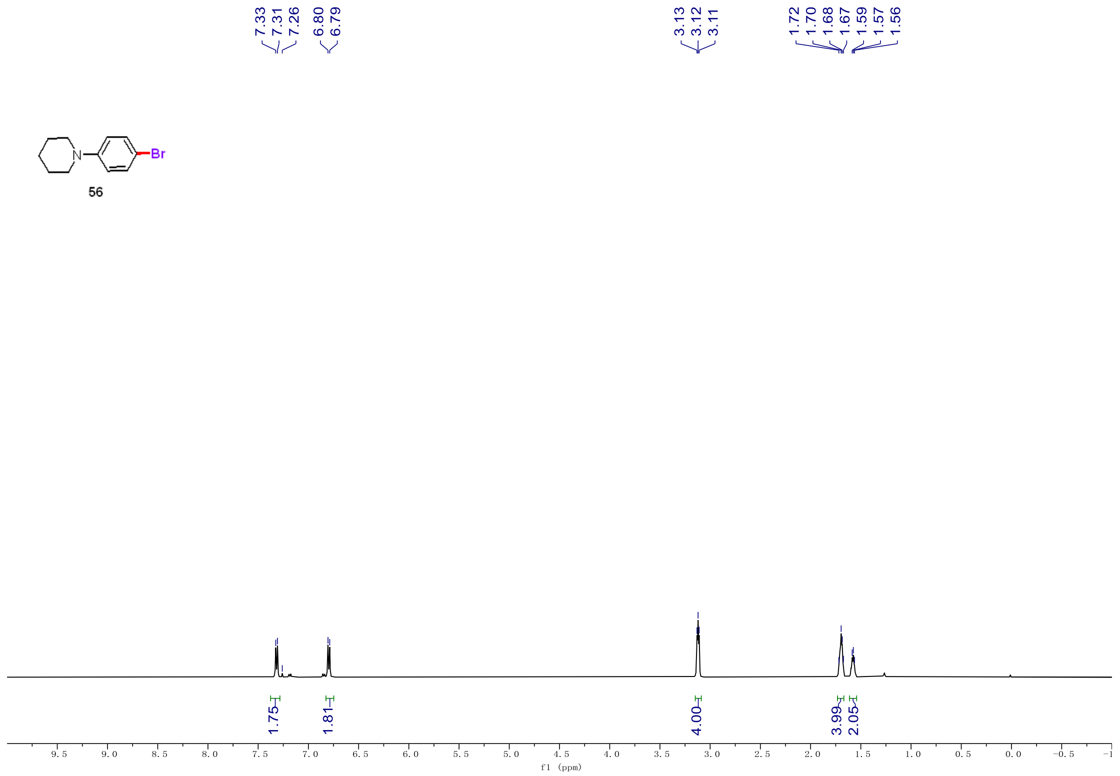

^{1}H NMR of compound **56**

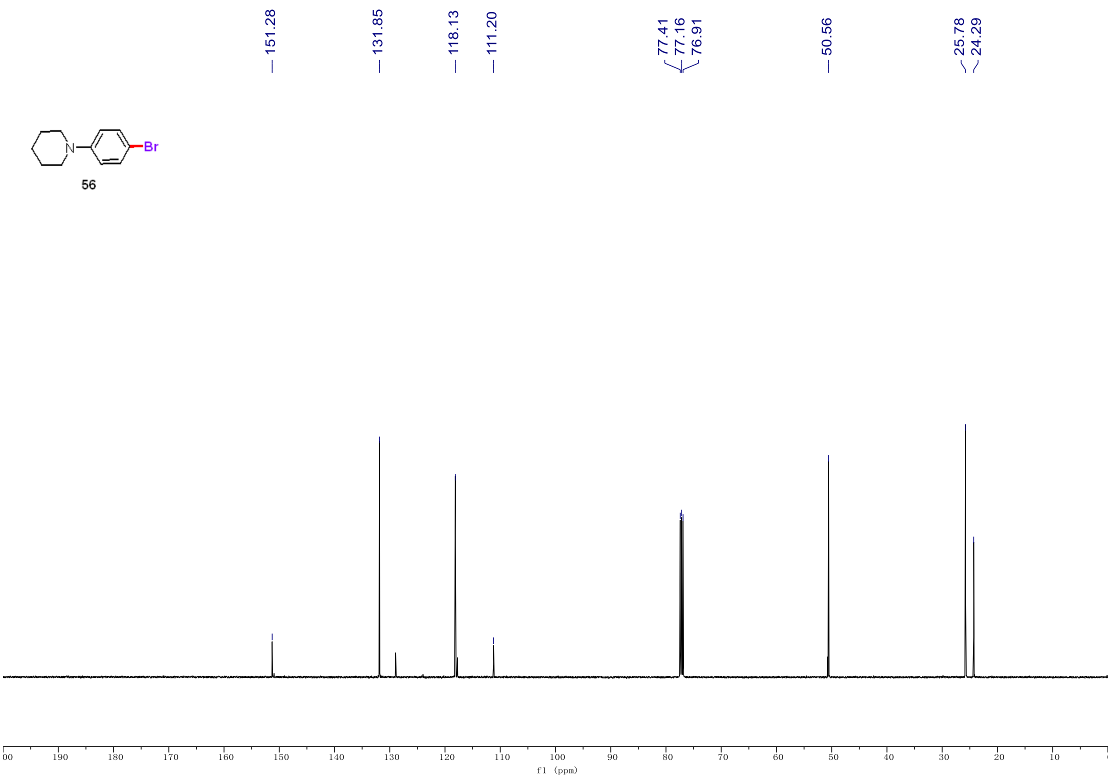

^{13}C NMR of compound **56**

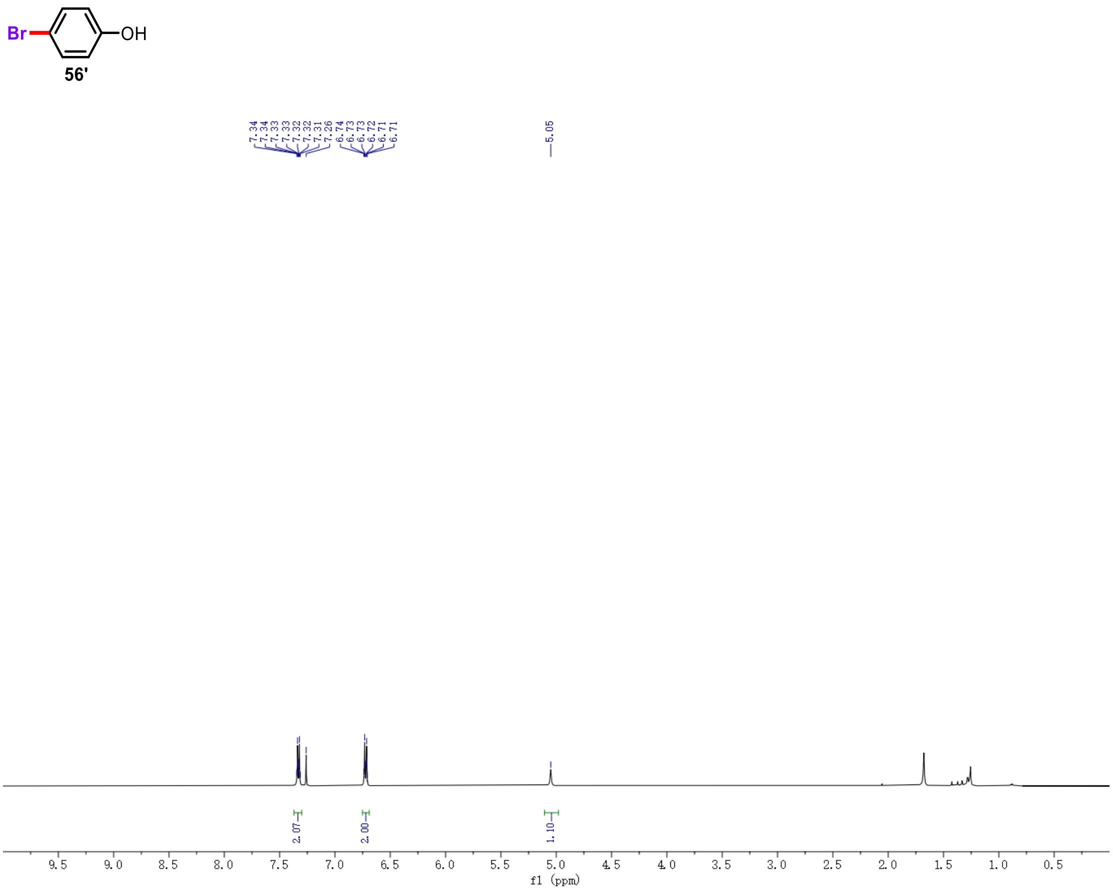

^{1}H NMR of compound **56’**

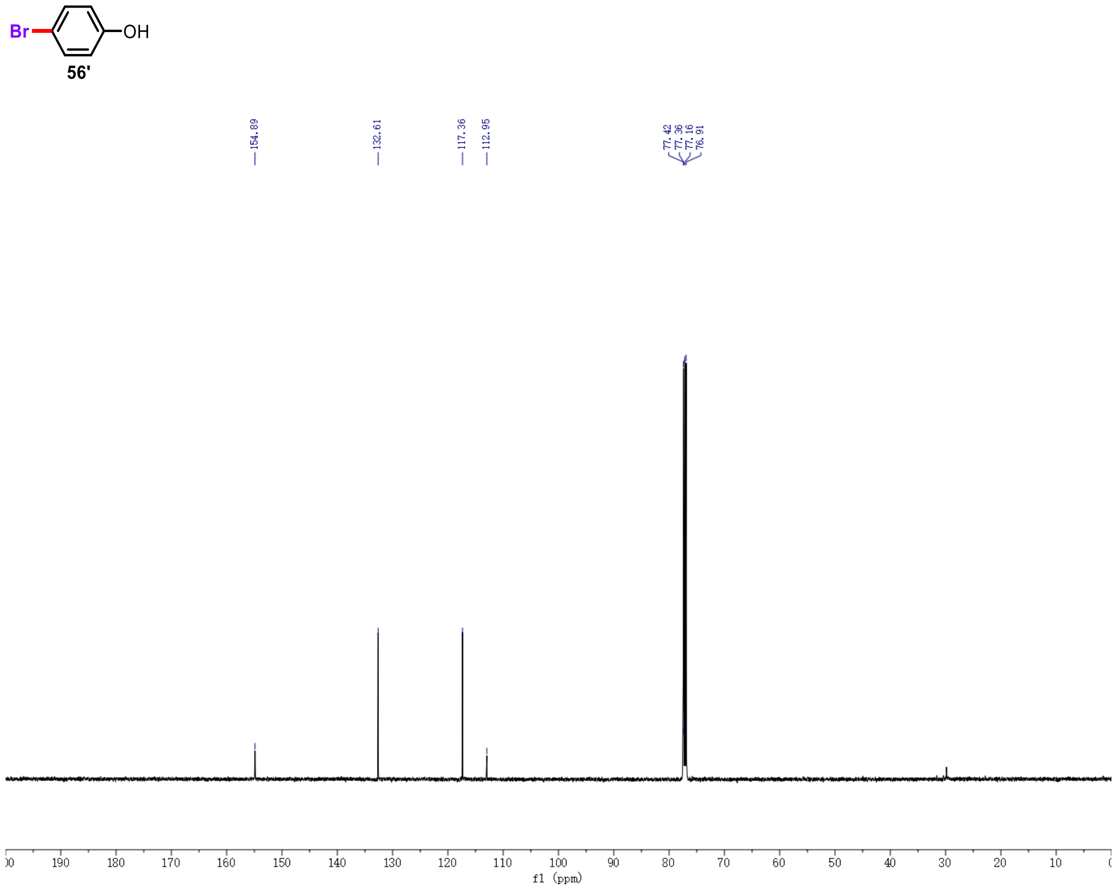

^{13}C NMR of compound **56’**

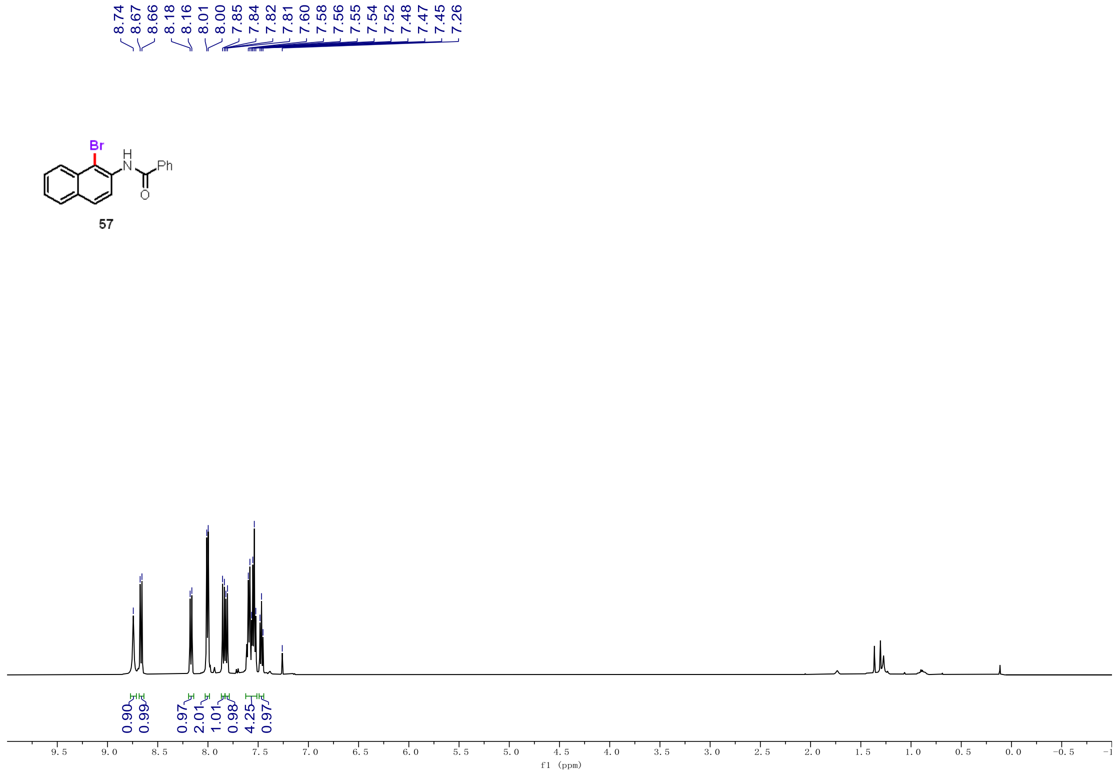

^{1}H NMR of compound **57**

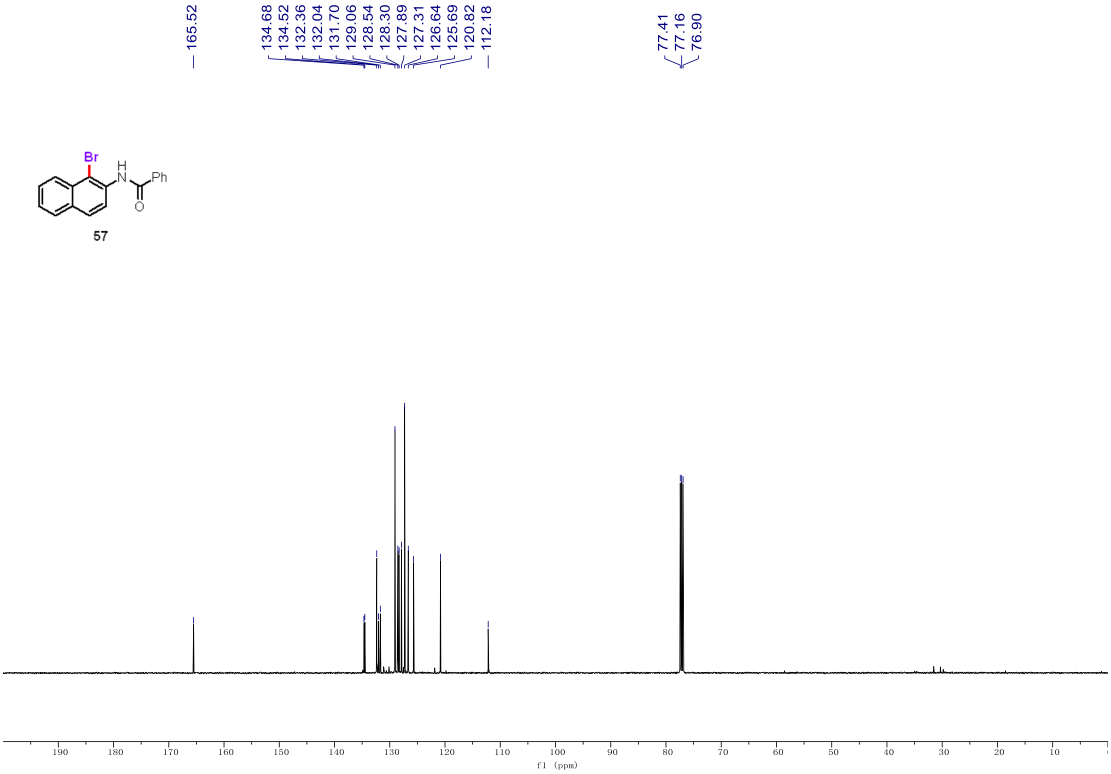

^{13}C NMR of compound **57**

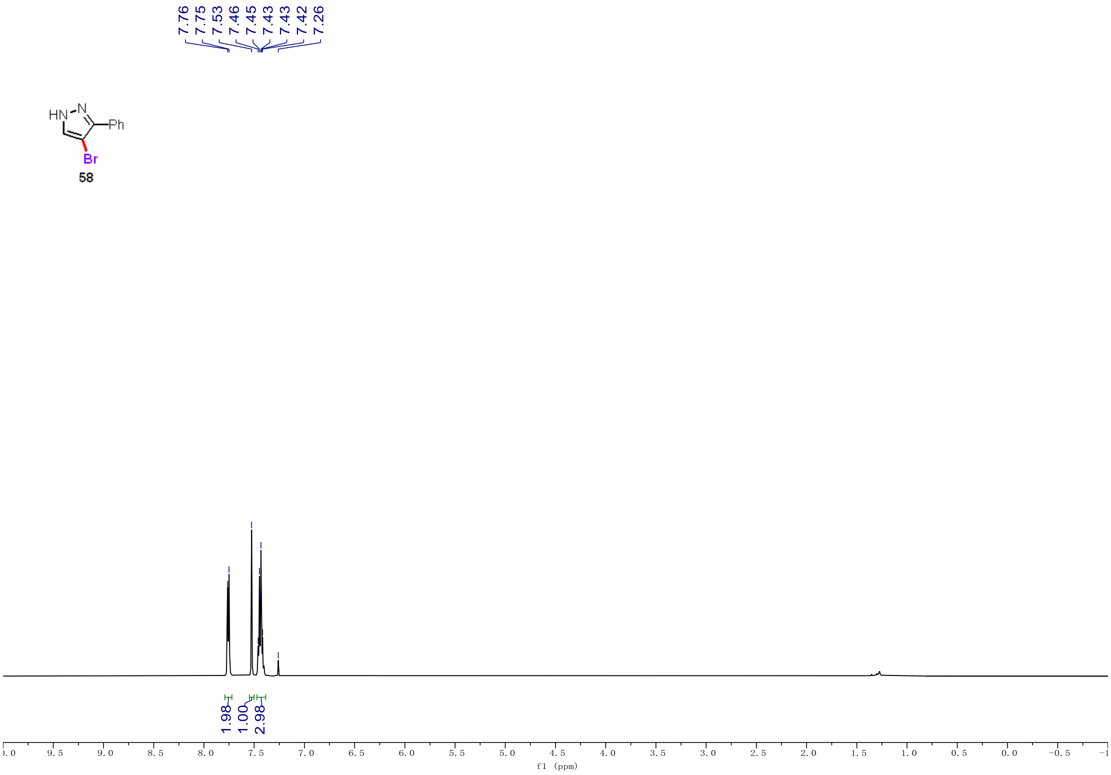

^{1}H NMR of compound **58**

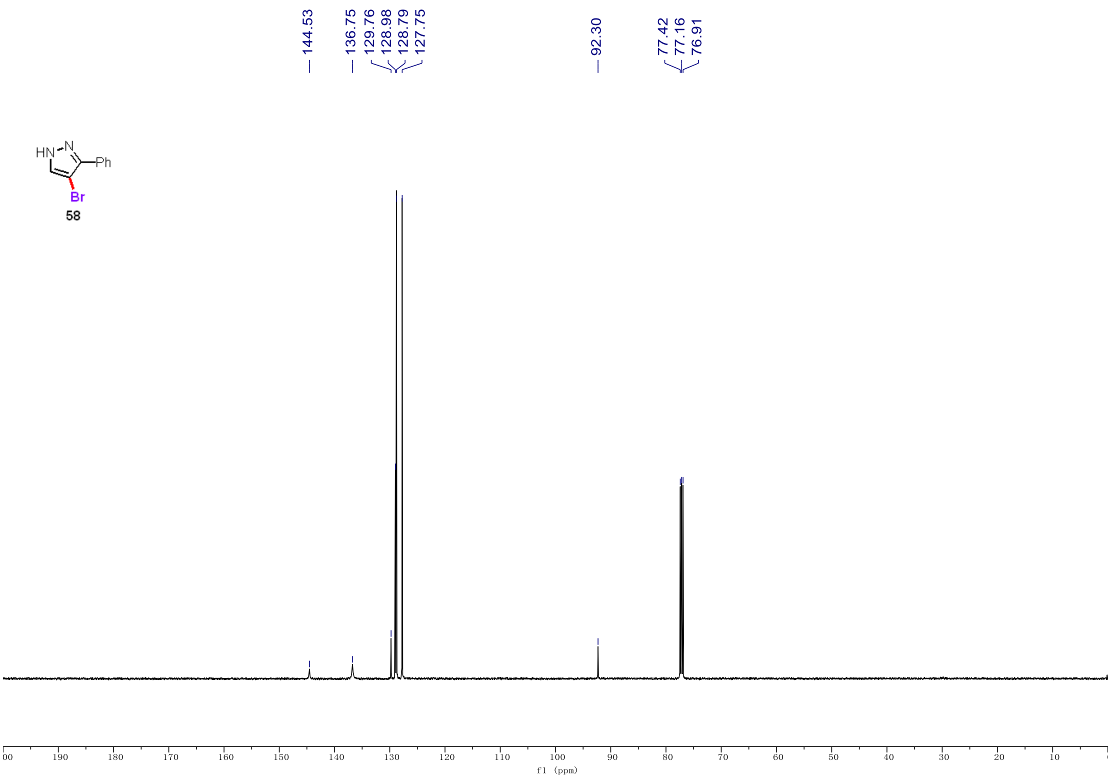

^{13}C NMR of compound **58**

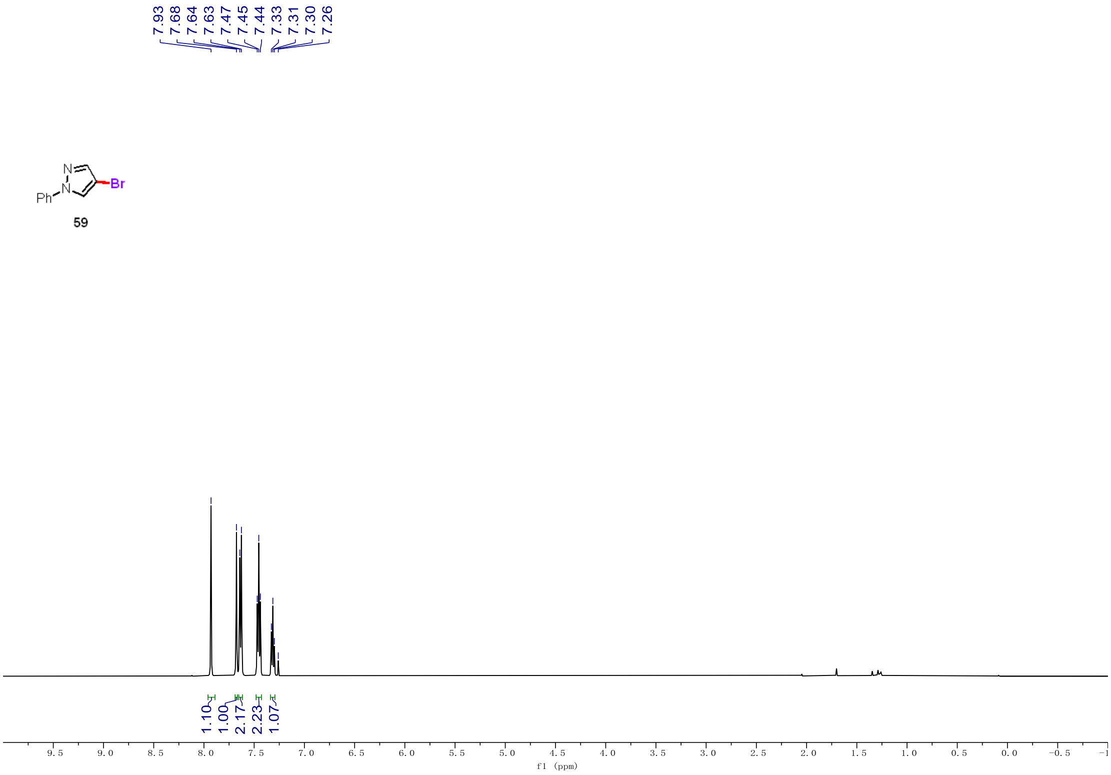

^{1}H NMR of compound **59**

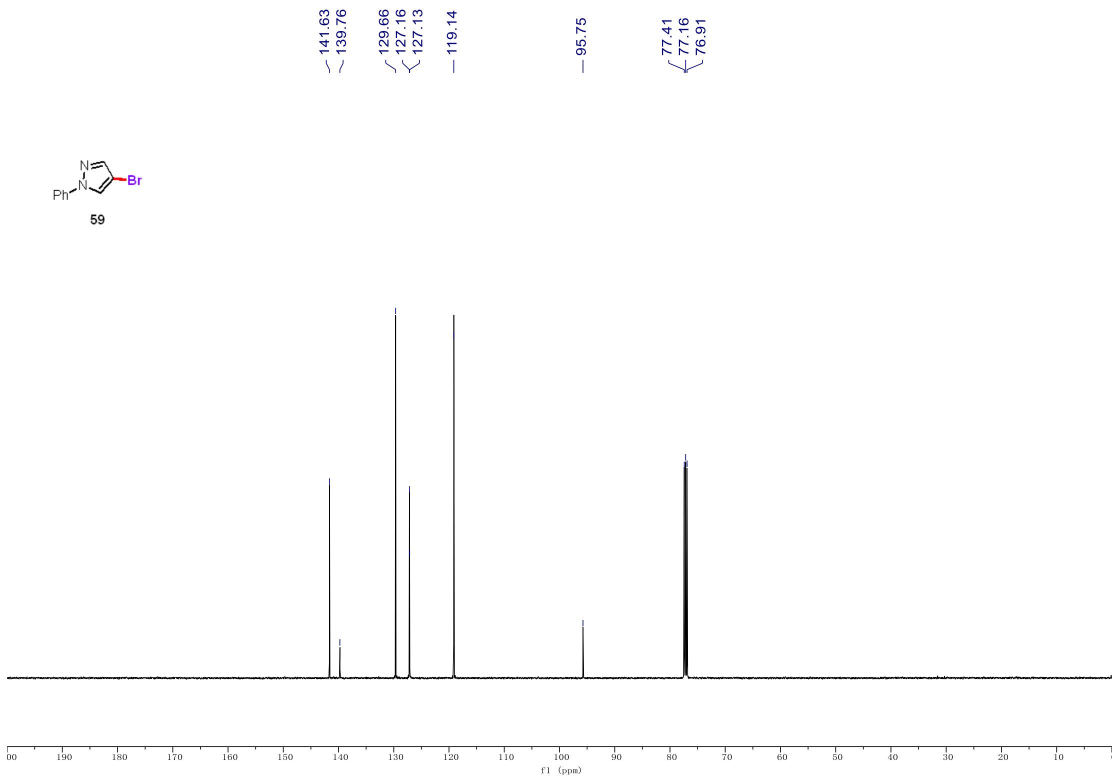

^{13}C NMR of compound **59**

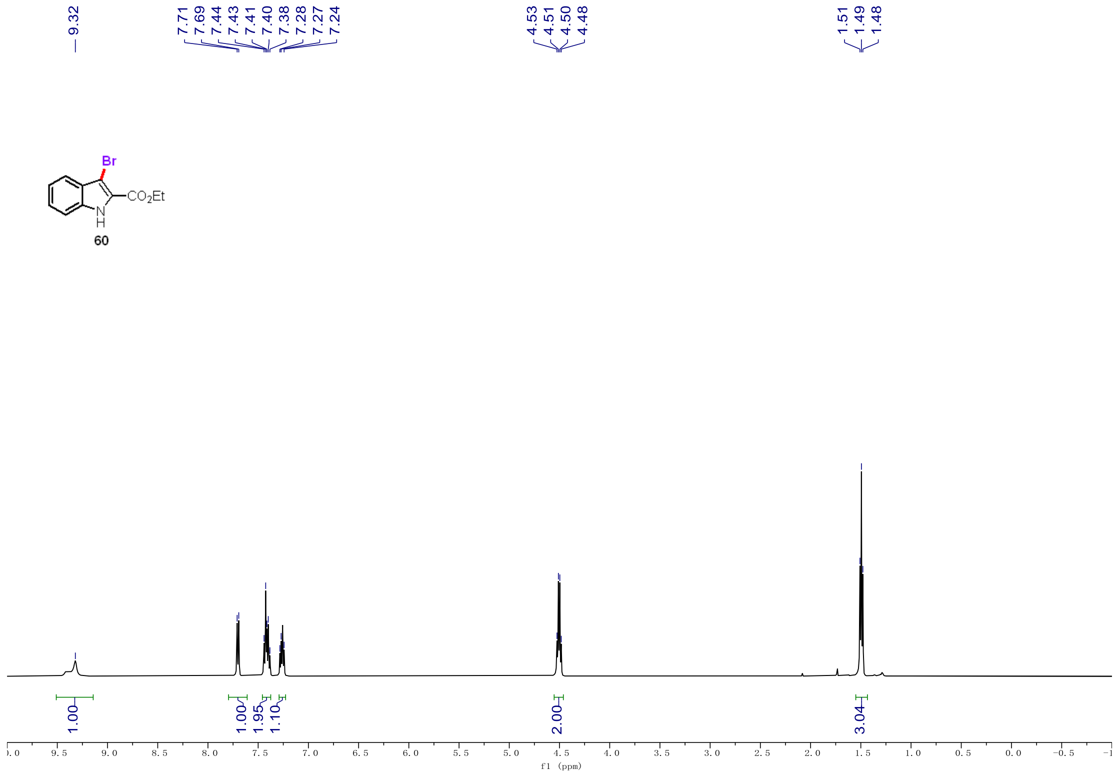

^{1}H NMR of compound **60**

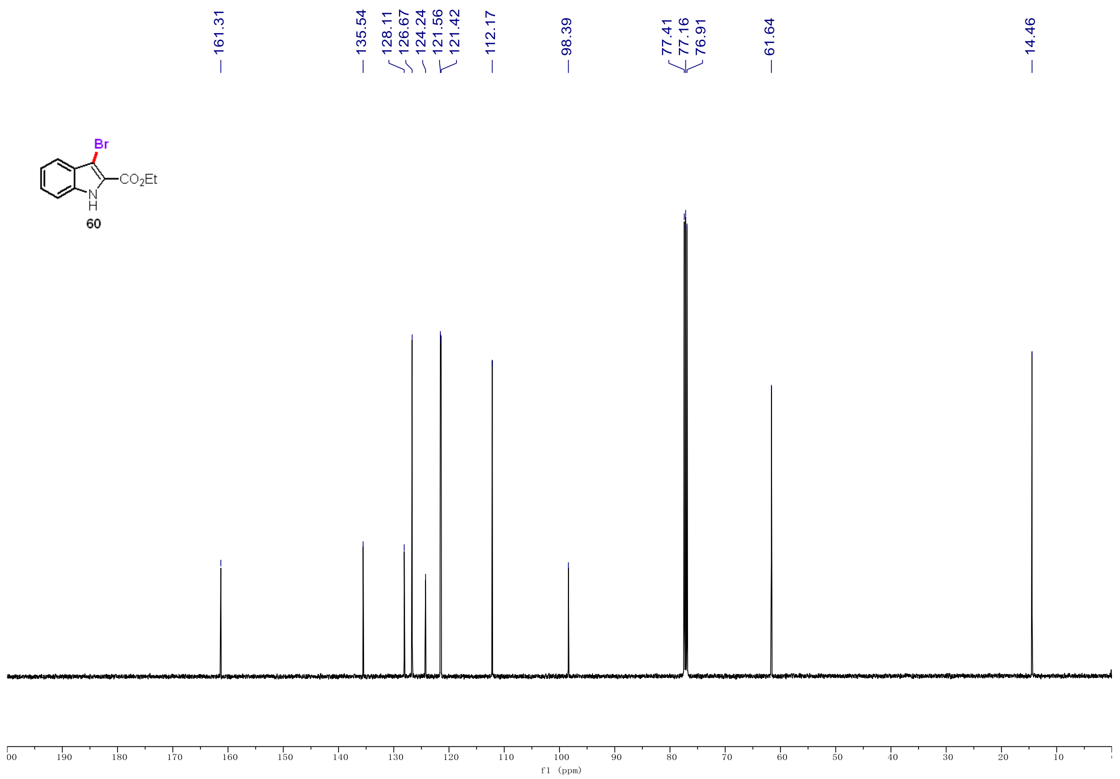

^{13}C NMR of compound **60**

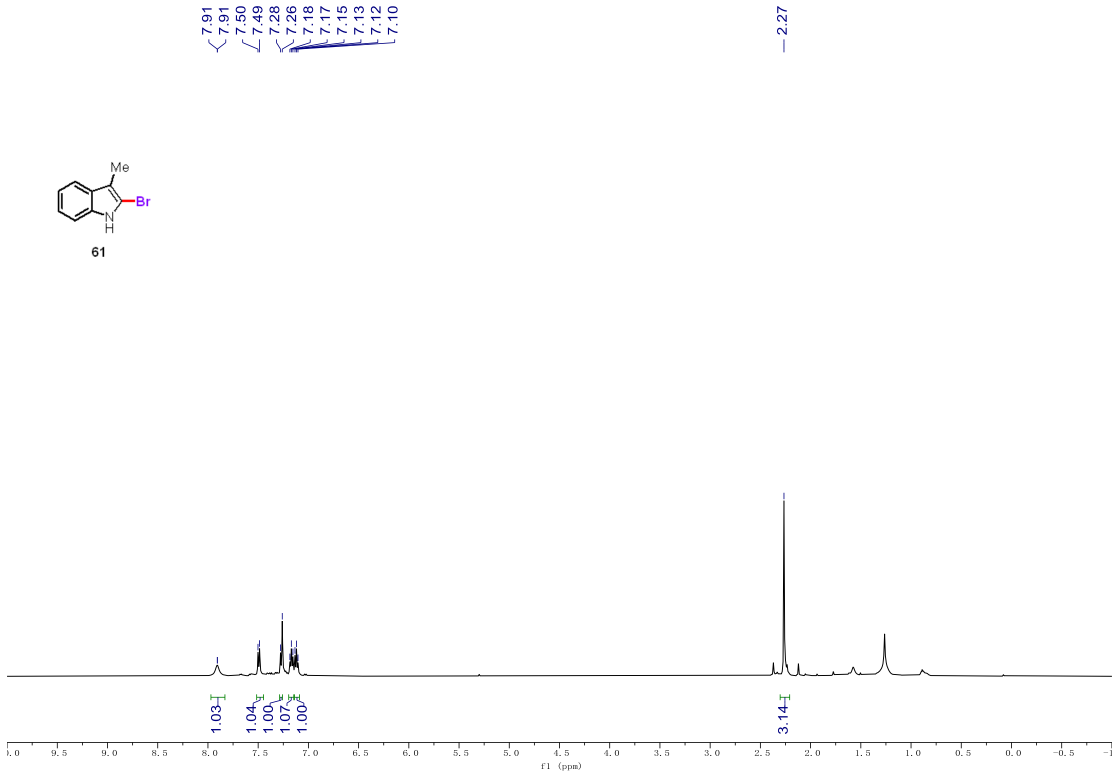

^{1}H NMR of compound **61**

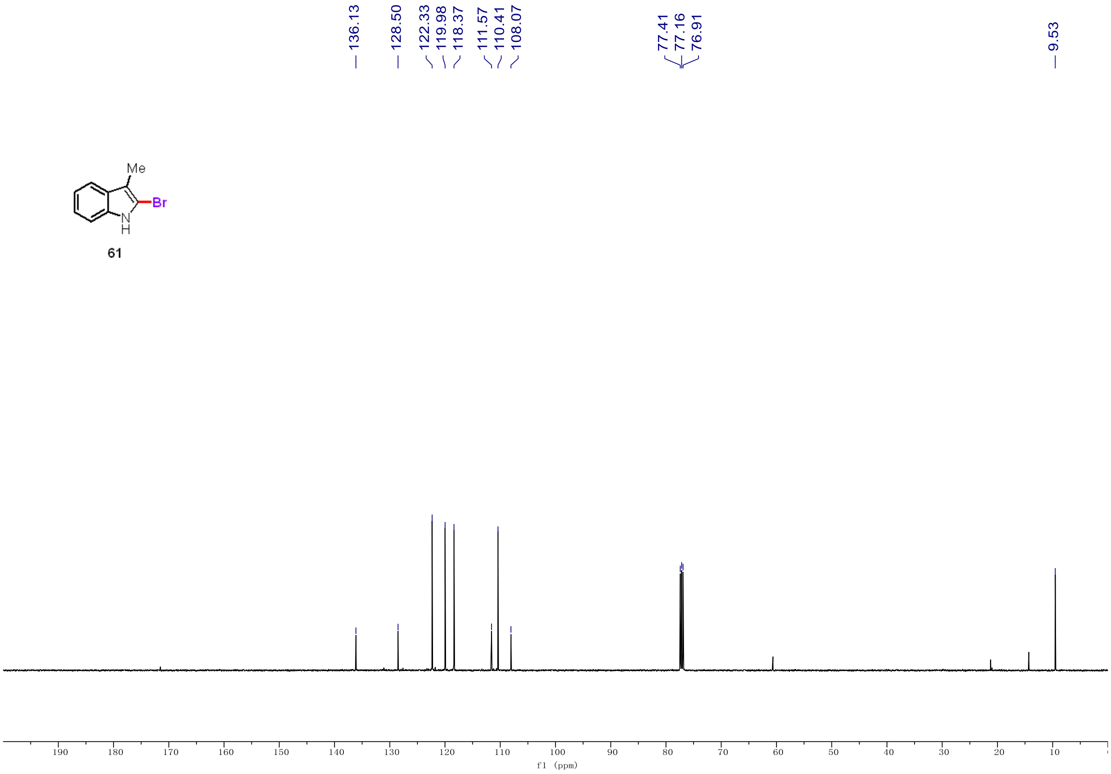

^{13}C NMR of compound **61**

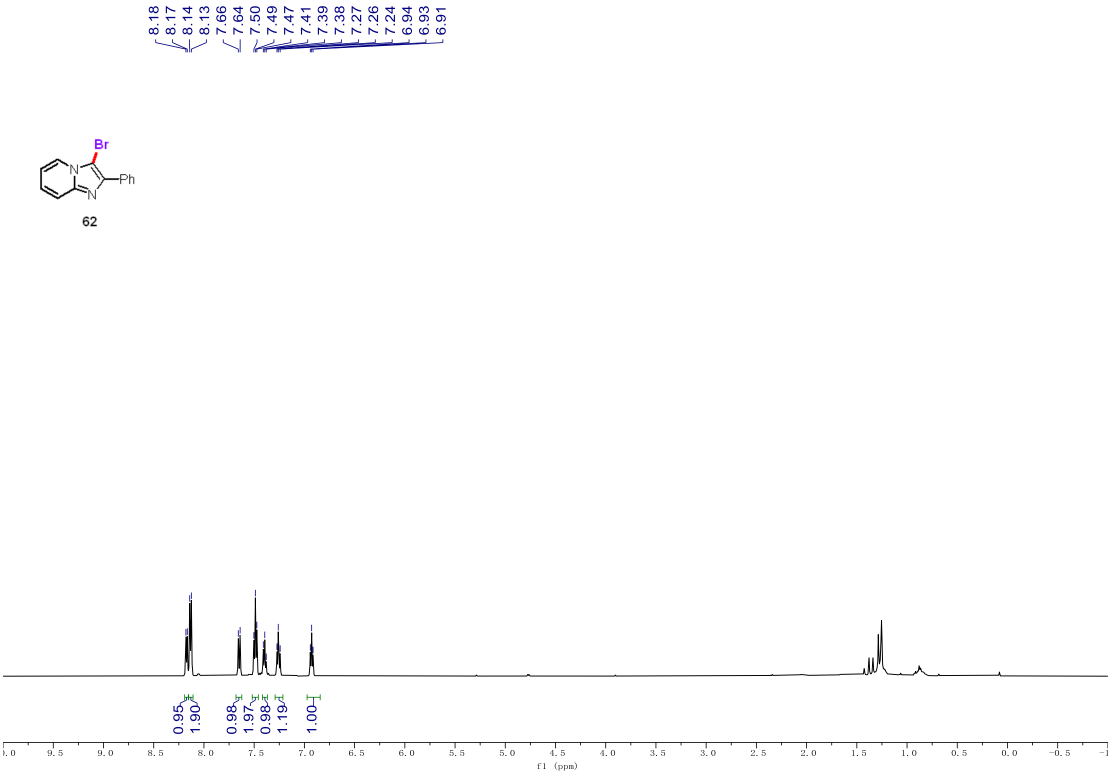

^{1}H NMR of compound **62**

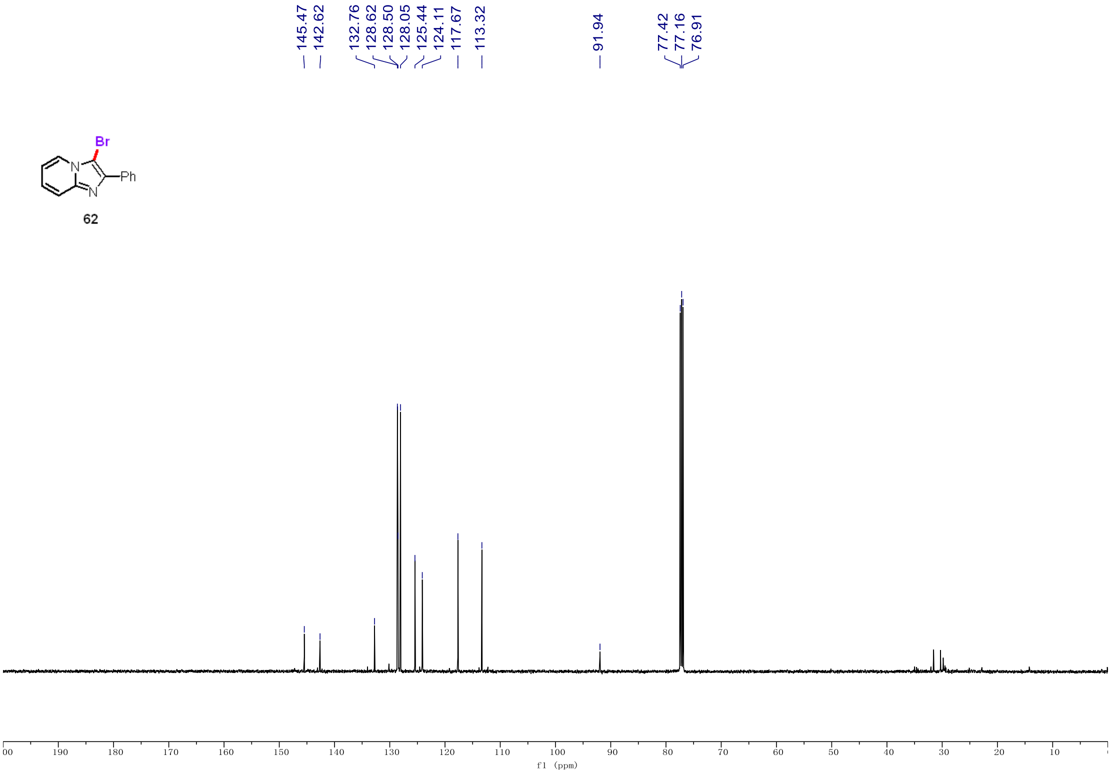

^{13}C NMR of compound **62**

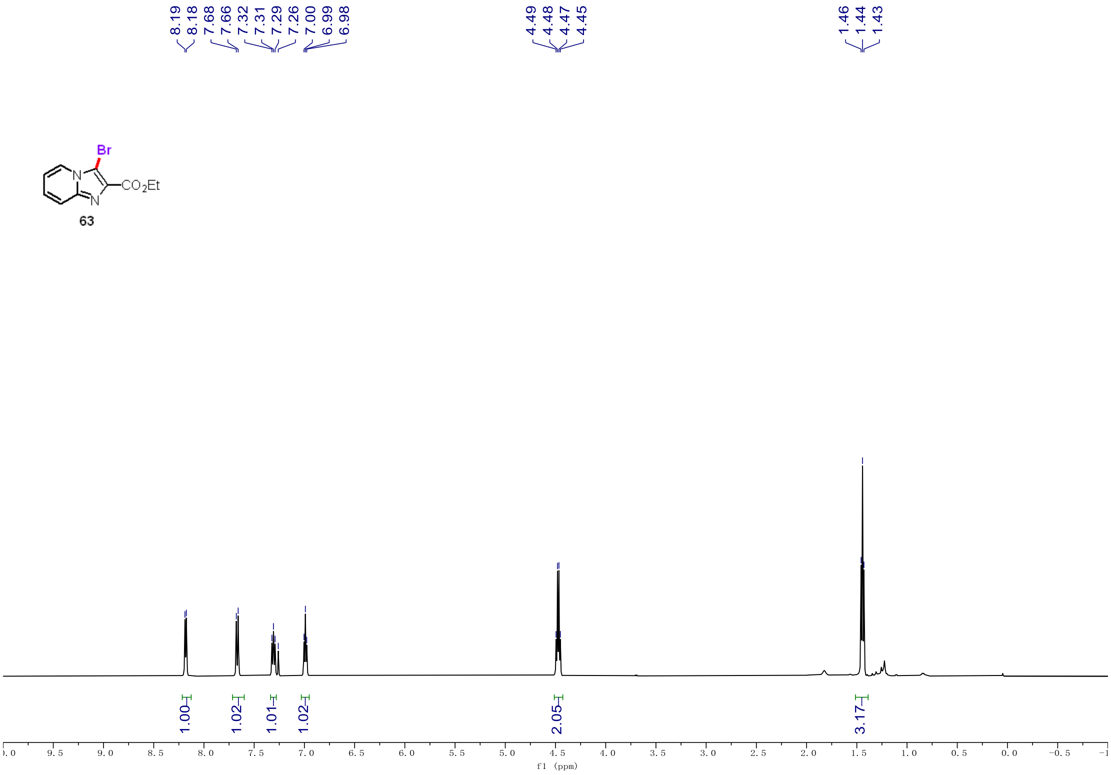

^{1}H NMR of compound **63**

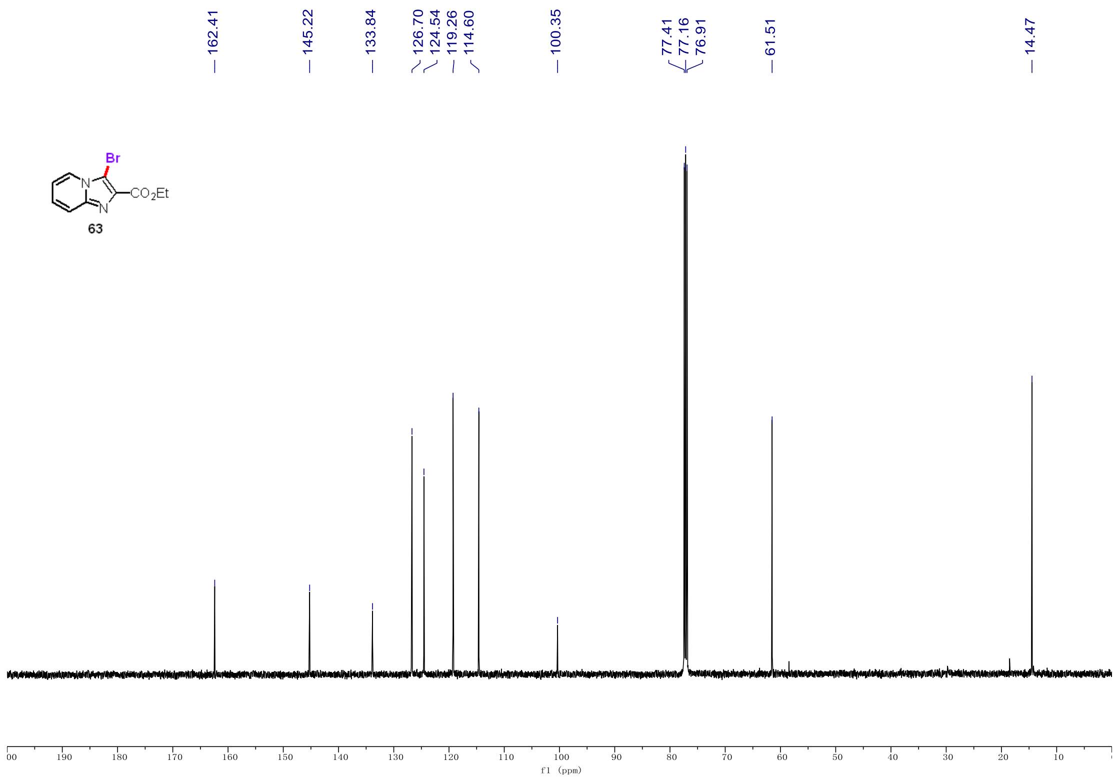

^{13}C NMR of compound **63**

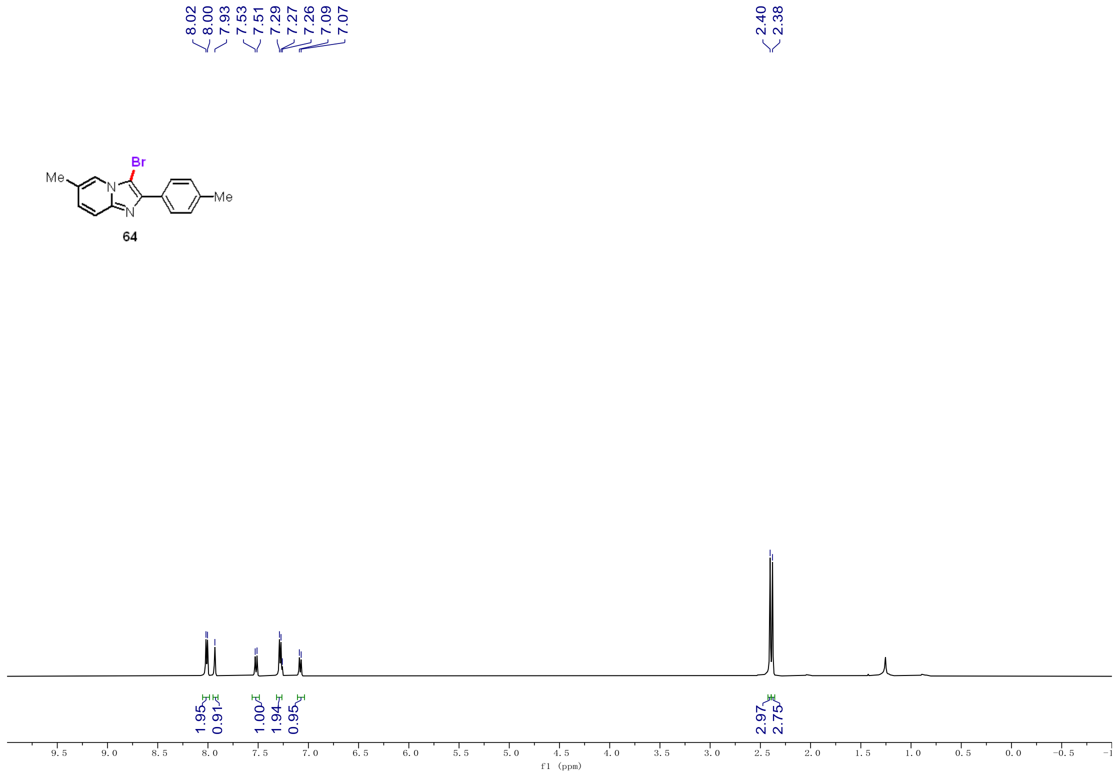

^{1}H NMR of compound **64**

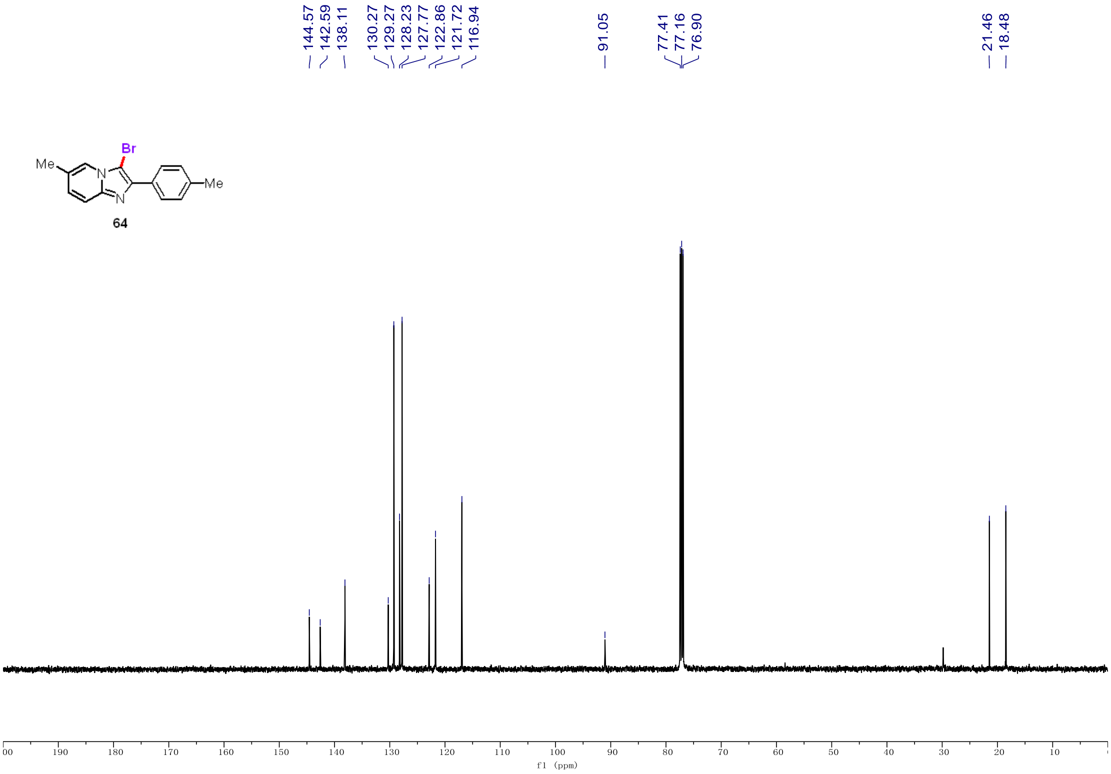

^{13}C NMR of compound **64**

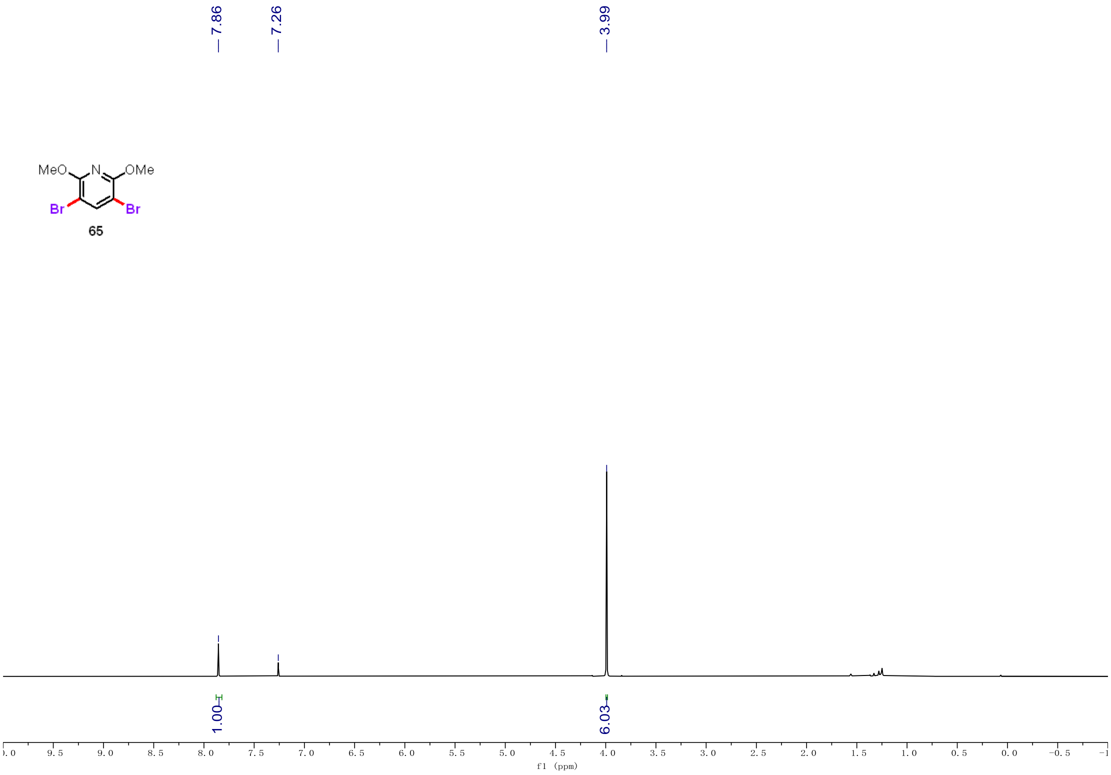

^{1}H NMR of compound **65**

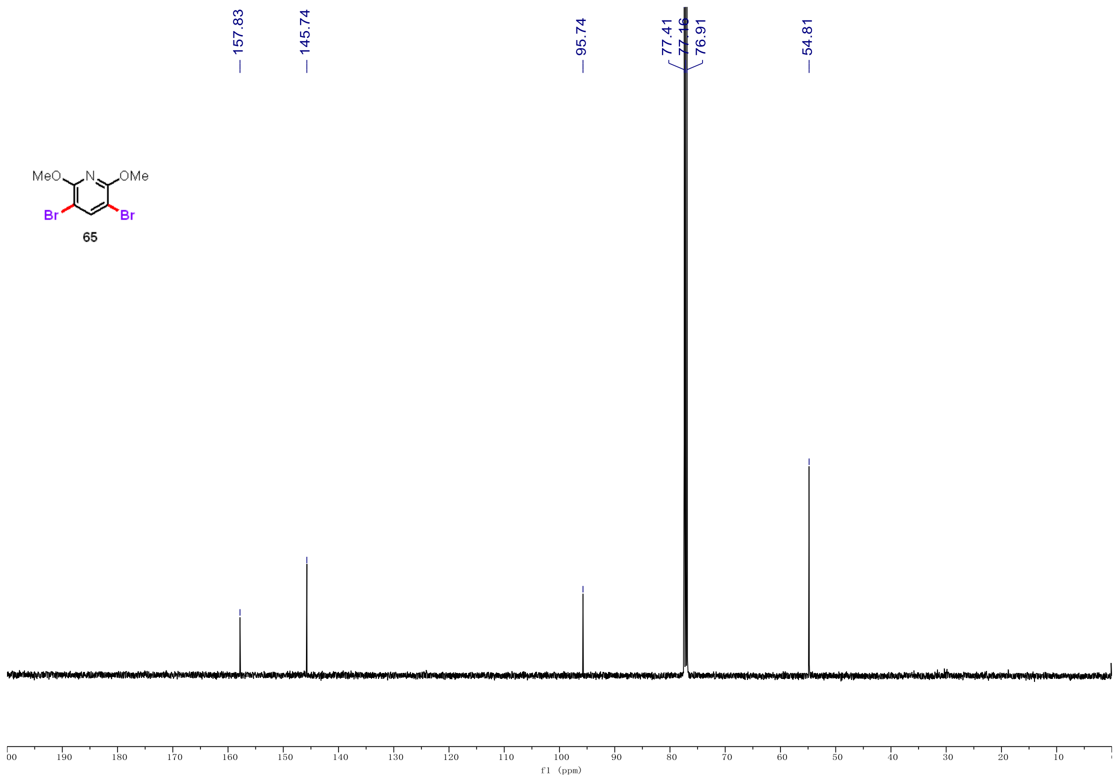

^{13}C NMR of compound **65**

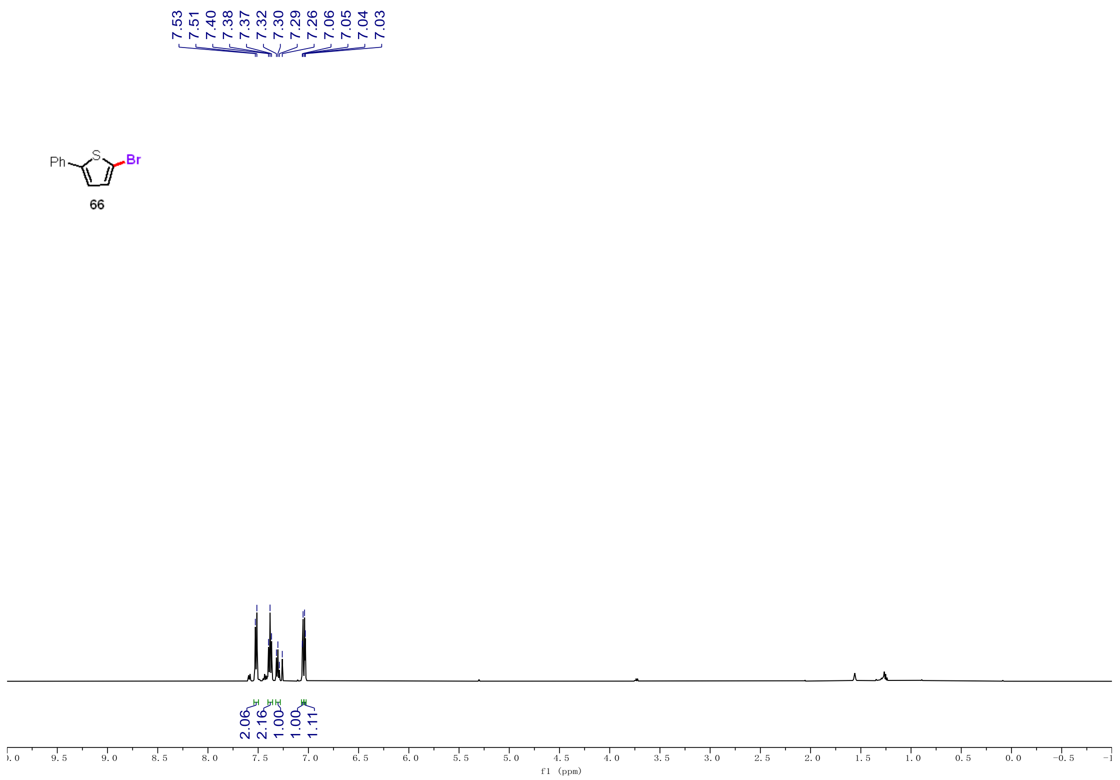

^{1}H NMR of compound **66**

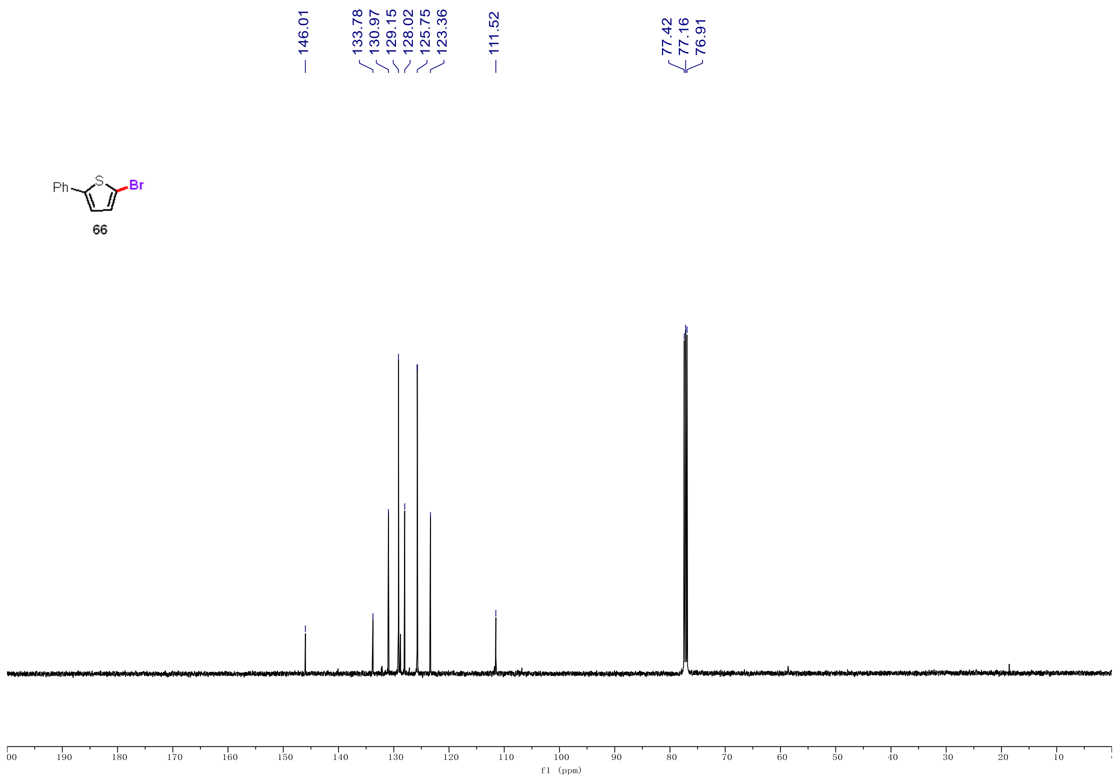

^{13}C NMR of compound **66**

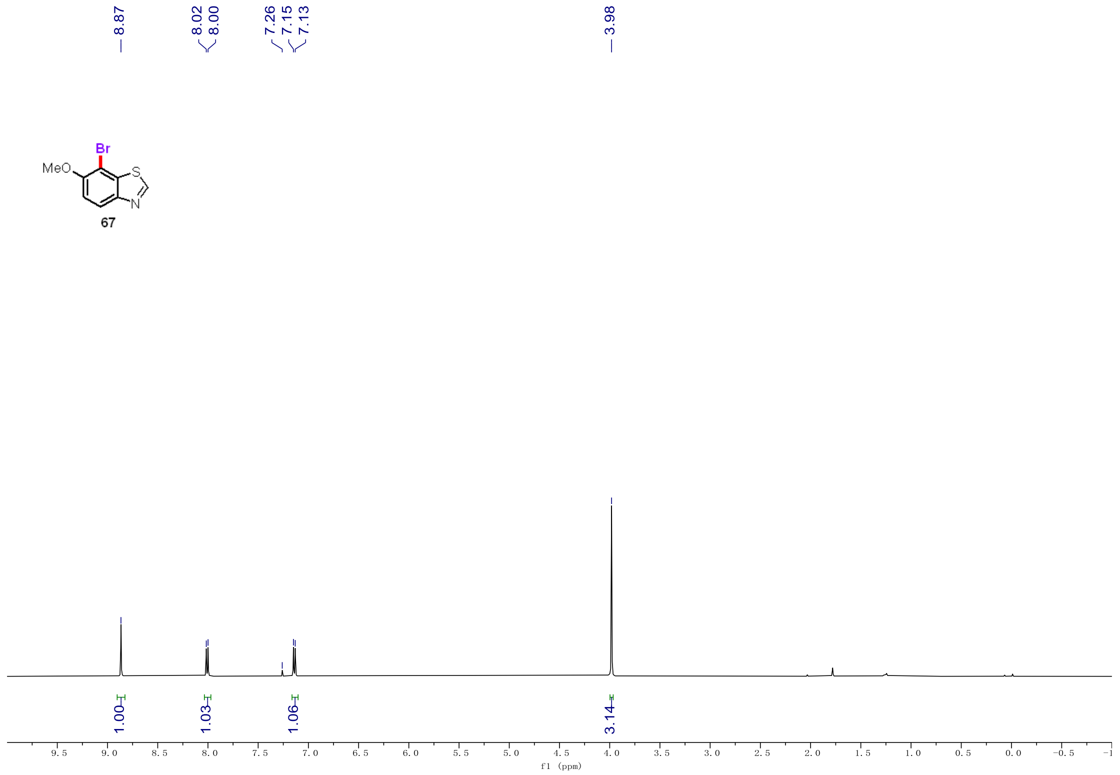

^{1}H NMR of compound **67**

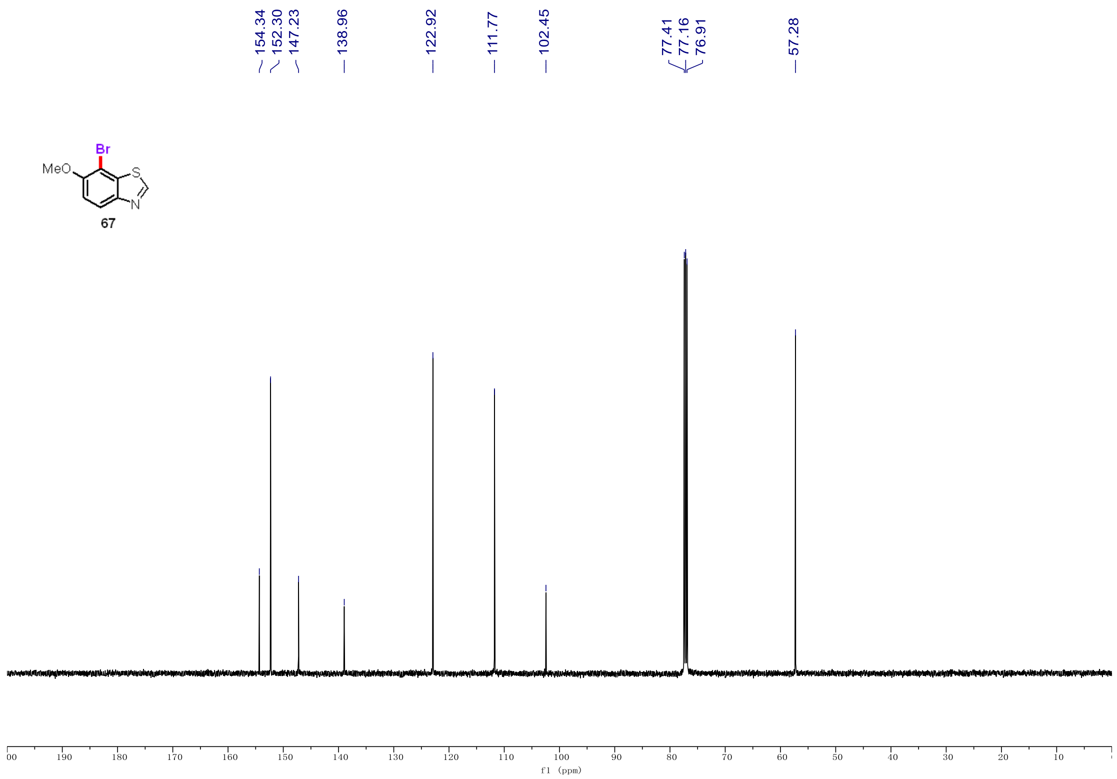

^{13}C NMR of compound **67**

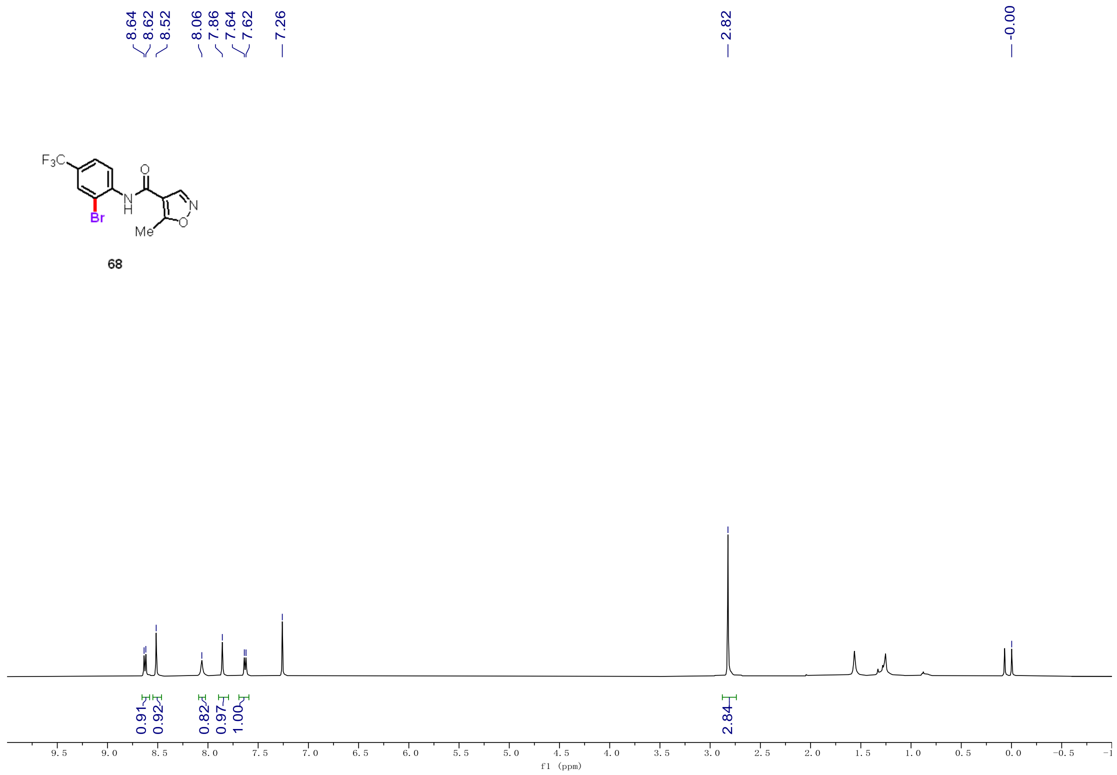

^{1}H NMR of compound **68**

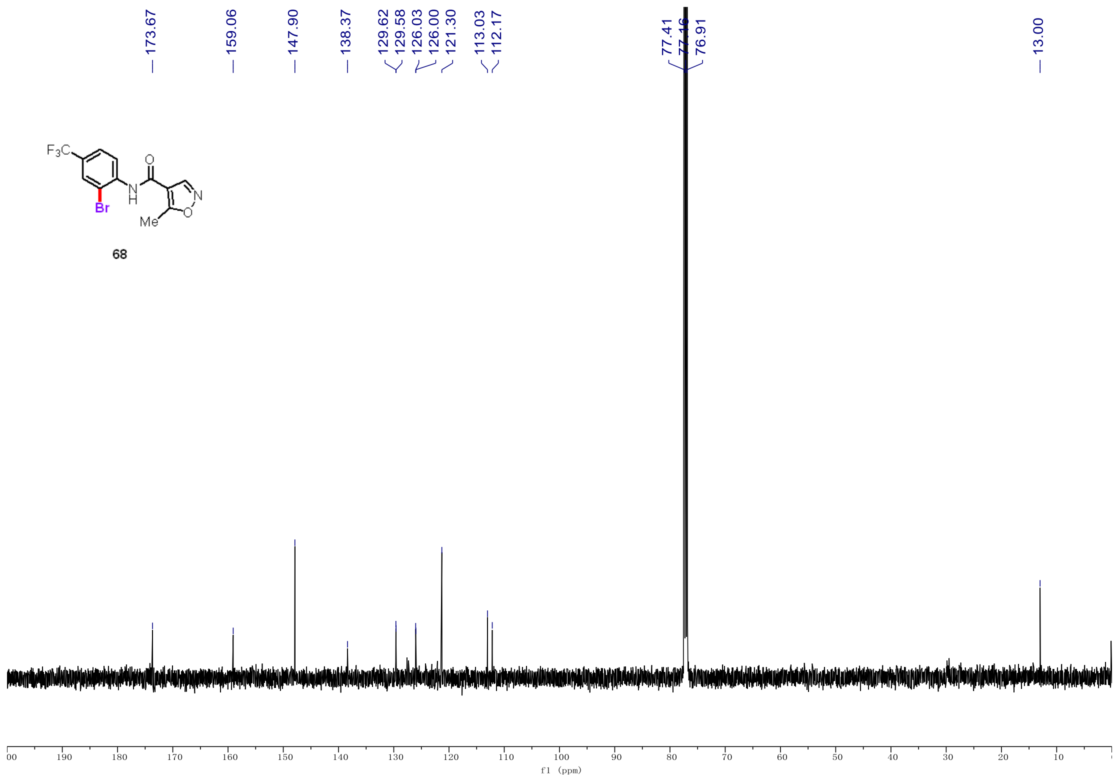

^{13}C NMR of compound **68**

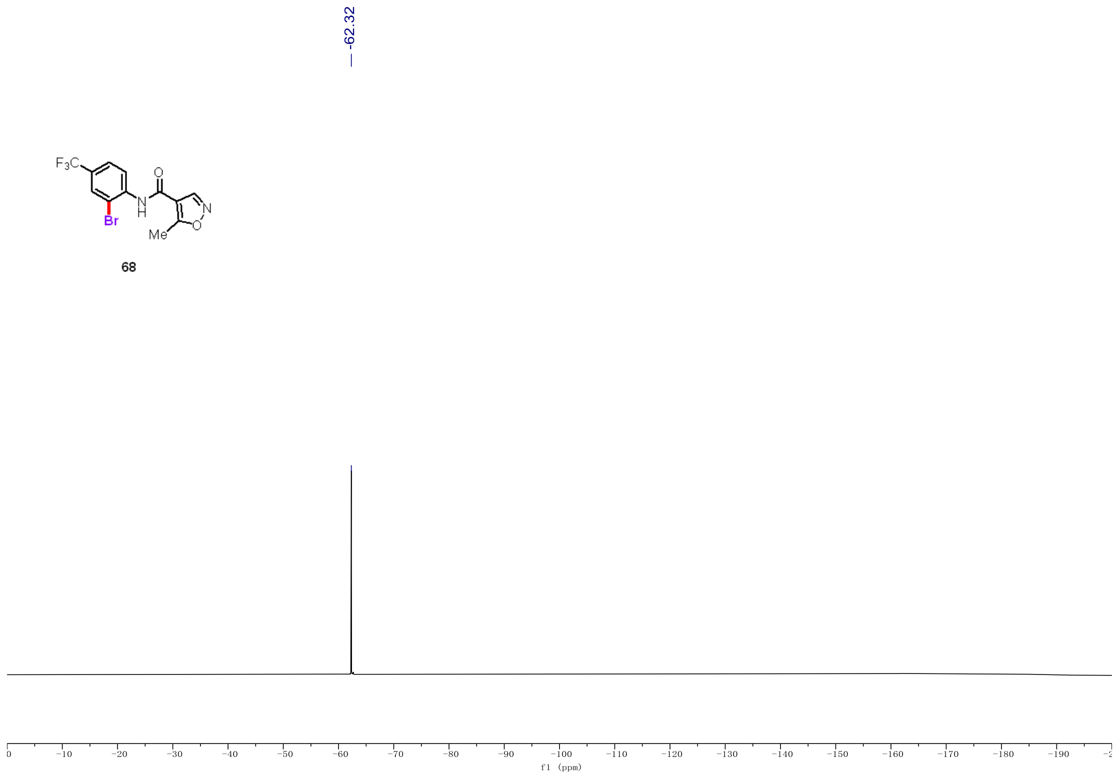

^{19}F NMR of compound **68**

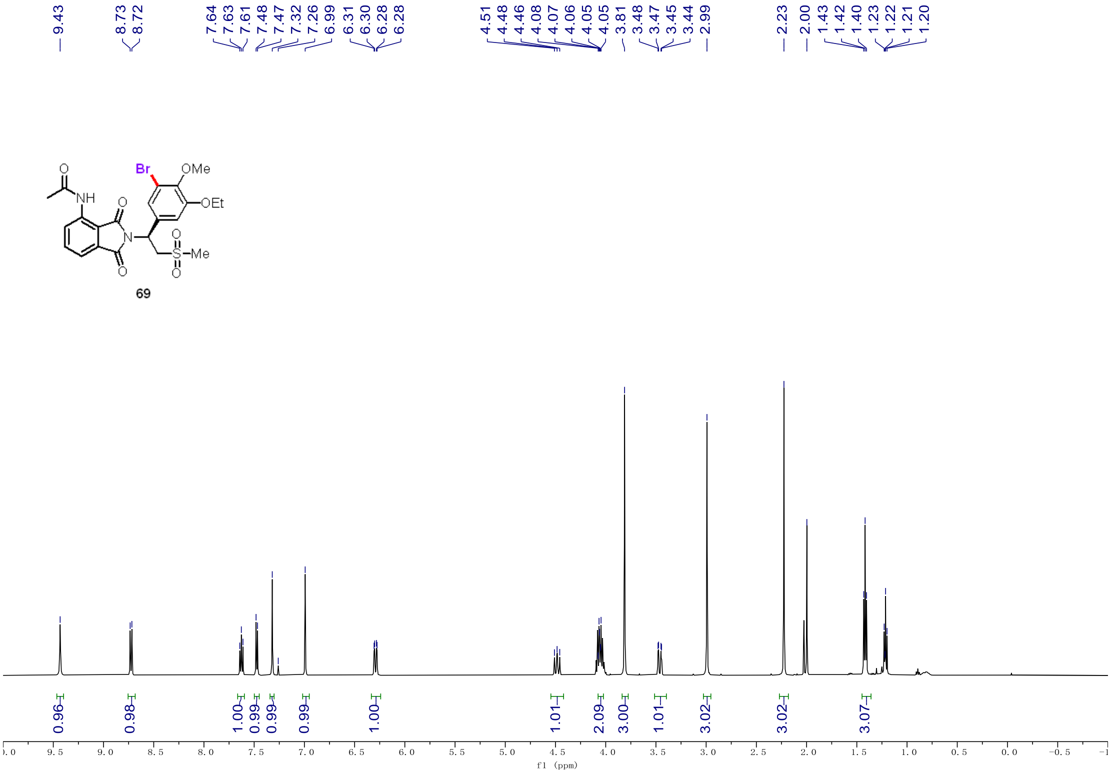

^{1}H NMR of compound **69**

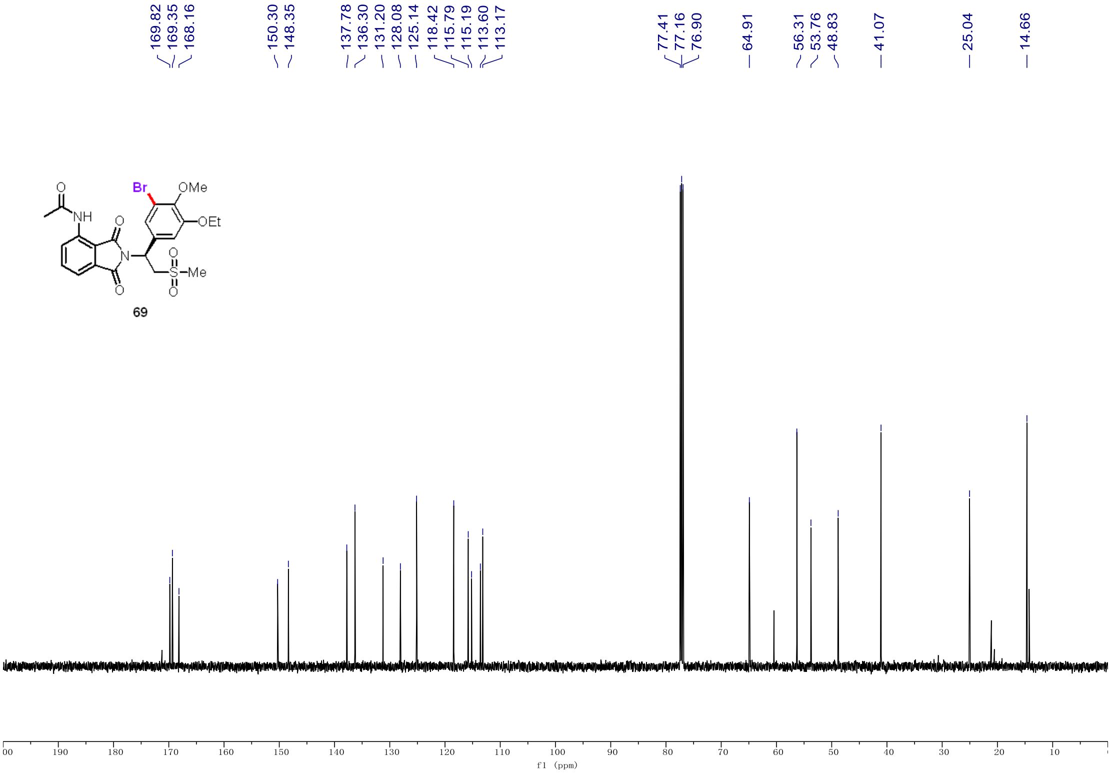

^{13}C NMR of compound **69**

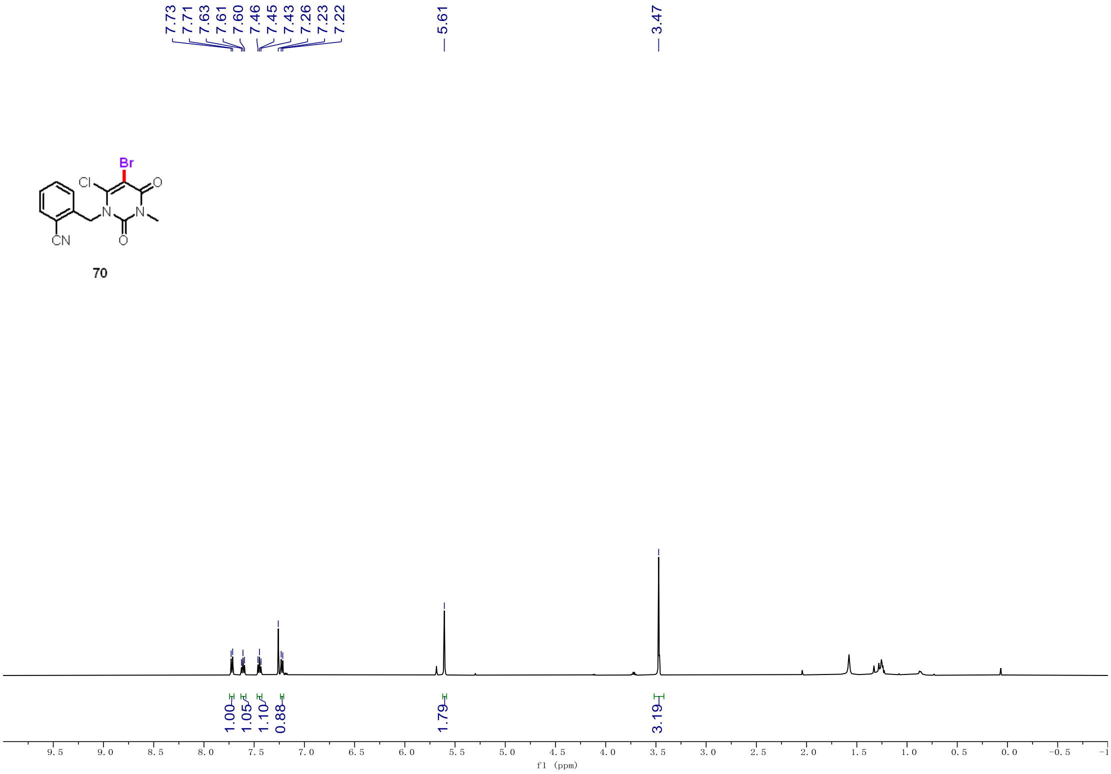

^{1}H NMR of compound **70**

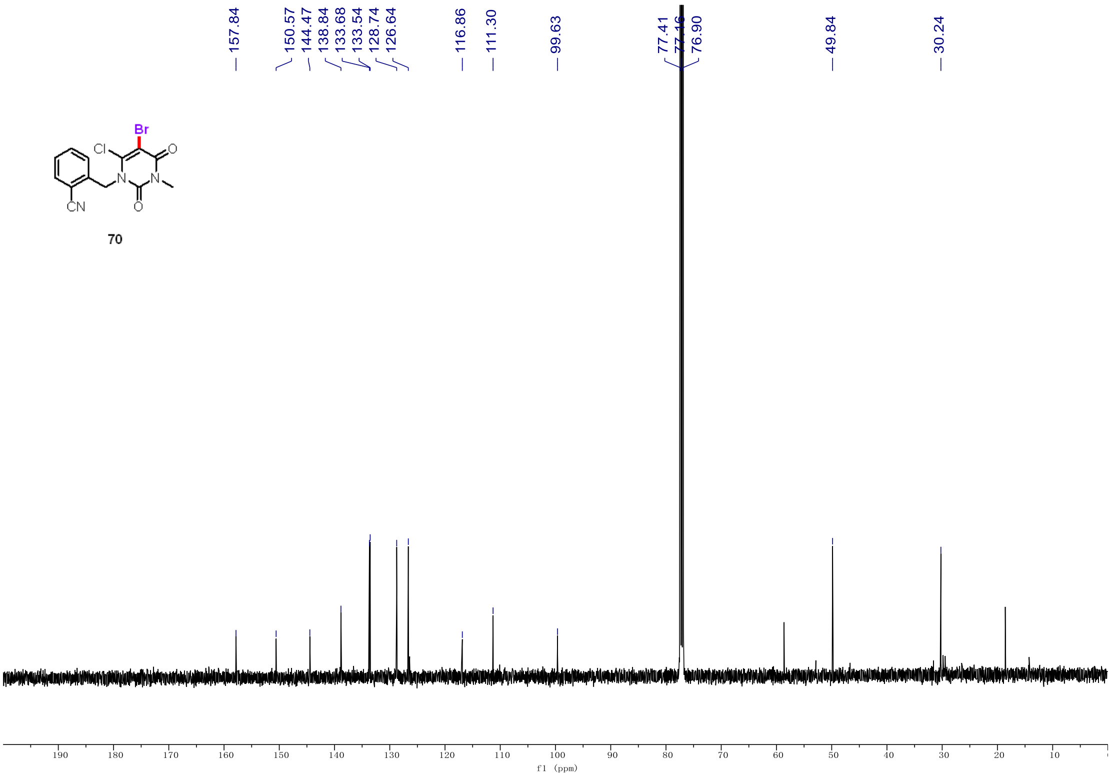

^{13}C NMR of compound **70**

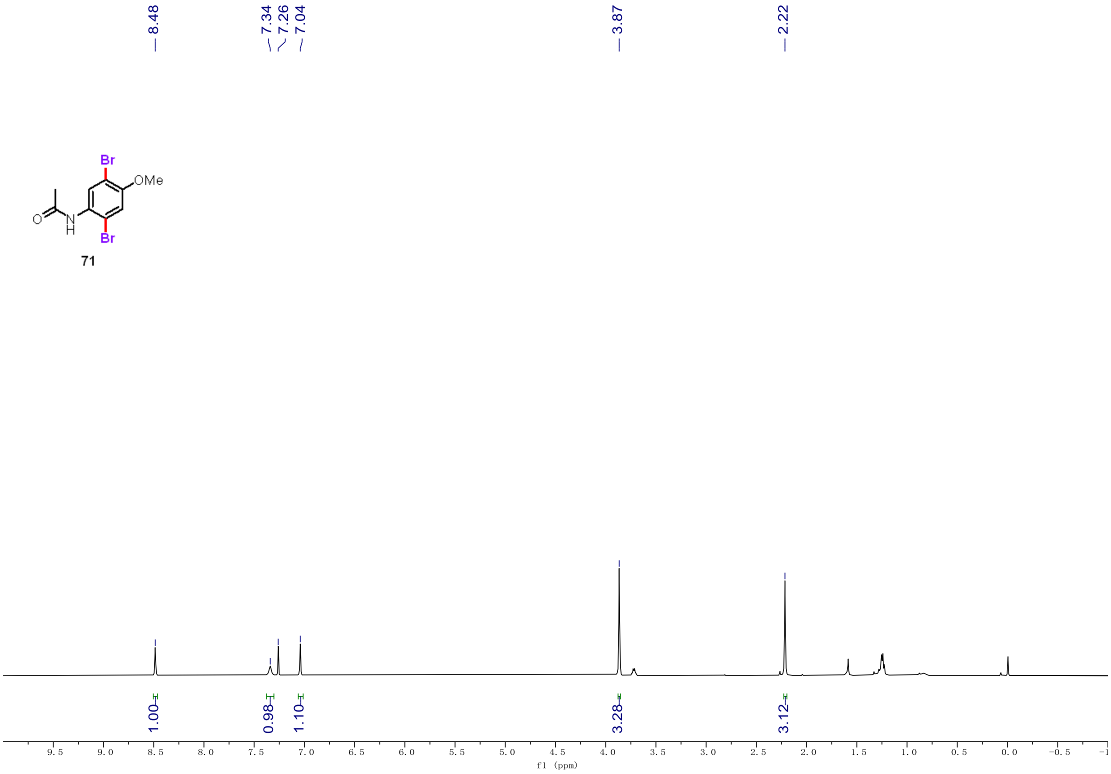

^{1}H NMR of compound **71**

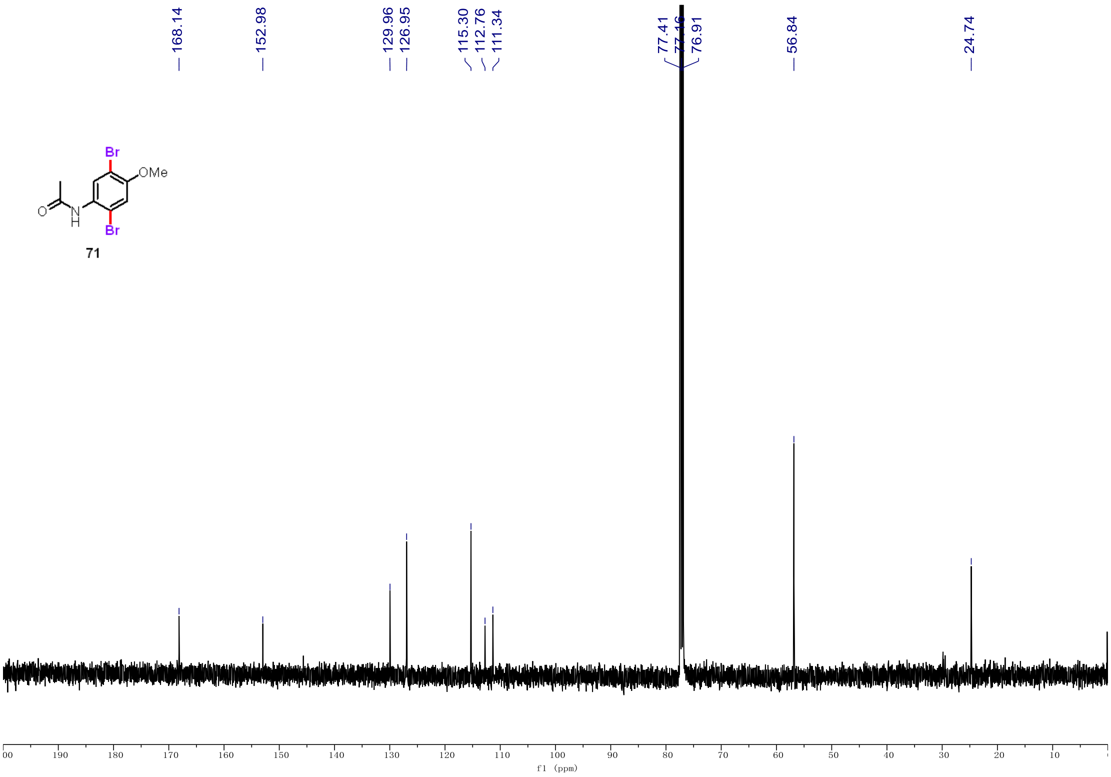

^{13}C NMR of compound **71**

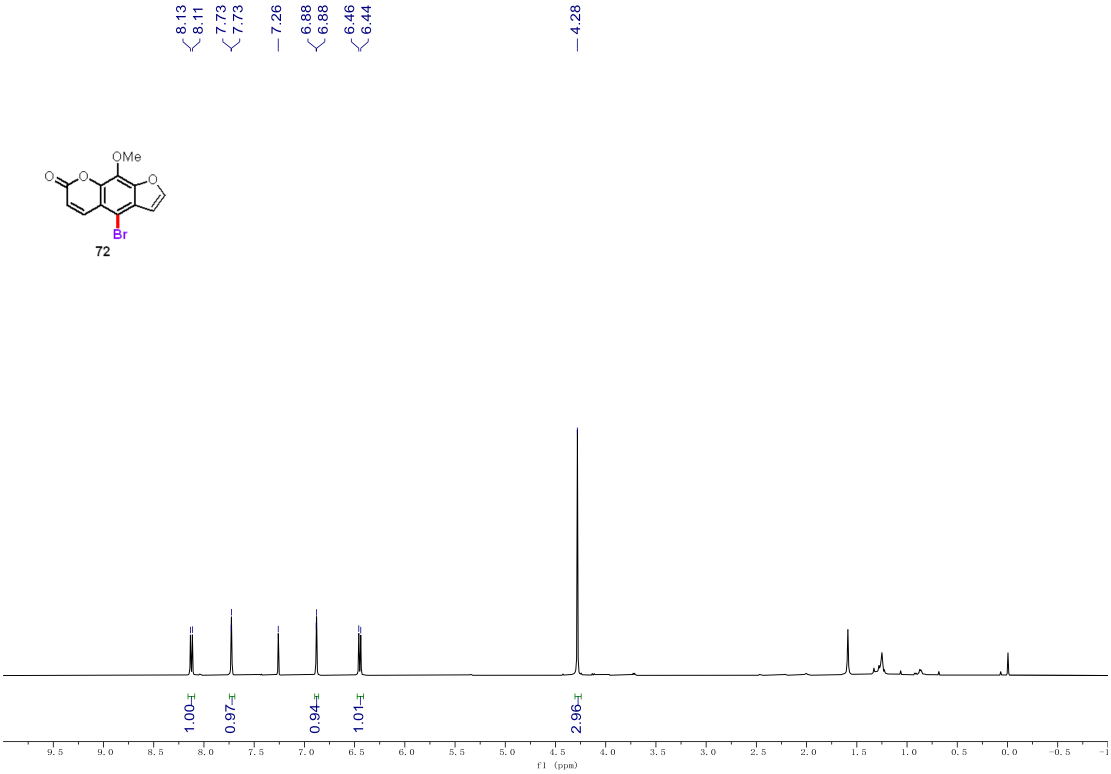

^{1}H NMR of compound **72**

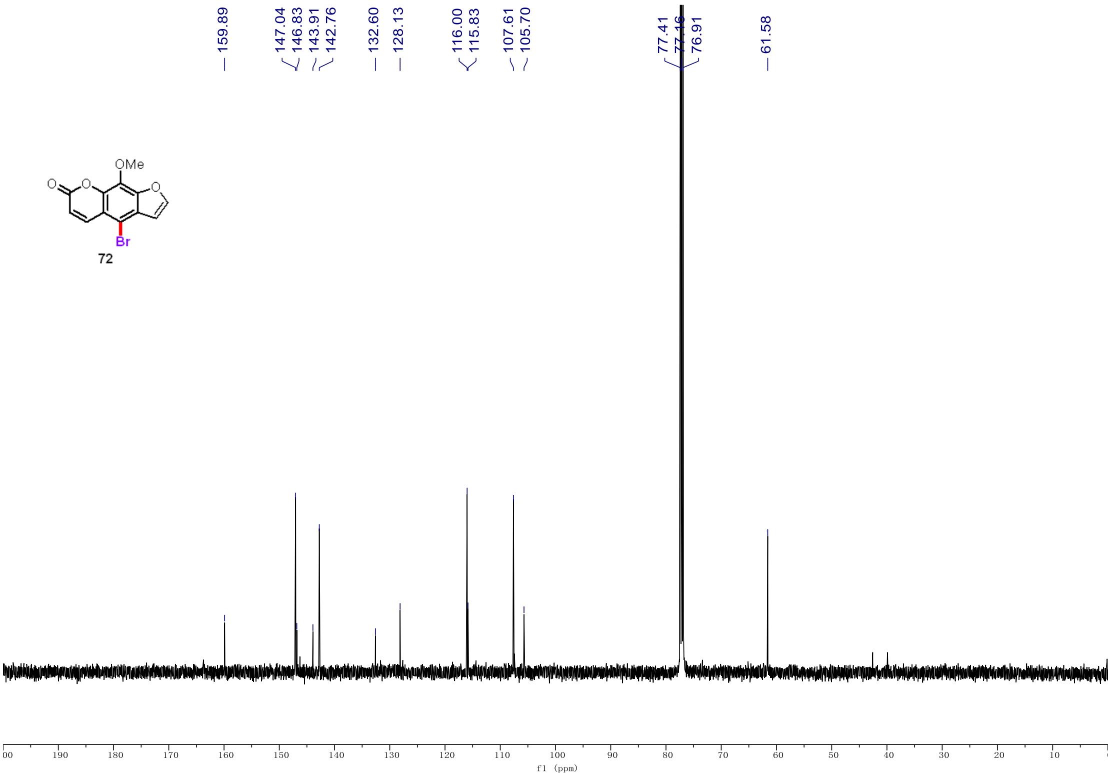

^{13}C NMR of compound **72**

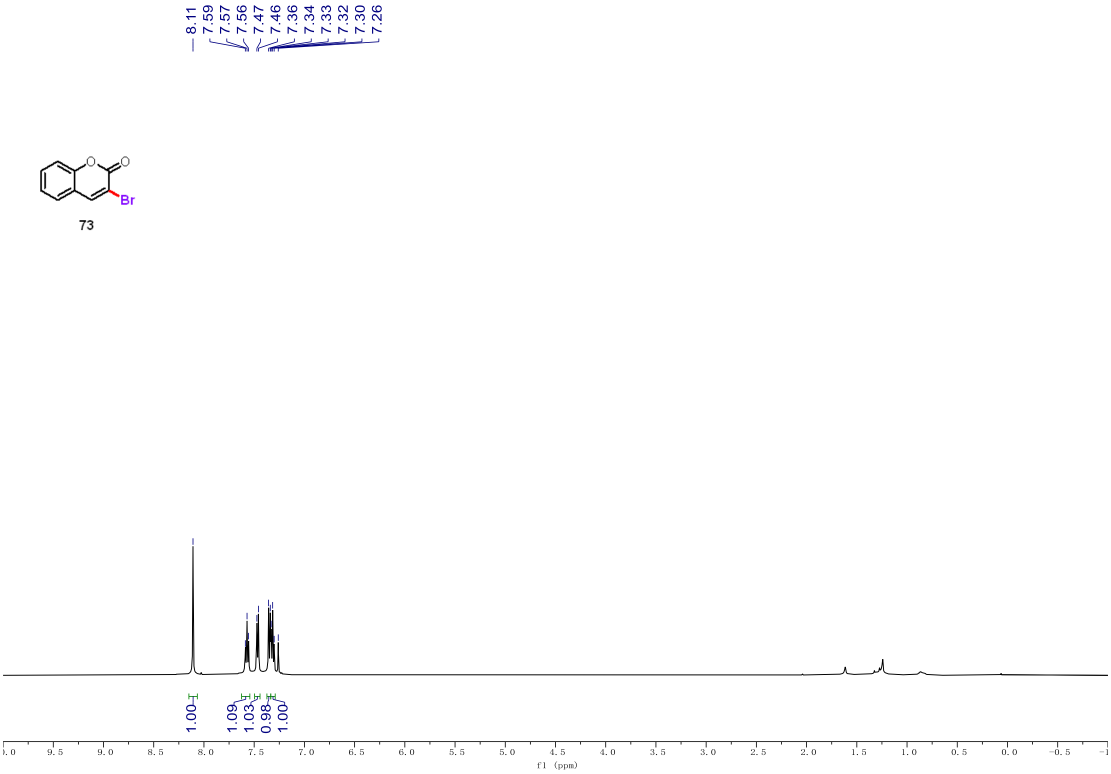

^{1}H NMR of compound **73**

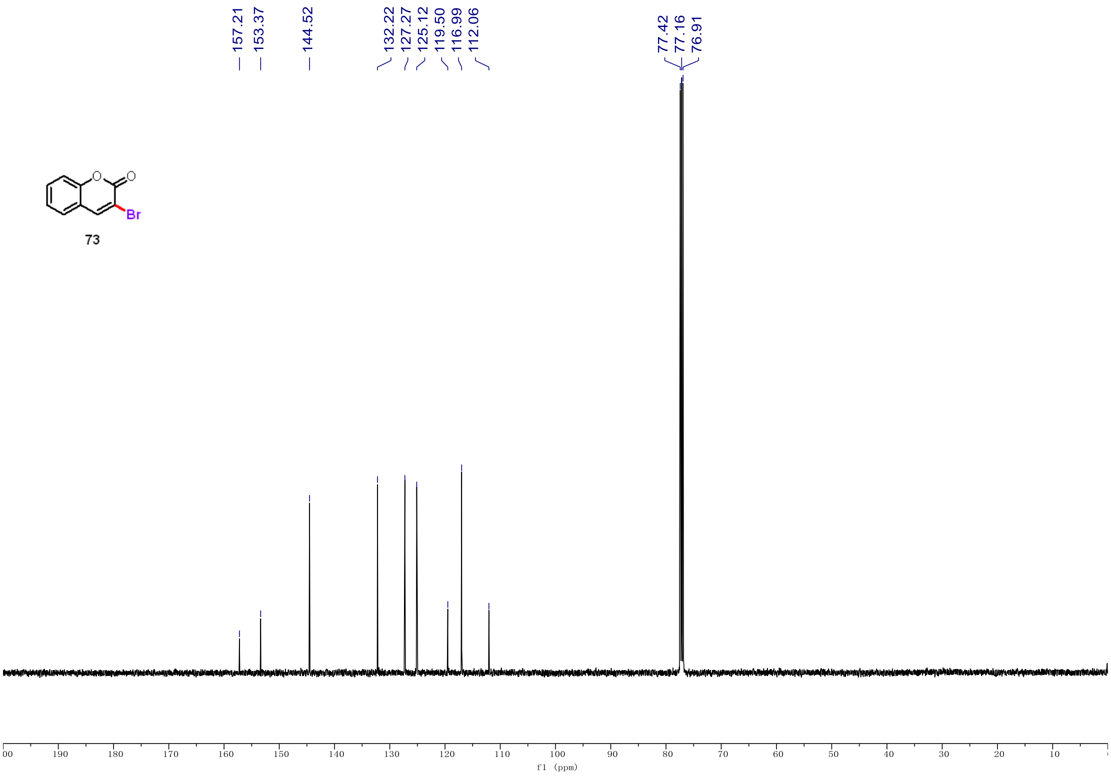

^{13}C NMR of compound **73**

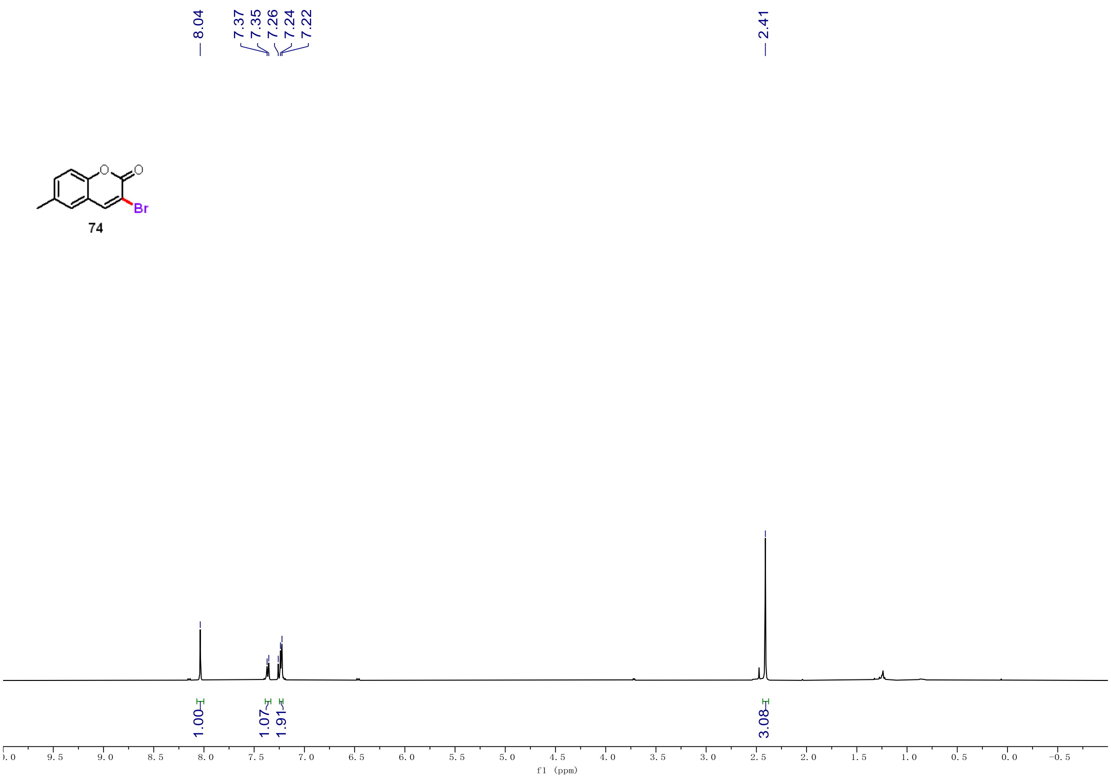

^{1}H NMR of compound **74**

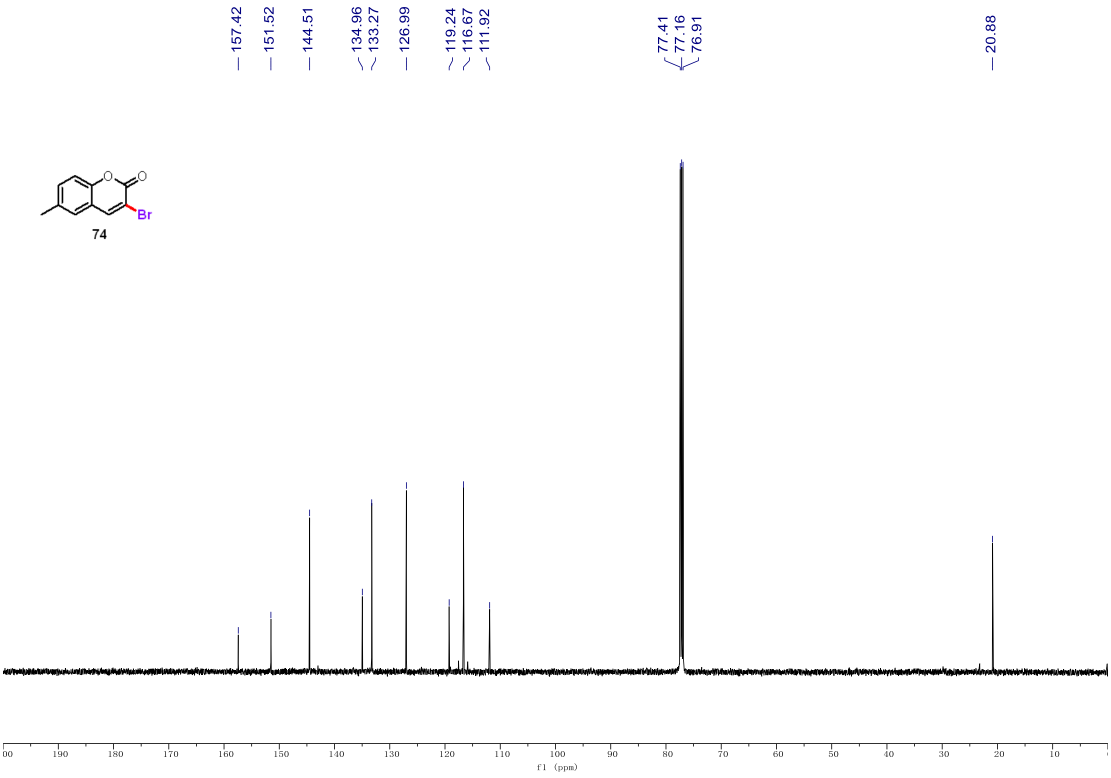

^{13}C NMR of compound **74**

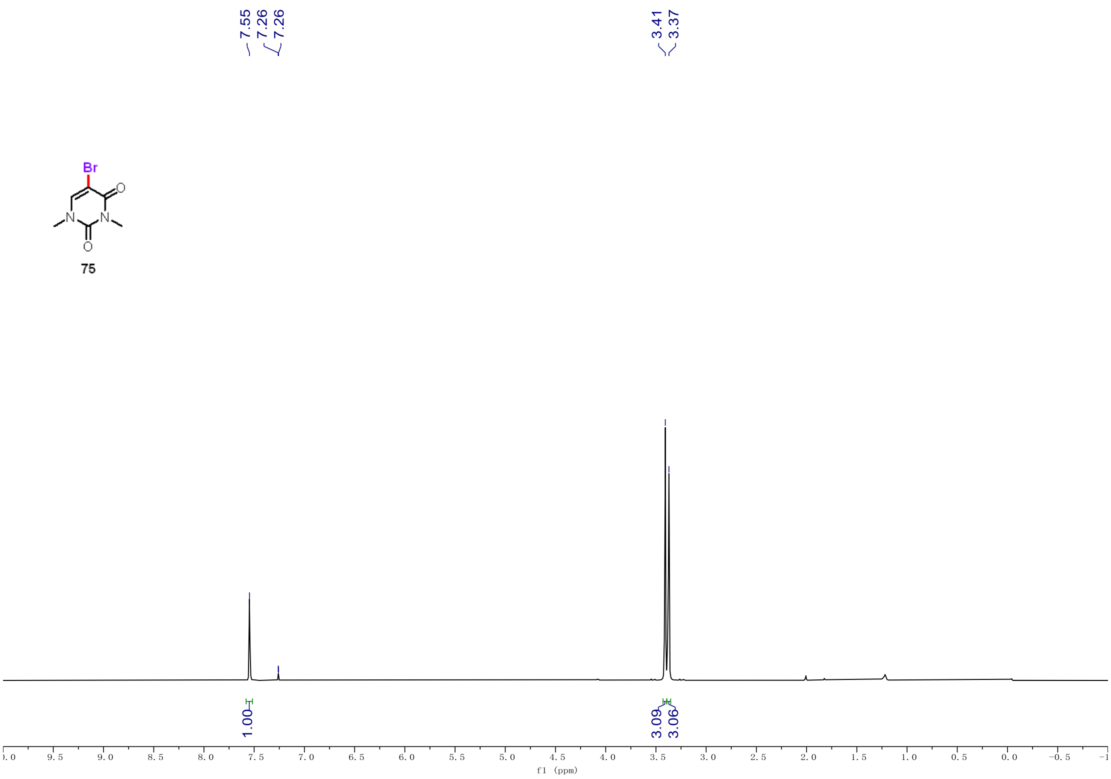

^{1}H NMR of compound **75**

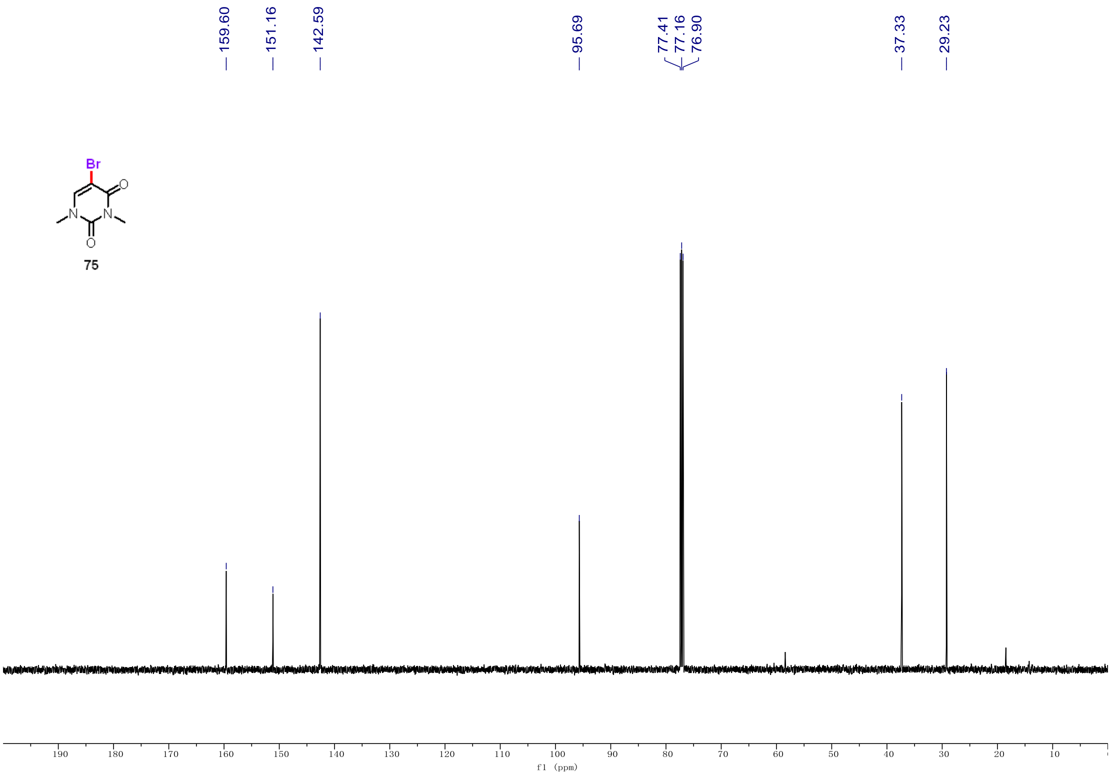

^{13}C NMR of compound **75**

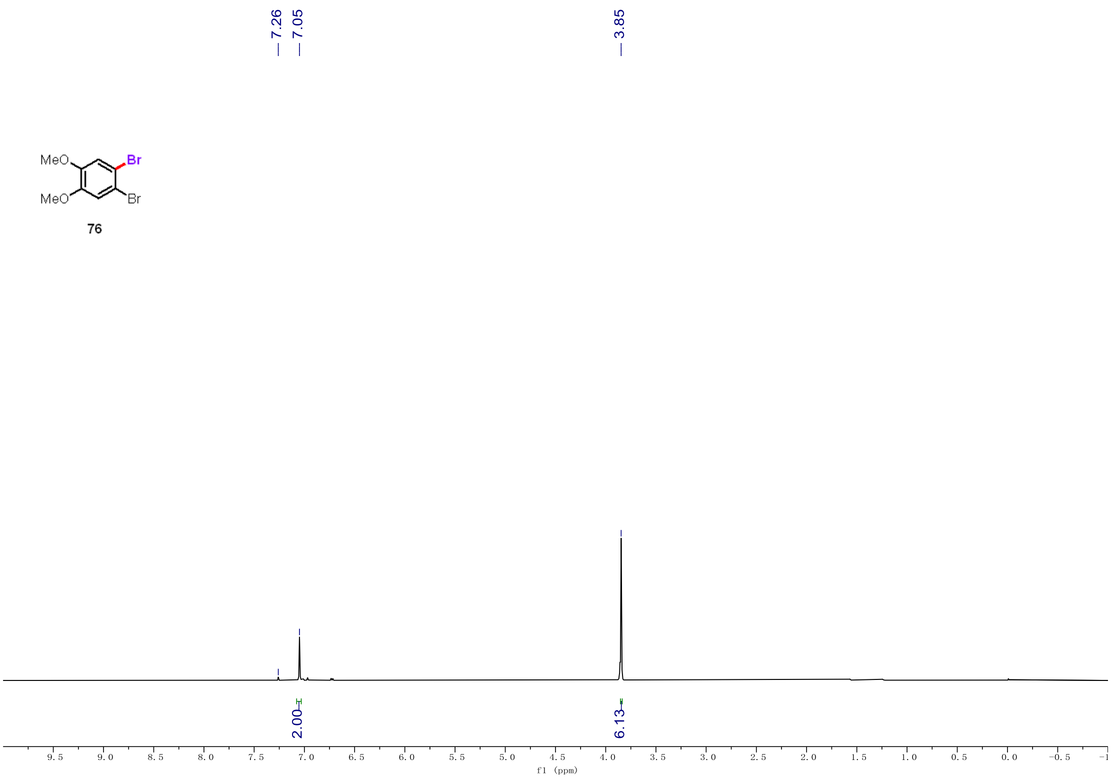

^{1}H NMR of compound **76**

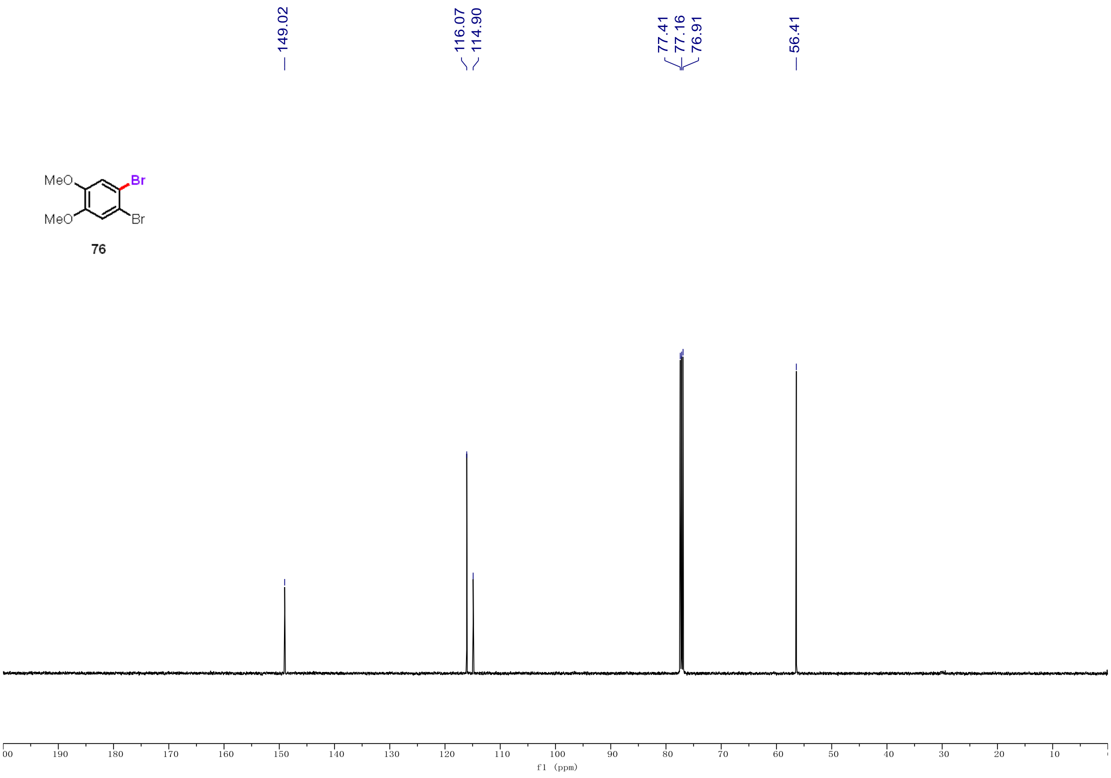

^{13}C NMR of compound **76**

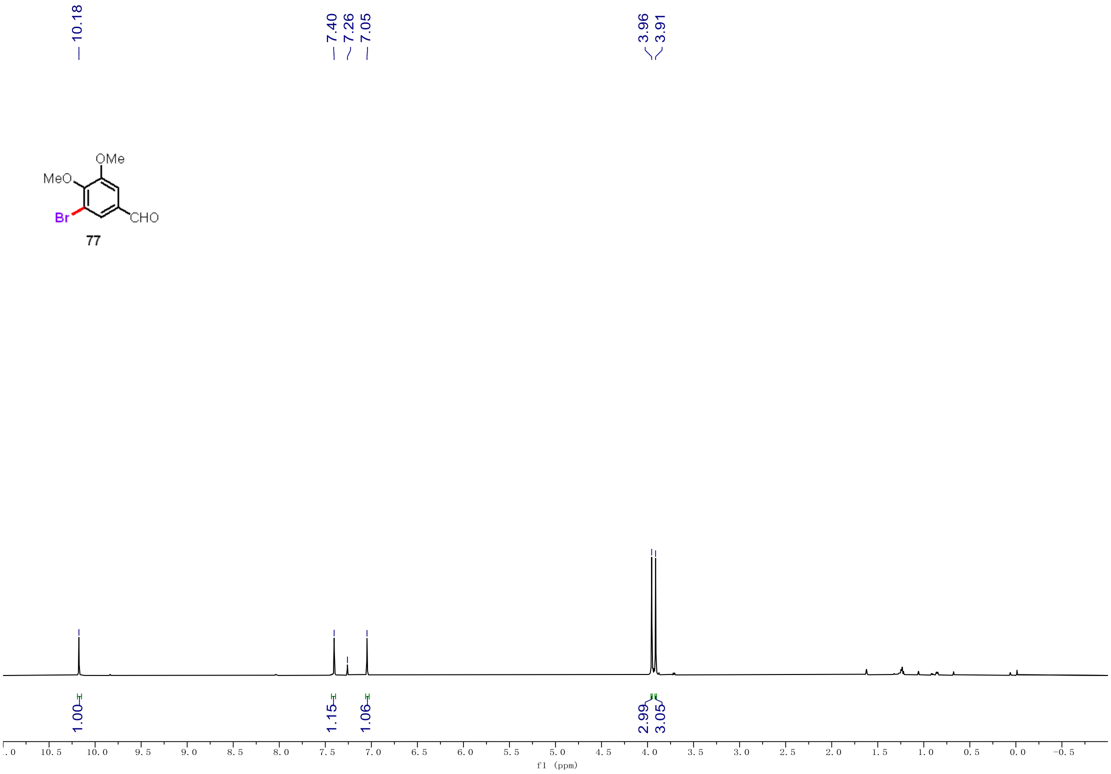

^{1}H NMR of compound **77**

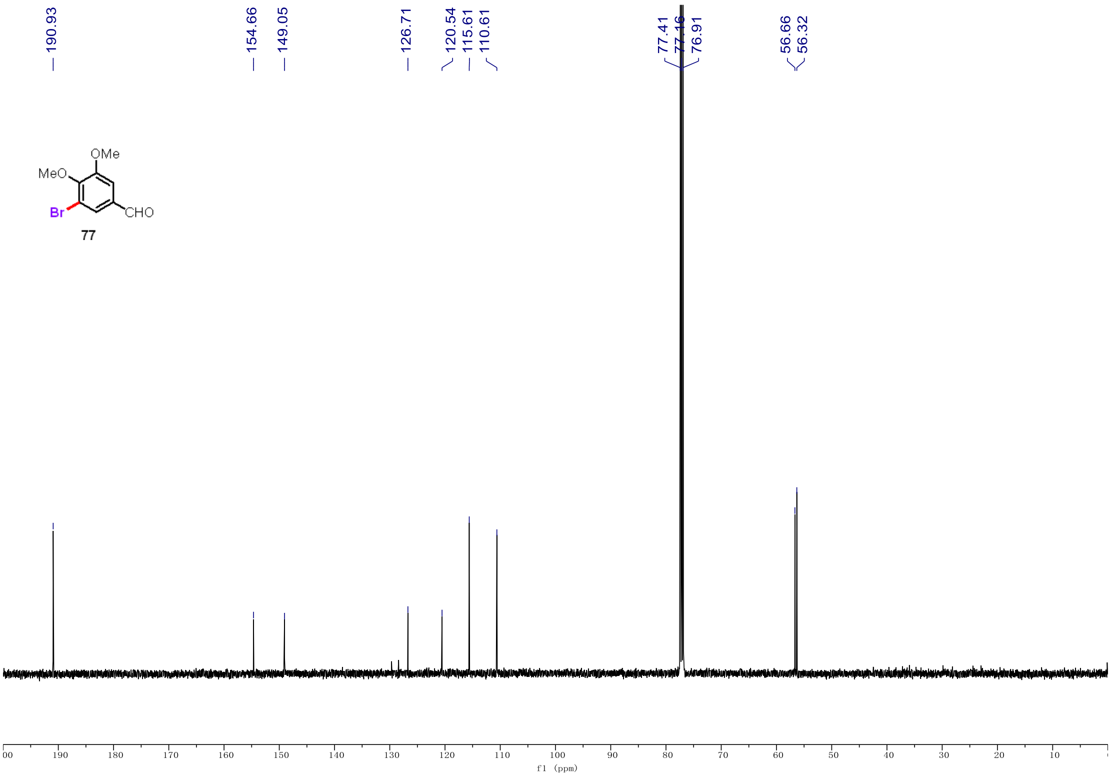

^{13}C NMR of compound **77**

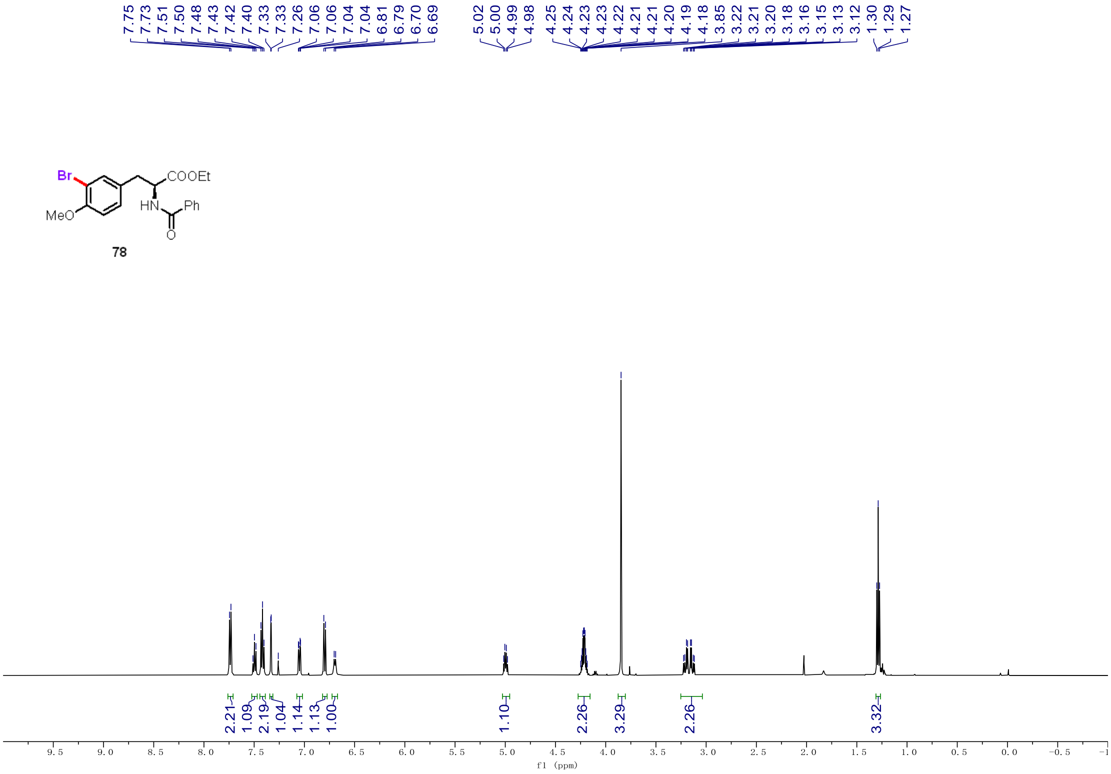

^{1}H NMR of compound **78**

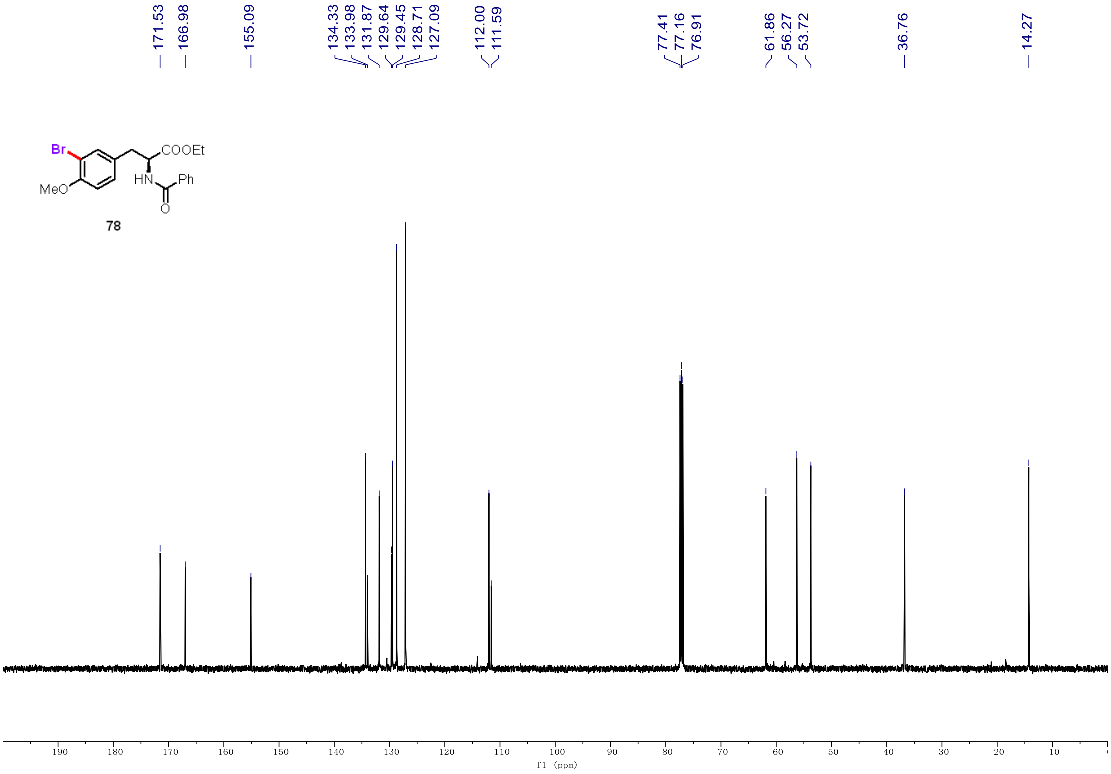

^{13}C NMR of compound **78**

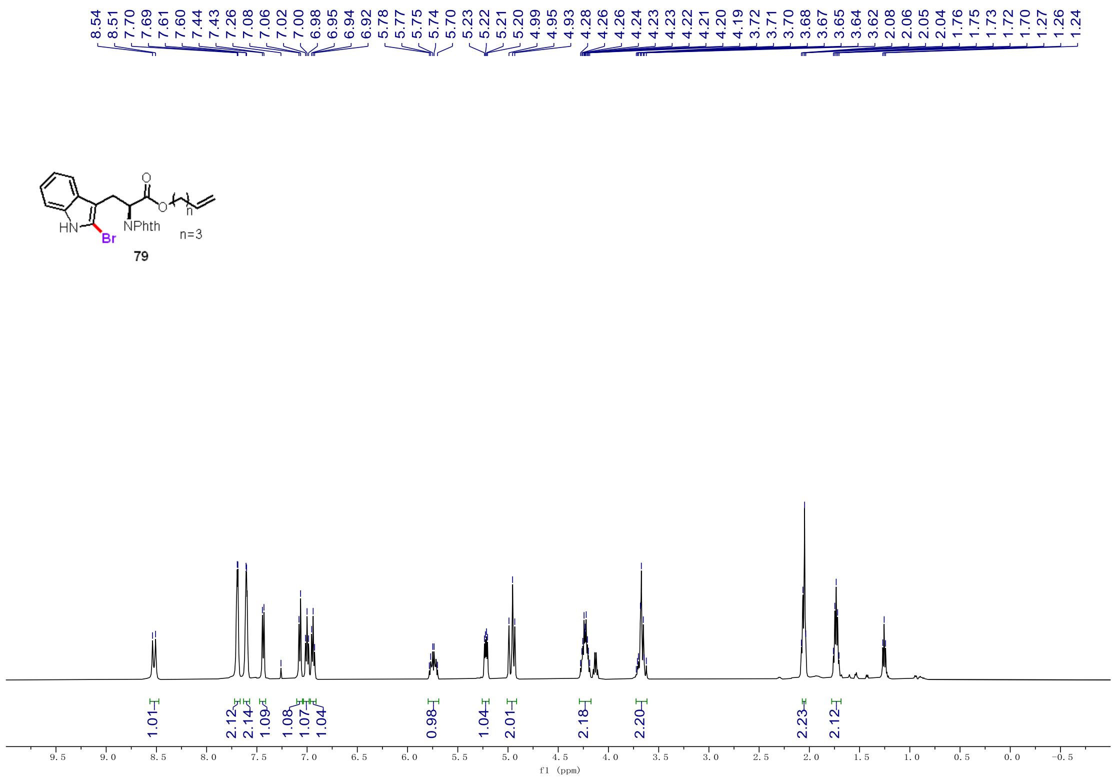

^{1}H NMR of compound **79**

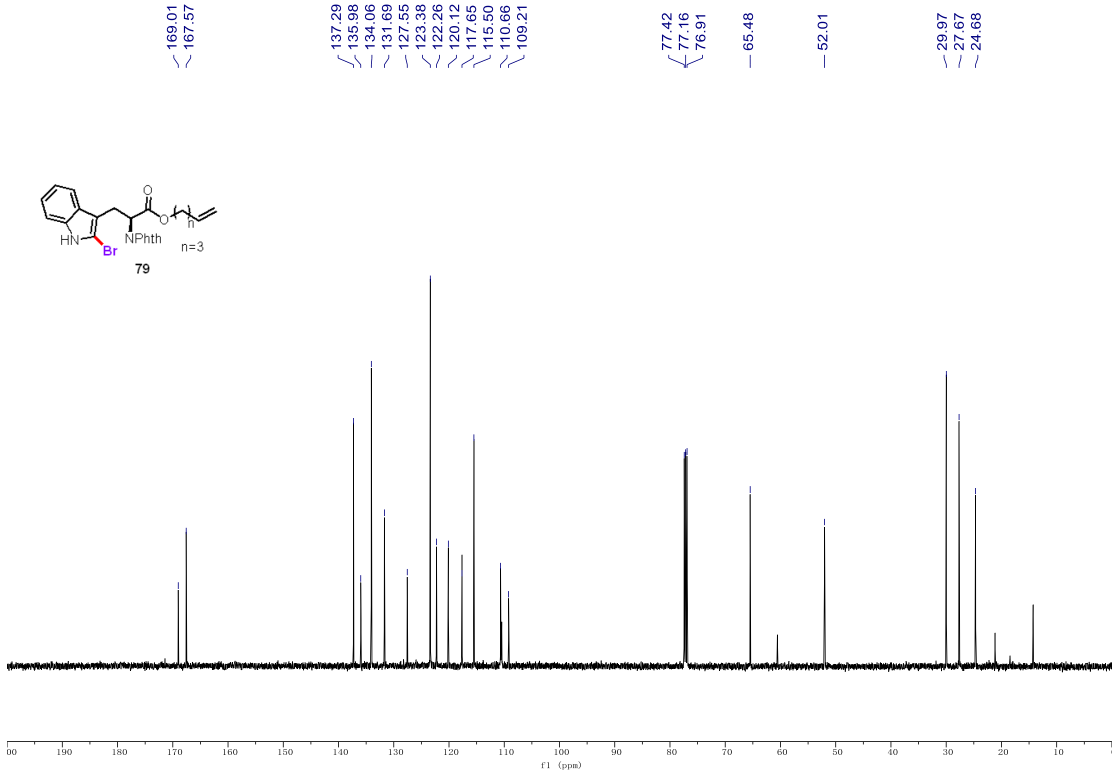

^{13}C NMR of compound **79**

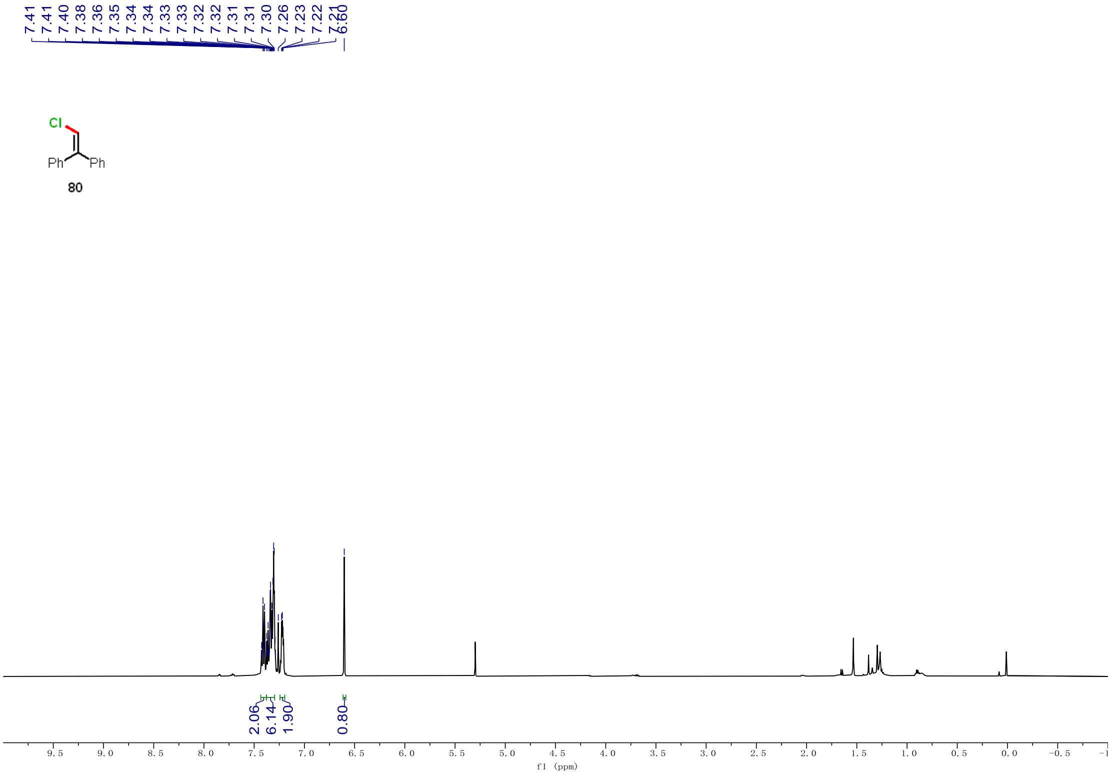

^{1}H NMR of compound **80**

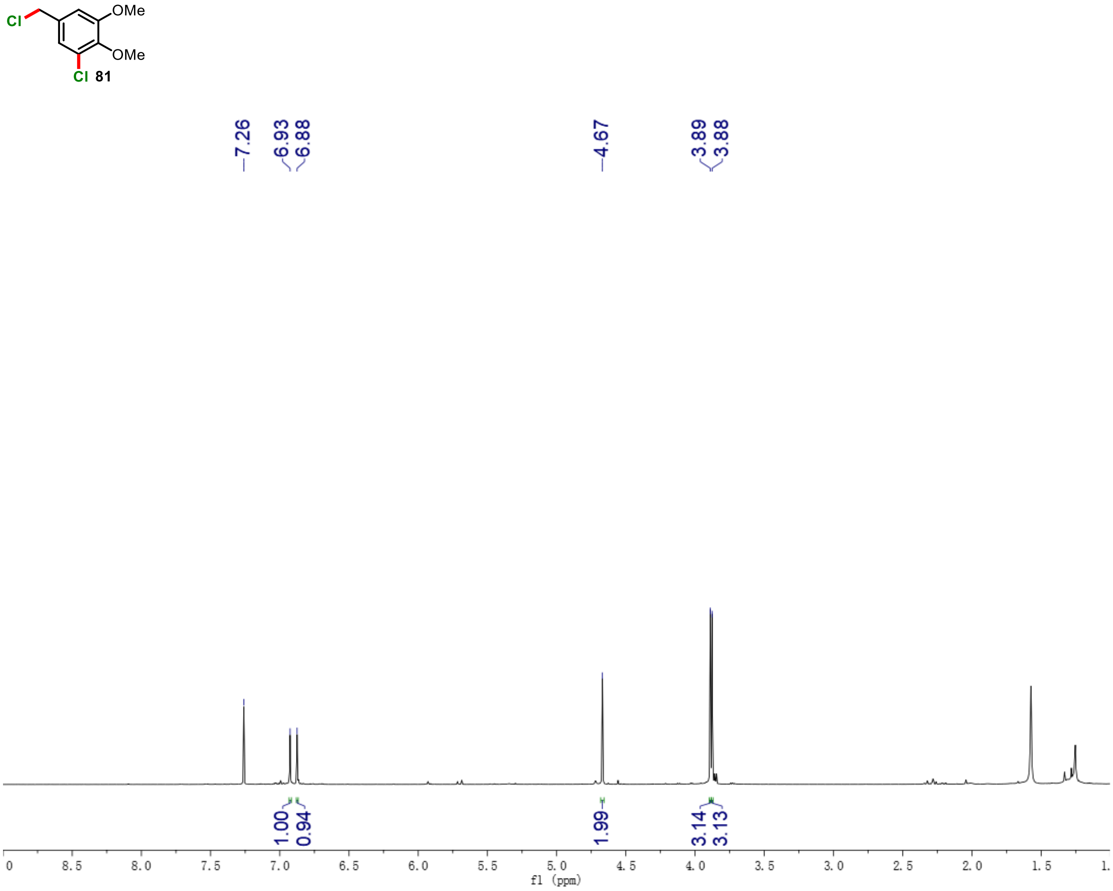

^{1}H NMR of compound **81**

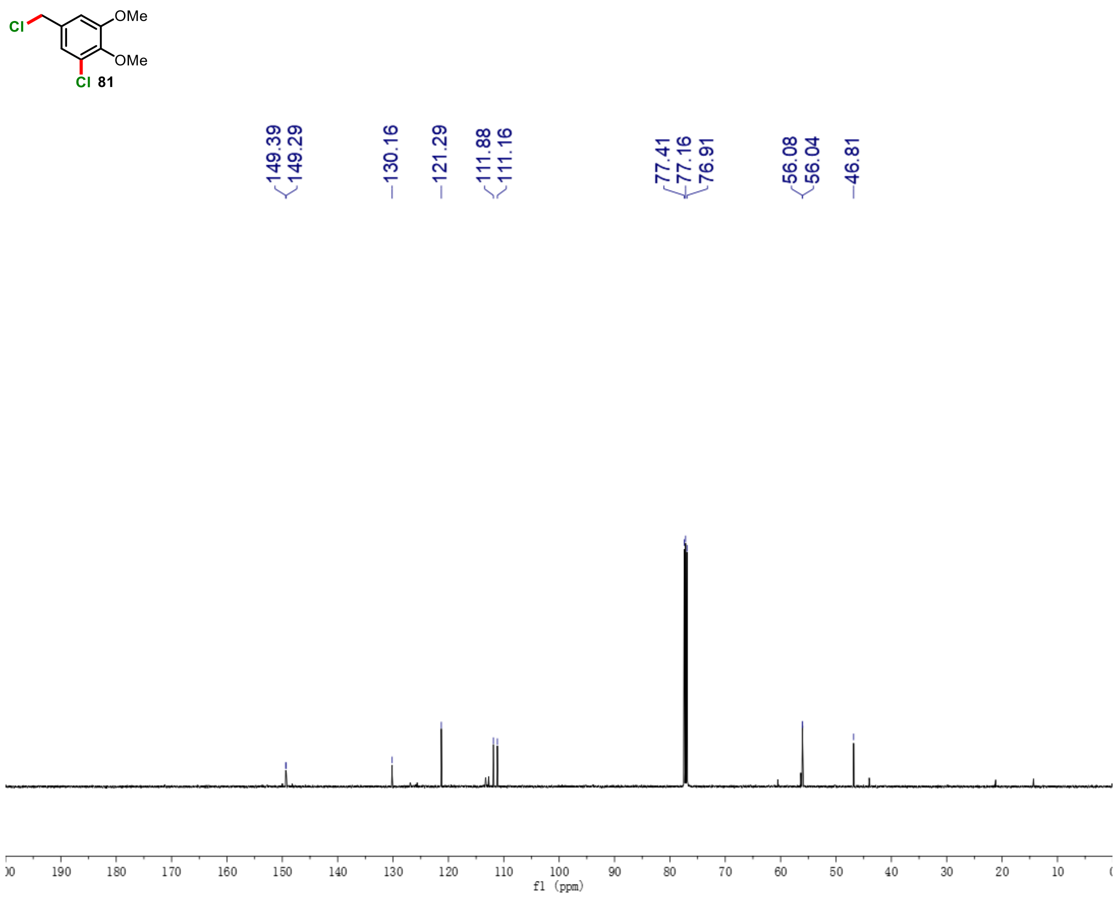

^{13}C NMR of compound **81**

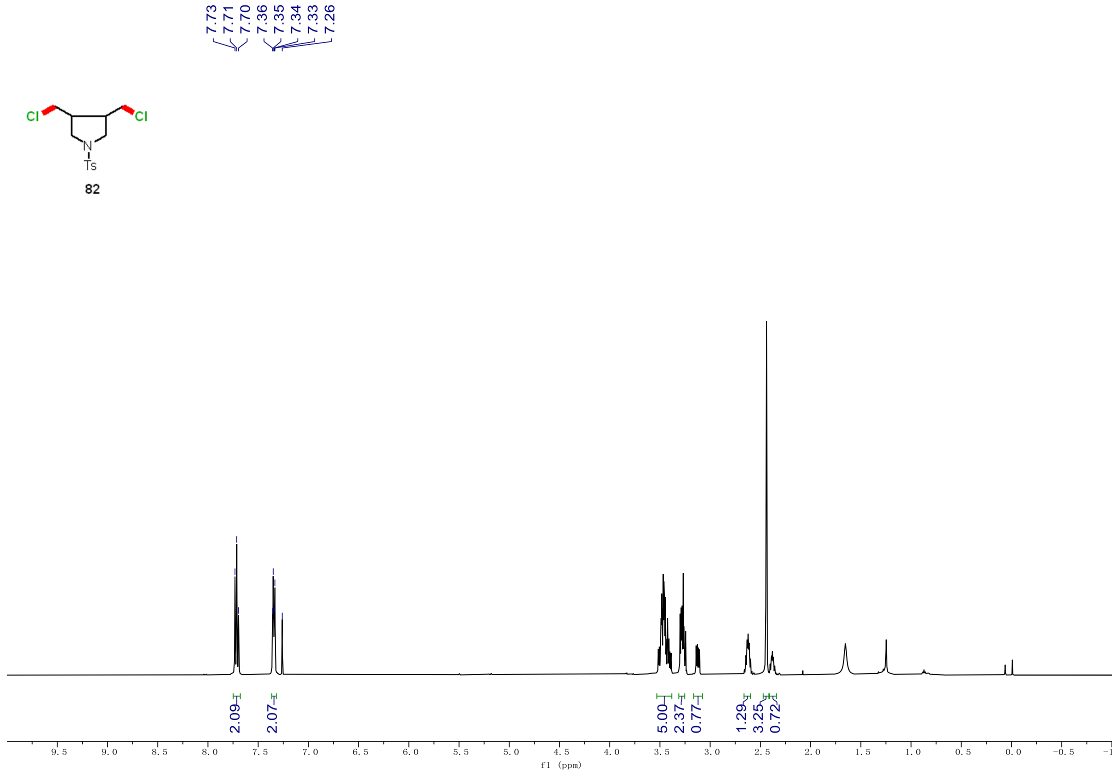

^{1}H NMR of compound **82**

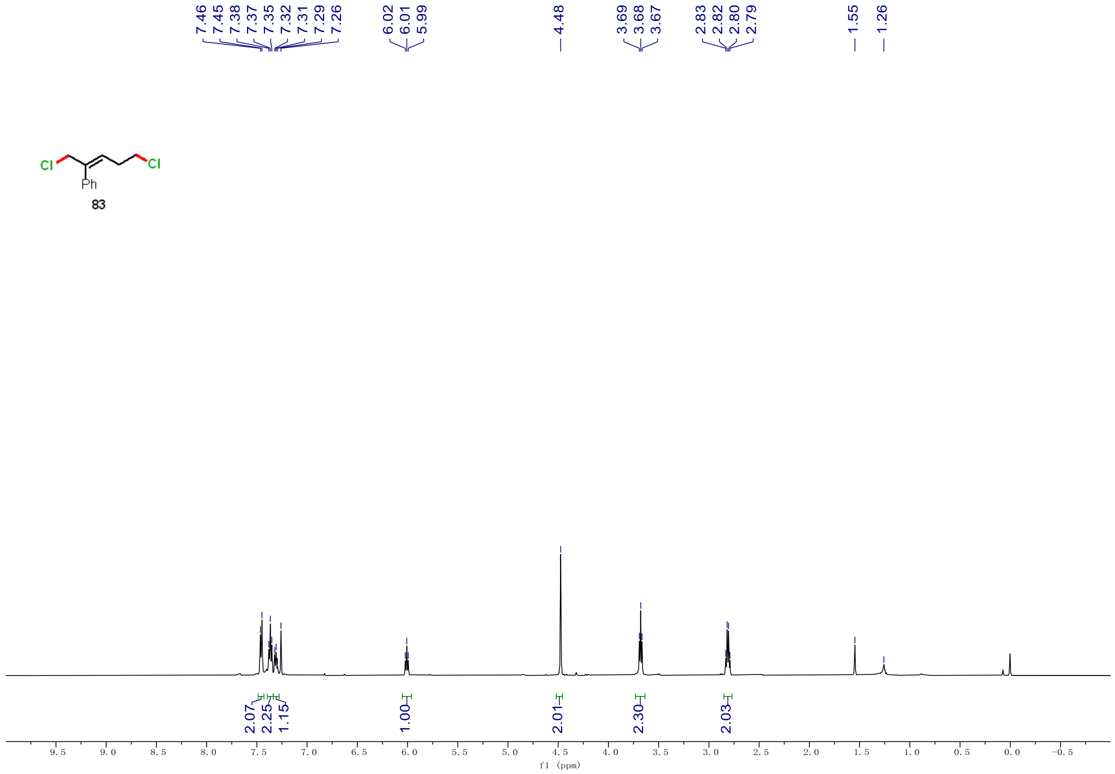

^{1}H NMR of compound **83**

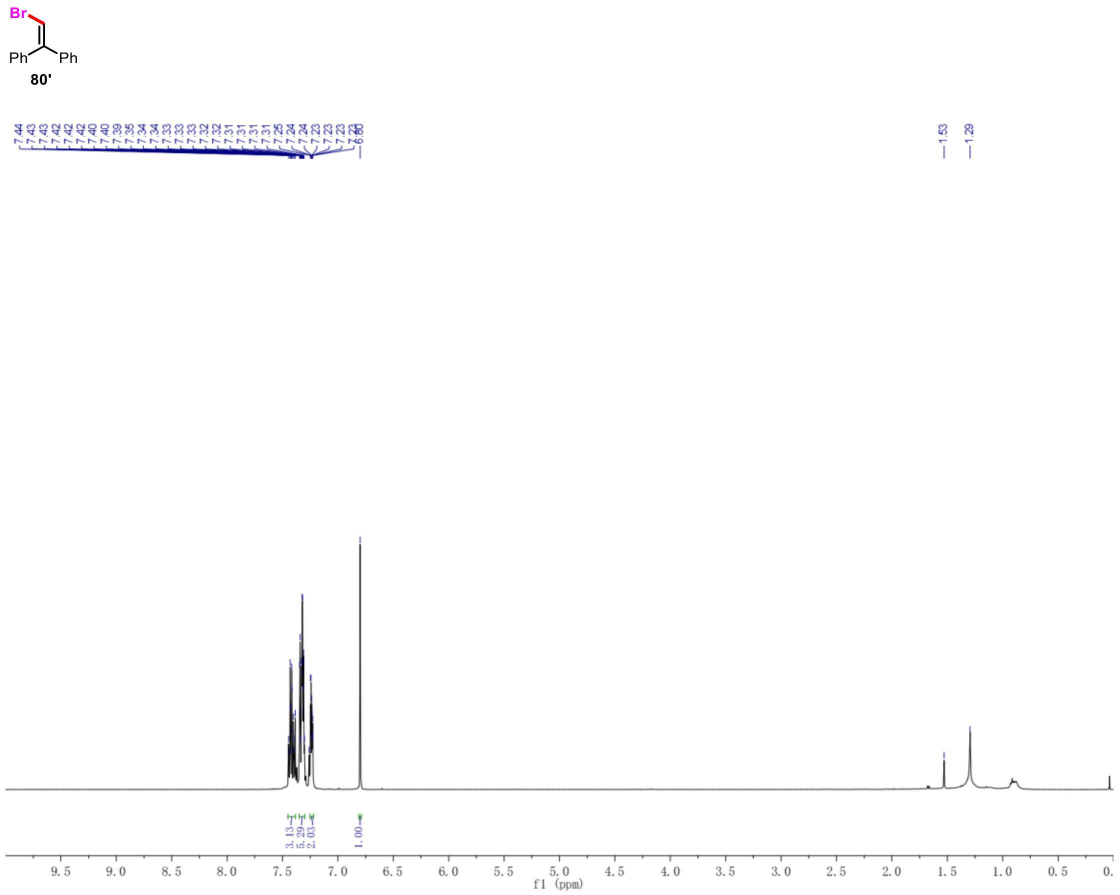

^{1}H NMR of compound **80’**

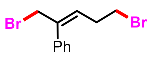

83'

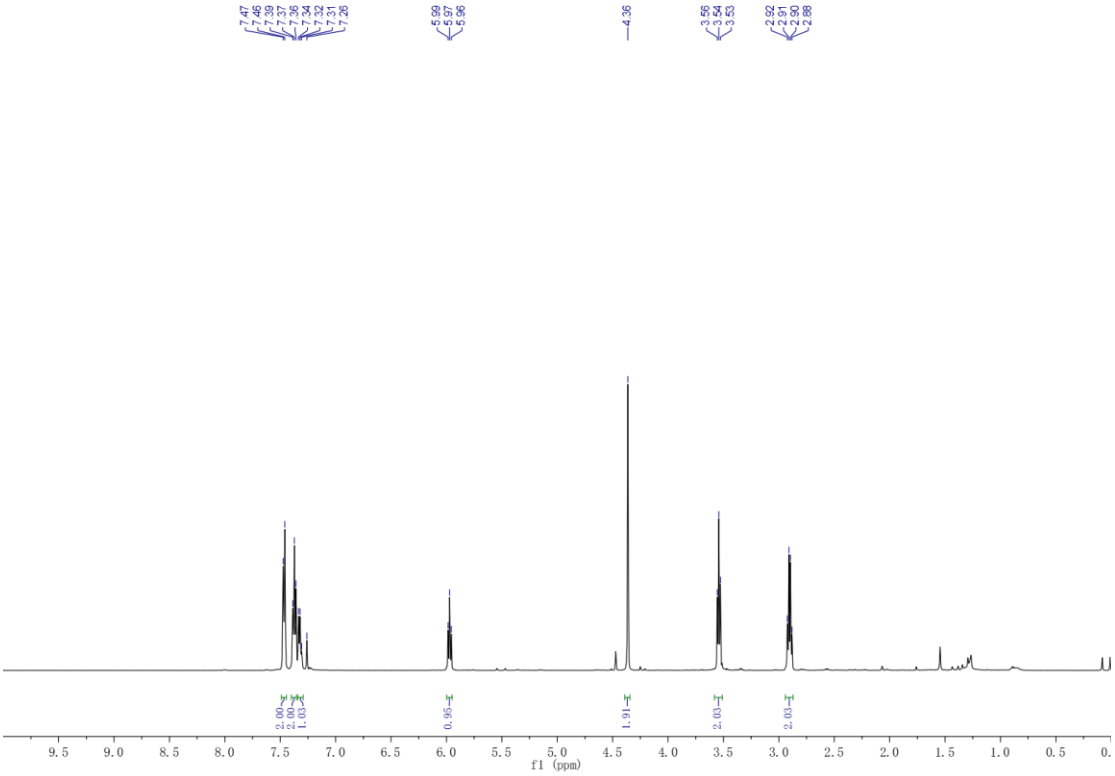

^{1}H NMR of compound **83’**

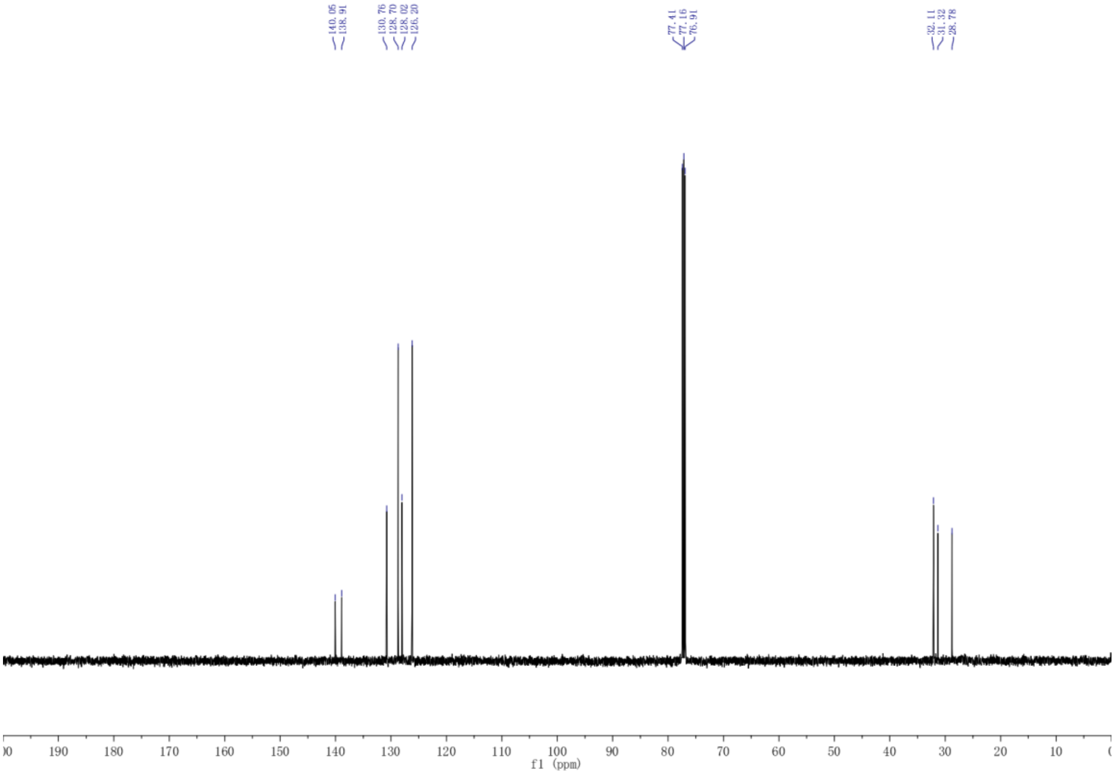

^{13}C NMR of compound **83’**
